# Proteome integral solubility alteration high-throughput proteomics assay identifies Collectin-12 as a non-apoptotic microglial caspase-3 substrate

**DOI:** 10.1038/s41419-023-05714-2

**Published:** 2023-03-11

**Authors:** Kathleen Grabert, Pinelopi Engskog-Vlachos, Martin Škandík, Guillermo Vazquez-Cabrera, Adriana-Natalia Murgoci, Lily Keane, Massimiliano Gaetani, Bertrand Joseph, Mathilde Cheray

**Affiliations:** 1 Institute of Environmental Medicine, Toxicology Unit, Karolinska Institutet, Stockholm, Sweden; 2 Chemical Proteomics, Division of Physiological Chemistry I, Department of Medical Biochemistry and Biophysics, Karolinska Institutet, Stockholm, Sweden; 3 Unit of Chemical Proteomics, Science for Life Laboratory (SciLifeLab), Stockholm, Sweden

**Keywords:** Cell biology, Neuroscience

## Abstract

Caspases are a family of proteins mostly known for their role in the activation of the apoptotic pathway leading to cell death. In the last decade, caspases have been found to fulfill other tasks regulating the cell phenotype independently to cell death. Microglia are the immune cells of the brain responsible for the maintenance of physiological brain functions but can also be involved in disease progression when overactivated. We have previously described non-apoptotic roles of caspase-3 (CASP3) in the regulation of the inflammatory phenotype of microglial cells or pro-tumoral activation in the context of brain tumors. CASP3 can regulate protein functions by cleavage of their target and therefore could have multiple substrates. So far, identification of CASP3 substrates has been performed mostly in apoptotic conditions where CASP3 activity is highly upregulated and these approaches do not have the capacity to uncover CASP3 substrates at the physiological level. In our study, we aim at discovering novel substrates of CASP3 involved in the normal regulation of the cell. We used an unconventional approach by chemically reducing the basal level CASP3-like activity (by DEVD-fmk treatment) coupled to a Mass Spectrometry screen (PISA) to identify proteins with different soluble amounts, and consequently, non-cleaved proteins in microglia cells. PISA assay identified several proteins with significant change in their solubility after DEVD-fmk treatment, including a few already known CASP3 substrates which validated our approach. Among them, we focused on the Collectin-12 (COLEC12 or CL-P1) transmembrane receptor and uncovered a potential role for CASP3 cleavage of COLEC12 in the regulation of the phagocytic capacity of microglial cells. Taken together, these findings suggest a new way to uncover non-apoptotic substrates of CASP3 important for the modulation of microglia cell physiology.

## Introduction

Caspases are a family of evolutionarily conserved intracellular cysteinyl proteases that recognize distinct tetrapeptide motifs and cleavage occurs after an aspartate residue in their substrates. Their role is mainly associated with cell death induction by apoptosis after activation of their cascade pathway. In the last decade, literature illustrates a switch in focus with the investigation of the potential functions of caspases beyond cell death [1–3]. Indeed, caspases are reported to be involved in multiple biological processes, including cell remodeling and migration as well as cell differentiation and proliferation [2].

Caspase-3, encoded by the *CASP3* gene, is probably best known for its role in the execution of apoptosis because of its functions in promoting the destruction of cellular structures such as DNA fragmentation or degradation of cytoskeletal proteins [4]. CASP3 has therefore been classified in nomenclature as a so-called apoptotic caspase [5, 6]. However, it should be noted that CASP3 is reported to exert biological functions which are not related to cell death; this is particularly true in the central nervous system (CNS) [7]. For example, early reports indicate that during postnatal development of the cerebellar cortex, active CASP3 is observed in non-apoptotic, proliferating, and differentiating neuronal cells [8]. Likewise, Bergmann glia use CASP3 activation for their differentiation in the postnatal cerebellum [9]. CASP3 is also involved in myeloid cell differentiation where it cleaves proteins that may prepare to expel mitochondria [10]. Several studies have implicated CASP3 in the regulation of synaptic plasticity (reviewed in [11]). Its activation is further required for long-term synaptic depression and AMPA receptor internalization in mouse hippocampal neurons [12, 13]. The importance of CASP3 is not only evident in mammals, as it is also essential for proper development of the auditory brainstem in chickens [14, 15]. A role for CASP3 activity localized to postsynaptic terminals within the auditory forebrain, is even described in the phenomenon of song-specific habituation in adult zebra finch [16]. Finally, the fine-tuned regulation of non-apoptotic CASP3 activity has been linked with neurodegenerative disorders such as Alzheimer´s disease (AD) or Huntington´s disease (HD) [17, 18].

Microglia are the immune resident cells of the CNS and constantly survey the brain environment by using their surface receptors to detect any brain damage or infection. Microglia can be potent immune effector cells, able to perform a broad range of functions, and mediate both innate and adaptive responses during CNS injury and disease, while remaining quiescent at steady state. As in other cell types, caspases have been shown to regulate microglia functions and cellular phenotypes acquisition (recently reviewed by us in [19]). Microglia cells are crucial for brain homeostasis, yet uncontrolled or over-activated microglia can contribute to brain diseases with contrasting effects, promoting neuronal dismiss in the context of AD and Parkinson’s disease (PD) or turning into pro-tumoral cells in the context of brain tumors, such as glioblastoma (GBM). In fact, there is increasing evidence of the non-apoptotic role of caspases in regulating the microglia phenotype in the context of diseases. Non-apoptotic caspase-3 and capase-8 activation in microglia is observed in the ventral mesencephalon of (PD) and the frontal cortex of (AD) patients or in tissues of ischemic stroke patients. Here, pro-inflammatory stimuli induce the orderly activation of caspase-8 (CASP8) and thereafter CASP3 in microglial cells. In turn, active CASP3 promotes, through a PKCδ-dependent pathway, the pro-inflammatory activation of microglia [20–22]. The inhibition of this caspase-dependent signaling pathway hinders the pro-inflammatory phenotype of microglia and associated neurotoxicity. In addition, we previously reported that a reduction in the basal activity of microglial CASP3 is part of their activation process in the context of GBM brain neoplasm. Indeed, glioma-induced microglia activation toward a tumor supporting phenotype is coupled to a reduction of basal microglial CASP3 activity, through thioredoxin-2-mediated S-nitrosylation of the enzyme. This demonstrated that CASP3 inhibition regulates microglial pro-tumoral functions [23]. Hence, we and others uncovered that a modest, yet significant, DEVDase proteolytic enzymatic activity (reflecting the presence of active CASP3) can be found in unstimulated microglia. In addition, regulation of this low CASP3 activity impacts on the activation of microglia towards distinct reactive states.

Based on the role of the fine-tuned regulation of CASP3 activity in the control of microglia polarization, uncovering which protein substrates can be regulated by the modest CASP3 enzymatic activities should contribute to a better understanding of signaling pathways controlling microglial reactive states. We therefore aimed at identifying new CASP3 substrates in microglia. Recent proteomics approaches which have allowed the detection of caspase substrates upon apoptosis have advanced in the field [24, 25], however those studies remain restricted to the cell death context. To date, most screening used to uncover novel CASP3 substrate candidates, have relied on a proteomic screen for proteolytic products performed on protein extracts collected directly from apoptotic cells, or protein extracts exposed to recombinant CASP3 [26–29]. None of the above-mentioned strategies, with high CASP3 proteolytic enzymatic activity, can be seen as a representation of what would be occurring in microglial cells that only exhibit modest DEVDase activity.

With a focus on CASP3, we developed an entirely novel approach to screen and identify new potential CASP3 substrates regulating specific microglia phenotypes and functions at the physiological level. To do so, we chemically inhibited the basal DEVDase activity in the BV2 microglia cell line by using DEVD-fmk inhibitor, which is a cell permeable irreversible CASP3 inhibitor. The proteome integral solubility alteration (PISA) assay was performed to uncover, in a proteome wide manner through mass spectrometry (MS)-based proteomics, the uncleaved proteins as proteins showing different soluble amounts between DEVD-fmk treated samples and DMSO controls, according to the PISA protocol perturbing the soluble state of the whole proteome by a temperature gradient (also named PISA-T) [30]. The application of the PISA assay in this strategical experimental setting revealed 99 potential microglial CASP3 substrates, including 12 previously established CASP3 substrates. A top candidate, Collectin-12 (COLEC12, also known as CL-P1), a scavenger receptor, is found to be increased in the soluble fraction after DEVD-fmk treatment. We then studied the effect of CASP3 inhibition on COLEC12 and observed that its expression increases at the microglia cell surface. Both, inhibition of CASP3-like enzymatic activity using DEVD-fmk and repression of *Colec12* gene expression by small interfering RNA (siRNA), increased the phagocytic ability of microglia for *Streptococcus pneumoniae* bacteria but only si*Colec12* was efficient to increase phagocytosis of amyloid beta (Aβ). Thus, we identified COLEC12 as a new microglial non-apoptotic CASP3 substrate, which can selectively regulate microglia functions. The continued search for new substrates for the modest and fine-tuned regulated CASP3 proteolytic activity in microglia should provide further insights into the signaling pathways regulating microglia activation states under both physiological and pathological conditions.

## Results

### PISA assay identifies COLEC12 solubility increase after CASP3 inhibition

Considering the multiple roles of caspases in regulating protein functions by cleavage in non-apoptotic conditions, we hypothesized that performing PISA assay and measuring soluble amounts of proteins after CASP3 inhibition would uncover new CASP3 substrates. We previously reported that baseline DEVDase activity is upregulated in microglia acquiring a pro-inflammatory activation state [20]. In contrast, when these immune cells acquire a tumor-supportive phenotype, baseline DEVDase activity is found to be repressed. *Casp3* silencing was shown to be able to mimic the effect of DEVD-fmk treatment in microglia, i.e., promotes a tumor supportive phenotype. Lastly, using a *Casp3*-deficient, but *Casp7* wild-type mouse model, we were able to confirm in vivo a specific role for CASP3 in the context of glioblastoma brain tumors. Having demonstrated that genetic or chemical inhibition of CASP3 in microglia, and associated decrease baseline DEVDase activity, contribute to the activation of these cells, we decided to investigate whether we can identify microglial potential CASP3 substrates in the context of inhibition of the baseline DEVDase activity upon DEVD-fmk treatment.

**Fig. 1:**
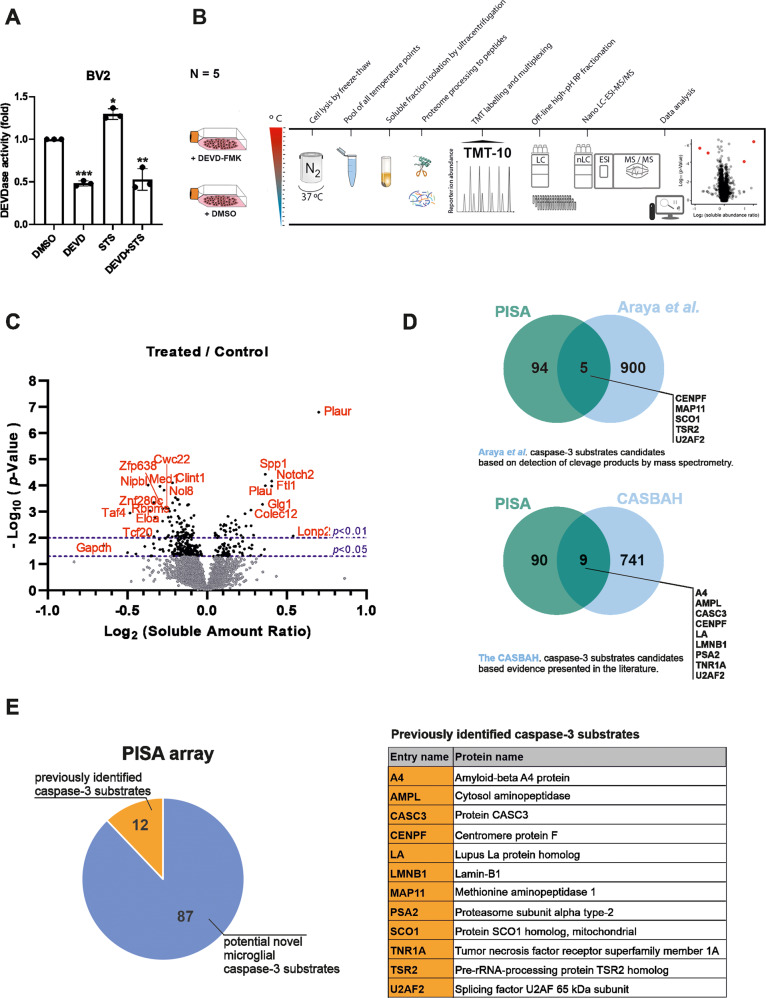
Identification of proteins with increase in solubility after DEVD-fmk treatment by PISA assay in microglia cells. **A** DEVDase activity measurement in BV2 microglia cells after treatment with DEVD-fmk, Staurosporine (STS), DEVD-fmk+STS compared to DMSO used as control. Statistics were performed with 2-way ANOVA from 3 independent experiments ±SEM, single data points are represented as dots. *P* value: *< 0.05, **<0.01, ***<0.001. **B** Workflow of the PISA assay in cell culture used in this study, using five flasks (biological replicates) treated with CASP3-like activity inhibitor DEVD-fmk (treated) and five biological replicates treated with equal volumes of DMSO (control). 2 h treated BV2 microglia cells with DEVD-fmk or DMSO (used as control) were lysed by freeze thaw and samples prepared for Proteome Integral Solubility Alteration (PISA) analysis. The data generated corresponds to a pool of 5 independent treated flasks. **C** Volcano plot of PISA results expressed as Log transformed values of the ratio of protein soluble amount per each protein of DEVD-fmk treated compared to DMSO control, and relative *p*-Value. Only proteins identified and quantified with ≥2 peptides are included in the final analysis and shown in the plot as dots. Protein reproducibly (*p*-Value < 0.05) altered in soluble amounts are shown as black dots and top-20 hits are labeled with gene name in red font. Gray dots represent identified proteins with no significant change (*p* > 0.05), black dots represent identified proteins with significant changes (*p* < 0.05). **D** Venn diagrams of comparative analysis of CASP3 substrates obtained from the PISA approach with available databases (top: Araya et al. database [27]; bottom: CASBAH database [31]). **E** Graphical representation of the distribution of the substrates identified by PISA compared to the 2 previously mentioned databases. Among the 99 significantly stabilized proteins after DEVD-fmk treatment, 12 were already known as CASP3 substrates and listed in the table below. 87 proteins are potential novel microglial CASP3 substrates.

We first established the effect of CASP3 inhibitor DEVD-fmk on the CASP3-like activity in microglia. Microglia cells treated with DEVD-fmk show a significant reduction in CASP3-like activity as measured by Caspase-Glo compared to control (DMSO) under basal conditions (Fig. 1A). These results confirmed the existence of a baseline CASP3-like activity even in control cells, where no cell death activity has been induced. We validated our model by inducing CASP3-like activity in microglia using a mild treatment with Staurosporin (STS) inducing a significant increase of DEVDase activity, which can also be inhibited by DEVD-fmk treatment (Fig. 1A).

After validating our setup and confirming the existence of a basal CASP3-like activity, non-related to apoptosis induction, we aimed at identifying proteins that would not be cleaved and therefore changed in soluble amounts in PISA after DEVD-fmk treatment and corresponding to potential CASP3 substrates. To do so, we applied PISA assay on cell cultures using 5 independent culture flasks treated either with DEVD-fmk or DMSO as control for 2 h before harvesting (Fig. 1B).

The PISA assay is a MS-based quantitative proteomics method that allows high throughput proteome profiling for drug target deconvolution and elucidation of mechanism of action. This enables identification of proteins that change reproducibly their soluble amount after thermal probing at different temperatures. PISA is technically more robust compared to previous methods due to the high multiplexing capacity and the hosting of statistically relevant number of biological replicates. The differential soluble amount after normalization (ΔSm), that can also be expressed as a ratio of soluble protein amounts of treated/control samples, describes a statistically relevant change, due to DEVD-fmk treatment, of the proteins in their relationship with the solution and with the events like denaturation or aggregation triggered by the PISA temperature range. Independently from any thermodynamic interpretation of single proteins in the complex molecular matrix of a cell, the proteins affected by the treatment are different in their solubility points at lower or higher temperature in PISA, this will be reflected in a total protein abundance in a robustly quantifiable manner across several biological replicates. For such properties, in this study, PISA is used as an innovative approach to reveal CASP3 enzyme substrates, by their change in soluble amount fraction compared to controls. The top candidates in the PISA results are ranked based on both the ratio of soluble fraction amounts and the p-value of such event, with a score based on the absolute values of their logarithmic transformed value.

In total, 5 399 proteins were identified with high confidence, and 4 599 quantified with at least 2 unique peptides across all biological replicates of treated and control samples. From the analysis, 99 proteins were found having significant changes after DEVD-fmk treatment compared to DMSO. No big shifts were observed among all proteins, however, small variations in thermal shift due to the DEVD-fmk treatment were found to be consistent between the five replicates. In our setup, the modulation of CASP3-like activity is very mild, as we reduce the already low basal level of activity. Hence, the degree of changes between protein thermal shifts is expected to be low (Fig. 1C and Supplementary Table 4).

We then made use of already available datasets from two previous studies to compare our results to already identified CASP3 substrates. Among the identified proteins significantly stabilized after DEVD-fmk in our PISA dataset, we found that 9 of them have been identified as CASP3 substrates by the CASBAH dataset and 5 are found in common to the dataset generated by Araya and collaborators (Fig. 1D, E) [27, 31]. Even though these two databases have identified CASP3 substrates in the context of cell death pathway activation, the comparison with our PISA dataset reveals common targets validating our approach as a new unconventional technique to identify CASP3 substrates (Fig. 1D). One cannot exclude those effects in term of protein solubility observed in the PISA assay, upon treatment of microglial cells with DEVD-fmk, are not the result of indirect effects of DEVD-fmk on those proteins, instead of an absence of CASP3-mediated protein cleavage. However, it should be mentioned that at the dose used, DEVD-fmk is reported to act as a CASP3 proteolytic activity inhibitor by binding irreversibly to its enzymatic catalytic site. In addition, to get further support that the proteins identified in the PISA assay are potential CASP3 substrates, we examined those proteins for the existence of possible CASP3 cleavage sites in their amino acid sequences (Supplementary Table 5). Finally, using this innovative approach we have identified 87 potential novel microglial non-apoptotic CASP3 substrate candidates, including COLEC12 (Fig. 1E and Supplementary Table 5). Gene Ontology enrichment, as well as KEGG pathway analyses performed on those protein candidates revealed that beyond apoptosis, a possible involvement of processes linked to AD, amyotrophic lateral sclerosis (ALS), neurodegeneration, and immune response, all processes of particular interest for microglia (Supplementary Fig. 1).

### COLEC12 expression increases after CASP3 inhibition in microglia

Among the different proteins identified by PISA, we choose to focus on COLEC12 (Collectin subfamily member 12, also known as CL-P1), a transmembrane receptor, which belongs to the group of proteins which have the most significant increase in solubility after DEVD-fmk treatment in microglia (Fig. 1C).

**Fig. 2:**
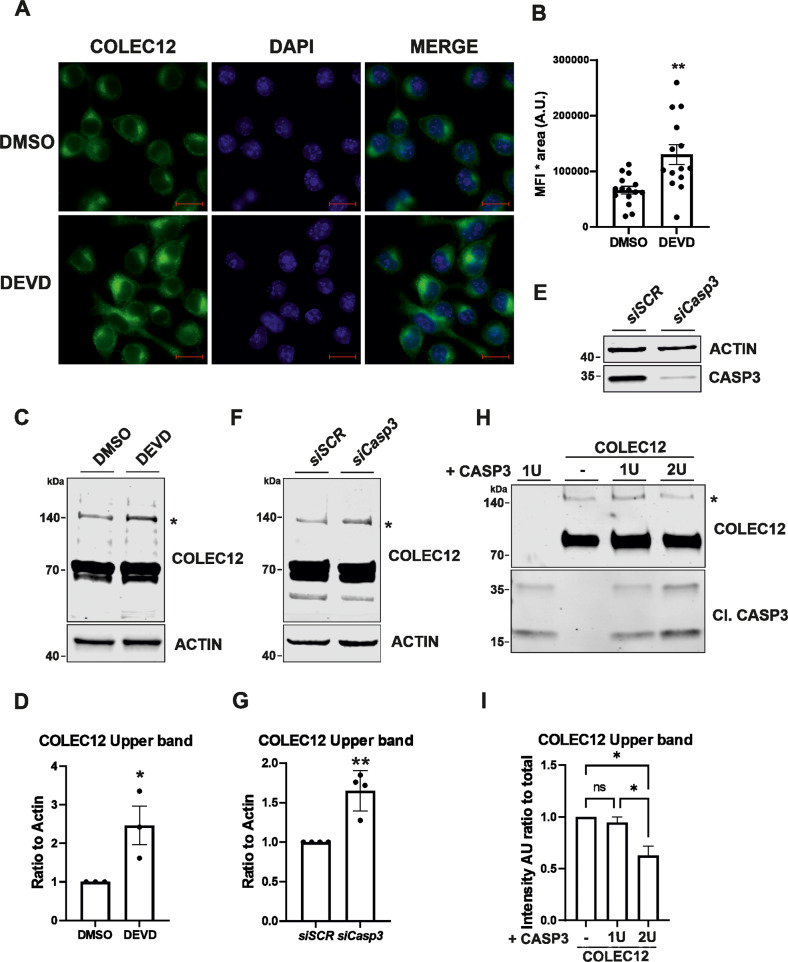
Microglial COLEC12 protein expression increases after DEVD-fmk treatment or CASP3 inhibition. **A** Immunofluorescence analysis of COLEC12 protein expression in BV2 microglia cells treated with DEVD-fmk or DMSO used as control and **B** quantification of the Mean fluorescence intensity of the COLEC12 staining depicted in panel (**A**). **C** Western blot analysis of COLEC12 expression in BV2 cells treated with DEVD-fmk or DMSO used as control. COLEC12 antibody reveals 2 bands at different molecular weight, upper band at 140 kDa is highlighted with a star. **D** Quantification of the expression of the COLEC12 140 kDa band (marked with a star in **C**) revealed by western blot, ratio to Actin expression. **E** Western blot analysis of CASP3 expression in BV2 cells transfected with *siCasp3* or *siSCR* used as control. **F** Western blot analysis of COLEC12 expression in BV2 cells transfected with *siCasp3* or *siSCR* used as control and quantification of the 140 kDa COLEC12 band from the corresponding western blots (**G**). **H** Western blot of CASP3-dependent COLEC12 proteolysis assay using recombinant proteins and different amounts of CASP3 (1 or 2U for Units of protein) revealed using COLEC12 and Cleaved Caspase 3 antibodies. **I** Quantification of the COLEC12 140 kDa band from the CASP3-dependent COLEC12 proteolysis assay depicted in panel **H**. Statistics were performed with Welch´s t test from 14 individual cells from 2 independent experiment (**B**), unpaired t-test (**D**, **G**) and one-way Anova (**I**) from 3 (**D**, **I**) or 4 (**G**) independent experiments ±SEM, single data points are represented as dots *P* value: *<0.05, **<0.01 Scale bars for panel **A**: 20 µm.

To understand the role CASP3 might have on the regulation/cleavage of COLEC12 in basal conditions, we investigated the expression of the COLEC12 protein in microglia after DMSO (used as control) or DEVD-fmk treatment. Immunofluorescence analysis of COLEC12 reveals that its expression is increased after DEVD-fmk treatment compared to control (Fig. 2A, B). We investigated if the observed increase of COLEC12 expression was due to the main protein increase or due to less cleavage by CASP3. Western blot analysis shows the presence of two main bands at 70 and 140 kDa when revealed by an antibody targeting COLEC12 (Fig. 2C). We observed that the 140 kDa band is found to be significantly increased in DEVD-fmk treated condition (Fig. 2C, D). To confirm this observation, we inhibited specifically CASP3 expression using an siRNA targeting *Casp3*. *Casp3* knock down was validated by western blot where we could observe a strong reduction of CASP3 expression in *siCasp3* transfected cells compared to *siSCR* used as control (Fig. 2E). Similar to CASP3-like inhibition by DEVD-fmk, *siCasp3* induces a significant increase in the expression of the COLEC12 140 kDa band as observed by western blot (Fig. 2F, G). Lastly, we used in vitro caspase-dependent substrate proteolysis assay to gain further evidence of the CASP3-dependent cleavage of COLEC12 using recombinant proteins. Western blot analysis of recombinant COLEC12 protein in Caspase substrate proteolysis assay shows a significant decrease of the 140 kDa COLEC12 band with 2 units of CASP3 recombinant protein compared to control, validating the hypothesis of COLEC12 being less cleaved after DEVD-fmk treatment (due to CASP3 inhibition) and hence being a substrate of CASP3 (Fig. 2H, I).

### COLEC12 interacts with CASP3 in microglia

**Fig. 3:**
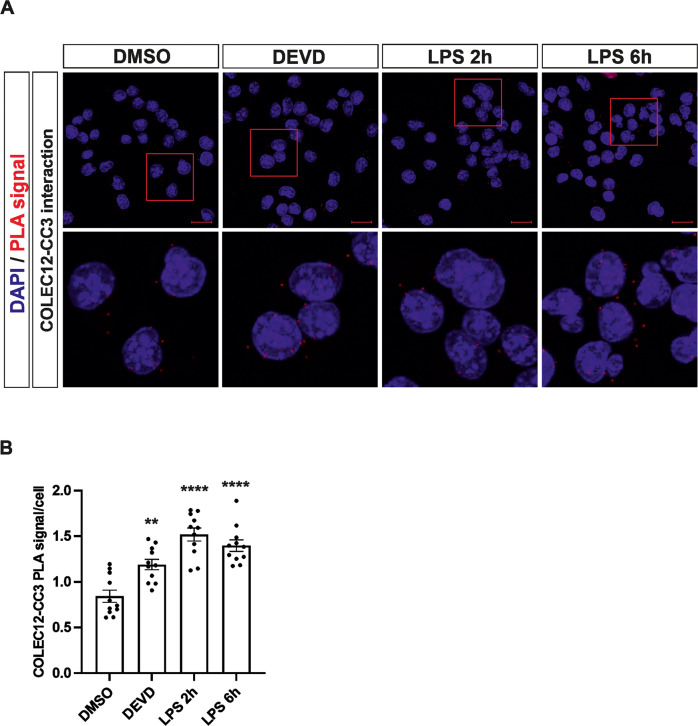
COLEC12 interacts with cleaved CASP3. **A** Confocal analysis of Proximity Ligation assay reveals protein interaction between COLEC12 and cleaved CASP3 (CC3) in BV2 cells treated with DEVD-fmk or LPS for 2 h or 6 h or DMSO for 2 h as control. DAPI was used for nuclear counterstaining. The bottom picture represents a crop of the above corresponding picture indicated by a red square. **B** Quantification of the PLA experiments displayed in panel (**A**). Statistics were performed with a 2-way ANOVA from 3 independent experiments ±SEM with dots counted in 3 to 5 pictures per experiment and analysis compared to DMSO control. *P* value: **<0.01, ****<0.0001. Scale bars for panel **A**: 20 µm.

The increased protein soluble amount measured by PISA of COLEC12 after CASP3-like activity inhibition highlights the possibility that CASP3 interacts and cleaves COLEC12 under physiological conditions. CASP3 expression and activation in microglia is very low at the basal level. In order to study the interactions between COLEC12 and cleaved CASP3 as activated form of CASP3, we took advantage of the high sensitivity of the Proximity Ligation Assay (PLA) approach. PLA reveals that activated cleaved CASP3 and COLEC12 interact in basal (DMSO-treated) and DEVD treated microglia cells (Fig. 3A). Of interest, we observed a significant increase of COLEC12-CASP3 interaction after DEVD-fmk treatment. This might be due to the accumulation of interaction between COLEC12 and CASP3, as CASP3-like activity is inhibited by DEVD-fmk, preventing the cleavage and therefore release of the soluble form of COLEC12. To confirm this interaction in activated microglia independently of cell death activation, we treated microglia cells with lipopolysaccharides (LPS) for 2 and 6 h. LPS is known to activate microglia into pro-inflammatory cells but also increases CASP3 activity without triggering cell death in microglia [20]. LPS treatment significantly increases the interaction between CASP3 and COLEC12 (Fig. 3A, B) and validates a role for CASP3 in COLEC12 protein regulation.

### COLEC12 protein structure reveals a conserved CASP3 cleavage site

**Fig. 4:**
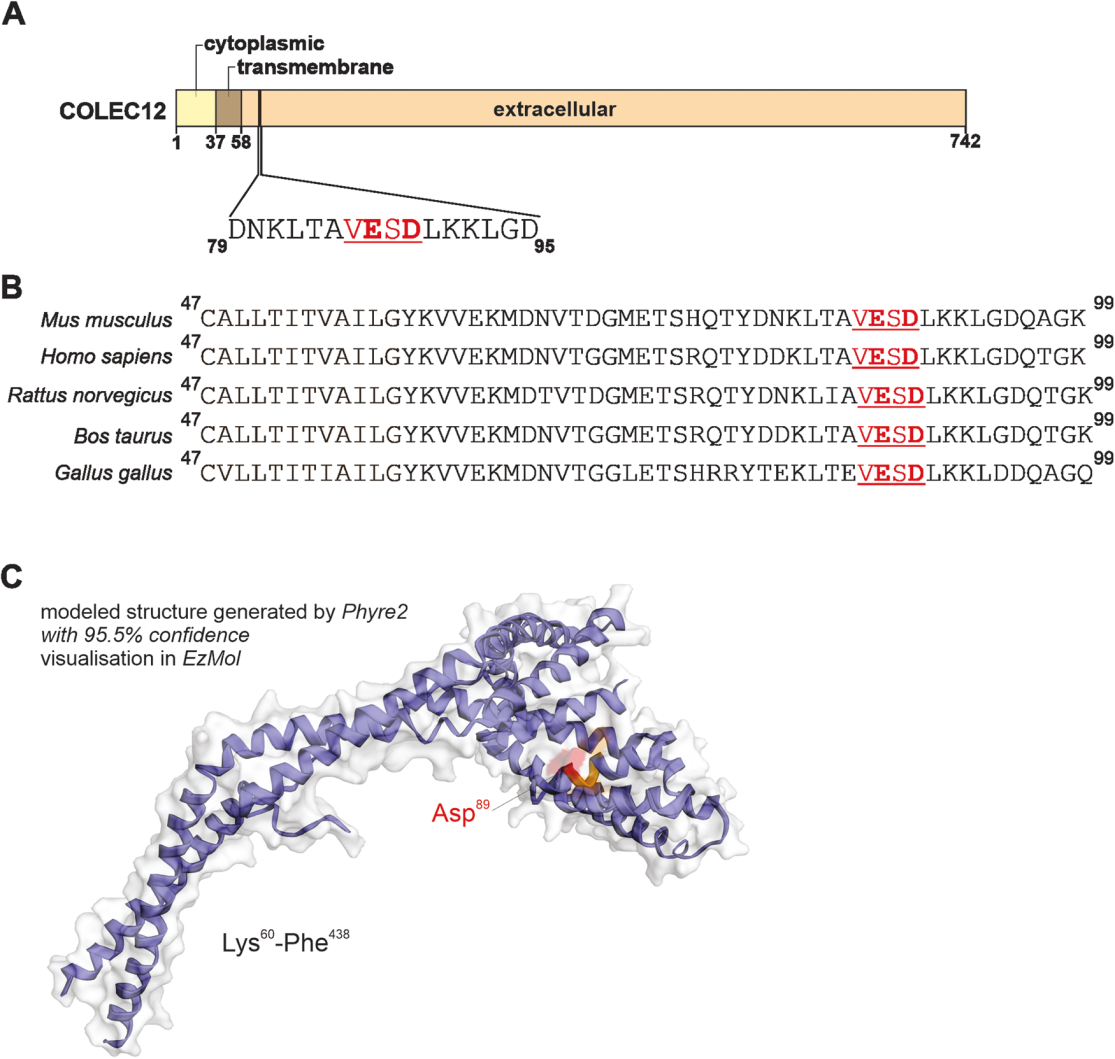
Potential CASP3 cleavage site in COLEC12 protein. **A** Schematic diagram of the functional domains and the potential CASP3 cleavage site in COLEC12. The full-length COLEC12 protein contains an intracellular (yellow), a transmembrane (brown) and an extracellular (orange) domains. **B** Using the prediction tools SitePrediction and Procleave, a conserved CASP3 substrate cleavage site (VESD.LK) at amino acid 86 with >99% specificity was identified in COLEC12 protein sequence from several species. **C** 3D modeling of 378 residues of the COLEC12 protein. The VESD cleavage site is highlighted in orange with the Asp (D) residue in red.

As COLEC12 can exist in a full or a soluble form, we hypothesized that CASP3 could cleave COLEC12 at basal level to regulate microglia function [32, 33]. Microglial cells are notoriously difficult to transfect, particularly with gene expression vectors, as the insertion of foreign DNA will lead to cell activation and very often to cell death. Therefore, a mutagenesis approach which would have required the transfection of the BV2 microglia with plasmid encoding for wild-type COLEC12 *versus* a mutated version of it was not considered as technically viable option. Instead, we took advantage of the prediction resources SitePrediction [34] and Procleave [35] to identify CASP3 substrates and discovered a potential CASP3 cleavage site (VESD.LK) located in the extracellular domain of COLEC12, 20 amino acids downstream of the protein transmembrane domain (Fig. 4A) which is further highly conserved between several species (Fig. 4B). Cleavage at this site could contribute to the formation of soluble form of COLEC12 reported in the literature [33]. Cleavage of COLEC12 by CASP3 would require that the identified potential cleavage site would be accessible by CASP3 to bind and cleave. To further validate the potential identified cleavage site, we investigated the accessibility of the VESD sequence identified on COLEC12 protein. Using the Phyre2 web portal for protein modeling, prediction, and analysis we were able to model 50% of the full protein sequence with a high confidence rate (more than 95%). 3D modeling of 378 residues of the COLEC12 protein showed a clear accessibility of the potential CASP3 cleavage site on COLEC12 (Fig. 4C).

### COLEC12 cleavage by CASP3 regulates microglia function by increasing their ability to phagocytose bacteria but not Aβ

**Fig. 5:**
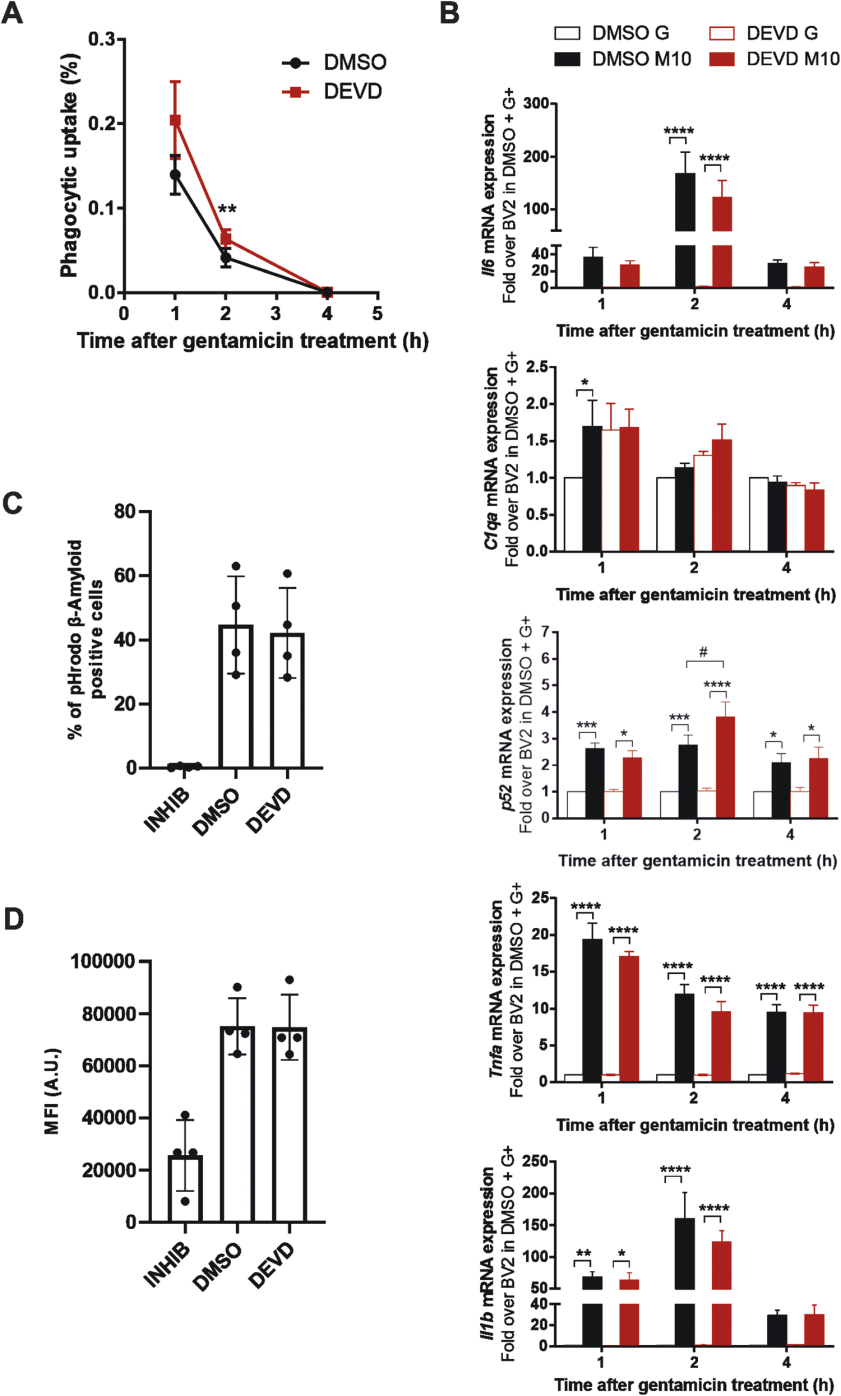
Inhibition of COLEC12 cleavage by CASP3 increases microglial phagocytic ability for bacteria but not Aβ. **A** Phagocytic uptake of BV2 cells treated with DEVD-fmk or DMSO used as control at different time points after gentamicin addition. **B** qPCR analysis of *Il1b*, *Il6*, *Tnfa*, *Nfkb2-p52* and *C1qa* mRNA expression in BV2 cells treated with DEVD-fmk or DMSO used as control following bacterial incubation and gentamicin treatment (M10) or gentamicin alone (G). **C**, **D** Aβ phagocytosis capacity of BV2 cells after treatment with DEVD-fmk or DMSO or with inhibition of phagocytosis (INHIB) used as controls represented by the percentage of microglia cells positive for pHrodo stained Aβ (**C**) or Mean fluorescence intensity (MFI) (**D**). Statistics were performed with a 2-way ANOVA from 4 (**C**, **D**) to 5 (**A**, **B**) independent experiments ±SEM compared to DMSO control; single data points are represented as dots. *P* value: *or #<0.05; **<0.01; ***<0.001; ****<0.0001.

COLEC12 has been shown to regulate several different functions in the cell. In AD, microglia cells have been shown to be neuroprotective by delaying the progression of AD through the clearance of Aβ, yet these immune cells can also be part of the disease progression through cellular overactivation [36]. COLEC12 has further demonstrated to contribute to the phagocytosis of bacteria or yeast through binding to bacteria resulting in opsonophagocytosis [32, 33]. Thus, we investigated the impact of CASP3 inhibition on the ability of microglia to phagocytose bacteria. For this purpose, we performed phagocytosis assays using *S. pneumoniae* serotype 4 strain TIGR4, which is a gram-positive bacterium and known to cause bacterial meningitis [37]. Figure 5A displays the percentage of intracellular *S. pneumoniae* after 90 min of infection and 1 h of gentamicin treatment. Here, DEVD-fmk treated BV2 exhibit a higher presence of intracellular bacteria, which is still evident at 2 h while at 4 h BV2 cells of both conditions were able to clear all intracellular bacteria. Shen et al. demonstrated that by the inhibition of the CASP3 activity microglia become less pro-inflammatory or in the case of glioma turn tumor-supportive [23]. Interestingly, when analyzing the gene expression of DEVD-fmk treated BV2 cells after *S. pneumoniae* infection their gene expression follows the same pattern of upregulation of genes associated with inflammation (Fig. 5B). While *Il1β, Il6* and *Tnf*α show a significantly altered gene expression after infection for both, DMSO and DEVD-fmk treated BV2. The treatment with DEVD-fmk alone resulted in no significant changes in expression levels of the assessed genes. Only *p52 (*i.e., *Nfkb2)* displays an increased expression in DEVD-fmk treated and *S. pneumoniae* infected BV2 at 2 h after gentamicin treatment. *C1qa*, a subunit of complement C1q, indicates that the bacterial infection leads to an increase in its gene expression. DEVD-fmk treatment alone can raise the gene expression too, however not significantly and in combination with bacterial incubation the heightened expression of *C1qa* does not increase further.

Scavenger receptors are established receptors for insoluble fibrillar Aβ aggregates, and are expressed by activated microglia, mediating the endocytosis of oligomeric and fibrillar Aβ. As COLEC12 has been described to potentially participate in Aβ phagocytosis through its scavenger receptor role to bind Aβ and be present in microglia and astrocytes close to Aβ plaques [38, 39], we hypothesized that the inhibition of CASP3-like activity resulting in an increased COLEC12 expression could affect the microglia ability to regulate Aβ endocytosis. We investigated the effect of CASP3 inhibition by DEVD-fmk on microglial Aβ phagocytosis and observed no change in the percentage of microglial cells positive for pHrodo stained Aβ, nor the mean of fluorescent intensity (MFI) (Fig. 5C, D). The difference observed between the microglial phagocytic ability towards *S. pneumoniae* and Aβ suggests that CASP3 inhibition of COLEC12 cleavage affects different phagocytosis pathways in microglia.

### Silencing of *Colec12* by siRNA increases microglial phagocytic capacity towards bacteria and Aβ

**Fig. 6:**
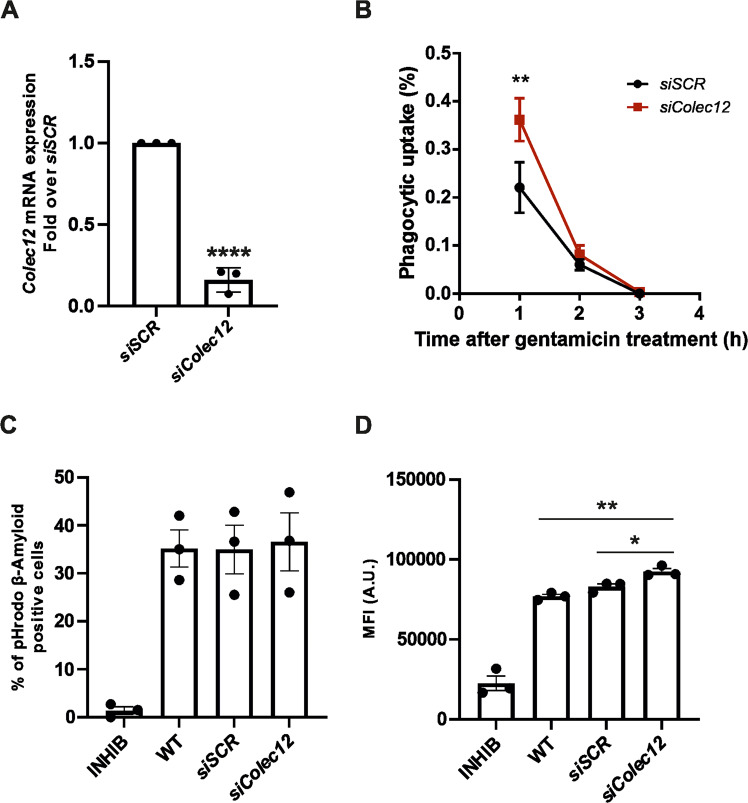
Inhibition of *Colec12* expression by siRNA increases microglial phagocytic capacity for bacteria and Aβ. **A** qPCR analysis of *Colec12* expression in microglia transfected with *siColec12* or Scramble (*siSCR*) used as control. **B** Phagocytic uptake of BV2 cells treated with *siColec12* or *siSCR* used as control at different time points after gentamicin addition. Aβ phagocytosis capacity of BV2 cells transfected with si*Colec12* or Scramble (*siSCR*) used as control represented by the percentage of microglia cells positive for pHrodo stained Aβ (**C**) or Mean fluorescence intensity (MFI) (**D**). Statistics were performed with unpaired t-test (**A** and **D**) or a 2-way ANOVA (**B**) from 3 (**A**, **C**, and **D**) to 5 (**B**) independent experiments ±SEM; single data points are represented as dots. *P* value: *<0.05; **<0.01; ****<0.0001.

The discovery that the inhibition of CASP3-like activity and thereby COLEC12 cleavage resulted in an increase of bacterial phagocytosis by microglia cells lead us to hypothesize that the soluble form of COLEC12 generated by CASP3 cleavage could be of importance in microglia phagocytic function. To investigate this role, we used an alternative approach to reduce the soluble form of COLEC12. The knockdown of *Colec12* expression by siRNA was used to reduce the release of its soluble form. After confirmation of the efficiency of si*Colec12* observed by the significant reduction of *Colec12* mRNA expression (Fig. 6A) we used the same approaches previously mentioned to investigate the effect of si*Colec12* on microglial phagocytic ability. Surprisingly, si*Colec*12 BV2 cells showed a significantly greater presence of intracellular bacteria after 1 h with gentamicin treatment implying greater phagocytotic uptake and with similar levels of intracellular bacteria after 2 h indicating that si*Colec12* BV2 can kill phagocyted bacteria faster compared with their respective control (Fig. 6B). Moreover, silencing of *Colec12* in BV2 did not show changes in the percentage of Aβ positive cells; however, MFI was significantly upregulated compared to *siSCR* (Fig. 6C, D). Increased MFI as well as bacterial phagocytosis in BV2 cells silenced for *Colec12* are in accordance with the increased bacterial phagocytosis after DEVD-fmk treatment and support a role for COLEC12 protein cleavage by CASP3 in the regulation of microglial phagocytic ability.

Overall, our results present a new innovative approach to identify new CASP3 substrates. From the identification of 99 CASP3 substrates, including 87 new ones, we focused our investigation on COLEC12 which belongs to the group of proteins which have the most significant increase in solubility after CASP3-like activity inhibition. COLEC12 cleavage by CASP3-like activity seem to play a role in regulating microglia phagocytic capability. Inhibition of the production of soluble COLEC12 by inhibiting CASP3-like activity or si*Colec12* impacts on the phagocytic capacity of microglia cells by increasing their bacterial and Aβ uptake.

## Discussion

CASP3 is commonly known for its role in cell death induction by apoptosis through the activation of the caspase cascade pathway. As a cysteine protease, CASP3 gets activated by cleavage of its pro-form, which in turn will cleave targeted substrates. At baseline levels, caspases are also able to cleave protein targets as part of the physiological protein regulation in the cell [40]. Non-apoptotic roles of CASP3 have been recently investigated as major player of cell physiology but also in the case of diseases [17, 18, 23].

So far, the identification of novel caspase substrates has been done in the context of cell death where caspase activity is strongly induced, leading to very high caspase levels in the cell [24, 25]. Significant progress has been made using high-throughput proteomics technologies to identify protein substrates for proteases, including for CASP3, based on the detection of proteolytic peptide fragments. However, it is important to note that these methods are still all based either on intact cells in which the enzymatic activity of endogenous caspase(s) is triggered by mostly apoptotic stimuli or in protein cell lysates with the addition of a selective exogenous recombinant caspase (reviewed in [29]). In this apoptotic context, the identification of physiological CASP3 substrates is hidden by the high caspase activity. None of these approaches are relevant in the context of surveying or activated microglia, where only modest CASP3-dependent DEVDase enzymatic activity are detected, far beyond the level observed in dying microglia [20, 23, 41]. The discovery of these new CASP3 substrates remains a challenge due to the mild changes in CASP3-like activity at baseline level, which ultimately complicates their detection and investigation. Basal microglial DEVDase activity was inhibited in BV2 cells taking advantage of the cell permeable irreversible CASP3 inhibitor, i.e., z-DEVD-fmk. Thereafter, the PISA assay was carried out to identify potential CASP3 substrate candidates altered in their solubility as probable measure of differentially cleaved substrates upon inhibition of CASP3 in microglia [30]. We took advantage of this very robust and sensitive method to uncover proteins that could be affected/cleaved by the CASP3 protein at baseline levels. Indeed, the PISA assay is a MS-based quantitative proteomics method that allows high throughput proteome profiling to be carried out for target deconvolution and mechanism of action, allowing identification of proteins that change reproducibly their soluble amount after thermal probing at different temperatures. The use of this method spares the use of cell death agents (commonly used for the discovery of CASP3 substrates) and allows us to follow CASP3-like activity at its fine-tuning baseline level.

The method has proven to be reliable as 12 previously established CASP3 substrates according to available databases [27, 31] were also found as results of the PISA assay performed on DEVD-fmk-treated BV2 cells compared to their counterpart treated with DMSO. This proteomic technology verified itself to be an adequate method for the discovery of potential microglial CASP3 protein substrates, with 87 newly identified candidates.

Of note, the majority of the identified candidate substrates in our study has not been previously reported as caspase targets (only 12 of the 99 proteins having a significant change in their solubility were already reported caspase substrates). An obvious explanation would be that our investigations are aiming at the identification of potential CASP3 substrates in the context of low DEVDase activity, which differ from the existing studies that looked at Caspase-3 substrates in the context of apoptotic cells characterized by high DEVDase activity. In this situation, it is easy to speculate that substrates not related to apoptosis induction (like in our model) would not be detected or would be hidden behind abundant apoptotic-related substrates. Moreover, the strategy we used to uncover CASP3 substrate candidates is entirely different from previous reports. Whereas earlier reports aimed at the detection of proteolytic products in the presence of CASP3-like enzymatic activity, we use the PISA assay to detect proteins that have a change in solubility in microglia when basal CASP3-like enzymatic activity was inhibited using DEVD-fmk. Hence, induction of high DEVDase activity in cells, versus inhibition of low baseline DEVDase activity in cells, as well as method aiming at the detection of cleavage products versus method aiming at the detection of stabilization of proteins, can both have contributed to the discovery of yet unreported CASP3 substrate candidates.

In the PISA assay, COLEC12, a transmembrane scavenger receptor, is found to be of higher amount or most stabilized in the soluble fraction after inhibition of CASP3-like activity and was therefore selected for further validation. COLEC12 has been reported to exist in myeloid cells, including microglia in a full but also soluble form which can have separate functions, including a role as pattern-recognition molecule or a protective role against pathogens [32, 33, 42]. Inhibition of CASP3-like basal activity in microglia lead to the increase of the 140 kDa COLEC12 protein form. The reduction of the cleaved form of COLEC12 in the DEVD-fmk condition as well as the reduction of the full protein expression by siRNA increased the ability of microglia to phagocyte *S. pneumoniae* bacteria, which reveals a new role for COLEC12 in regulating microglia function. Collectively, these data suggest that cleaved COLEC12 could mediate an inhibitory effect on the bacterial uptake, possibly acting as a decoy receptor binding a ligand on *S. pneumoniae* and keeping it from binding to its regular receptor. A decoy receptor can recognize and bind specific growth factors or cytokines efficiently but is not structurally able to signal or activate the intended receptor complex. It acts as an inhibitor, binding a ligand and keeping it from binding to its regular receptor. For example, the cleaved N-terminal E-cadherin peptides have been shown to act as decoy protein to protect epithelial cells from *Listeria monocytogenes* infection [43]. The protective effect of decoy protein has also been demonstrated in the context of excessive T-cell activation where bacterial clearance can be impaired. In this context, the use of a decoy receptor analog can reduce T-cell activation and enhance proper bacterial clearance [44]. In the case of COLEC12, our data highlight the role of soluble COLEC12 in binding surrounding bacteria and blocking their interaction with microglia, which is prevented by CASP3 inhibition or *Colec12* silencing. These results highlight a new role for CASP3 in regulating cleavage of COLEC12 to release its soluble form which, in the case of microglia and based on our results, seems to act as a decoy, protecting microglia against bacterial infection as previously described in endothelial cells [32]. Worth an additional note, the uptake of Aβ and bacteria by microglia are reported to be mediated by distinct set of receptors, which could explain the differences of microglial phagocytic capacity observed after CASP3 inhibition [45, 46].

Here, we chose to focus only on COLEC12 and its regulation by CASP3 but the PISA assay has also uncovered a variety of other proteins significantly increased in soluble amount as potential new CASP3 targets, which may be of interest for microglia function. Interestingly, these protein candidates include several molecular players established to regulate various biological functions in microglia, such as CSF1R [47], Galectin-9 [48], Osteopontin/SPP1 [49] and the urokinase plasminogen activator (uPA) and its receptor [50, 51]. The role of CASP3 in regulating these and other identified proteins as potential new substrates of microglial basal CASP3 remains to be investigated.

Finally, more detailed investigations on cleavage sites of COLEC12 by CASP3 as well as detection of the soluble cleaved form of COLEC12 and its mode of action in microglia physiology would be of interest. As microglia have been shown to participate in the progression of certain pathologies such as AD or GB [52, 53], it would be of interest to study the role of microglial cleaved COLEC12 in a pathological context. Thus, collectively our data indicates that adopting PISA for the identification of proteins altered in solubility upon the inhibition of basal CASP3 proteolytic activity in microglia offer a suitable method for the identification of novel non-apoptotic CASP3 substrates.

## Materials and methods

### Cell culture and treatment

BV2 mouse microglia cells were cultivated in DMEM + glutamax medium (Gibco, Fisher Scientific, Waltham, MA, USA) supplemented with 10% FBS (fetal bovine serum) and 1% P/S (Penicillin/Streptomycin) and grown in an incubator at 37 °C and 5% CO_2_. For treatment conditions, microglia cells were seeded in 6 or 12 well plates the day before the treatment at 200,000 or 100,000 cells per well. 24 h after seeding, cells were treated with either 20 μM Caspase-3 inhibitor (DEVD-fmk, R&D, Minneapolis, MN, USA), 100 ng/mL Lipopolysaccharides (LPS, Sigma Aldrich, Saint Louis, MO, USA), 0.5 μM Staurosporine (STS, Sigma Aldrich) or DMSO used as control for 2 h before harvest.

### PISA assay

The Proteome Integral Solubility Alteration (PISA) assay was carried out using the PISA assay and Tandem Mass Tag (TMT) 10-plex reporter ion quantitative proteomics technology, similarly to the originally published PISA assay method [30]. Briefly, BV2 cells were cultured as above-described into ten T25 flasks, five of which were treated for two hours in cell culture with 20 μM DEVD-fmk and five with DMSO used as control. After treatment, cells were detached using TrypLE solution (Fisher Scientific), washed with 10 ml phosphate buffered saline (PBS) twice and cell pellets were collected and resuspended in 1 ml of PBS supplemented with protease inhibitors (Roche, Basel, Switzerland).

#### PISA thermal treatment and soluble fraction isolation

Each cell suspension of each biological replicate was split into fifteen 0.2 ml PCR tubes, distributing homogenous cell suspension of 60μl, one for each temperature point, with the first being 43 °C and the last 57 °C, with one °C of interval. The thermal treatment at each temperature point was performed for all 10 biological replicates for 3 min at each temperature using a SimplyAmp thermal cycler (Applied Biosystems, Fisher Scientific). Samples were then left at room temperature for 6 min before being snap frozen in liquid N2. Cell lysis was then obtained by freezing cells in liquid N2 and then thawing at 35 °C. After four freeze-thaw cycles all temperature points aliquots of the same biological replicates were re-united, then ultracentrifuge sedimentation was performed at 150,000 × *g* for 30 min at 4 °C using an Optima XPN-80 (Beckman Coulter, Brea, CA, USA) ultracentrifuge.

#### PISA-Proteomics sample processing

Total protein content of the sample was determined by bicinchoninic acid -based assay. Fifty μg of each sample was processed as follows: reduction with 8 mM dithiothreitol (55 °C, 45 min), alkylation with 25 mM iodoacetamide (30 min, 25 °C, in darkness), protein precipitation overnight at −20 °C using sample:cold acetone in a ratio of 1:5. After precipitation, the supernatant was discarded and protein pellets were resuspended in 20 mM EPPS containing 8 M urea, then samples were progressively diluted in 20 mM EPPS to lower urea concentration and digested using LysC enzyme at 30 °C for 6 h at the substrate:enzyme ratio of 1:75 followed by trypsin digestion overnight at 37 °C (enzyme:substrate ratio of 1:50). The digested proteins were labeled using TMT 10‐plex for two hours and pooled in the final 10-plex sample according to manufacturer’s protocol. The 10-plex sample was cleaned and desalted using on C18 SepPack column C18 (Sep-Pak, C18, Vac 1 cm^3^, 50 mg, Waters, USA), then all the eluate was speedvac dried.

#### 1D – Off-line peptide separation by reversed-phase high pH fractionation

The first dimension of peptide separation was carried out according to previously published protocol [30] using a C-18 at a capillary flow rate of 200 μL/min. Fractionation was applied using a binary solvent system consisting of 20 mM NH_4_OH in H_2_O and 20 mM NH_4_OH in acetonitrile. The elution was monitored measuring UV absorbance at 214 nm. A total of 96 fractions of 100 μL each were collected and concatenated into 24 fractions. Each fraction was dried by speedvac concentrator overnight.

#### 2D-nLC coupled t ESI-MS/MS analysis

1 μg of each fraction was injected for LC-ESI-MS/MS analysis using a Q Exactive HF mass spectrometer (Thermo Scientific, Waltham, MA, USA) equipped with an EASY Spray Source and connected to an UltiMate 3000 RSLC nanoUPLC system (Thermo Scientific), as previously described [30]. Peptide separation was performed using an EASY-Spray C18 reversed-phase nano LC column (length, 50 cm; inner diameter, 75 μm; particle size, 3 μm; pore size, 100 Å; Thermo Scientific) at 55 °C and a flow rate of 300 nL/min. Peptides were separated using a binary solvent system consisting of 0.1% (v/v) formic acid (FA), 2% (v/v) acetonitrile (ACN) (solvent A) and 98% ACN (v/v), 0.1% (v/v) FA (solvent B) and eluted with a gradient of 4–26% B in 91 min, 26–95% B in 9 min. Subsequently, the analytical column was washed with 95% B for 5 min before re-equilibration with 4% B. Data dependent acquisition using a top 17 method was used, with an isolation window of 1.6 m/z and dynamic exclusion time of 45 s.

#### Protein identification, quantitative, and statistical data analysis

Protein identification and quantification was performed using Proteome Discoverer v2.4 using Mascot and Sequest in series as search engines. The complete Uniprot mouse proteome reference database (UP000000589) was applied for matching MS/MS spectra. TMT10 quantification of peptide and protein abundances was used. Cysteine carbamidomethylation was used as a fixed modification; methionine oxidation, arginine, and glutamine deamination were used as variable modifications for both identification and quantification. Trypsin cleavage with maximum two missed cleavages were allowed, and only high confidence (1% false discovery rate) was retained in the dataset at both protein and peptide levels. After removing contaminants, only proteins with at least two unique peptides were included in the final data set. Quantification values for each protein were normalized on the total ion abundance of TMT10 reporters for a given protein and then on the average value for the untreated sample. Soluble abundance ratio and ΔSm value were obtained on normalized values, two-tailed Student’s t test was applied to calculate *p*-values, and logarithmic transformation was applied prior to graphical visualization (raw data can be found in Supplementary Table 4 and raw proteomic data can has been uploaded to the PRIDE repository with accession number: PXD032835).

### Enrichment analysis

Respective genes of selected 99 proteins were analyzed for Gene Ontology (GO) enrichment by bioinformatic online database tool Metascape.org [54]. GO enrichment maps were created using Cytoscape (v3.7.1, www.cytoscape.org) [55] with the Enrichment Map plugin (www.baderlab.org/Software/EnrichmentMap) [56]. The individual nodes represent enriched GO terms. Connecting lines, or edges, represent the degree of overlap between nodes. KEGG pathway analysis was performed using DAVID bioinformatic database [57, 58].

### Comparative analysis of CASP3 substrate data sets

Protein exhibiting an increased ΔSm in the PISA assay (with a *P* value <0.05%), i.e., thermal proteome profiling, upon CASP3 inhibition using DEVD-fmk in BV2 cells were defined as potential microglial CASP3 substrates. The 99 identified microglial CASP3 substrate protein candidates were compared to two caspase substrates databases. The first dataset was recently published by Araya et al. [27] and employed deep profiling by subtiligase N-terminomics, to identify recombinant CASP3-induced proteolytic peptides on Jurkat cell lysates and thereby CASP3 substrates. The second dataset used, The CASBAH, is a searchable caspase substrates database (http://bioinf.gen.tcd.ie/cgi-bin/casbah/casbah.pl) compiled from literature search for caspase substrates [31]. Only mammalian proteins were included from The CASBAH database. VENN diagrams were generated using the online tool jvenn (http://jvenn.toulouse.inra.fr/app/example.html; [59]) to illustrate overlap in the lists of CASP3 substrate candidates between these two datasets (905 and 752, respectively) and the microglial CASP3 substrate candidates identified in the PISA assay.

### CASP3-like activity assay

Analysis of CASP3-like activity was carried out using the Caspase-Glo 3/7 assay (Promega, Madison, WY, USA) following the manufacturer´s instructions. Briefly, 75,000 BV2 cells per well were seeded the day before in 24 well plates. The day after, cells were treated with DMSO or DEVD-fmk for 2 h in 400 μl of medium or STS for 1 h. At the end of the treatment, cells were detached by flushing and 100ul were transferred into 96 well white plate and 100ul of Caspase-Glo reagent was added to each well. The plate was incubated 45 min at room temperature in the dark before reading luminescence in a Tecan Spark Plate reader (Tecan, Männedorf, Switzerland).

### Western blot

Total protein extracts were made directly in Laemmli buffer by scraping of the cells. For immunoblot analysis, protein extracts were resolved on 4–15% gradient precast SDS–polyacrylamide gel electrophoresis (BioRad) and then blotted onto 0.45 µm pore-size nitrocellulose membranes. Membranes were blocked with 0.1% Tween/5% milk in PBS and incubated with indicated primary antibodies, overnight at 4 °C. The day after, membranes were washed 3 times with PBS-T and incubated with the appropriate secondary antibody, RDye® 680RD Goat anti-Rabbit or RDye® 800CW Goat anti-Mouse IgG (1:5000; LI-COR Bioscience, Lincoln, NE, USA) for 1 h at room temperature. Visualization was obtained using an Odyssey CLx infrared imaging system (LI-COR Bioscience). Analysis was made using anti-β-actin as housekeeping gene for standardization of protein loading, and densitometry was done using the ImageJ software normalizing the protein of interest with the housekeeping gene. Details about antibodies used in this study can be found in Supplementary Table 1.

### Caspase 3-dependent substrate proteolysis assay

1.5 μg of COLEC12 (R&D Systems, Cat#3130-CL) and 1 or 2 Units of active CASP3 Recombinant proteins (Millipore, Cat #CC119) were incubated simultaneously in Caspase reaction buffer (50 mM HEPES, pH7.2, 50 mM NaCl, 0.1% CHAPS. 10 mM EDTA, 5% glycerol and 10 mM DTT) for 20 minutes at room temperature. After incubation, Laemmli buffer was added and samples were boiled and processed as described in the western blot section.

### Immunofluorescence

For microscopy analysis, BV2 cells were grown on coverslips for 24 hours and treated with the corresponding reagent for 2 h. Cells were fixed with 4% Paraformaldehyde for 15 min at room temperature, and blocked/permeabilized in HEPES 10 mM, 3% bovine serum albumin, 0.3% Triton X-100 diluted in PBS at room temperature for 1 h. Later, the slides were left overnight in a humidified chamber at 4 °C with the indicated primary antibody diluted on blocking solution. The day after, the samples were washed 3 times with 0.1 Tween/PBS for 5 min and incubated with the corresponding secondary antibody AlexaFluor® 488 Goat anti-Rabbit IgGs or AlexaFluor® 488 Goat anti-Mouse (1:500; Molecular probes/Invitrogen, Waltham, MA, USA) at room temperature for 1 h. Slides were mounted using ProLong Gold antifade mounting medium containing DAPI used to stain the nuclei. Samples were analyzed under Zeiss LSM700 confocal laser scanning microscopy equipped with ZEN Zeiss software. Antibodies are listed in the Supplementary Table 1.

### Proximity Ligation Assay

BV2 cells were grown on coverslips and for 24 h and treated with DEVD-fmk or DMSO as control for 2 h or 100 ng/ml LPS (Sigma Aldrich) for 2 or 6 h. Cells were fixed with 4% paraformaldehyde in PBS for 15 min. Cells were permeabilized 10 minutes in PBS-0.01% Triton X100 and blocked 2 h in the PLA blocking buffer. Then, the In-situ PLA (Duolink II Detection Reagents Red Kit, Olink Bioscience, Uppsala, Sweden) was performed according to the manufacturer’s instruction. The samples were mounted using ProLong Gold antifade mounting medium containing DAPI. Fluorescence signal amplification were observed using Zeiss LSM800 confocal laser scanning microscopy. Antibodies used are listed in Supplementary Table 1.

### *Colec12 andCasp3* silencing by siRNA

Transfection of BV2 cells was carried out with Lipofectamine 3000 (Invitrogen). Non-targeting control (*siSCR*), and *Colec12* or *Casp3* ON-TARGET plus SMARTpools siRNAs, whose sequences can be found in the Supplementary Table 2, were obtained from Dharmacon (Dharmacon, Lafayette, CO, USA).

### Phagocytosis assay

100,000 BV2 per well were seeded in 12 well plates. 24 h after seeding, the cells were treated with DMSO or DEVD-fmk for 2 h. Subsequently, BV2 cells were infected with *S. pneumoniae* serotype 4 strain TIGR4 (gift from Federico Iovino, Karolinska Institutet) with a multiplicity of infection of 10 (MOI 10) for 90 min at 37 °C and 5% CO_2_. Extracellular bacteria were killed by adding 200 μg/ml of gentamicin. At 1, 2, and 4 h after the gentamicin treatment samples were collected for RNA isolation and for the quantification of intracellular bacteria. For the latter, BV2 cells were washed twice and lysed with 1% filtered saponin (Sigma) for 10 min at 37 °C. Cell lysates were diluted 1:5 and 10 or 100 μl were plated on blood agar plates o/n (37 °C, 5% CO_2_). The following day, colony forming units (CFU) were counted.

### Aβ preparation, treatment, and phagocytosis

Beta Amyloid (1–42) Aggregation Kit (rPeptide) was used, and aggregation was performed following manufacturer’s instructions with small adjustments. The formation of aggregates is measured by thioflavin assay by detection of fluorescence at λex 430 nm/λem 485 nm using a Tecan Spark plate reader. Aggregated Aβ was stained with pHrodo™ Red, Succinimidyl Ester (Invitrogen) according to iCell® Microglia Application protocol (FujiFilm Cellular Dynamics, Madison, WI, USA) with minor modifications.

BV2 cells were pre-treated for 2 h with 20 µM DEVD-fmk or DMSO, as vehicle. Subsequently, 0.5 µM pHrodo stained Aβ was added for 3 h. BV2 cells treated the same conditions were cultivated at 4 °C to inhibit phagocytic capacity (corresponding to INHIB condition). Next, cells were washed with PBS and detached from cultivation surface by trypsinization. Cells were afterwards washed and maintained in HBSS until measured by flow cytometry (LSRFortessa, BD). Total percentage of cells positive for pHrodo fluorescence and mean of the fluorescent intensity were evaluated. For RNA isolation, BV2 cells were seeded and let to attach for 24 h. Afterwards, BV2 were treated with 20 µM DEVD-fmk or DMSO for 8 h. At the same time, 0.5 µM aggregated Aβ was added for the equal time of incubation. Cells treated with LPS (100 ng/ml) were pre-treated with DEVD for 6 h and then treated with LPS for additional 2 h.

### RNA isolation, cDNA synthesis, and qPCR

Total RNA was extracted using the RNeasy Mini Kit (Qiagen, Hilden, Germany). RNA concentrations were quantified using NanoDrop® spectrophotometer (Fisher Scientific). cDNA was synthesized from 500 ng to 1 µg RNA using Oligo dT, dNTPs, and Superscript IV Reverse Transcriptase (Invitrogen). qPCR was run on a StepOne plus (Applied Biosystems) using the SYBR™ Green master mix (Fisher Scientific) and primers listed in Supplementary Table 3. *Actin* was used as a housekeeping gene for normalization. Results were calculated using delta Ct method and represented as a fold over control cells (DMSO condition).

### Structural modeling of Collectin-12

The modeled structure of Collectin-12 was generated by Phyre2 web portal for protein modeling, prediction, and analysis [60]. Met^1^-Leu^742^ amino acids sequence of Collectin-12 was submitted to the Phyre2 server, and the structures resulting from the analysis used as 3D model. 378 residues (Lys^60^-Phe^438^), 50.9% coverage of the submitted sequence, were modeled with 95.5% confidence by the single highest scoring template. Model was depicted in the EzMol molecular viewer [61].

### Statistical analysis

Statistical analysis was performed using GraphPad Prism (GraphPad Software, Version 6.0), the threshold for statistical significance was considered when the *p*-value was less than 0.05; *p < 0.05, **p < 0.01, ***p < 0.001. If two conditions were to be compared, two-tailed unpaired Student *t* test was used. Two-way ANOVA was used for multiple comparisons. No samples were excluded from the analyses performed. The number of experimental repeats is described in the relevant figure legends. The investigators were not blinded to allocation during experiments and outcome assessment.

## Supplementary information

Supplementary Figure 1

Supplementary Tables 1-3

Supplementary Table 4

Supplementary Table 5

Supplementary data file 1

Reproducibility checklist

## Data Availability

Immunoblot membranes are presented in a Supplementary data file 1. All other data are available from the corresponding author upon reasonable request.
