# Supplementary material for: Proteome integral solubility alteration high-throughput proteomics assay identifies Collectin-12 as a non-apoptotic microglial caspase-3 substrate
Source: Cell Death Dis. 2023 Mar 11;14(3):192. doi: 10.1038/s41419-023-05714-2 (PMC10008626; doi:10.1038/s41419-023-05714-2)
Supplement: Supplementary file 2 — Supplementary Tables 1-3 [file 41419_2023_5714_MOESM2_ESM.docx]

***Supplementary Information***

| **Primary Antibodies** | **Companies** | **Dilution used** |
| --- | --- | --- |
|  |  |  |
| **β-Actin** (AC40; mouse mAb) | Sigma Aldrich (A-3853) | 1/2000 |
|  |  |  |
| **COLEC12** | R&D (AF3130) | 1/400 |
|  |  |  |
| **COLEC12** | Boster Bio (A07213) | 1/1000 |
|  |  |  |
| **Caspase 3** | Cell Signaling (#9662) | 1/1000 |
|  |  |  |
| **Cleaved Caspase 3** | Cell Signaling (#9662) | 1/400 (IF) 1/1000 (WB) |
|  |  |  |
| **Secondary antibodies** |  |  |
|  |  |  |
| **Alexa Fluor Donkey anti-Goat 488** | | 1/500 |
| **Licor Goat Anti-Mouse IRDye 800CW** | | 1/5000 |
| **Licor Goat Anti-Rabbit IRDye 680RD** | | 1/5000 |
|  |  |  |

**Supplementary Table 1 | Antibodies used in this study**

**Supplementary Table 2 |ON-TARGETplus SMART pool small interfering RNAs used in this study.**

| **ON-TARGET plus SMARTpools siRNAs** | **Company** |
| --- | --- |
|  |  |
| ***Casp3*** (mouse, CASP3 NM_001284409) | Dharmacon (L-043042) |
| GAAAUGGGCAUAUGCAUAA |  |
| CAACGGAAUUCGAGUCCUU |  |
| GGAUAGUGUUUCUAAGGAA |  |
| CGCACAAGCUAGAAUUUAU |  |
|  |  |
| ***Colec12*** (mouse, COLEC12 NM_130449) | Dharmacon (L-045281) |
| ACUCAUUGGUUGAUAGGCA |  |
| ACUCAGAACAGGAAAGCGA |  |
| AGAUAAUAAUAGCGGUACU |  |
| GGUUACAAGAGGUUUGGUA |  |
|  |  |
| **Non-targeting siRNA pool** *(siSCR)* | Dharmacon (D-001810) |
| UGGUUUACAUGUCGACUAA |  |
| UGGUUUACAUGUUGUGUGA |  |
| UGGUUUACAUGUUUUCUGA |  |
| UGGUUUACAUGUUUUCCUA |  |
|  |  |

**Supplementary Table 3 | Primer sequences**

All sequences are given 5´to 3´

| **cDNA (organism)** | **Forward primer** | **Reverse primer** |
| --- | --- | --- |
| ***Actb*** (mouse) | GATGTATGAAGGCTTTGGTC | TGTGCACTTTTATTGGTCTC |
| ***Colec12*** (mouse) | CATCCGCTTGGATTCTATTTC | AGATCCTCTGTCACCTTTTG |
| ***C1qa*** (mouse) | ACGTGGTTATCTTTGACAAG | GATAAACAGACAAAGGTCCC |
| ***Il1β*** (mouse) | GCTGCTTCCAAACCTTTGAC | TTCTCCACAGCCACAATGAG |
| ***Il6*** (mouse) | GGACCAAGACCATCCAATTC | GGCATAACGCACTAGGTTTG |
| ***P52*** (mouse) | TCAAGATCTGTAACTATGAGGG | TTCTTCTTGGTTACATGCAG |
| ***Tnfα*** (mouse) | CTGAGGTCAATCTGCCCAA | CTTCACAGAGCAATGACTCCAAG |
