# Supplementary material for: Proteome integral solubility alteration high-throughput proteomics assay identifies Collectin-12 as a non-apoptotic microglial caspase-3 substrate
Source: Cell Death Dis. 2023 Mar 11;14(3):192. doi: 10.1038/s41419-023-05714-2 (PMC10008626; doi:10.1038/s41419-023-05714-2)
Supplement: Supplementary file 4 — Supplementary Table 5 [file 41419_2023_5714_MOESM4_ESM.pdf]

List of proteins identified as potential microglial caspase-3 substrates (P<0.05)

Protein Symbol	Protein name	UniProt	rank	position	site	N fragment	C fragment	frequency score	similarity maxscore	similarity maxsite	average score	specificity
UPAR	Urokinase plasminogen activator surface receptor	P35456	1	123 to 128	TSLD.QS	14.0 kD	21.4 kD	0.035	53.571	ESVDKS	1.874	>95%
LONP2	Lon protease homolog 2, peroxisomal	Q9DBN5	1	442 to 447	DEVD.KL	50.4 kD	44.1 kD	2.041	90.323	DEVDKM	184.365	>99.9%
FTL1	Ferritin	Q9CPX4	1	39 to 44	DRDD.VA	4.9 kD	15.8 kD	0.024	67.742	DMDDVV	1.617	>95%
NOTC2	Neurogenic locus notch homolog protein 2	O35516	1	1851 to 1856	DAED.SS	199.8 kD	65.8 kD	1.476	75.000	DALDSS	110.665	>99.9%
OSTP	Osteopontin	F8WIP8	1	103 to 108	DSVD.SD	11.7 kD	20.9 kD	6.380	71.875	DTTDSD	458.529	>99.9%
UROK	Urokinase-type plasminogen activator	P06869	1	63 to 68	CEID.AS	7.2 kD	41.0 kD	0.330	78.125	CEVDAL	25.779	>99%
A0A1W2P6U8	Submitted name: RIKEN cDNA 4932415D10 gene	A0A1W2P6U8	1	365 to 370	EITD.SV	41.0 kD	510.8 kD	0.537	81.481	SVTDSV	43.719	>99%
GSLG1	Golgi apparatus protein 1	Q61543	1	702 to 707	DVAD.NQ	79.6 kD	54.1 kD	0.276	87.097	DVVDNQ	24.060	>99%
CEP83	Centrosomal protein of 83 kDa	Q9D5R3	1	337 to 342	SELD.GL	40.3 kD	41.6 kD	5.916	76.667	SDVDGL	453.587	>99.9%
TFR1	Transferrin receptor protein 1	Q62351	1	105 to 110	EETD.KS	12.2 kD	73.5 kD	0.340	73.333	DETDSS	24.898	>99%
GOGA5	Golgin subfamily A member 5	Q9QYE6	1	640 to 645	SGVD.SG	72.5 kD	9.9 kD	1.070	80.000	SGVDIG	85.585	>99.9%
FRIH	Ferritin heavy chain	P09528	1	43 to 48	DRDD.VA	5.4 kD	15.7 kD	0.024	67.742	DMDDVV	1.617	>95%
LRP1	Prolow-density lipoprotein receptor-related protein 1	Q91ZX7	1	3479 to 3484	DCVD.GS	387.4 kD	117.3 kD	5.183	72.222	DCADGM	374.347	>99.9%
COL12	Collectin-12	Q8K4Q8	6	86 to 91	VESD.LK	10.1 kD	71.2 kD	0.098	60.714	KESDLS	5.949	>99%
TF3C6	General transcription factor 3C polypeptide 6	Q9D8P7	1	126 to 131	ENID.GV	14.6 kD	10.9 kD	0.138	75.000	DNVDGL	10.343	>99%
KAD6	Adenylate kinase isoenzyme 6	Q8VCP8	1	95 to 100	LRTD.NG	11.1 kD	8.9 kD	0.023	66.667	IETDSG	1.503	>95%
CTL2B	Protein CTLA-2-beta	P12400	1	95 to 100	EYED.LG	11.5 kD	1.6 kD	0.036	56.250	DYKDIA	1.997	>95%
NEO1	Neogenin	P97798	1	114 to 119	LLPD.GS	12.7 kD	150.4 kD	0.596	70.588	MMPDGT	42.094	>99%
LA	Lupus La protein homolog	P32067	1	99 to 104	EVTD.EY	11.8 kD	35.9 kD	0.046	53.333	EVTDAG	2.447	>95%
TARA	TRIO and F-actin-binding protein	Q99KW3	1	1480 to 1485	DELD.GE	162.2 kD	61.1 kD	12.720	90.625	DEVDGE	1152.764	>99.9%
LMNB1	Lamin-B1	P14733	1	229 to 234	VEVD.SG	26.2 kD	40.5 kD	4.838	100.000	VEVDSG	483.795	>99.9%
FA20C	Extracellular serine/threonine protein kinase FAM20C	Q5MJS3	1	556 to 561	VEKD.GL	63.6 kD	2.2 kD	0.292	60.000	TEKDSM	17.522	>99%
SEM4D	Semaphorin-4D	O09126	1	282 to 287	SKPD.SG	32.0 kD	63.6 kD	0.283	75.758	SQPDTG	21.441	>99%
CATB	Cathepsin B	P10605	1	76 to 81	EDID.LP	8.9 kD	28.4 kD	0.105	78.788	DDVDIP	8.234	>99%
NOSIP	Nitric oxide synthase-interacting protein	Q9D6T0	1	207 to 212	DSVD.RV	23.4 kD	9.8 kD	1.111	71.429	DSLDSV	79.379	>99.9%
A2AGH5	Predicted gene 20489	A2AGH5	1	361 to 366	ELVD.SL	42.4 kD	9.5 kD	3.053	88.889	ELVDSV	271.379	>99.9%
NICA	Nicastrin	P57716	1	84 to 89	VLTD.GP	9.1 kD	69.3 kD	0.849	72.727	EITDGP	61.757	>99.9%
RUXE	Small nuclear ribonucleoprotein E	P62305	1	56 to 61	LVLD.DA	7.1 kD	3.7 kD	0.080	56.667	LVIDNG	4.525	>99%
CSF1R	Macrophage colony-stimulating factor 1 receptor	P09581	1	691 to 696	SEGD.SS	77.6 kD	31.6 kD	0.797	85.185	SESDSS	67.862	>99.9%
TNR1B	Tumor necrosis factor receptor superfamily member 1	P25119	1	196 to 201	ASTD.AV	21.6 kD	28.7 kD	0.354	64.286	SSTDAK	22.761	>99%
LEG9	Galectin 9	O08573	1	245 to 250	VLPD.AT	27.9 kD	12.1 kD	0.107	72.414	VVPDAL	7.752	>99%
PROP	Properdin	P97458	1	34 to 39	STVD.RS	4.0 kD	21.0 kD	0.153	62.069	ETVDTS	9.513	>99%
LGMN	Legumain	O89017	1	24 to 29	DPED.GG	2.8 kD	46.6 kD	0.397	82.353	DEEDGG	32.704	>99%
SRP14	Signal recognition particle 14 kDa protein	P16254	1	59 to 64	RATD.GK	7.0 kD	5.4 kD	0.046	75.000	DATDGK	3.462	>95%
ATPB	ATP synthase subunit beta, mitochondrial	P56480	1	366 to 371	DLTD.PA	38.7 kD	17.6 kD	0.741	82.759	DLTDAA	61.296	>99.9%
IL6RB	Interleukin-6 receptor subunit beta	Q00560	1	798 to 803	DSVD.GG	89.4 kD	13.1 kD	24.037	70.968	DSTDGA	1705.880	>99.9%
ATPA	ATP synthase subunit alpha, mitochondrial	Q03265	1	451 to 456	SDLD.AA	48.8 kD	10.9 kD	1.126	77.778	SELDAS	87.582	>99.9%
DEK	Protein DEK	Q7TNV0	1	307 to 312	ESED.SS	35.0 kD	8.1 kD	0.571	82.759	EQEDSS	47.243	>99%
EED	Polycomb protein EED	Q921E6	1	36 to 41	DEND.DA	4.1 kD	46.1 kD	0.631	75.000	DEEDDS	47.304	>99%
ATRN	Attractin	Q9WU60	1	143 to 148	FVTD.GP	14.9 kD	143.1 kD	0.154	72.727	EITDGP	11.165	>99%
A4	Amyloid-beta A4 protein	P12023	1	194 to 199	DSVD.SA	22.1 kD	64.6 kD	25.873	84.615	SSVDSA	2189.217	>99.9%
CATL1	Procathepsin L	P06797	1	286 to 291	EGTD.SN	32.4 kD	5.1 kD	0.276	61.290	LGTDSD	16.887	>99%
SRSF4	Serine/arginine-rich-splicing factor 4	Q8VE97	1	44 to 49	DLRD.AD	5.6 kD	50.3 kD	0.452	67.742	DLRDGS	30.616	>99%
SYHM	Histidine--tRNA ligase, mitochondrial	Q99K9	1	318 to 323	ISLD.LS	36.4 kD	20.5 kD	0.047	48.276	VSLDSP	2.273	>95%
RALY	RNA-binding protein Raly	Q64012	1	305 to 310	DAED.GA	32.8 kD	0.4 kD	2.461	71.875	DEQDGA	176.912	>99.9%
CATK	Cathepsin K	P55097	1	196 to 201	DSED.AY	22.5 kD	14.3 kD	0.559	66.667	DSLDAF	37.282	>99%
TRA2B	Transformer-2 protein homolog beta	P62996	1	182 to 187	MELD.GR	21.5 kD	12.2 kD	0.512	74.194	TEIDGR	38.002	>99%
TRFE	Serotransferrin	Q921I1	1	74 to 79	SEAD.AM	8.2 kD	68.5 kD	0.336	77.778	SESDAV	26.167	>99%
CATZ	Cathepsin Z	Q9WUU7	1	252 to 257	VSND.GI	28.3 kD	5.7 kD	0.032	75.000	VASDGV	2.394	>95%
PHB1	Prohibitin 1	P67778	1	214 to 219	AEGD.SK	23.9 kD	5.9 kD	0.214	60.714	ASTDSK	12.992	>99%
OGFD1	2-oxoglutarate and iron-dependent oxygenase domain	Q3U0K8	1	147 to 152	EFTD.AL	17.3 kD	45.4 kD	0.331	70.000	DFLDAL	23.149	>99%
NIP7	60S ribosome subunit biogenesis protein NIP7 homolo	Q9CXK8	1	29 to 34	DRPD.GT	3.8 kD	16.6 kD	0.437	72.222	DRHDGT	31.567	>99%
TNR1A	Tumor necrosis factor receptor superfamily member 1	P25118	1	361 to 366	AVVD.GV	39.7 kD	10.5 kD	0.883	72.414	SVVDGT	63.921	>99.9%
SCO1	Protein SCO1 homolog, mitochondrial	Q5SUC9	1	215 to 220	EEID.GV	24.1 kD	7.5 kD	2.916	83.871	DEVDGV	244.535	>99.9%
CASC3	Protein CASC3	Q8K3W3	1	119 to 124	DAAD.SS	12.4 kD	63.4 kD	2.257	82.143	DALDSS	185.372	>99.9%
HIC2	Hypermethylated in cancer 2 protein	Q9JLZ6	1	409 to 414	GSED.SG	43.5 kD	23.3 kD	0.147	53.333	ESMDSG	7.859	>99%
PSA2	Proteasome subunit alpha type-2	P49722	1	183 to 188	ELED.AI	20.6 kD	5.3 kD	0.070	62.963	ELVDSV	4.381	>99%
PCP	Lysosomal Pro-X carboxypeptidase	Q7TMR0	1	438 to 443	DITD.TL	49.2 kD	5.8 kD	0.467	68.966	DITDSS	32.204	>99%
CD68	Macrosialin	P31996	1	18 to 23	TEED.CP	2.3 kD	32.5 kD	0.048	53.333	VEVDAP	2.566	>95%
COX17	Cytochrome c oxidase copper chaperone	P56394	not validated									
U119A	Protein unc-119 homolog A	Q9Z2R6	1	214 to 219	TQSD.SF	24.3 kD	2.7 kD	0.032	67.742	TETDAF	2.148	>95%
U2AF2	Splicing factor U2AF 65 kDa subunit	P26369	1	253 to 258	VVPD.SA	29.4 kD	24.1 kD	0.625	75.862	IVPDIA	47.439	>99%
CATS	Cathepsin S	O70370	1	295 to 300	GTLD.GK	33.6 kD	4.9 kD	0.253	68.750	DTVDGK	17.406	>99%
KGD4	Alpha-ketoglutarate dehydrogenase component 4	Q9CQX8	1	59 to 64	TSPD.LL	6.4 kD	4.6 kD	0.108	58.065	DSPDSL	6.277	>99%
CHCH7	Coiled-coil-helix-coiled-coil-helix domain-containing p	Q8K2Q5	1	18 to 23	SESD.AS	2.4 kD	7.7 kD	1.087	88.889	SESDSS	96.617	>99.9%
PSB8	Proteasome subunit beta type-8	P28063	1	263 to 268	DVSD.LL	29.1 kD	1.1 kD	0.322	67.857	DVLDVL	21.837	>99%
SUMF1	Formylglycine-generating enzyme	Q8R0F3	1	190 to 195	EGPD.SS	20.5 kD	20.1 kD	0.514	67.742	EEPDSA	34.818	>99%
TPP1	Tripeptidyl-peptidase 1	O89023	1	326 to 331	DDED.SL	36.3 kD	25.0 kD	1.669	70.000	DEEDIL	116.813	>99.9%
ZC3H3	Zinc finger CCCH domain-containing protein 3	Q8CHP0	1	82 to 87	ESSD.PA	9.7 kD	93.5 kD	0.047	58.065	GSSDPL	2.714	>95%
HNRPL	Heterogeneous nuclear ribonucleoprotein L	Q8R081	1	181 to 186	DSDD.SR	19.3 kD	44.6 kD	0.383	68.966	DSSDSE	26.383	>99%
ODPA	Pyruvate dehydrogenase E1 component subunit alpha	P35486	1	345 to 350	EIED.AA	38.5 kD	4.7 kD	0.133	62.069	EQEDAS	8.268	>99%
BCL7A	B-cell CLL/lymphoma 7 protein family member A	Q9CXE2	1	135 to 140	AQAD.GK	15.2 kD	7.6 kD	0.155	63.333	AQMDGA	9.817	>99%
CLIC1	Chloride intracellular channel protein 1	Q9Z1Q5	1	150 to 155	EEVD.ET	17.0 kD	10.0 kD	0.263	76.667	DEVDES	20.165	>99%
TRXR1	Thioredoxin reductase 1, cytoplasmic	Q9JMH6	1	88 to 93	STPD.SR	9.9 kD	57.0 kD	0.218	68.966	SVPDSS	15.026	>99%
SYCE2	Synaptonemal complex central element protein 2	Q505B8	1	53 to 58	SSLD.SS	5.9 kD	13.6 kD	1.905	76.923	SSVDSA	146.530	>99.9%
CENPF	Centromere protein F	Q155P7	1	2713 to 2718	SLVD.SN	311.9 kD	30.6 kD	1.125	89.286	SLLDSN	100.445	>99.9%
LC7L2	utative RNA-binding protein Luc7-like 2	Q7TNC4	1	17 to 22	TSRD.GD	2.2 kD	44.3 kD	0.111	66.667	TSTDGS	7.400	>99%
SRSF7	Serine/arginine-rich splicing factor 7	Q8BL97	1	88 to 93	DAED.AV	10.3 kD	20.5 kD	0.570	64.286	DIADAV	36.648	>99%
HMGB2	High mobility group protein B2	P30681	1	198 to 203	EEED.DE	23.0 kD	1.1 kD	0.070	75.000	DEEDDS	5.284	>99%
PPID	Peptidyl-prolyl cis-trans isomerase D	Q9CR16	1	140 to 145	PHLD.GK	15.6 kD	25.1 kD	0.051	78.947	PHLDGP	3.998	>99%
SIAE	Sialate O-acetylesterase	P70665	1	170 to 175	TEVD.LS	18.9 kD	41.9 kD	1.024	71.429	TEVDAA	73.138	>99.9%
ARSA	Arylsulfatase A	P50428	1	251 to 256	MELD.GA	27.2 kD	26.6 kD	2.199	75.758	MELDGP	166.626	>99.9%
KAT3	Kynurenine--oxoglutarate transaminase 3	Q71RI9	1	438 to 443	STLD.AA	49.5 kD	1.6 kD	0.711	67.857	SSLDGA	48.259	>99%
GSTO1	Glutathione S-transferase omega-1	O09131	1	170 to 175	SMVD.YL	19.6 kD	7.9 kD	0.131	57.692	SSVDSL	7.550	>99%
HOT	Hydroxyacid-oxoacid transhydrogenase, mitochondria	Q8R0N6	1	111 to 116	EPTD.GS	12.3 kD	37.6 kD	0.416	63.333	TSTDGS	26.326	>99%
AMPL	Cytosol aminopeptidase	Q9CPY7	1	361 to 366	DNTD.AE	38.9 kD	17.2 kD	0.334	70.000	DETDAS	23.389	>99%
TERA	Transitional endoplasmic reticulum ATPase	Q01853	1	577 to 582	DELD.SI	64.5 kD	24.8 kD	7.179	100.000	DELDSI	717.907	>99.9%
NDKB	Nucleoside diphosphate kinase	Q01768	1	138 to 143	ELID.YK	16.0 kD	1.4 kD	0.100	67.742	ESVDYR	6.789	>99%
MAP11	Methionine aminopeptidase 1	Q8BP48	1	164 to 169	EEID.HA	18.6 kD	24.6 kD	0.343	100.000	EEIDHA	34.286	>99%
TSR2	Pre-rRNA-processing protein TSR2 homolog	Q8C8T8	1	138 to 143	DDVD.SV	15.3 kD	5.6 kD	10.170	86.207	DEVDSI	876.694	>99.9%
AT2B1	Plasma membrane calcium-transporting ATPase 1	P14231	1	136 to 141	EQPD.NG	16.4 kD	16.9 kD	0.191	72.727	SQPDTG	13.911	>99%
PSA7	Proteasome subunit alpha type-7	Q9Z2U0	1	181 to 186	IETD.DL	20.5 kD	7.4 kD	0.104	71.875	DETDDL	7.487	>99%
SNR40	5 small nuclear ribonucleoprotein 40 kDa protein	Q6PE01	1	118 to 123	YNTD.GS	12.9 kD	26.4 kD	0.053	66.667	TSTDGS	3.527	>95%
PNPH	Purine nucleoside phosphorylase	P23492	1	259 to 264	EVLD.AG	29.4 kD	2.8 kD	0.863	90.000	EVMDAG	77.653	>99.9%
HIBCH	3-hydroxyisobutyryl-CoA hydrolase, mitochondrial	Q8QZS1	1	365 to 370	DVTD.ED	41.1 kD	1.9 kD	0.194	75.000	DMTDSD	14.527	>99%
RPP40	Ribonuclease P protein subunit p40	Q8R1F9	1	149 to 154	DLMD.LS	17.4 kD	24.1 kD	0.312	72.414	DMLDLA	22.569	>99%
AGO2	Protein argonaute-2	Q8CJG0	1	250 to 255	PLTD.SQ	28.5 kD	68.7 kD	0.121	64.286	TLTDSS	7.756	>99%
BUB3	Mitotic checkpoint protein BUB3	Q9WVA3	1	58 to 63	AVLD.CA	6.8 kD	30.1 kD	0.059	48.148	AEVDAA	2.865	>95%
G6PE	GDH/6PGL endoplasmic bifunctional protein	Q8CFX1	1	625 to 630	SDPD.SN	71.1 kD	17.8 kD	0.386	65.517	SVPDSS	25.260	>99%

>UPAR _MOUSE_

rank	position	site	N fragment	C fragment	frequency score	similarity maxscore	similarity maxsite	average score	specificity
1	123 to 128	TSLD.QS	14.0 kD	21.4 kD	0.035	53.571	ESVDKS	1.874	>95%
2	247 to 252	TGLD.VL	27.5 kD	7.9 kD	0.026	58.621	TNLDSL	1.513	>95%
3	201 to 206	PVLD.LQ	22.7 kD	12.7 kD	0.013	44.828	DMLDLA	0.600	>95%
4	272 to 277	HVAD.SF	30.1 kD	5.3 kD	0.005	57.576	HLADSP	0.315	>95%
5	158 to 163	SLKD.ED	18.1 kD	17.3 kD	0.004	48.148	SLLDEL	0.193	>95%
6	54 to 59	EWQD.DR	6.3 kD	29.1 kD	0.001	66.667	EGEDDR	0.088	>95%

>LONP
2_MO
USE_

rank	position	site	N fragment	C fragment	frequency score	similarity maxscore	similarity maxsite	average score	specificity
1	442 to 447	DEVD.KL	50.4 kD	44.1 kD	2.041	90.323	DEVDKM	184.365	>99.9%
2	593 to 598	DVAD.GE	67.1 kD	27.4 kD	1.203	72.414	DVVDAE	87.132	>99.9%
3	721 to 726	DLLD.NT	81.0 kD	13.6 kD	0.568	76.667	DVLDNV	43.521	>99%
4	615 to 620	DTAD.LA	69.5 kD	25.1 kD	0.443	75.000	DSVDLA	33.240	>99%
5	31 to 36	TSVD.TA	3.6 kD	90.9 kD	0.398	76.923	SSVDSA	30.594	>99%
6	846 to 851	GLID.SK	94.2 kD	0.3 kD	0.190	68.750	GLLDPK	13.044	>99%
7	833 to 838	AAFD.GG	92.8 kD	1.7 kD	0.101	83.333	AAVDGG	8.386	>99%
8	263 to 268	EEED.ND	30.1 kD	64.4 kD	0.113	66.667	DEEDMD	7.515	>99%
9	69 to 74	DTQD.LP	7.7 kD	86.8 kD	0.091	72.727	DTTDVP	6.623	>99%
10	125 to 130	EQLD.RL	13.9 kD	80.6 kD	0.088	75.000	SELDRL	6.598	>99%
11	325 to 330	STTD.RL	37.4 kD	57.1 kD	0.077	60.714	SELDRL	4.688	>99%
12	604 to 609	VLED.AK	68.3 kD	26.2 kD	0.081	55.172	VLKDTQ	4.489	>99%
13	590 to 595	DRSD.VA	66.9 kD	27.6 kD	0.063	63.333	DKTDIS	3.968	>99%
14	737 to 742	VTKD.GP	82.7 kD	11.8 kD	0.041	63.889	ATHDGP	2.622	>95%
15	406 to 411	DQSD.IR	46.4 kD	48.1 kD	0.045	57.143	DQTDHR	2.590	>95%
16	159 to 164	EMLD.MS	18.1 kD	76.5 kD	0.034	68.966	DMLDLA	2.343	>95%
17	612 to 617	SISD.TA	69.2 kD	25.3 kD	0.037	55.556	SVTDSV	2.081	>95%
18	724 to 729	DNTD.IH	81.3 kD	13.2 kD	0.027	74.194	DNTDLA	1.976	>95%
19	182 to 187	ALPD.IL	20.6 kD	73.9 kD	0.032	60.714	ALDDLI	1.938	>95%
20	328 to 333	DRLD.IR	37.8 kD	56.7 kD	0.034	56.667	DRLDRA	1.919	>95%

>FTL1_MOUSE_

rank	position	site	N fragment	C fragment	frequency score	similarity maxscore	similarity maxsite	average score	specificity
1	39 to 44	DRDD.VA	4.9 kD	15.8 kD	0.024	67.742	DMDDVV	1.617	>95%
2	110 to 115	ALLD.LH	13.0 kD	7.8 kD	0.023	50.000	SLLDSN	1.127	>95%
3	11 to 16	TEVE.AA	1.7 kD	19.1 kD	0.009	85.714	TEVDAA	0.749	>95%
4	38 to 43	FDRD.DV	4.8 kD	15.9 kD	0.003	53.125	VDFDDI	0.180	>95%
5	128 to 133	DFLE.SH	14.9 kD	5.8 kD	0.001	63.636	DELDSH	0.064	>95%
6	78 to 83	LFQD.VQ	9.4 kD	11.4 kD	0.001	44.828	VLKDTQ	0.058	>95%
7	69 to 74	FQND.RG	8.4 kD	12.4 kD	0.001	48.276	LSVDRG	0.056	>95%

>NOT C2_MOUSE_

rank	position	site	N fragment	C fragment	frequency score	similarity maxscore	similarity maxsite	average score	specificity
1	1851 to 1856	DAED.SS	199.8 kD	65.8 kD	1.476	75.000	DALDSS	110.665	>99.9%
2	586 to 591	DGID.SY	62.8 kD	202.8 kD	0.586	76.667	DSIDSF	44.891	>99%
3	2150 to 2155	SPVD.SL	232.3 kD	33.3 kD	0.363	80.769	SSVDSL	29.346	>99%
4	1039 to 1044	TCVD.GL	111.0 kD	154.6 kD	0.420	68.966	TVADGL	28.948	>99%
5	1530 to 1535	DGLD.CA	164.3 kD	101.3 kD	0.346	71.429	DALDAA	24.732	>99%
6	274 to 279	VCVD.GV	29.6 kD	236.0 kD	0.292	67.857	VVADGV	19.816	>99%
7	1818 to 1823	DVLD.VN	196.4 kD	69.2 kD	0.252	75.000	DVLDVL	18.925	>99%
8	434 to 439	VNTD.GA	46.5 kD	219.2 kD	0.253	63.333	TSTDGS	16.015	>99%
9	1936 to 1941	TDLD.AR	208.9 kD	56.8 kD	0.142	74.074	SSLDAR	10.522	>99%
10	1815 to 1820	QEVD.VL	196.1 kD	69.5 kD	0.114	67.857	QEIDSI	7.731	>99%
11	1001 to 1006	TCVD.GI	107.1 kD	158.5 kD	0.121	62.069	TVADGL	7.526	>99%
12	658 to 663	VCVD.GI	70.5 kD	195.1 kD	0.103	64.286	VVADGV	6.596	>99%
13	1358 to 1363	IHTD.SG	145.2 kD	120.4 kD	0.078	83.333	IETDSG	6.521	>99%
14	2005 to 2010	DMQD.NK	216.1 kD	49.5 kD	0.067	87.500	DMQDNS	5.823	>99%
15	1848 to 1853	EDED.AE	199.5 kD	66.1 kD	0.083	68.966	EQEDAS	5.738	>99%
16	1889 to 1894	SRAD.AA	203.9 kD	61.7 kD	0.083	67.857	SQTDAA	5.630	>99%
17	2038 to 2043	DITD.HM	219.9 kD	45.7 kD	0.073	76.471	DLKDHM	5.555	>99%
18	1844 to 1849	DLSD.ED	199.0 kD	66.6 kD	0.065	65.625	DMTDSD	4.248	>99%
19	1627 to 1632	LEID.NR	175.3 kD	90.4 kD	0.066	61.290	TEIDGR	4.034	>99%
20	2123 to 2128	EAKD.AK	229.3 kD	36.3 kD	0.051	57.143	TAVDAK	2.937	>95%

>OSTP
_MOU
SE_

rank	position	site	N fragment	C fragment	frequency score	similarity maxscore	similarity maxsite	average score	specificity
1	103 to 108	DSVD.SD	11.7 kD	20.9 kD	6.380	71.875	DTTDSD	458.529	>99.9%
2	240 to 245	DVID.SQ	26.6 kD	6.0 kD	1.776	86.207	DLVDSQ	153.113	>99.9%
3	108 to 113	DESD.ES	12.2 kD	20.4 kD	0.677	80.000	DEVDES	54.190	>99.9%
4	92 to 97	DDDD.GD	10.5 kD	22.0 kD	0.402	76.471	DDLDGD	30.739	>99%
5	100 to 105	ESED.SV	11.4 kD	21.2 kD	0.377	62.069	EQEDSS	23.411	>99%
6	117 to 122	DESD.ET	13.2 kD	19.3 kD	0.206	70.000	DEVDES	14.394	>99%
7	169 to 174	DATD.ED	18.8 kD	13.8 kD	0.149	71.875	DTTDSD	10.740	>99%
8	91 to 96	DDDD.DG	10.4 kD	22.2 kD	0.118	71.429	DEDDDD	8.398	>99%
9	20 to 25	KVTD.SG	2.5 kD	30.1 kD	0.098	76.667	EVTDAG	7.499	>99%
10	94 to 99	DDGD.HA	10.7 kD	21.9 kD	0.109	66.667	DDSDAA	7.287	>99%
11	90 to 95	DDDD.DD	10.3 kD	22.3 kD	0.040	91.429	DEDDDD	3.651	>95%
12	88 to 93	DDDD.DD	10.1 kD	22.5 kD	0.040	91.429	DEDDDD	3.651	>95%
13	87 to 92	DDDD.DD	10.0 kD	22.6 kD	0.040	91.429	DEDDDD	3.651	>95%
14	89 to 94	DDDD.DD	10.2 kD	22.4 kD	0.040	91.429	DEDDDD	3.651	>95%
15	86 to 91	DDDD.DD	9.8 kD	22.7 kD	0.040	91.429	DEDDDD	3.651	>95%
16	214 to 219	SQLD.EP	23.6 kD	8.9 kD	0.053	60.714	SQLDSM	3.232	>95%
17	186 to 191	ESLD.VI	20.7 kD	11.9 kD	0.052	60.714	DSLDSV	3.128	>95%
18	237 to 242	DQSD.VI	26.3 kD	6.3 kD	0.046	65.517	DQLDAI	3.028	>95%
19	234 to 239	ESAD.QS	25.9 kD	6.6 kD	0.030	71.429	ESVDKS	2.114	>95%
20	83 to 88	DHMD.DD	9.5 kD	23.1 kD	0.020	60.000	DEDDDD	1.229	>95%

>UROK_MOUSE_

rank	position	site	N fragment	C fragment	frequency score	similarity maxscore	similarity maxsite	average score	specificity
1	63 to 68	CEID.AS	7.2 kD	41.0 kD	0.330	78.125	CEVDAL	25.779	>99%
2	324 to 329	SESD.YL	36.4 kD	11.9 kD	0.215	66.667	SESDAV	14.346	>99%
3	160 to 165	SSVD.QQ	18.1 kD	30.2 kD	0.058	65.385	SSVDSA	3.800	>99%
4	83 to 88	ANTD.TK	9.4 kD	38.9 kD	0.014	78.571	ASTDSK	1.065	>95%
5	25 to 30	GAPD.ES	2.9 kD	45.4 kD	0.017	58.621	SVPDSS	1.003	>95%
6	305 to 310	RFTD.AP	34.4 kD	13.8 kD	0.010	54.545	EITDGP	0.568	>95%
7	374 to 379	CKGD.SG	42.2 kD	6.1 kD	0.010	51.515	CVVDAG	0.492	>95%
8	267 to 272	YRED.SL	30.2 kD	18.0 kD	0.007	41.379	EQEDSS	0.296	>95%
9	74 to 79	GNGD.SY	8.4 kD	39.9 kD	0.004	63.636	GQKDSY	0.268	>95%
10	148 to 153	MVHD.CS	16.8 kD	31.4 kD	0.004	48.387	TSHDAS	0.177	>95%
11	108 to 113	HRPD.AI	12.1 kD	36.1 kD	0.002	58.065	EEPDSI	0.103	>95%
12	186 to 191	TEVE.NQ	21.0 kD	27.3 kD	0.001	63.636	TEVDNY	0.069	>95%
13	363 to 368	CAAD.PE	41.0 kD	7.3 kD	0.001	42.857	IEADSE	0.055	>95%
14	308 to 313	DAPF.GS	34.7 kD	13.5 kD	0.001	50.000	DSPDGQ	0.053	>95%

>A0A1
W2P6U
8_MOU
SE_

rank	position	site	N fragment	C fragment	frequency score	similarity maxscore	similarity maxsite	average score	specificity
1	365 to 370	EITD.SV	41.0 kD	510.8 kD	0.537	81.481	SVTDSV	43.719	>99%
2	375 to 380	DKTD.LS	42.1 kD	509.8 kD	0.430	93.333	DKTDIS	40.174	>99%
3	825 to 830	ESVD.LL	90.8 kD	461.0 kD	0.576	67.857	DSVDLA	39.103	>99%
4	3152 to 3157	DLND.SR	347.1 kD	204.7 kD	0.344	63.636	DLNDGT	21.860	>99%
5	3429 to 3434	SEPD.LQ	378.0 kD	173.8 kD	0.348	61.290	DEEDLQ	21.346	>99%
6	2336 to 2341	VDFD.SG	258.6 kD	293.3 kD	0.128	72.414	VEVDSG	9.281	>99%
7	3073 to 3078	QGVD.SS	338.2 kD	213.6 kD	0.141	65.625	DGVDNS	9.273	>99%
8	589 to 594	KVPD.SS	65.2 kD	486.6 kD	0.065	86.207	SVPDSS	5.639	>99%
9	530 to 535	KVPD.SS	58.9 kD	492.9 kD	0.065	86.207	SVPDSS	5.639	>99%
10	2334 to 2339	TSVD.FD	258.3 kD	293.5 kD	0.076	68.750	TEVDFN	5.244	>99%
11	644 to 649	KVLD.SS	71.0 kD	480.9 kD	0.085	60.714	DALDSS	5.163	>99%
12	115 to 120	ESYD.TS	13.5 kD	538.3 kD	0.067	68.966	ETVDTS	4.592	>99%
13	430 to 435	NIVD.SR	48.1 kD	503.7 kD	0.064	68.966	NSVDSK	4.437	>99%
14	2467 to 2472	KALD.GK	272.8 kD	279.0 kD	0.057	74.194	NAIDGK	4.226	>99%
15	3591 to 3596	GNTD.SV	395.9 kD	155.9 kD	0.068	59.259	SVTDSV	4.047	>99%
16	3465 to 3470	TESD.RV	382.2 kD	169.6 kD	0.056	70.370	SESDAV	3.967	>99%
17	1352 to 1357	LMFD.SA	148.8 kD	403.0 kD	0.054	71.429	SLFDSA	3.846	>99%
18	144 to 149	CSTD.YS	16.6 kD	535.2 kD	0.063	60.526	CDTDFG	3.785	>99%
19	3422 to 3427	SDQD.SI	377.3 kD	174.6 kD	0.053	66.667	DEQDSV	3.517	>95%
20	3895 to 3900	SSVD.KT	429.6 kD	122.2 kD	0.047	71.429	ESVDKS	3.393	>95%

>GSLG 1_MOUSE_

rank	position	site	N fragment	C fragment	frequency score	similarity maxscore	similarity maxsite	average score	specificity
1	702 to 707	DVAD.NQ	79.6 kD	54.1 kD	0.276	87.097	DVVDNQ	24.060	>99%
2	658 to 663	DHLD.DL	74.7 kD	59.0 kD	0.256	65.625	DETDDL	16.809	>99%
3	889 to 894	PEAD.SK	101.6 kD	32.1 kD	0.195	63.333	DEVDSK	12.363	>99%
4	706 to 711	NQID.SG	80.1 kD	53.6 kD	0.105	67.742	DELDSG	7.089	>99%
5	814 to 819	LEPD.LY	92.8 kD	41.0 kD	0.095	54.286	LEVDCY	5.151	>99%
6	709 to 714	DSGD.LM	80.3 kD	53.4 kD	0.060	70.968	DMGDLV	4.263	>99%
7	991 to 996	SALD.YR	113.4 kD	20.3 kD	0.046	66.667	SSLDAR	3.060	>95%
8	485 to 490	QETD.PG	54.5 kD	79.2 kD	0.042	68.966	QETDSA	2.901	>95%
9	418 to 423	EMLD.YR	46.8 kD	86.9 kD	0.029	74.194	ESVDYR	2.168	>95%
10	870 to 875	PELD.YT	99.3 kD	34.4 kD	0.031	62.500	PELDGS	1.966	>95%
11	635 to 640	CLID.LG	72.0 kD	61.7 kD	0.027	72.727	CVVDAG	1.957	>95%
12	252 to 257	GEKD.AH	27.6 kD	106.1 kD	0.022	66.667	GQKDSY	1.483	>95%
13	678 to 683	ESED.IQ	76.9 kD	56.8 kD	0.020	71.429	ESQDVS	1.444	>95%
14	907 to 912	ELMD.PK	103.7 kD	30.0 kD	0.014	68.750	GLLDPK	0.930	>95%
15	563 to 568	CQGD.AS	63.7 kD	70.1 kD	0.017	50.000	CEVDAL	0.856	>95%
16	162 to 167	LTTD.PK	17.4 kD	116.3 kD	0.014	53.571	ASTDSK	0.742	>95%
17	199 to 204	CLVD.HR	21.5 kD	112.2 kD	0.014	48.485	CVVDAG	0.698	>95%
18	625 to 630	VKLD.PA	70.9 kD	62.8 kD	0.012	58.065	VEVDPM	0.686	>95%
19	1126 to 1131	GFSD.LA	128.6 kD	5.1 kD	0.011	48.148	TIADLA	0.543	>95%
20	1067 to 1072	CALD.IK	121.9 kD	11.9 kD	0.010	50.000	TAVDAK	0.509	>95%

>CEP8
3_MO
USE_

rank	position	site	N fragment	C fragment	frequency score	similarity maxscore	similarity maxsite	average score	specificity
1	337 to 342	SELD.GL	40.3 kD	41.6 kD	5.916	76.667	SDVDGL	453.587	>99.9%
2	479 to 484	SEND.LL	57.0 kD	24.9 kD	0.096	59.259	SESDAV	5.715	>99%
3	313 to 318	EITD.IK	37.5 kD	44.4 kD	0.042	65.517	DVTDIA	2.754	>95%
4	190 to 195	LSVD.PT	23.4 kD	58.6 kD	0.020	54.839	VEVDPM	1.107	>95%
5	194 to 199	PTRD.SK	23.9 kD	58.1 kD	0.021	50.000	ASTDSK	1.035	>95%
6	511 to 516	AQLD.VE	60.8 kD	21.2 kD	0.015	48.387	TELDMD	0.706	>95%
7	342 to 347	LQSD.NE	40.8 kD	41.1 kD	0.008	60.714	IEADSE	0.512	>95%
8	361 to 366	VEKD.RE	43.0 kD	39.0 kD	0.008	60.714	IEADSE	0.470	>95%
9	78 to 83	LLED.LR	9.7 kD	72.3 kD	0.008	42.857	ALDDLI	0.349	>95%
10	384 to 389	VLQD.EK	45.8 kD	36.2 kD	0.006	51.724	VLKDTQ	0.287	>95%
11	25 to 30	MLID.ER	3.1 kD	78.8 kD	0.004	51.852	SLLDEL	0.219	>95%
12	178 to 183	LEKD.KE	22.0 kD	60.0 kD	0.003	60.714	IEADSE	0.181	>95%
13	404 to 409	VERD.VW	48.2 kD	33.8 kD	0.004	41.379	IETDAM	0.155	>95%
14	10 to 15	PGGD.SR	1.4 kD	80.6 kD	0.003	45.161	DGADST	0.138	>95%
15	489 to 494	MLKD.MV	58.2 kD	23.8 kD	0.001	46.429	ALDDLI	0.066	>95%
16	577 to 582	EVLE.AK	68.9 kD	13.1 kD	0.001	50.000	EVMDAG	0.059	>95%
17	395 to 400	RLSD.LE	47.1 kD	34.9 kD	0.001	46.429	KESDLS	0.058	>95%
18	685 to 690	EELG.SP	81.6 kD	0.4 kD	0.001	61.290	EEADSP	0.053	>95%

>TFR1_MOUSE_

rank	position	site	N fragment	C fragment	frequency score	similarity maxscore	similarity maxsite	average score	specificity
1	105 to 110	EETD.KS	12.2 kD	73.5 kD	0.340	73.333	DETDSS	24.898	>99%
2	293 to 298	VEAD.LA	33.3 kD	52.4 kD	0.233	66.667	VEVDAA	15.537	>99%
3	399 to 404	EEPD.RY	45.0 kD	40.7 kD	0.153	67.742	EEPDSI	10.398	>99%
4	562 to 567	EDAD.YP	62.6 kD	23.1 kD	0.068	70.968	EEADSP	4.821	>99%
5	409 to 414	AQRD.AL	46.1 kD	39.6 kD	0.055	56.250	AQRDSH	3.085	>95%
6	27 to 32	RQVD.GD	3.4 kD	82.3 kD	0.037	74.194	AEVDGD	2.779	>95%
7	634 to 639	DIRD.MG	71.0 kD	14.7 kD	0.039	60.000	DAADLG	2.367	>95%
8	538 to 543	LSFD.NA	60.1 kD	25.6 kD	0.036	61.290	LVFDNQ	2.193	>95%
9	518 to 523	HPVD.GK	57.7 kD	28.0 kD	0.025	67.647	YPVDGL	1.660	>95%
10	114 to 119	ETED.VP	13.2 kD	72.5 kD	0.020	69.697	DTTDVP	1.380	>95%
11	525 to 530	LYRD.SN	58.5 kD	27.2 kD	0.023	57.576	VYRDGT	1.337	>95%
12	29 to 34	VDGD.NS	3.5 kD	82.2 kD	0.019	64.516	MDVDNS	1.230	>95%
13	659 to 664	LTTD.FH	73.9 kD	11.8 kD	0.009	60.000	TATDYH	0.562	>95%
14	457 to 462	TAGD.FG	51.0 kD	34.7 kD	0.009	62.500	DAGDVG	0.552	>95%
15	161 to 166	SQKD.ES	18.6 kD	67.2 kD	0.008	59.259	SESDSS	0.488	>95%
16	46 to 51	ENAD.NN	5.4 kD	80.3 kD	0.009	56.667	ESLDNQ	0.488	>95%
17	571 to 576	TRLD.TY	63.7 kD	22.0 kD	0.008	57.576	TEVDNY	0.462	>95%
18	141 to 146	EFAD.TI	16.3 kD	69.4 kD	0.008	55.556	ELVDSV	0.456	>95%
19	440 to 445	ISKD.GF	49.2 kD	36.5 kD	0.006	60.714	VSVDAF	0.374	>95%
20	43 to 48	DEEE.NA	5.1 kD	80.6 kD	0.004	64.516	DEDDSA	0.263	>95%

>GOG A5_M OUSE_

rank	position	site	N fragment	C fragment	frequency score	similarity maxscore	similarity maxsite	average score	specificity
1	640 to 645	SGVD.SG	72.5 kD	9.9 kD	1.070	80.000	SGVDIG	85.585	>99.9%
2	425 to 430	ELVD.YK	47.5 kD	34.8 kD	0.371	70.968	ESVDYR	26.359	>99%
3	116 to 121	SEPD.DE	13.1 kD	69.3 kD	0.181	62.500	SEPDGA	11.302	>99%
4	380 to 385	MEVD.RQ	42.3 kD	40.1 kD	0.101	64.286	MEVDAA	6.465	>99%
5	283 to 288	AQVD.DL	31.3 kD	51.1 kD	0.088	57.692	SSVDSL	5.098	>99%
6	182 to 187	NSSD.SV	19.8 kD	62.5 kD	0.076	60.714	DSLDSV	4.629	>99%
7	211 to 216	TPSD.GS	22.8 kD	59.5 kD	0.052	70.000	TSTDGS	3.656	>95%
8	353 to 358	HEAD.AT	39.1 kD	43.3 kD	0.026	65.517	NEADAL	1.673	>95%
9	293 to 298	AAKD.SQ	32.3 kD	50.1 kD	0.021	55.172	VLKDTQ	1.146	>95%
10	65 to 70	SAAD.NI	7.6 kD	74.8 kD	0.020	53.846	SSVDSL	1.067	>95%
11	90 to 95	TVGD.AT	10.2 kD	72.2 kD	0.018	48.387	DTGDAV	0.877	>95%
12	48 to 53	QNTD.SN	5.8 kD	76.6 kD	0.014	62.069	QETDSA	0.871	>95%
13	514 to 519	DLQD.QI	57.8 kD	24.5 kD	0.011	56.667	DEQDSV	0.631	>95%
14	543 to 548	MEED.LH	61.5 kD	20.9 kD	0.011	54.839	DEEDLQ	0.630	>95%
15	287 to 292	DLTE.AV	31.7 kD	50.6 kD	0.008	72.414	DLTDAA	0.593	>95%
16	122 to 127	LLFD.FL	13.8 kD	68.5 kD	0.012	39.286	SLFDSA	0.454	>95%
17	307 to 312	QEAD.QV	33.8 kD	48.5 kD	0.006	64.286	QEIDSI	0.414	>95%
18	503 to 508	SEAE.SA	56.5 kD	25.8 kD	0.006	67.857	SETDSA	0.386	>95%
19	676 to 681	SSID.QF	76.3 kD	6.0 kD	0.005	66.667	DSIDSF	0.362	>95%
20	493 to 498	ELQD.ME	55.4 kD	26.9 kD	0.005	53.571	ESQDVS	0.264	>95%

>FRIH_MOUSE_

rank	position	site	N fragment	C fragment	frequency score	similarity maxscore	similarity maxsite	average score	specificity
1	43 to 48	DRDD.VA	5.4 kD	15.7 kD	0.024	67.742	DMDDVV	1.617	>95%
2	148 to 153	ELGD.HV	17.7 kD	3.4 kD	0.014	55.882	DLKDHM	0.764	>95%
3	121 to 126	LATD.KN	14.5 kD	6.6 kD	0.006	56.250	LQTDGN	0.365	>95%
4	42 to 47	FDRD.DV	5.2 kD	15.8 kD	0.003	53.125	VDFDDI	0.180	>95%
5	124 to 129	DKND.PH	14.8 kD	6.2 kD	0.003	58.333	DINDGH	0.150	>95%
6	90 to 95	DRDD.WE	11.0 kD	10.0 kD	0.001	65.789	DESDWD	0.097	>95%
7	177 to 182	GHGD.ES	20.8 kD	0.2 kD	0.001	56.250	SHTDQS	0.077	>95%
8	82 to 87	FLQD.IK	10.1 kD	11.0 kD	0.001	50.000	FIQDRA	0.071	>95%
9	13 to 18	YHQD.AE	1.8 kD	19.2 kD	0.002	40.625	LHTDSR	0.069	>95%

>LRP1_MOUSE_

rank	position	site	N fragment	C fragment	frequency score	similarity maxscore	similarity maxsite	average score	specificity
1	3479 to 3484	DCVD.GS	387.4 kD	117.3 kD	5.183	72.222	DCADGM	374.347	>99.9%
2	2800 to 2805	DCTD.GA	311.6 kD	193.1 kD	4.236	83.871	DSTDGA	355.309	>99.9%
3	53 to 58	DCPD.GS	6.1 kD	498.7 kD	2.057	70.588	DSPDGQ	145.190	>99.9%
4	2843 to 2848	DCAD.GS	316.5 kD	188.2 kD	1.070	83.333	DCADGM	89.130	>99.9%
5	3600 to 3605	DCAD.GS	400.9 kD	103.8 kD	1.070	83.333	DCADGM	89.130	>99.9%
6	3217 to 3222	ASLD.GS	358.2 kD	146.5 kD	0.965	78.571	SSLDGA	75.813	>99.9%
7	3773 to 3778	DEED.CS	420.3 kD	84.4 kD	0.685	71.875	DEEDDS	49.266	>99%
8	1255 to 1260	LEPD.GE	139.2 kD	365.5 kD	0.645	65.625	SEPDGA	42.310	>99%
9	836 to 841	LDTD.GV	93.7 kD	411.0 kD	0.538	74.194	MDIDGV	39.889	>99%
10	3638 to 3643	DCMD.GS	405.2 kD	99.5 kD	0.535	69.444	DCADGM	37.138	>99%
11	98 to 103	DCMD.GS	10.9 kD	493.8 kD	0.535	69.444	DCADGM	37.138	>99%
12	1088 to 1093	DCMD.SS	121.4 kD	383.3 kD	0.418	78.571	DALDSS	32.836	>99%
13	879 to 884	DCLD.NS	98.4 kD	406.3 kD	0.425	75.000	DALDSS	31.874	>99%
14	3445 to 3450	DERD.CP	383.5 kD	121.2 kD	0.477	64.865	DITDCP	30.964	>99%
15	2685 to 2690	DERD.CP	299.0 kD	205.8 kD	0.477	64.865	DITDCP	30.964	>99%
16	2591 to 2596	DCGD.GS	288.8 kD	215.9 kD	0.370	75.000	DSGDGA	27.767	>99%
17	1172 to 1177	DCGD.GS	130.3 kD	374.4 kD	0.370	75.000	DSGDGA	27.767	>99%
18	2930 to 2935	DCGD.GS	325.9 kD	178.8 kD	0.370	75.000	DSGDGA	27.767	>99%
19	2759 to 2764	DCGD.GS	307.4 kD	197.4 kD	0.370	75.000	DSGDGA	27.767	>99%
20	3767 to 3772	DCGD.GS	419.7 kD	85.1 kD	0.370	75.000	DSGDGA	27.767	>99%

>COL1
2_MO
USE_

rank	position	site	N fragment	C fragment	frequency score	similarity maxscore	similarity maxsite	average score	specificity
1	667 to 672	GLTD.SE	73.2 kD	8.1 kD	0.209	60.714	TLTDSS	12.678	>99%
2	114 to 119	DILD.LR	13.1 kD	68.2 kD	0.161	68.966	DMLDLA	11.122	>99%
3	425 to 430	KLVD.SK	48.5 kD	32.7 kD	0.141	62.963	ELVDSV	8.854	>99%
4	145 to 150	SLVD.RQ	16.6 kD	64.6 kD	0.130	62.069	DLVDSQ	8.098	>99%
5	67 to 72	NVTD.GM	8.0 kD	73.3 kD	0.099	67.742	DIVDGM	6.720	>99%
6	86 to 91	VESD.LK	10.1 kD	71.2 kD	0.098	60.714	KESDLS	5.949	>99%
7	683 to 688	SPVD.YK	75.0 kD	6.2 kD	0.044	62.500	QEIDYK	2.763	>95%
8	313 to 318	DLQD.LH	35.6 kD	45.7 kD	0.047	50.000	DTQDAN	2.355	>95%
9	339 to 344	FETD.IV	38.8 kD	42.5 kD	0.037	55.172	IETDAM	2.025	>95%
10	370 to 375	TCTD.TL	42.2 kD	39.1 kD	0.031	62.500	SCTDSS	1.961	>95%
11	226 to 231	SVDD.TS	25.9 kD	55.4 kD	0.026	62.069	SVPDSS	1.586	>95%
12	225 to 230	QSVD.DT	25.7 kD	55.5 kD	0.018	62.500	DQIDDT	1.113	>95%
13	678 to 683	KWLD.GS	74.6 kD	6.7 kD	0.016	71.053	YWIDGS	1.105	>95%
14	408 to 413	SKLD.TE	46.7 kD	34.6 kD	0.019	55.556	SELDAS	1.059	>95%
15	310 to 315	SLKD.LQ	35.2 kD	46.0 kD	0.014	58.621	VLKDTQ	0.850	>95%
16	63 to 68	EKMD.NV	7.6 kD	73.7 kD	0.012	64.516	DQLDNI	0.772	>95%
17	141 to 146	ANGD.SL	16.2 kD	65.1 kD	0.012	58.065	DEGDSL	0.696	>95%
18	181 to 186	LQQD.TS	20.8 kD	60.5 kD	0.009	55.172	MEIDTS	0.517	>95%
19	532 to 537	PGKD.GL	58.8 kD	22.5 kD	0.008	60.606	DGTDGL	0.489	>95%
20	392 to 397	IRLD.SI	44.7 kD	36.6 kD	0.009	51.724	DELDSI	0.477	>95%

>TF3C
6_MO
USE_

rank	position	site	N fragment	C fragment	frequency score	similarity maxscore	similarity maxsite	average score	specificity
1	126 to 131	ENID.GV	14.6 kD	10.9 kD	0.138	75.000	DNVDGL	10.343	>99%
2	63 to 68	MQVD.SY	7.4 kD	18.1 kD	0.179	57.576	TEVDNY	10.298	>99%
3	37 to 42	GIID.SD	4.5 kD	21.1 kD	0.031	53.571	SLLDSN	1.641	>95%
4	73 to 78	EYED.TL	8.6 kD	16.9 kD	0.027	57.143	DYEDFM	1.550	>95%
5	194 to 199	EVED.PA	22.4 kD	3.1 kD	0.027	51.613	VMRDPA	1.395	>95%
6	215 to 220	GTQD.GN	24.6 kD	0.9 kD	0.019	66.667	DAQDGN	1.298	>95%
7	154 to 159	EHED.EA	18.1 kD	7.5 kD	0.012	60.000	EHNDGA	0.741	>95%
8	94 to 99	EGTD.KT	10.8 kD	14.7 kD	0.010	61.290	DGADST	0.640	>95%
9	89 to 94	ERVD.PE	10.3 kD	15.2 kD	0.010	58.065	ERVDSP	0.574	>95%
10	9 to 14	AAVD.PI	1.1 kD	24.4 kD	0.010	55.556	MAVDAV	0.528	>95%
11	39 to 44	IDSD.FL	4.7 kD	20.9 kD	0.007	58.621	LSSDFT	0.389	>95%
12	54 to 59	LGID.TE	6.4 kD	19.2 kD	0.005	58.065	LGTDSD	0.308	>95%
13	169 to 174	ELED.QE	19.6 kD	6.0 kD	0.006	48.387	AMEDGE	0.270	>95%
14	206 to 211	SEME.SS	23.6 kD	1.9 kD	0.002	66.667	SESDSS	0.146	>95%
15	157 to 162	DEAA.GP	18.3 kD	7.2 kD	0.002	58.065	DEADGS	0.121	>95%
16	193 to 198	SEVE.DP	22.3 kD	3.2 kD	0.002	46.667	VEVDAP	0.087	>95%
17	137 to 142	KDND.FS	15.9 kD	9.6 kD	0.001	64.286	KESDLS	0.087	>95%
18	162 to 167	PASD.KP	18.8 kD	6.8 kD	0.001	62.857	PAPDAP	0.079	>95%

>KAD6_MOUSE_

rank	position	site	N fragment	C fragment	frequency score	similarity maxscore	similarity maxsite	average score	specificity
1	95 to 100	LRTD.NG	11.1 kD	8.9 kD	0.023	66.667	IETDSG	1.503	>95%
2	42 to 47	QLYD.GY	4.8 kD	15.2 kD	0.020	51.515	EELDGY	1.007	>95%
3	74 to 79	VIVD.YH	8.4 kD	11.5 kD	0.015	57.143	TATDYH	0.838	>95%
4	45 to 50	DGYD.EE	5.1 kD	14.8 kD	0.014	53.125	DGVDNS	0.726	>95%
5	33 to 38	NVGD.LA	3.7 kD	16.2 kD	0.008	62.963	TIADLA	0.491	>95%
6	56 to 61	LDED.RV	6.4 kD	13.6 kD	0.008	50.000	LDVDSL	0.383	>95%
7	60 to 65	RVVD.EL	6.8 kD	13.1 kD	0.006	62.963	RAIDAL	0.377	>95%
8	63 to 68	DELE.HQ	7.2 kD	12.7 kD	0.002	71.429	DELDHN	0.148	>95%
9	151 to 156	QLED.NI	17.7 kD	2.2 kD	0.003	51.852	ELVDSV	0.137	>95%
10	54 to 59	PILD.ED	6.1 kD	13.8 kD	0.002	44.444	SLLDEL	0.071	>95%

>CTL2 B_MOUSE_

rank	position	site	N fragment	C fragment	frequency score	similarity maxscore	similarity maxsite	average score	specificity
1	95 to 100	EYED.LG	11.5 kD	1.6 kD	0.036	56.250	DYKDIA	1.997	>95%
2	89 to 94	MAPD.LP	10.7 kD	2.4 kD	0.022	60.000	PAPDAP	1.310	>95%
3	10 to 15	PSLD.NE	1.3 kD	11.8 kD	0.016	70.000	ESLDNQ	1.090	>95%
4	6 to 11	PSPD.PS	0.9 kD	12.2 kD	0.009	54.286	PAPDAP	0.506	>95%
5	27 to 32	YSLD.EE	3.4 kD	9.7 kD	0.005	46.667	ESLDNQ	0.242	>95%
6	66 to 71	QFSD.LT	8.2 kD	4.9 kD	0.001	50.000	EETDLT	0.053	>95%

>NEO1_MOUSE_

rank	position	site	N fragment	C fragment	frequency score	similarity maxscore	similarity maxsite	average score	specificity
1	114 to 119	LLPD.GS	12.7 kD	150.4 kD	0.596	70.588	MMPDGT	42.094	>99%
2	223 to 228	TEGD.GG	24.5 kD	138.6 kD	0.523	75.000	TELDGG	39.196	>99%
3	865 to 870	SEVD.LF	94.7 kD	68.5 kD	0.304	54.839	TETDAF	16.660	>99%
4	1371 to 1376	QEED.SG	150.5 kD	12.7 kD	0.178	74.194	EERDSG	13.208	>99%
5	322 to 327	TEDD.AG	35.2 kD	128.0 kD	0.212	60.714	TEVDAA	12.888	>99%
6	256 to 261	EIVD.LV	28.1 kD	135.0 kD	0.145	70.370	ELVDSV	10.207	>99%
7	1250 to 1255	ESED.SM	137.4 kD	25.7 kD	0.143	65.517	EQEDSS	9.350	>99%
8	142 to 147	ATVD.NL	15.8 kD	147.3 kD	0.117	75.862	ATIDGL	8.876	>99%
9	1129 to 1134	SPLD.SN	123.8 kD	39.4 kD	0.079	75.000	SLLDSN	5.935	>99%
10	298 to 303	EVLD.TE	32.7 kD	130.4 kD	0.076	62.069	DVVDAE	4.745	>99%
11	69 to 74	EPVD.TL	7.7 kD	155.5 kD	0.064	58.621	ETVDTS	3.723	>95%
12	728 to 733	EGLD.RG	79.5 kD	83.6 kD	0.054	62.500	DGVDAG	3.349	>95%
13	1323 to 1328	SLSD.RA	145.4 kD	17.7 kD	0.061	53.571	SLFDSA	3.271	>95%
14	1229 to 1234	TPVD.NS	134.9 kD	28.3 kD	0.050	58.333	QPVDNH	2.906	>95%
15	1053 to 1058	LTLD.TP	115.7 kD	47.4 kD	0.035	68.966	VSLDSP	2.421	>95%
16	1086 to 1091	DSSD.KM	119.4 kD	43.7 kD	0.035	64.516	DEVDKM	2.261	>95%
17	511 to 516	EGVD.RE	55.5 kD	107.6 kD	0.035	60.714	ESVDKS	2.142	>95%
18	1215 to 1220	VMTD.TP	133.4 kD	29.8 kD	0.035	55.172	VSLDSP	1.904	>95%
19	439 to 444	LEHD.VA	47.9 kD	115.2 kD	0.037	50.000	MEVDAA	1.870	>95%
20	1460 to 1465	MLED.SE	159.8 kD	3.3 kD	0.033	54.839	AMEDGE	1.807	>95%

>LA_M
OUSE_

rank	position	site	N fragment	C fragment	frequency score	similarity maxscore	similarity maxsite	average score	specificity
1	99 to 104	EVTD.EY	11.8 kD	35.9 kD	0.046	53.333	EVTDAG	2.447	>95%
2	237 to 242	GDLD.DQ	27.8 kD	20.0 kD	0.027	58.065	ADIDGQ	1.582	>95%
3	156 to 161	AVFD.SI	18.5 kD	29.2 kD	0.025	60.714	SLFDSA	1.518	>95%
4	380 to 385	DDDD.RR	43.9 kD	3.8 kD	0.025	58.065	DEDDSA	1.460	>95%
5	394 to 399	DGRD.RE	45.6 kD	2.1 kD	0.022	51.724	DSSDSE	1.155	>95%
6	129 to 134	EWLD.DK	15.4 kD	32.4 kD	0.019	57.500	DWFDGK	1.105	>95%
7	61 to 66	LTTD.FN	7.6 kD	40.1 kD	0.016	62.069	LSSDFT	1.003	>95%
8	238 to 243	DLDD.QT	27.9 kD	19.8 kD	0.012	60.606	DLNDGT	0.706	>95%
9	122 to 127	ATLD.DI	14.5 kD	33.3 kD	0.011	62.069	ATIDGL	0.676	>95%
10	123 to 128	TLDD.IK	14.6 kD	33.1 kD	0.006	53.571	ALDDLI	0.316	>95%
11	118 to 123	FPTD.AT	14.1 kD	33.7 kD	0.005	56.250	FPADEA	0.295	>95%
12	82 to 87	VSAD.KT	9.9 kD	37.9 kD	0.004	51.724	VETDKA	0.232	>95%
13	40 to 45	IKLD.EG	5.1 kD	42.6 kD	0.004	58.621	VEVDSG	0.213	>95%
14	259 to 264	KWVD.FA	30.5 kD	17.3 kD	0.004	43.333	DSVDFS	0.158	>95%
15	3 to 8	ENGD.NE	0.6 kD	47.1 kD	0.003	53.333	ESLDNQ	0.151	>95%
16	316 to 321	KITD.DQ	36.9 kD	10.9 kD	0.003	53.333	DVTDAQ	0.139	>95%
17	391 to 396	RGRD.GR	45.3 kD	2.4 kD	0.002	48.485	EGEDDR	0.120	>95%
18	320 to 325	DQQE.SL	37.4 kD	10.4 kD	0.001	66.667	DEQDSV	0.095	>95%
19	159 to 164	DSIQ.SA	18.9 kD	28.9 kD	0.001	57.692	SSVDSA	0.076	>95%
20	377 to 382	TRFD.DD	43.6 kD	4.2 kD	0.001	46.667	TQFDAA	0.056	>95%

>TARA
_MOU
SE_

rank	position	site	N fragment	C fragment	frequency score	similarity maxscore	similarity maxsite	average score	specificity
1	1480 to 1485	DELD.GE	162.2 kD	61.1 kD	12.720	90.625	DEVDGE	1152.764	>99.9%
2	1462 to 1467	VLTD.SS	160.1 kD	63.2 kD	0.953	82.143	TLTDSS	78.269	>99.9%
3	1641 to 1646	ESTD.GR	180.4 kD	43.0 kD	1.031	73.333	ESVDGQ	75.572	>99.9%
4	758 to 763	DTSD.AD	82.9 kD	140.4 kD	0.305	78.125	DTTDSD	23.791	>99%
5	363 to 368	SQRD.SS	39.2 kD	184.2 kD	0.295	70.370	SESDSS	20.749	>99%
6	1903 to 1908	EEID.RL	210.7 kD	12.6 kD	0.235	78.571	SELDRL	18.451	>99%
7	1477 to 1482	EEAD.EL	161.9 kD	61.5 kD	0.144	75.862	EEADSM	10.899	>99%
8	130 to 135	DDND.NK	14.0 kD	209.4 kD	0.135	78.788	DDNDSE	10.627	>99%
9	587 to 592	TQSD.SP	64.3 kD	159.0 kD	0.137	62.069	TDSDSS	8.528	>99%
10	866 to 871	PSID.SL	94.6 kD	128.7 kD	0.100	76.923	SSVDSL	7.699	>99%
11	1625 to 1630	LERD.LA	178.4 kD	45.0 kD	0.118	56.250	VHRDMA	6.623	>99%
12	459 to 464	TQRD.SP	50.1 kD	173.2 kD	0.111	56.667	TEKDSM	6.303	>99%
13	147 to 152	SSQD.SN	15.8 kD	207.5 kD	0.099	59.375	DTQDAN	5.904	>99%
14	814 to 819	PSTD.SM	89.1 kD	134.3 kD	0.072	62.963	SSTDSA	4.555	>99%
15	1350 to 1355	EQID.HL	147.4 kD	76.0 kD	0.041	75.000	EEIDHA	3.106	>95%
16	1389 to 1394	DSAD.KR	151.7 kD	71.7 kD	0.053	58.621	DSSDSE	3.088	>95%
17	192 to 197	LRAD.SA	20.9 kD	202.5 kD	0.050	60.714	LKTDSS	3.051	>95%
18	1651 to 1656	PSGD.GS	181.4 kD	42.0 kD	0.042	68.750	DSGDGA	2.901	>95%
19	1484 to 1489	GEID.LR	162.6 kD	60.7 kD	0.048	53.333	EEVDLN	2.581	>95%
20	760 to 765	SDAD.NE	83.1 kD	140.3 kD	0.039	60.606	TDMDNQ	2.341	>95%

>LMN
B1_MO
USE_

rank	position	site	N fragment	C fragment	frequency score	similarity maxscore	similarity maxsite	average score	specificity
1	229 to 234	VEVD.SG	26.2 kD	40.5 kD	4.838	100.000	VEVDSG	483.795	>99.9%
2	448 to 453	IDVD.GK	51.5 kD	15.2 kD	0.363	73.333	VEVDGQ	26.593	>99%
3	163 to 168	DLED.LK	18.5 kD	48.3 kD	0.277	66.667	DLPDMK	18.437	>99%
4	86 to 91	ELAD.AR	10.0 kD	56.8 kD	0.139	58.621	DMADAA	8.158	>99%
5	446 to 451	EEID.VD	51.3 kD	15.5 kD	0.113	71.875	EEIDGD	8.102	>99%
6	252 to 257	EQHD.AQ	29.1 kD	37.7 kD	0.049	68.966	EQEDAS	3.349	>95%
7	125 to 130	KESD.LS	14.5 kD	52.3 kD	0.020	100.000	KESDLS	1.969	>95%
8	144 to 149	NSKD.AA	16.6 kD	50.2 kD	0.029	56.250	NSPDAQ	1.649	>95%
9	331 to 336	KERD.NS	38.3 kD	28.4 kD	0.019	67.742	EERDGS	1.296	>95%
10	461 to 466	SEQD.QP	53.0 kD	13.7 kD	0.021	54.839	EEADSP	1.146	>95%
11	324 to 329	ELED.ML	37.5 kD	29.3 kD	0.021	53.571	ALDDLI	1.122	>95%
12	93 to 98	ALDD.TA	10.8 kD	56.0 kD	0.017	64.286	ALDDLI	1.065	>95%
13	39 to 44	ELND.RL	4.6 kD	62.2 kD	0.016	62.500	DLNDGL	1.027	>95%
14	160 to 165	LEGD.LE	18.1 kD	48.6 kD	0.015	57.143	IEADSE	0.878	>95%
15	363 to 368	QLLD.VK	42.3 kD	24.5 kD	0.015	46.875	GLLDPK	0.692	>95%
16	191 to 196	LKVD.LE	21.5 kD	45.3 kD	0.015	46.429	LKTDSS	0.675	>95%
17	166 to 171	DLKD.QI	18.8 kD	47.9 kD	0.008	64.706	DLKDHM	0.504	>95%
18	113 to 118	AEHD.QL	13.1 kD	53.7 kD	0.009	50.000	SELDRL	0.429	>95%
19	369 to 374	LALD.ME	42.9 kD	23.8 kD	0.006	48.387	TELDMD	0.294	>95%
20	524 to 529	TGED.VK	59.9 kD	6.8 kD	0.006	50.000	TAVDAK	0.285	>95%

>FA20
C_MO
USE_

rank	position	site	N fragment	C fragment	frequency score	similarity maxscore	similarity maxsite	average score	specificity
1	556 to 561	VEKD.GL	63.6 kD	2.2 kD	0.292	60.000	TEKDSM	17.522	>99%
2	110 to 115	EPVD.HA	12.0 kD	53.7 kD	0.052	78.125	EEIDHA	4.024	>99%
3	565 to 570	VEDD.LA	64.5 kD	1.3 kD	0.072	55.556	VEVDAA	3.984	>99%
4	149 to 154	PSGD.GS	16.2 kD	49.5 kD	0.042	68.750	DSGDGA	2.901	>95%
5	239 to 244	PAID.AL	26.3 kD	39.4 kD	0.032	75.862	PAADAI	2.446	>95%
6	25 to 30	IAVD.LL	3.2 kD	62.5 kD	0.040	55.556	MAVDAV	2.245	>95%
7	173 to 178	TEDD.VL	18.8 kD	46.9 kD	0.031	63.333	DEEDIL	1.985	>95%
8	539 to 544	EALD.RR	61.5 kD	4.2 kD	0.031	56.667	ESLDNQ	1.753	>95%
9	386 to 391	FLPD.LS	43.3 kD	22.5 kD	0.023	58.621	IVPDIA	1.362	>95%
10	244 to 249	LLRD.LG	26.9 kD	38.8 kD	0.025	46.429	VLVDAG	1.180	>95%
11	71 to 76	AGGD.AG	7.8 kD	57.9 kD	0.018	60.714	AAVDAG	1.074	>95%
12	564 to 569	VVED.DL	64.4 kD	1.4 kD	0.017	51.724	VVPDAL	0.862	>95%
13	470 to 475	IHLD.NG	53.6 kD	12.2 kD	0.013	63.333	LVIDNG	0.829	>95%
14	443 to 448	TVFD.FL	50.3 kD	15.5 kD	0.014	48.276	TVADGL	0.678	>95%
15	438 to 443	DIMD.MT	49.7 kD	16.1 kD	0.010	67.742	DLFDLT	0.655	>95%
16	134 to 139	LLRD.PG	14.8 kD	51.0 kD	0.008	67.742	VMRDPA	0.517	>95%
17	377 to 382	DQIE.GS	42.4 kD	23.4 kD	0.008	61.290	DEADGS	0.472	>95%
18	551 to 556	AVRD.CV	63.0 kD	2.7 kD	0.008	54.545	KLTDCV	0.430	>95%
19	334 to 339	EIRD.VT	37.4 kD	28.4 kD	0.005	53.571	ESQDVS	0.289	>95%
20	172 to 177	LTED.DV	18.7 kD	47.0 kD	0.007	39.394	DEDDDV	0.286	>95%

>SEM4
D_MO
USE_

rank	position	site	N fragment	C fragment	frequency score	similarity maxscore	similarity maxsite	average score	specificity
1	282 to 287	SKPD.SG	32.0 kD	63.6 kD	0.283	75.758	SQPDTG	21.441	>99%
2	856 to 861	SDAD.GD	95.5 kD	0.2 kD	0.243	67.742	AEVDGD	16.465	>99%
3	828 to 833	DRED.SQ	92.3 kD	3.4 kD	0.178	70.968	DEEDSK	12.605	>99%
4	615 to 620	SDGD.SG	68.9 kD	26.7 kD	0.194	59.259	SESDSS	11.472	>99%
5	219 to 224	KSPD.GP	24.7 kD	70.9 kD	0.082	69.444	ADPDGP	5.668	>99%
6	657 to 662	TSED.AQ	73.6 kD	22.0 kD	0.073	61.290	TSHDAS	4.490	>99%
7	429 to 434	QALD.GT	48.2 kD	47.4 kD	0.051	63.333	SALDGD	3.228	>95%
8	780 to 785	DFSD.LE	87.0 kD	8.7 kD	0.048	59.375	DFTDSQ	2.839	>95%
9	613 to 618	NLSD.GD	68.7 kD	26.9 kD	0.043	56.250	DISDGG	2.397	>95%
10	441 to 446	ISTD.RG	49.6 kD	46.0 kD	0.027	79.310	LSVDRG	2.148	>95%
11	854 to 859	ADSD.AD	95.3 kD	0.4 kD	0.036	58.621	TDSDSS	2.082	>95%
12	369 to 374	ACID.SE	41.5 kD	54.2 kD	0.026	78.788	ECVDSE	2.038	>95%
13	148 to 153	KSED.GK	16.9 kD	78.7 kD	0.027	71.875	EEEDGK	1.943	>95%
14	225 to 230	EGED.DK	25.3 kD	70.3 kD	0.015	90.909	EGEDDR	1.370	>95%
15	808 to 813	PALD.TG	90.0 kD	5.6 kD	0.019	67.742	DAVDTG	1.283	>95%
16	57 to 62	EDKD.TL	6.7 kD	88.9 kD	0.022	53.333	DDLDAL	1.192	>95%
17	552 to 557	SCLD.KS	61.7 kD	33.9 kD	0.016	68.750	SCTDSS	1.132	>95%
18	816 to 821	TEQD.TI	90.9 kD	4.7 kD	0.017	63.333	TEKDSM	1.075	>95%
19	399 to 404	LMDD.SV	44.8 kD	50.8 kD	0.017	50.000	ALDDLI	0.840	>95%
20	466 to 471	LFRD.SE	52.5 kD	43.1 kD	0.015	46.429	IEADSE	0.699	>95%

>CATB
_MOU
SE_

rank	position	site	N fragment	C fragment	frequency score	similarity maxscore	similarity maxsite	average score	specificity
1	76 to 81	EDID.LP	8.9 kD	28.4 kD	0.105	78.788	DDVDIP	8.234	>99%
2	82 to 87	ETFD.AR	9.6 kD	27.7 kD	0.038	60.000	DTYDGR	2.277	>95%
3	134 to 139	SAED.LL	15.2 kD	22.1 kD	0.037	53.333	DEEDIL	1.994	>95%
4	303 to 308	DWGD.NG	33.5 kD	3.8 kD	0.028	56.250	DAGDVG	1.566	>95%
5	200 to 205	GEGD.TP	22.0 kD	15.3 kD	0.026	56.250	GQVDVP	1.488	>95%
6	255 to 260	VFSD.FL	28.1 kD	9.2 kD	0.007	48.276	LSSDFT	0.344	>95%
7	25 to 30	PLSD.DL	3.2 kD	34.1 kD	0.007	48.148	SLLDEL	0.340	>95%
8	48 to 53	YNVD.IS	5.9 kD	31.3 kD	0.005	56.250	HNLDAS	0.283	>95%
9	16 to 21	SAHD.KP	2.2 kD	35.1 kD	0.004	55.556	ATHDGP	0.238	>95%
10	26 to 31	LSDD.LI	3.3 kD	34.0 kD	0.003	60.714	ALDDLI	0.198	>95%
11	270 to 275	EAGD.MM	29.8 kD	7.5 kD	0.002	51.613	DMGDLV	0.109	>95%
12	116 to 121	AISD.RT	13.2 kD	24.1 kD	0.002	43.333	FIQDRA	0.083	>95%

>NOSIP_MOUSE_

rank	position	site	N fragment	C fragment	frequency score	similarity maxscore	similarity maxsite	average score	specificity
1	207 to 212	DSVD.RV	23.4 kD	9.8 kD	1.111	71.429	DSLDSV	79.379	>99.9%
2	258 to 263	DMVD.PV	29.0 kD	4.2 kD	0.204	62.963	ELVDSV	12.852	>99%
3	58 to 63	VTPD.GY	6.7 kD	26.5 kD	0.150	57.576	ITLDGH	8.656	>99%
4	225 to 230	VTRD.SL	25.5 kD	7.7 kD	0.090	55.172	TLRDSM	4.958	>99%
5	203 to 208	TQLD.DS	23.0 kD	10.2 kD	0.078	59.375	DQIDDT	4.609	>99%
6	35 to 40	LSRD.AV	4.2 kD	29.0 kD	0.068	51.852	MAVDAV	3.535	>95%
7	204 to 209	QLDD.SV	23.1 kD	10.1 kD	0.017	62.963	ELVDSV	1.095	>95%
8	269 to 274	TERD.II	30.2 kD	3.0 kD	0.014	56.667	TEKDSM	0.818	>95%
9	263 to 268	VNGD.TL	29.5 kD	3.7 kD	0.002	45.161	DEGDSL	0.096	>95%
10	39 to 44	AVKD.FD	4.6 kD	28.6 kD	0.002	37.500	DITDFQ	0.083	>95%
11	106 to 111	AAQD.QV	12.5 kD	20.7 kD	0.001	42.857	VASDGV	0.061	>95%

>A2AG H5_M OUSE_

rank	position	site	N fragment	C fragment	frequency score	similarity maxscore	similarity maxsite	average score	specificity
1	361 to 366	ELVD.SL	42.4 kD	9.5 kD	3.053	88.889	ELVDSV	271.379	>99.9%
2	425 to 430	SDED.GP	49.3 kD	2.6 kD	0.344	71.429	GDSDGP	24.594	>99%
3	255 to 260	LQPD.YS	30.2 kD	21.7 kD	0.044	61.290	MEVDYS	2.712	>95%
4	299 to 304	DLCD.ES	35.5 kD	16.4 kD	0.029	71.429	DFCDSS	2.055	>95%
5	146 to 151	PSVD.EL	17.8 kD	34.1 kD	0.025	65.385	SSVDSL	1.644	>95%
6	315 to 320	TPGD.SA	37.2 kD	14.7 kD	0.023	51.724	TDADAA	1.180	>95%
7	296 to 301	DTID.LC	35.2 kD	16.7 kD	0.016	71.429	DSVDLA	1.134	>95%
8	229 to 234	NLED.LV	27.4 kD	24.4 kD	0.009	60.714	ALDDLI	0.570	>95%
9	126 to 131	SNRD.RS	15.4 kD	36.5 kD	0.006	46.875	SHTDQS	0.283	>95%
10	440 to 445	TRAD.FL	51.0 kD	0.8 kD	0.006	44.828	TVADGL	0.252	>95%
11	368 to 373	TQCD.FL	43.2 kD	8.7 kD	0.003	46.875	TEVDFN	0.126	>95%
12	26 to 31	KVSD.NN	3.5 kD	48.4 kD	0.002	61.290	DVSDNE	0.116	>95%

>NICA
_MOU
SE_

rank	position	site	N fragment	C fragment	frequency score	similarity maxscore	similarity maxsite	average score	specificity
1	84 to 89	VLTD.GP	9.1 kD	69.3 kD	0.849	72.727	EITDGP	61.757	>99.9%
2	279 to 284	TRLD.SR	30.7 kD	47.8 kD	0.045	59.259	SSLDAR	2.652	>95%
3	268 to 273	LEPD.VR	29.5 kD	49.0 kD	0.048	51.724	IVPDIA	2.503	>95%
4	356 to 361	ENID.SF	39.2 kD	39.3 kD	0.026	76.667	DSIDSF	2.010	>95%
5	478 to 483	FVTD.TA	53.0 kD	25.5 kD	0.034	56.667	FIQDRA	1.941	>95%
6	543 to 548	SYLD.DR	60.1 kD	18.4 kD	0.029	61.290	SYLDSG	1.747	>95%
7	454 to 459	SIYD.TA	50.2 kD	28.3 kD	0.023	71.429	SLFDSA	1.656	>95%
8	8 to 13	SGPD.PG	1.0 kD	77.4 kD	0.022	60.606	SQPDTG	1.308	>95%
9	439 to 444	VLAD.HS	48.3 kD	30.2 kD	0.024	46.429	TLTDSS	1.110	>95%
10	77 to 82	KEED.LK	8.3 kD	70.2 kD	0.011	71.429	KESDLS	0.773	>95%
11	594 to 599	ESKD.LY	65.7 kD	12.7 kD	0.014	53.571	ESQDVS	0.762	>95%
12	472 to 477	PEED.LN	52.3 kD	26.2 kD	0.011	61.290	DEEDLQ	0.663	>95%
13	371 to 376	TSLD.LW	40.8 kD	37.6 kD	0.009	61.111	TFSDLW	0.573	>95%
14	171 to 176	AYED.FS	18.5 kD	59.9 kD	0.009	60.000	DYEDFM	0.544	>95%
15	332 to 337	ETFD.YI	36.4 kD	42.1 kD	0.007	50.000	DTYDAL	0.358	>95%
16	392 to 397	QVED.LL	43.3 kD	35.1 kD	0.006	53.333	GVEDTL	0.323	>95%
17	65 to 70	ISGD.TG	6.9 kD	71.5 kD	0.006	50.000	DAGDVG	0.278	>95%
18	691 to 696	AKAD.VL	77.0 kD	1.5 kD	0.005	44.828	NEADAL	0.225	>95%
19	249 to 254	PLSD.YN	27.3 kD	51.2 kD	0.003	51.515	DLADYG	0.174	>95%
20	181 to 186	LLED.EN	19.8 kD	58.7 kD	0.004	39.286	SLLDSN	0.143	>95%

>RUXE_MOUSE_

rank	position	site	N fragment	C fragment	frequency score	similarity maxscore	similarity maxsite	average score	specificity
1	56 to 61	LVLD.DA	7.1 kD	3.7 kD	0.080	56.667	LVIDNG	4.525	>99%
2	57 to 62	VLDD.AE	7.2 kD	3.6 kD	0.018	51.724	VLKDTQ	0.938	>95%

>CSF1
R_MO
USE_

rank	position	site	N fragment	C fragment	frequency score	similarity maxscore	similarity maxsite	average score	specificity
1	691 to 696	SEGD.SS	77.6 kD	31.6 kD	0.797	85.185	SESDSS	67.862	>99.9%
2	269 to 274	DFQD.AG	30.1 kD	79.1 kD	0.231	61.765	DFQDGR	14.285	>99%
3	931 to 936	SGSD.SG	104.8 kD	4.4 kD	0.178	60.000	SGVDIG	10.698	>99%
4	314 to 319	SVGD.SL	34.8 kD	74.3 kD	0.151	62.963	SVTDSV	9.532	>99%
5	730 to 735	SSSD.SF	82.0 kD	27.2 kD	0.099	62.963	SSTDSA	6.228	>99%
6	133 to 138	ALKD.SV	14.6 kD	94.5 kD	0.048	58.621	TLRDSM	2.824	>95%
7	168 to 173	KVLD.SN	18.7 kD	90.4 kD	0.029	75.000	SLLDSN	2.164	>95%
8	251 to 256	DFQD.NY	27.9 kD	81.2 kD	0.030	66.667	DEQDSY	2.024	>95%
9	707 to 712	VRRD.SG	79.6 kD	29.6 kD	0.031	61.290	EERDSG	1.892	>95%
10	866 to 871	LVKD.GY	97.4 kD	11.8 kD	0.024	60.000	LISDTY	1.423	>95%
11	266 to 271	NAVD.FQ	29.7 kD	79.5 kD	0.021	60.000	DSVDFS	1.261	>95%
12	323 to 328	VHAD.AY	35.8 kD	73.4 kD	0.018	57.143	VSVDAF	1.021	>95%
13	128 to 133	LITD.PA	14.1 kD	95.1 kD	0.017	58.065	VMRDPA	0.966	>95%
14	506 to 511	QLPD.ES	57.0 kD	52.2 kD	0.011	62.069	SVPDSS	0.692	>95%
15	603 to 608	GKED.AV	68.1 kD	41.0 kD	0.012	55.556	SESDAV	0.682	>95%
16	280 to 285	ASND.VG	31.1 kD	78.0 kD	0.009	50.000	AAVDAG	0.447	>95%
17	824 to 829	SIFD.CV	92.5 kD	16.7 kD	0.007	66.667	DLYDCV	0.435	>95%
18	437 to 442	DRCD.EA	49.2 kD	60.0 kD	0.006	66.667	DRLDRA	0.398	>95%
19	716 to 721	QGVD.TY	80.5 kD	28.7 kD	0.008	50.000	SGVDAA	0.385	>95%
20	86 to 91	ELED.PM	9.6 kD	99.6 kD	0.007	48.148	ELVDSV	0.314	>95%

>TNR1
B_MO
USE_

rank	position	site	N fragment	C fragment	frequency score	similarity maxscore	similarity maxsite	average score	specificity
1	196 to 201	ASTD.AV	21.6 kD	28.7 kD	0.354	64.286	SSTDAK	22.761	>99%
2	408 to 413	GDPD.AK	43.5 kD	6.8 kD	0.141	63.889	DDPDGK	9.002	>99%
3	391 to 396	SSSD.HS	41.9 kD	8.4 kD	0.047	62.963	SESDSS	2.955	>95%
4	295 to 300	LQRD.AK	32.0 kD	18.3 kD	0.041	46.875	AQRDSH	1.942	>95%
5	452 to 457	GVPD.MG	48.3 kD	2.0 kD	0.016	55.172	SVPDSS	0.868	>95%
6	176 to 181	SSTD.VC	19.5 kD	30.8 kD	0.008	62.963	SSTDSA	0.529	>95%
7	368 to 373	RISD.SS	39.6 kD	10.7 kD	0.008	58.621	DITDSS	0.460	>95%
8	45 to 50	EYYD.RK	5.4 kD	44.9 kD	0.010	46.154	DYYDDY	0.449	>95%
9	308 to 313	KSQD.AV	33.4 kD	16.9 kD	0.007	51.852	SESDAV	0.367	>95%
10	101 to 106	CTTD.QV	11.7 kD	38.6 kD	0.005	54.286	CSTDSP	0.259	>95%
11	330 to 335	SSLE.SS	35.7 kD	14.6 kD	0.004	61.538	SSVDSA	0.222	>95%
12	170 to 175	TFSD.TT	18.9 kD	31.4 kD	0.004	50.000	TFSDLW	0.205	>95%
13	337 to 342	SAGD.RR	36.2 kD	14.1 kD	0.004	50.000	SATDRG	0.187	>95%
14	303 to 308	HVPD.EK	32.9 kD	17.5 kD	0.003	55.882	DVPDGK	0.168	>95%
15	75 to 80	VCAD.CE	8.7 kD	41.6 kD	0.002	66.667	TCADCG	0.129	>95%
16	70 to 75	KTSD.TV	8.2 kD	42.1 kD	0.002	48.387	ETTDGV	0.092	>95%
17	406 to 411	TVGD.PD	43.3 kD	7.0 kD	0.002	44.118	DAGDGD	0.074	>95%

>LEG9
_MOU
SE_

rank	position	site	N fragment	C fragment	frequency score	similarity maxscore	similarity maxsite	average score	specificity
1	245 to 250	VLPD.AT	27.9 kD	12.1 kD	0.107	72.414	VVPDAL	7.752	>99%
2	128 to 133	HLVD.TI	15.1 kD	24.9 kD	0.007	66.667	ELVDSV	0.489	>95%
3	342 to 347	VAGD.IQ	39.1 kD	0.9 kD	0.002	43.750	DAGDVG	0.103	>95%
4	258 to 263	CGGD.IA	29.4 kD	10.6 kD	0.002	42.857	CFADVG	0.065	>95%
5	333 to 338	NLQD.IN	38.2 kD	1.8 kD	0.001	50.000	DTQDAN	0.064	>95%
6	53 to 58	NGND.IA	6.1 kD	33.9 kD	0.001	46.667	SGVDIG	0.062	>95%

>PROP_MOUSE_

rank	position	site	N fragment	C fragment	frequency score	similarity maxscore	similarity maxsite	average score	specificity
1	34 to 39	STVD.RS	4.0 kD	21.0 kD	0.153	62.069	ETVDTS	9.513	>99%
2	99 to 104	LAQD.TG	11.6 kD	13.4 kD	0.008	48.387	DAVDTG	0.400	>95%
3	79 to 84	EQLE.SA	9.4 kD	15.7 kD	0.003	75.000	EELDSA	0.240	>95%

>LGM
N_MO
USE_

rank	position	site	N fragment	C fragment	frequency score	similarity maxscore	similarity maxsite	average score	specificity
1	24 to 29	DPED.GG	2.8 kD	46.6 kD	0.397	82.353	DEEDGG	32.704	>99%
2	244 to 249	DVED.LT	27.7 kD	21.7 kD	0.098	61.290	DLFDLT	6.032	>99%
3	20 to 25	VGVD.DP	2.3 kD	47.0 kD	0.046	62.069	VSLDSP	2.847	>95%
4	308 to 313	PSPD.VP	34.9 kD	14.5 kD	0.013	80.000	PAPDAP	1.038	>95%
5	241 to 246	EDSD.VE	27.3 kD	22.0 kD	0.018	56.667	DDSDAA	1.013	>95%
6	302 to 307	THLD.LT	34.3 kD	15.1 kD	0.013	59.375	DHVDLS	0.772	>95%
7	339 to 344	QFLD.AR	38.5 kD	10.9 kD	0.011	62.963	SSLDAR	0.706	>95%
8	62 to 67	GIPD.EQ	7.0 kD	42.3 kD	0.004	55.172	SVPDSS	0.243	>95%
9	146 to 151	YFTD.HG	16.2 kD	33.1 kD	0.006	43.333	SATDRG	0.240	>95%
10	93 to 98	NGTD.VY	10.5 kD	38.9 kD	0.005	45.455	SMTDFY	0.219	>95%
11	101 to 106	VLKD.YT	11.4 kD	38.0 kD	0.004	55.172	VLKDTQ	0.204	>95%
12	119 to 124	LRGD.AE	13.3 kD	36.0 kD	0.003	60.606	MQGDGE	0.152	>95%
13	376 to 381	TAHD.CY	42.6 kD	6.7 kD	0.002	58.065	TSHDAS	0.138	>95%
14	199 to 204	HLPD.DI	22.4 kD	26.9 kD	0.002	50.000	TLPDGL	0.121	>95%
15	72 to 77	MYDD.IA	8.3 kD	41.1 kD	0.002	53.125	DYKDIA	0.110	>95%
16	106 to 111	TGED.VT	11.9 kD	37.4 kD	0.003	42.857	ESQDVS	0.109	>95%
17	425 to 430	MAMD.KV	48.5 kD	0.8 kD	0.001	70.370	MAVDAV	0.095	>95%
18	305 to 310	DLTP.SP	34.6 kD	14.8 kD	0.001	59.375	DSTDSP	0.088	>95%
19	23 to 28	DDPE.DG	2.6 kD	46.7 kD	0.001	51.429	DDEDDD	0.072	>95%
20	239 to 244	WMED.SD	27.1 kD	22.2 kD	0.001	50.000	DMTDSD	0.051	>95%

>SRP1 4_MO USE_

rank	position	site	N fragment	C fragment	frequency score	similarity maxscore	similarity maxsite	average score	specificity
1	59 to 64	RATD.GK	7.0 kD	5.4 kD	0.046	75.000	DATDGK	3.462	>95%
2	89 to 94	ANMD.GL	10.4 kD	2.1 kD	0.022	72.414	ATIDGL	1.604	>95%
3	44 to 49	SSVE.GL	5.4 kD	7.1 kD	0.007	69.231	SSVDSL	0.499	>95%
4	31 to 36	KKYD.GR	4.0 kD	8.5 kD	0.002	62.857	DTYDGR	0.119	>95%

>ATPB_MOUSE_

rank	position	site	N fragment	C fragment	frequency score	similarity maxscore	similarity maxsite	average score	specificity
1	366 to 371	DLTD.PA	38.7 kD	17.6 kD	0.741	82.759	DLTDAA	61.296	>99.9%
2	399 to 404	DPLD.ST	42.0 kD	14.2 kD	0.381	80.000	DTLDST	30.448	>99%
3	124 to 129	KVLD.SG	12.9 kD	43.4 kD	0.080	66.667	EVMDAG	5.366	>99%
4	447 to 452	SEED.KL	47.4 kD	8.8 kD	0.051	64.286	SELDRL	3.294	>95%
5	69 to 74	AVVD.VQ	6.9 kD	49.4 kD	0.049	55.172	SVIDGQ	2.720	>95%
6	377 to 382	AHLD.AT	39.8 kD	16.5 kD	0.033	59.259	SELDAS	1.942	>95%
7	335 to 340	LATD.MG	35.3 kD	21.0 kD	0.022	60.000	SATDRG	1.305	>95%
8	188 to 193	KVVD.LL	19.7 kD	36.5 kD	0.022	53.571	DVLDVL	1.203	>95%
9	497 to 502	GEYD.HL	53.1 kD	3.1 kD	0.022	50.000	GSYDSY	1.102	>95%
10	111 to 116	IAMD.GT	11.5 kD	44.8 kD	0.012	79.310	IVLDGT	0.943	>95%
11	416 to 421	EHYD.VA	44.0 kD	12.3 kD	0.014	53.125	DHVDLS	0.753	>95%
12	114 to 119	DGTE.GL	11.8 kD	44.5 kD	0.007	87.879	DGTDGL	0.653	>95%
13	396 to 401	PAVD.PL	41.7 kD	14.6 kD	0.010	62.069	PAADAI	0.645	>95%
14	297 to 302	EGQD.VL	31.2 kD	25.0 kD	0.008	64.286	ESQDVS	0.540	>95%
15	42 to 47	PARD.YA	4.4 kD	51.9 kD	0.006	64.516	SQRDYA	0.416	>95%
16	73 to 78	VQFD.EG	7.4 kD	48.9 kD	0.007	58.621	VEVDSG	0.389	>95%
17	89 to 94	QGRD.SR	9.1 kD	47.2 kD	0.006	57.576	EGEDDR	0.333	>95%
18	257 to 262	NLKD.AT	26.9 kD	29.3 kD	0.006	48.485	DLNDGT	0.297	>95%
19	433 to 438	SLQD.II	45.9 kD	10.4 kD	0.005	60.714	ALDDLI	0.283	>95%
20	362 to 367	VPAD.DL	38.2 kD	18.1 kD	0.004	53.125	FPADEA	0.232	>95%

>IL6R
B_MO
USE_

rank	position	site	N fragment	C fragment	frequency score	similarity maxscore	similarity maxsite	average score	specificity
1	798 to 803	DSVD.GG	89.4 kD	13.1 kD	24.037	70.968	DSTDGA	1705.880	>99.9%
2	566 to 571	VHVD.SS	63.8 kD	38.6 kD	0.414	62.069	VEVDSG	25.726	>99%
3	795 to 800	QLVD.SV	89.1 kD	13.4 kD	0.274	88.889	ELVDSV	24.342	>99%
4	871 to 876	STAD.AL	97.7 kD	4.7 kD	0.175	62.069	NEADAL	10.885	>99%
5	213 to 218	DPVD.KV	24.3 kD	78.1 kD	0.068	67.742	DEVDKM	4.609	>99%
6	878 to 883	TGAD.GQ	98.3 kD	4.1 kD	0.075	56.667	TSTDGS	4.254	>99%
7	249 to 254	GLLD.LK	28.1 kD	74.3 kD	0.061	68.750	GLLDPK	4.227	>99%
8	783 to 788	PLLD.SE	87.7 kD	14.8 kD	0.069	60.714	SLLDSN	4.173	>99%
9	299 to 304	SIKD.SG	34.1 kD	68.4 kD	0.059	55.172	SVPDSS	3.279	>95%
10	680 to 685	MYSD.GN	76.6 kD	25.8 kD	0.024	62.500	LQTDGN	1.484	>95%
11	580 to 585	LSSD.TL	65.3 kD	37.1 kD	0.027	53.333	LISDTY	1.425	>95%
12	592 to 597	AYTD.EG	66.7 kD	35.7 kD	0.023	61.290	SYLDSG	1.397	>95%
13	839 to 844	NEED.FV	94.0 kD	8.4 kD	0.022	60.000	DEEDIL	1.327	>95%
14	597 to 602	GGKD.GP	67.2 kD	35.2 kD	0.017	68.571	GDSDGP	1.199	>95%
15	90 to 95	TFTD.VV	10.5 kD	91.9 kD	0.022	53.125	DYTDAV	1.188	>95%
16	283 to 288	TVQD.LK	32.1 kD	70.4 kD	0.013	59.259	TIADLA	0.773	>95%
17	652 to 657	NVPD.PS	73.4 kD	29.1 kD	0.010	72.414	SVPDSS	0.723	>95%
18	685 to 690	NFTD.VS	77.2 kD	25.2 kD	0.012	53.333	DKTDIS	0.625	>95%
19	469 to 474	QQED.AT	53.2 kD	49.3 kD	0.007	79.310	EQEDAS	0.519	>95%
20	801 to 806	DGGD.EI	89.6 kD	12.8 kD	0.007	58.065	DEGDSL	0.397	>95%

>ATPA_MOUSE_

rank	position	site	N fragment	C fragment	frequency score	similarity maxscore	similarity maxsite	average score	specificity
1	451 to 456	SDLD.AA	48.8 kD	10.9 kD	1.126	77.778	SELDAS	87.582	>99.9%
2	387 to 392	SITD.GQ	41.9 kD	17.9 kD	0.421	79.310	SVIDGQ	33.395	>99%
3	109 to 114	LEPD.NV	11.8 kD	47.9 kD	0.211	61.290	EEPDSI	12.910	>99%
4	534 to 539	EQSD.AK	58.0 kD	1.8 kD	0.166	68.966	EQEDAS	11.421	>99%
5	234 to 239	DGTD.EK	25.1 kD	34.7 kD	0.175	64.516	DETDSK	11.306	>99%
6	156 to 161	NAID.GK	16.7 kD	43.1 kD	0.089	100.000	NAIDGK	8.939	>99%
7	278 to 283	TASD.AA	29.9 kD	29.8 kD	0.140	62.069	TDADAA	8.691	>99%
8	194 to 199	KAVD.SL	20.7 kD	39.1 kD	0.102	74.074	RAIDAL	7.569	>99%
9	64 to 69	TSVD.LE	7.0 kD	52.8 kD	0.109	57.143	TAVDAK	6.227	>99%
10	149 to 154	RVVD.AL	16.0 kD	43.7 kD	0.052	81.481	RAIDAL	4.208	>99%
11	264 to 269	TDAD.AM	28.5 kD	31.3 kD	0.047	82.759	TDADAA	3.872	>99%
12	136 to 141	AIVD.VP	14.6 kD	45.1 kD	0.032	56.250	GQVDVP	1.783	>95%
13	76 to 81	SIGD.GI	8.2 kD	51.5 kD	0.024	70.000	VLGDGV	1.679	>95%
14	262 to 267	RLTD.AD	28.3 kD	31.4 kD	0.016	56.250	DMTDSD	0.916	>95%
15	60 to 65	LGAD.TS	6.6 kD	53.2 kD	0.013	54.839	LGTDSD	0.691	>95%
16	526 to 531	IRSD.GK	57.1 kD	2.6 kD	0.011	60.000	SRVDGK	0.668	>95%
17	494 to 499	GYLD.KL	53.5 kD	6.2 kD	0.008	54.545	GFLDSY	0.429	>95%
18	126 to 131	KEGD.VV	13.6 kD	46.1 kD	0.003	55.556	SESDAV	0.186	>95%
19	221 to 226	IAID.TI	23.6 kD	36.2 kD	0.003	62.963	MAVDAV	0.179	>95%
20	299 to 304	YFRD.NG	32.2 kD	27.5 kD	0.003	43.333	LVIDNG	0.138	>95%

>DEK_MOUSE_

rank	position	site	N fragment	C fragment	frequency score	similarity maxscore	similarity maxsite	average score	specificity
1	307 to 312	ESED.SS	35.0 kD	8.1 kD	0.571	82.759	EQEDSS	47.243	>99%
2	40 to 45	DEDD.DE	4.5 kD	38.6 kD	0.156	88.571	DEDDDD	13.775	>99%
3	44 to 49	DEED.EE	5.0 kD	38.1 kD	0.176	75.000	DEEDDS	13.219	>99%
4	310 to 315	DSSD.DE	35.3 kD	7.8 kD	0.135	86.207	DSSDSE	11.652	>99%
5	39 to 44	DDED.DD	4.4 kD	38.7 kD	0.085	100.000	DDEDDD	8.485	>99%
6	36 to 41	EEED.DE	4.1 kD	39.1 kD	0.070	75.000	DEEDDS	5.284	>99%
7	166 to 171	EVLD.LE	19.3 kD	23.8 kD	0.085	58.621	DVVDAE	4.979	>99%
8	10 to 15	EGED.AP	1.2 kD	42.0 kD	0.071	58.621	EQEDAS	4.172	>99%
9	337 to 342	LLAD.AN	38.4 kD	4.7 kD	0.048	53.571	VLVDAG	2.575	>95%
10	37 to 42	EEDD.ED	4.2 kD	39.0 kD	0.018	77.143	DEDDDD	1.364	>95%
11	236 to 241	SDED.EK	27.0 kD	16.1 kD	0.018	58.065	DEEDSK	1.032	>95%
12	234 to 239	SSSD.ED	26.8 kD	16.4 kD	0.013	55.172	DSSDSE	0.740	>95%
13	288 to 293	KKAD.SS	32.9 kD	10.2 kD	0.010	60.714	LKTDSS	0.590	>95%
14	311 to 316	SSDD.EP	35.4 kD	7.7 kD	0.011	51.515	DSTDKP	0.570	>95%
15	247 to 252	SSED.EE	28.3 kD	14.8 kD	0.011	51.724	DSSDSE	0.562	>95%
16	41 to 46	EDDD.EE	4.7 kD	38.5 kD	0.005	60.606	DDNDSE	0.275	>95%
17	229 to 234	ILSD.ES	26.3 kD	16.9 kD	0.005	50.000	TLTDSS	0.273	>95%
18	360 to 365	PAYD.LT	41.1 kD	2.0 kD	0.001	51.613	DLFDLT	0.070	>95%
19	363 to 368	DLTE.RK	41.5 kD	1.7 kD	0.001	51.724	DLTDAA	0.063	>95%
20	105 to 110	KKPD.EL	12.2 kD	30.9 kD	0.001	54.839	EEPDSI	0.056	>95%

>EED_
MOUS
E_

rank	position	site	N fragment	C fragment	frequency score	similarity maxscore	similarity maxsite	average score	specificity
1	36 to 41	DEND.DA	4.1 kD	46.1 kD	0.631	75.000	DEEDDS	47.304	>99%
2	137 to 142	SYVD.AD	15.5 kD	34.7 kD	0.269	58.065	SYLDSG	15.626	>99%
3	139 to 144	VDAD.AD	15.6 kD	34.5 kD	0.051	64.286	IEADSE	3.272	>95%
4	338 to 343	DDID.KI	38.5 kD	11.7 kD	0.041	74.194	DEVDKM	3.031	>95%
5	418 to 423	FSRD.SS	47.8 kD	2.4 kD	0.052	46.429	LKTDSS	2.409	>95%
6	29 to 34	SNPD.LS	3.4 kD	46.8 kD	0.035	57.576	DNPDAA	2.043	>95%
7	51 to 56	ERPD.TP	5.7 kD	44.5 kD	0.025	70.968	ERVDSP	1.808	>95%
8	220 to 225	IQTD.TL	24.8 kD	25.4 kD	0.024	65.517	IETDAM	1.605	>95%
9	276 to 281	ESYD.YN	31.1 kD	19.0 kD	0.021	67.742	ESVDYR	1.445	>95%
10	307 to 312	NYVD.CV	34.9 kD	15.2 kD	0.015	56.250	DYTDAV	0.846	>95%
11	37 to 42	ENDD.AV	4.2 kD	46.0 kD	0.015	51.852	SESDAV	0.769	>95%
12	240 to 245	LSAD.YD	26.9 kD	23.3 kD	0.012	58.621	LSSDFT	0.733	>95%
13	237 to 242	DEVL.SA	26.6 kD	23.6 kD	0.015	48.276	DEVDSL	0.716	>95%
14	242 to 247	ADYD.LL	27.2 kD	23.0 kD	0.016	42.857	LDVDSL	0.683	>95%
15	428 to 433	VCDD.AS	48.8 kD	1.3 kD	0.013	53.125	SCTDSS	0.681	>95%
16	11 to 16	AGTD.MP	1.4 kD	48.7 kD	0.010	48.571	CSTDSP	0.483	>95%
17	209 to 214	VSKD.HA	23.5 kD	26.7 kD	0.009	41.935	VMRDPA	0.392	>95%
18	141 to 146	ADAD.EN	15.8 kD	34.3 kD	0.005	48.276	TDSDSS	0.260	>95%
19	23 to 28	LSSD.EN	2.7 kD	47.5 kD	0.005	51.724	LSSDFT	0.259	>95%
20	336 to 341	MEDD.ID	38.2 kD	11.9 kD	0.004	51.515	DEEDMD	0.205	>95%

>ATRN_MOUSE_

rank	position	site	N fragment	C fragment	frequency score	similarity maxscore	similarity maxsite	average score	specificity
1	143 to 148	FVTD.GP	14.9 kD	143.1 kD	0.154	72.727	EITDGP	11.165	>99%
2	1394 to 1399	ALVD.IS	154.6 kD	3.4 kD	0.100	59.259	AAVDTS	5.932	>99%
3	207 to 212	PERD.GN	22.3 kD	135.7 kD	0.082	71.875	PELDGS	5.870	>99%
4	1334 to 1339	LETD.EE	148.6 kD	9.4 kD	0.054	64.286	IEADSE	3.469	>95%
5	885 to 890	EPSD.AG	98.6 kD	59.4 kD	0.048	66.667	EVTDAG	3.186	>95%
6	956 to 961	QCVD.SN	106.1 kD	52.0 kD	0.032	75.758	ECVDSE	2.421	>95%
7	332 to 337	EYSD.LK	36.0 kD	122.1 kD	0.031	51.613	DYLDLA	1.623	>95%
8	536 to 541	YDVD.TQ	59.0 kD	99.1 kD	0.025	59.375	DDVDTK	1.505	>95%
9	1046 to 1051	CLED.SR	115.7 kD	42.3 kD	0.025	41.935	AMEDGE	1.068	>95%
10	408 to 413	GKID.ST	44.6 kD	113.4 kD	0.019	54.839	RKLDNT	1.058	>95%
11	1158 to 1163	LLID.YQ	128.0 kD	30.0 kD	0.021	48.387	MEVDYS	1.026	>95%
12	806 to 811	ENYD.NA	89.5 kD	68.6 kD	0.017	56.250	DNIDNL	0.930	>95%
13	186 to 191	YVYD.GD	20.2 kD	137.9 kD	0.014	64.706	DVFDGD	0.905	>95%
14	1232 to 1237	EYKD.SF	136.5 kD	21.5 kD	0.015	60.606	FYSDSF	0.888	>95%
15	635 to 640	LLSD.VL	70.2 kD	87.8 kD	0.016	50.000	LISDTY	0.800	>95%
16	229 to 234	FFSD.AA	24.7 kD	133.4 kD	0.013	54.545	FYSDSF	0.707	>95%
17	1169 to 1174	SQED.DR	129.4 kD	28.7 kD	0.011	60.606	EGEDDR	0.690	>95%
18	545 to 550	ILKD.SR	60.1 kD	98.0 kD	0.008	68.966	VLKDTQ	0.562	>95%
19	504 to 509	SVYD.DR	55.2 kD	102.8 kD	0.011	48.571	DTYDGR	0.510	>95%
20	645 to 650	EQCD.AH	71.3 kD	86.7 kD	0.007	72.727	EECDAA	0.498	>95%

>A4_MOUSE_

rank	position	site	N fragment	C fragment	frequency score	similarity maxscore	similarity maxsite	average score	specificity
1	194 to 199	DSVD.SA	22.1 kD	64.6 kD	25.873	84.615	SSVDSA	2189.217	>99.9%
2	736 to 741	VEVD.AA	83.0 kD	3.7 kD	3.897	100.000	VEVDAA	389.650	>99.9%
3	560 to 565	DEVD.EL	63.8 kD	22.9 kD	3.266	86.207	DEVDSL	281.543	>99.9%
4	216 to 221	DYAD.GG	24.5 kD	62.2 kD	1.923	87.879	DYADGA	169.018	>99.9%
5	191 to 196	EESD.SV	21.8 kD	64.9 kD	1.407	75.862	EEADSM	106.758	>99.9%
6	197 to 202	DSAD.AE	22.4 kD	64.3 kD	0.770	79.310	DSSDSE	61.050	>99.9%
7	247 to 252	DVED.GD	27.9 kD	58.8 kD	0.787	73.529	DVFDGD	57.849	>99.9%
8	202 to 207	EEDD.SD	22.9 kD	63.8 kD	0.261	71.429	DEDDDD	18.629	>99%
9	370 to 375	STPD.AV	41.3 kD	45.4 kD	0.277	61.290	DSPDSV	16.995	>99%
10	373 to 378	DAVD.KY	41.6 kD	45.1 kD	0.166	63.333	DSIDSF	10.482	>99%
11	213 to 218	ADTD.YA	24.1 kD	62.6 kD	0.133	57.143	SETDSA	7.627	>99%
12	240 to 245	EEAD.DD	27.0 kD	59.7 kD	0.108	65.714	DEDDDD	7.081	>99%
13	638 to 643	EPVD.AR	72.5 kD	14.2 kD	0.092	62.069	EEIDAQ	5.708	>99%
14	201 to 206	AEED.DS	22.8 kD	63.9 kD	0.076	75.000	DEEDDS	5.679	>99%
15	244 to 249	DDED.VE	27.5 kD	59.2 kD	0.068	70.968	DEEDLQ	4.817	>99%
16	624 to 629	FGVD.SV	71.0 kD	15.7 kD	0.078	55.556	MAVDAV	4.322	>99%
17	20 to 25	VPTD.GN	2.5 kD	84.2 kD	0.056	71.875	LQTDGN	4.055	>99%
18	233 to 238	EVAD.VE	26.2 kD	60.5 kD	0.020	58.621	DVVDAE	1.195	>95%
19	122 to 127	FVSD.AL	13.9 kD	72.8 kD	0.022	55.172	VVPDAL	1.187	>95%
20	310 to 315	DVTE.GK	34.9 kD	51.8 kD	0.015	75.000	DATDGK	1.098	>95%

>CATL
1_MO
USE_

rank	position	site	N fragment	C fragment	frequency score	similarity maxscore	similarity maxsite	average score	specificity
1	286 to 291	EGTD.SN	32.4 kD	5.1 kD	0.276	61.290	LGTDSD	16.887	>99%
2	205 to 210	EAKD.GS	23.8 kD	13.7 kD	0.166	70.968	EERDGS	11.762	>99%
3	247 to 252	VAMD.AS	28.2 kD	9.3 kD	0.056	55.556	VEVDAA	3.126	>95%
4	165 to 170	NLVD.CS	19.4 kD	18.1 kD	0.053	51.724	DLVDAE	2.725	>95%
5	194 to 199	GGLD.SE	22.5 kD	15.0 kD	0.059	46.429	SGVDAA	2.719	>95%
6	224 to 229	GFVD.IP	25.8 kD	11.7 kD	0.016	71.875	GQVDVP	1.169	>95%
7	181 to 186	GLMD.FA	21.0 kD	16.5 kD	0.017	44.828	DMLDLA	0.741	>95%
8	219 to 224	VAND.TG	25.3 kD	12.2 kD	0.009	50.000	IETDSG	0.427	>95%
9	116 to 121	KSVD.WR	14.1 kD	23.4 kD	0.002	70.968	ESVDYR	0.126	>95%
10	197 to 202	DSEE.SY	22.8 kD	14.7 kD	0.002	63.636	DEQDSY	0.115	>95%
11	22 to 27	DQTF.SA	2.7 kD	34.8 kD	0.002	60.000	DQTDSS	0.109	>95%
12	272 to 277	KNLD.HG	31.0 kD	6.5 kD	0.001	53.125	EEIDHA	0.065	>95%
13	316 to 321	KDRD.NH	36.0 kD	1.5 kD	0.001	59.375	AQRDSH	0.057	>95%

>SRSF
4_MO
USE_

rank	position	site	N fragment	C fragment	frequency score	similarity maxscore	similarity maxsite	average score	specificity
1	44 to 49	DLRD.AD	5.6 kD	50.3 kD	0.452	67.742	DLRDGS	30.616	>99%
2	29 to 34	LEVD.LK	3.9 kD	52.1 kD	0.454	55.172	LEVDAG	25.065	>99%
3	47 to 52	DADD.AV	5.9 kD	50.0 kD	0.268	67.742	DMDDVV	18.173	>99%
4	158 to 163	EKLD.GT	18.6 kD	37.3 kD	0.173	64.516	RKLDNT	11.164	>99%
5	40 to 45	VEFD.DL	5.1 kD	50.8 kD	0.054	81.250	VDFDDI	4.377	>99%
6	377 to 382	SKRD.SK	44.2 kD	11.8 kD	0.069	53.571	SSTDAK	3.696	>95%
7	119 to 124	DLKD.YM	14.1 kD	41.8 kD	0.036	82.353	DLKDHM	2.937	>95%
8	293 to 298	SRHD.SK	34.0 kD	22.0 kD	0.026	60.000	DRHDSG	1.534	>95%
9	131 to 136	TYAD.AH	15.5 kD	40.4 kD	0.018	48.649	CYADVY	0.894	>95%
10	73 to 78	PRRD.GS	8.9 kD	47.1 kD	0.011	65.625	PELDGS	0.747	>95%
11	394 to 399	KDTD.HS	45.9 kD	10.0 kD	0.009	70.588	DETDHS	0.604	>95%
12	149 to 154	SYSD.MK	17.5 kD	38.4 kD	0.008	46.429	SSTDAK	0.382	>95%
13	279 to 284	QNND.SA	32.5 kD	23.4 kD	0.005	65.517	QETDSA	0.358	>95%
14	46 to 51	RDAD.DA	5.8 kD	50.1 kD	0.004	58.621	TDADAA	0.239	>95%
15	250 to 255	PSKD.NK	29.2 kD	26.8 kD	0.004	50.000	ASTDSK	0.177	>95%
16	41 to 46	EFDD.LR	5.3 kD	50.7 kD	0.004	44.118	DFQDGR	0.162	>95%
17	172 to 177	LVED.KP	20.2 kD	35.8 kD	0.004	42.857	YVPDSP	0.159	>95%
18	261 to 266	RSPD.KS	30.5 kD	25.5 kD	0.003	60.714	ESVDKS	0.157	>95%
19	116 to 121	SWQD.LK	13.8 kD	42.2 kD	0.003	40.541	GWADER	0.139	>95%
20	14 to 19	RERD.VE	2.1 kD	53.9 kD	0.002	46.429	KESDLS	0.095	>95%

>SYHM_MOUSE_

rank	position	site	N fragment	C fragment	frequency score	similarity maxscore	similarity maxsite	average score	specificity
1	318 to 323	ISLD.LS	36.4 kD	20.5 kD	0.047	48.276	VSLDSP	2.273	>95%
2	215 to 220	RVVD.GI	25.0 kD	32.0 kD	0.033	62.069	SVVDGT	2.026	>95%
3	116 to 121	DLKD.QG	13.5 kD	43.5 kD	0.032	62.500	DLLDDG	1.985	>95%
4	239 to 244	DKLD.KM	27.6 kD	29.4 kD	0.022	77.419	DEVDKM	1.714	>95%
5	185 to 190	MIPD.AE	21.6 kD	35.4 kD	0.019	62.069	VVPDAL	1.199	>95%
6	236 to 241	SSMD.KL	27.2 kD	29.8 kD	0.013	73.077	SSVDSL	0.948	>95%
7	262 to 267	EVAD.RI	30.1 kD	26.9 kD	0.012	60.714	DIADAV	0.745	>95%
8	61 to 66	GTRD.LS	7.1 kD	49.9 kD	0.013	51.724	STTDLT	0.669	>95%
9	456 to 461	EKAD.IP	51.8 kD	5.2 kD	0.009	67.742	EEADSP	0.641	>95%
10	298 to 303	GLGD.LK	34.1 kD	22.9 kD	0.009	48.387	DMGDLV	0.412	>95%
11	174 to 179	CDFD.IA	20.4 kD	36.5 kD	0.006	53.333	DDIDVA	0.294	>95%
12	180 to 185	GEFD.PM	21.1 kD	35.9 kD	0.005	61.290	VEVDPM	0.280	>95%
13	113 to 118	LMYD.LK	13.2 kD	43.8 kD	0.005	50.000	LMVDGK	0.238	>95%
14	90 to 95	KGLD.TP	10.4 kD	46.6 kD	0.004	57.143	DGLDGP	0.221	>95%
15	279 to 284	LVED.LF	32.0 kD	25.0 kD	0.003	50.000	LISDTY	0.172	>95%
16	368 to 373	AQFD.PK	41.7 kD	15.3 kD	0.003	50.000	TQFDAA	0.154	>95%
17	361 to 366	GRYD.NL	40.9 kD	16.1 kD	0.003	52.941	GSYDSY	0.147	>95%
18	203 to 208	QLGD.FL	23.6 kD	33.4 kD	0.003	48.387	DMGDLV	0.137	>95%
19	430 to 435	ELWD.AG	48.8 kD	8.2 kD	0.002	70.000	EVMDAG	0.117	>95%
20	339 to 344	VLLE.SP	38.8 kD	18.2 kD	0.001	65.517	VSLDSP	0.068	>95%

>RALY_MOUSE_

rank	position	site	N fragment	C fragment	frequency score	similarity maxscore	similarity maxsite	average score	specificity
1	305 to 310	DAED.GA	32.8 kD	0.4 kD	2.461	71.875	DEQDGA	176.912	>99.9%
2	199 to 204	SNID.AL	22.4 kD	10.8 kD	0.067	73.077	SSVDSL	4.888	>99%
3	217 to 222	ANPD.GK	24.4 kD	8.8 kD	0.072	67.647	DSPDGK	4.867	>99%
4	302 to 307	QDTD.AE	32.5 kD	0.7 kD	0.042	62.069	QETDSA	2.618	>95%
5	283 to 288	DDGD.EE	30.3 kD	2.9 kD	0.024	69.697	DDNDSE	1.670	>95%
6	264 to 269	PQED.TA	28.4 kD	4.8 kD	0.007	58.621	EQEDSS	0.430	>95%
7	281 to 286	TRDD.GD	30.1 kD	3.0 kD	0.007	50.000	TSTDGS	0.373	>95%
8	280 to 285	QTRD.DG	30.0 kD	3.2 kD	0.004	58.065	EERDSG	0.213	>95%
9	223 to 228	KKGD.SS	25.0 kD	8.2 kD	0.003	55.556	SESDSS	0.187	>95%
10	83 to 88	QTLD.IN	9.3 kD	23.8 kD	0.003	53.333	EEVDLN	0.161	>95%
11	112 to 117	YSFD.YD	12.5 kD	20.7 kD	0.002	38.710	ESVDYR	0.085	>95%
12	114 to 119	FDYD.YY	12.8 kD	20.4 kD	0.002	52.500	DYYDYY	0.083	>95%
13	296 to 301	EELE.HS	31.8 kD	1.4 kD	0.001	71.875	EEIDHA	0.069	>95%

>CAT
K_MO
USE_

rank	position	site	N fragment	C fragment	frequency score	similarity maxscore	similarity maxsite	average score	specificity
1	196 to 201	DSED.AY	22.5 kD	14.3 kD	0.559	66.667	DSLDAF	37.282	>99%
2	117 to 122	DSID.YR	14.1 kD	22.8 kD	0.234	87.097	ESVDYR	20.401	>99%
3	247 to 252	VSID.AS	28.0 kD	8.8 kD	0.267	67.857	VSVDAF	18.121	>99%
4	20 to 25	EMLD.TQ	2.7 kD	34.1 kD	0.047	63.333	ESLDNQ	2.997	>95%
5	166 to 171	NLVD.CV	19.3 kD	17.5 kD	0.035	69.697	KLTDCV	2.426	>95%
6	193 to 198	GGID.SE	22.2 kD	14.7 kD	0.031	53.571	SGVDAA	1.641	>95%
7	39 to 44	SKVD.EI	5.2 kD	31.7 kD	0.012	61.538	SSVDSL	0.730	>95%
8	114 to 119	RVPD.SI	13.8 kD	23.1 kD	0.010	62.069	SVPDSS	0.628	>95%
9	269 to 274	CDRD.NV	30.6 kD	6.2 kD	0.010	53.125	DTRDNV	0.517	>95%
10	101 to 106	YSND.TL	12.2 kD	24.6 kD	0.004	38.462	SSVDSL	0.138	>95%
11	204 to 209	VGQD.ES	23.4 kD	13.4 kD	0.003	39.394	EGEDDR	0.110	>95%
12	263 to 268	VYYD.EN	29.9 kD	7.0 kD	0.002	43.590	DYYDDY	0.068	>95%

>TRA2
B_MO
USE_

rank	position	site	N fragment	C fragment	frequency score	similarity maxscore	similarity maxsite	average score	specificity
1	182 to 187	MELD.GR	21.5 kD	12.2 kD	0.512	74.194	TEIDGR	38.002	>99%
2	231 to 236	DDRD.YY	27.3 kD	6.3 kD	0.107	64.706	DDADYK	6.914	>99%
3	167 to 172	ENVD.DA	19.8 kD	13.9 kD	0.100	57.692	SSVDSA	5.778	>99%
4	220 to 225	DYYD.RG	26.0 kD	7.7 kD	0.064	67.742	DIVDRG	4.314	>99%
5	130 to 135	TERD.LR	15.4 kD	18.2 kD	0.042	50.000	TEKDSM	2.097	>95%
6	39 to 44	SKED.SR	4.5 kD	29.2 kD	0.031	58.065	DEEDSK	1.792	>95%
7	168 to 173	NVDD.AK	19.9 kD	13.7 kD	0.012	50.000	DVTDAQ	0.623	>95%
8	189 to 194	IRVD.FS	22.3 kD	11.3 kD	0.009	58.065	MEVDYS	0.530	>95%
9	143 to 148	PIAD.VS	16.9 kD	16.7 kD	0.003	55.556	TIADLA	0.165	>95%
10	86 to 91	YSRD.YR	10.3 kD	23.3 kD	0.003	54.839	ESVDYR	0.149	>95%
11	252 to 257	AAQD.RD	29.5 kD	4.2 kD	0.002	47.059	GEDDRD	0.082	>95%
12	229 to 234	GYDD.RD	27.1 kD	6.6 kD	0.001	79.412	GEDDRD	0.061	>95%
13	112 to 117	ANPD.PN	13.5 kD	20.2 kD	0.001	42.424	DNPDAA	0.061	>95%

>TRFE_MOUSE_

rank	position	site	N fragment	C fragment	frequency score	similarity maxscore	similarity maxsite	average score	specificity
1	74 to 79	SEAD.AM	8.2 kD	68.5 kD	0.336	77.778	SESDAV	26.167	>99%
2	79 to 84	MTLD.GG	8.8 kD	67.9 kD	0.259	62.500	TELDGG	16.184	>99%
3	407 to 412	MTLD.GG	45.0 kD	31.7 kD	0.259	62.500	TELDGG	16.184	>99%
4	402 to 407	GEAD.AM	44.5 kD	32.2 kD	0.105	72.414	NEADAL	7.613	>99%
5	49 to 54	LPPD.GP	5.6 kD	71.1 kD	0.057	66.667	ADPDGP	3.775	>99%
6	353 to 358	GSID.NS	39.0 kD	37.7 kD	0.063	57.692	SSVDSA	3.654	>95%
7	582 to 587	LCPD.GT	64.3 kD	12.4 kD	0.041	73.529	MMPDGT	3.025	>95%
8	557 to 562	TVLD.NT	61.4 kD	15.3 kD	0.046	63.333	DVLDNV	2.882	>95%
9	213 to 218	CLKD.GG	23.1 kD	53.6 kD	0.042	60.606	CVVDAG	2.535	>95%
10	313 to 318	LFKD.SA	34.5 kD	42.2 kD	0.025	41.379	VLKDTQ	1.040	>95%
11	474 to 479	TGVD.RT	52.1 kD	24.6 kD	0.016	57.143	SGVDAA	0.900	>95%
12	63 to 68	SYPD.CI	7.2 kD	69.6 kD	0.011	50.000	DYPDSS	0.559	>95%
13	245 to 250	LCLD.NT	26.7 kD	50.0 kD	0.009	55.172	IVLDGT	0.469	>95%
14	546 to 551	EKGD.VA	60.2 kD	16.5 kD	0.008	53.125	DAGDVG	0.434	>95%
15	643 to 648	TTKD.LL	70.8 kD	5.9 kD	0.007	50.000	TEKDSM	0.371	>95%
16	19 to 24	AVPD.KT	2.2 kD	74.6 kD	0.005	65.517	SVPDSS	0.349	>95%
17	235 to 240	EKAD.RD	25.4 kD	51.3 kD	0.005	62.500	DEVDRD	0.303	>95%
18	560 to 565	DNTE.GK	61.7 kD	15.0 kD	0.003	72.727	DKTDGK	0.213	>95%
19	455 to 460	KASD.TS	50.0 kD	26.7 kD	0.003	60.714	KESDLS	0.196	>95%
20	85 to 90	WVYD.AG	9.5 kD	67.3 kD	0.003	53.333	EVMDAG	0.167	>95%

>CATZ_MOUSE_

rank	position	site	N fragment	C fragment	frequency score	similarity maxscore	similarity maxsite	average score	specificity
1	252 to 257	VSND.GI	28.3 kD	5.7 kD	0.032	75.000	VASDGV	2.394	>95%
2	286 to 291	GTGD.SY	32.2 kD	1.8 kD	0.014	61.765	GSYDSY	0.891	>95%
3	124 to 129	NVID.CG	14.2 kD	19.7 kD	0.011	56.667	EVMDAG	0.607	>95%
4	60 to 65	SPAD.LP	7.0 kD	27.0 kD	0.011	45.161	EEADSP	0.497	>95%
5	234 to 239	EHQD.QA	26.4 kD	7.5 kD	0.004	60.000	EHNDGA	0.218	>95%
6	150 to 155	GIPD.ET	16.9 kD	17.0 kD	0.003	58.621	SVPDSS	0.169	>95%
7	136 to 141	GGND.LP	15.3 kD	18.7 kD	0.003	46.875	GQVDVP	0.124	>95%
8	102 to 107	AMAD.RI	11.7 kD	22.2 kD	0.002	42.857	ALDDLI	0.078	>95%
9	191 to 196	RVGD.YG	21.8 kD	12.2 kD	0.001	54.545	DLADYG	0.058	>95%

>PHB1 _MOUSE_

rank	position	site	N fragment	C fragment	frequency score	similarity maxscore	similarity maxsite	average score	specificity
1	214 to 219	AEGD.SK	23.9 kD	5.9 kD	0.214	60.714	ASTDSK	12.992	>99%
2	230 to 235	TAGD.GL	25.5 kD	4.4 kD	0.081	72.414	TVADGL	5.862	>99%
3	28 to 33	YNVD.AG	3.1 kD	26.7 kD	0.036	65.517	LEVDAG	2.391	>95%
4	163 to 168	LILD.DV	18.3 kD	11.5 kD	0.017	48.276	IVLDGT	0.837	>95%
5	132 to 137	ARFD.AG	14.9 kD	14.9 kD	0.012	64.286	AAVDAG	0.766	>95%
6	243 to 248	AAED.IA	26.9 kD	2.9 kD	0.011	44.444	AAVDTS	0.480	>95%
7	112 to 117	EDYD.ER	12.7 kD	17.1 kD	0.007	50.000	EDLDGK	0.348	>95%
8	150 to 155	VSDD.LT	16.9 kD	12.9 kD	0.006	55.172	LSSDFT	0.305	>95%
9	149 to 154	QVSD.DL	16.8 kD	13.0 kD	0.006	46.875	DETDDL	0.263	>95%
10	81 to 86	GSKD.LQ	9.1 kD	20.7 kD	0.005	48.387	DEEDLQ	0.244	>95%
11	44 to 49	GVQD.IV	4.9 kD	24.9 kD	0.003	60.000	GVEDTL	0.198	>95%
12	153 to 158	DLTE.RA	17.3 kD	12.5 kD	0.002	68.966	DLTDAA	0.162	>95%
13	164 to 169	ILDD.VS	18.4 kD	11.4 kD	0.003	50.000	ALDDLI	0.156	>95%
14	181 to 186	EAVE.AK	20.3 kD	9.5 kD	0.002	64.286	TAVDAK	0.113	>95%
15	110 to 115	IGED.YD	12.4 kD	17.4 kD	0.001	54.839	LGTDSD	0.056	>95%

>OGF
D1_M
OUSE_

rank	position	site	N fragment	C fragment	frequency score	similarity maxscore	similarity maxsite	average score	specificity
1	147 to 152	EFTD.AL	17.3 kD	45.4 kD	0.331	70.000	DFLDAL	23.149	>99%
2	183 to 188	DLYD.TD	21.5 kD	41.3 kD	0.090	60.000	DLFDSA	5.410	>99%
3	25 to 30	EFSD.AV	3.0 kD	59.7 kD	0.072	62.500	DYTDAV	4.518	>99%
4	275 to 280	LEMD.YQ	32.2 kD	30.5 kD	0.027	67.742	MEVDYS	1.847	>95%
5	157 to 162	DELE.GR	18.5 kD	44.3 kD	0.023	74.194	DSLDGR	1.691	>95%
6	336 to 341	NLPD.VL	39.6 kD	23.2 kD	0.020	56.250	TLPDGL	1.134	>95%
7	131 to 136	SGID.LE	15.5 kD	47.2 kD	0.017	64.516	DGVDLK	1.072	>95%
8	180 to 185	GTLD.LY	21.1 kD	41.6 kD	0.018	57.576	GFLDSY	1.016	>95%
9	84 to 89	LSLD.FH	9.7 kD	53.0 kD	0.016	55.172	LSSDFT	0.867	>95%
10	371 to 376	SEDD.ET	43.5 kD	19.3 kD	0.015	59.259	SESDSS	0.864	>95%
11	74 to 79	DFLE.GL	8.6 kD	54.1 kD	0.005	90.323	DFVEGL	0.479	>95%
12	418 to 423	EQAD.PE	48.2 kD	14.5 kD	0.008	55.172	EEADTS	0.466	>95%
13	99 to 104	QQSD.DL	11.7 kD	51.0 kD	0.005	59.375	DETDDL	0.276	>95%
14	374 to 379	DETE.EK	43.8 kD	18.9 kD	0.003	74.194	DETDSK	0.243	>95%
15	452 to 457	LVHD.NT	52.1 kD	10.6 kD	0.004	58.065	LVFDNQ	0.239	>95%
16	71 to 76	QSQD.FL	8.3 kD	54.5 kD	0.003	50.000	ESQDVS	0.175	>95%
17	137 to 142	PTID.MS	16.1 kD	46.6 kD	0.003	55.172	ETVDTS	0.171	>95%
18	100 to 105	QSDD.LK	11.8 kD	50.9 kD	0.003	46.429	ESQDVS	0.156	>95%
19	54 to 59	IALD.MD	6.3 kD	56.5 kD	0.003	61.290	TELDMD	0.153	>95%
20	506 to 511	VYRD.RE	58.3 kD	4.4 kD	0.002	57.576	VYRDGT	0.139	>95%

>NIP7_MOUSE_

rank	position	site	N fragment	C fragment	frequency score	similarity maxscore	similarity maxsite	average score	specificity
1	29 to 34	DRPD.GT	3.8 kD	16.6 kD	0.437	72.222	DRHDGT	31.567	>99%
2	26 to 31	LLVD.RP	3.4 kD	17.0 kD	0.069	62.500	LMIDGP	4.332	>99%
3	84 to 89	TALD.YL	10.1 kD	10.3 kD	0.066	51.724	TNLDSL	3.405	>95%
4	135 to 140	SMAD.IP	15.8 kD	4.6 kD	0.012	50.000	GQVDVP	0.619	>95%
5	149 to 154	STQD.CR	17.2 kD	3.2 kD	0.004	51.852	SSLDAR	0.191	>95%
6	165 to 170	HQAD.IG	19.0 kD	1.4 kD	0.002	56.250	HSLDAG	0.114	>95%
7	58 to 63	ISGD.KL	7.2 kD	13.3 kD	0.001	41.935	DEGDSL	0.055	>95%

>TNR1
A_MO
USE_

rank	posit ion	site	N fragmen t	C fragmen t	frequen cy score	similar ity maxscor e	similar ity maxsite	average score	specifi city
1	361 to 366	AVVD.GV	39.7 kD	10.5 kD	0.883	72.414	SVVDGT	63.921	>99.9%
2	352 to 357	DNAD.LA	38.7 kD	11.4 kD	0.129	83.871	DNTDLA	10.842	>99%
3	38 to 43	EKRD.SL	4.2 kD	45.9 kD	0.094	58.621	EEADSM	5.488	>99%
4	68 to 73	LVSD.CP	7.5 kD	42.6 kD	0.009	59.459	DITDCP	0.546	>95%
5	205 to 210	NPQD.SG	22.9 kD	27.2 kD	0.006	78.125	NPQDSV	0.497	>95%
6	142 to 147	QCVD.CS	15.9 kD	34.2 kD	0.008	60.606	ECVDSE	0.484	>95%
7	349 to 354	QRPD.NA	38.4 kD	11.7 kD	0.007	64.516	EEPDSA	0.448	>95%
8	119 to 124	ADKD.TV	13.2 kD	36.9 kD	0.006	50.000	TEKDSM	0.317	>95%
9	33 to 38	SLGD.RE	3.5 kD	46.6 kD	0.006	41.176	SECDRE	0.266	>95%
10	341 to 346	KWED.SA	37.5 kD	12.6 kD	0.004	51.724	EQEDSS	0.214	>95%
11	75 to 80	PGRD.TV	8.2 kD	41.9 kD	0.002	51.724	PAADAI	0.099	>95%
12	320 to 325	QGAD.PL	35.2 kD	15.0 kD	0.001	51.613	GSSDPL	0.061	>95%
13	117 to 122	CQAD.KD	13.0 kD	37.1 kD	0.001	46.429	IEADSE	0.053	>95%

>SCO1_MOUSE_

rank	position	site	N fragment	C fragment	frequency score	similarity maxscore	similarity maxsite	average score	specificity
1	215 to 220	EEID.GV	24.1 kD	7.5 kD	2.916	83.871	DEVDGV	244.535	>99.9%
2	168 to 173	EEID.SI	18.9 kD	12.7 kD	0.801	89.286	QEIDSI	71.476	>99.9%
3	250 to 255	IGPD.GE	28.1 kD	3.5 kD	0.027	55.263	DGPDGP	1.487	>95%
4	188 to 193	PERD.TK	21.1 kD	10.5 kD	0.019	51.613	DEEDSK	1.005	>95%
5	235 to 240	EDED.YI	26.3 kD	5.3 kD	0.015	53.333	DEEDIL	0.790	>95%
6	26 to 31	GLRD.VA	3.3 kD	28.3 kD	0.019	41.379	DVTDIA	0.767	>95%
7	255 to 260	EFLD.YF	28.6 kD	3.0 kD	0.013	53.333	DFLDAL	0.715	>95%
8	134 to 139	TDKD.YL	14.8 kD	16.8 kD	0.010	60.000	TEKDSM	0.599	>95%
9	239 to 244	YIVD.HT	26.8 kD	4.8 kD	0.003	48.276	SVVDGT	0.158	>95%
10	184 to 189	ITID.PE	20.6 kD	11.0 kD	0.001	56.250	FVVDPQ	0.065	>95%

>CASC
3_MO
USE_

rank	position	site	N fragment	C fragment	frequency score	similarity maxscore	similarity maxsite	average score	specificity
1	119 to 124	DAAD.SS	12.4 kD	63.4 kD	2.257	82.143	DALDSS	185.372	>99.9%
2	361 to 366	PEPD.AP	39.9 kD	35.8 kD	0.225	85.714	PAPDAP	19.311	>99%
3	77 to 82	ESED.GM	7.9 kD	67.9 kD	0.183	65.625	EEEDGK	11.985	>99%
4	21 to 26	SGSD.SG	2.6 kD	73.1 kD	0.178	60.000	SGVDIG	10.698	>99%
5	169 to 174	DDED.RK	17.7 kD	58.0 kD	0.119	74.194	DEEDSK	8.860	>99%
6	116 to 121	EAND.AA	12.1 kD	63.6 kD	0.113	60.714	DALDAA	6.851	>99%
7	93 to 98	SAED.SE	9.5 kD	66.2 kD	0.088	55.172	DSSDSE	4.868	>99%
8	82 to 87	MEGD.AV	8.4 kD	67.4 kD	0.071	66.667	SESDAV	4.702	>99%
9	99 to 104	GEED.YS	10.2 kD	65.6 kD	0.068	54.839	MEVDYS	3.715	>95%
10	448 to 453	APVD.ST	49.0 kD	26.8 kD	0.054	55.556	AAVDTS	3.018	>95%
11	457 to 462	LEQD.VA	49.9 kD	25.9 kD	0.042	53.571	MEVDAA	2.226	>95%
12	12 to 17	DTED.EE	1.8 kD	73.9 kD	0.029	62.500	DTVDNE	1.786	>95%
13	144 to 149	QSGD.GQ	15.0 kD	60.8 kD	0.020	66.667	ESVDGQ	1.306	>95%
14	554 to 559	THGD.SP	60.8 kD	15.0 kD	0.024	51.613	EEADSP	1.258	>95%
15	383 to 388	AVPD.IT	42.1 kD	33.6 kD	0.010	68.966	IVPDIA	0.662	>95%
16	87 to 92	VLSD.YE	8.8 kD	66.9 kD	0.012	48.276	VLKDTQ	0.566	>95%
17	390 to 395	PAPD.RP	42.8 kD	32.9 kD	0.006	85.714	PAPDAP	0.511	>95%
18	315 to 320	GFKD.GR	34.8 kD	40.9 kD	0.007	67.647	DFQDGR	0.447	>95%
19	9 to 14	ASQD.TE	1.5 kD	74.3 kD	0.006	53.571	ASTDSK	0.305	>95%
20	410 to 415	KVGD.AV	45.2 kD	30.5 kD	0.005	61.290	DTGDAV	0.279	>95%

>HIC2_MOUSE_

rank	position	site	N fragment	C fragment	frequency score	similarity maxscore	similarity maxsite	average score	specificity
1	409 to 414	GSED.SG	43.5 kD	23.3 kD	0.147	53.333	ESMDSG	7.859	>99%
2	491 to 496	EAED.LS	52.2 kD	14.5 kD	0.053	67.857	ESQDVS	3.618	>95%
3	271 to 276	QLSD.SQ	29.2 kD	37.5 kD	0.032	58.621	DLVDSQ	1.880	>95%
4	177 to 182	GLVD.VR	19.9 kD	46.9 kD	0.032	53.125	GLLDPK	1.699	>95%
5	244 to 249	LGLD.LS	26.4 kD	40.3 kD	0.035	43.333	SGVDIG	1.517	>95%
6	83 to 88	LDTD.MV	9.7 kD	57.0 kD	0.021	57.143	LDVDSL	1.221	>95%
7	75 to 80	VLHD.NL	8.8 kD	57.9 kD	0.018	44.828	TLRDSM	0.795	>95%
8	405 to 410	NGKD.GS	43.1 kD	23.6 kD	0.015	51.613	EERDGS	0.778	>95%
9	440 to 445	SYGD.NV	46.7 kD	20.0 kD	0.014	44.444	SVTDSV	0.611	>95%
10	502 to 507	YTAD.SR	53.3 kD	13.4 kD	0.014	42.857	IEADSE	0.607	>95%
11	196 to 201	GSDD.EL	21.9 kD	44.9 kD	0.004	61.290	GSSDPL	0.245	>95%
12	517 to 522	TYKD.PA	55.1 kD	11.7 kD	0.003	58.065	VMRDPA	0.166	>95%
13	307 to 312	MDVE.GA	32.9 kD	33.9 kD	0.002	70.968	MDIDGV	0.147	>95%
14	369 to 374	GTGD.RV	39.5 kD	27.2 kD	0.002	61.290	DTGDAV	0.139	>95%
15	318 to 323	SLLE.GQ	34.1 kD	32.7 kD	0.002	68.966	SVIDGQ	0.133	>95%
16	15 to 20	GHRD.MG	2.0 kD	64.7 kD	0.002	65.625	VHRDMA	0.120	>95%
17	265 to 270	TPED.PA	28.6 kD	38.1 kD	0.002	48.387	VMRDPA	0.106	>95%
18	95 to 100	QILD.FI	11.1 kD	55.7 kD	0.002	50.000	DVLDVL	0.100	>95%
19	81 to 86	INLD.TD	9.5 kD	57.3 kD	0.002	48.276	MEIDTS	0.094	>95%
20	305 to 310	EPMD.VE	32.7 kD	34.1 kD	0.002	50.000	ESQDVS	0.087	>95%

>PSA2_MOUSE_

rank	position	site	N fragment	C fragment	frequency score	similarity maxscore	similarity maxsite	average score	specificity
1	183 to 188	ELED.AI	20.6 kD	5.3 kD	0.070	62.963	ELVDSV	4.381	>99%
2	225 to 230	EVRD.YL	25.3 kD	0.6 kD	0.047	48.485	DVTDYK	2.289	>95%
3	79 to 84	MGPD.YR	8.6 kD	17.3 kD	0.004	45.161	ESVDYR	0.203	>95%
4	203 to 208	MTED.NI	22.8 kD	3.1 kD	0.003	53.125	DTRDNV	0.140	>95%
5	147 to 152	FQSD.PS	16.5 kD	9.4 kD	0.002	48.148	SESDSS	0.093	>95%

>PCP_
MOUS
E_

rank	position	site	N fragment	C fragment	frequency score	similarity maxscore	similarity maxsite	average score	specificity
1	438 to 443	DITD.TL	49.2 kD	5.8 kD	0.467	68.966	DITDSS	32.204	>99%
2	148 to 153	ALAD.FA	17.1 kD	37.9 kD	0.042	59.259	TIADLA	2.482	>95%
3	447 to 452	NIHD.GA	50.2 kD	4.8 kD	0.025	81.818	EVHDGA	2.066	>95%
4	133 to 138	SFKD.SQ	15.4 kD	39.6 kD	0.025	62.500	DFTDSQ	1.579	>95%
5	315 to 320	NVSD.TV	35.4 kD	19.6 kD	0.008	62.963	SVTDSV	0.523	>95%
6	205 to 210	WQLD.GM	23.2 kD	31.8 kD	0.008	60.714	SQLDSM	0.456	>95%
7	90 to 95	NEGD.IV	10.4 kD	44.7 kD	0.008	58.621	NEADAL	0.441	>95%
8	36 to 41	PTPD.PA	4.0 kD	51.0 kD	0.006	51.429	PAPDAP	0.306	>95%
9	425 to 430	GELD.PW	47.9 kD	7.1 kD	0.005	53.125	GLLDPK	0.260	>95%
10	240 to 245	NVID.KL	27.2 kD	27.8 kD	0.005	55.556	SLLDEL	0.252	>95%
11	435 to 440	VTRD.IT	48.9 kD	6.1 kD	0.003	51.724	STTDLT	0.134	>95%
12	461 to 466	NAFD.PS	51.8 kD	3.2 kD	0.002	50.000	DALDSS	0.100	>95%
13	372 to 377	NGID.DM	41.6 kD	13.4 kD	0.002	53.125	NKVDGM	0.097	>95%
14	220 to 225	VTND.FR	24.8 kD	30.2 kD	0.002	48.276	LSSDFT	0.092	>95%
15	129 to 134	FGQD.SF	15.0 kD	40.1 kD	0.002	57.576	FYSDSF	0.086	>95%
16	71 to 76	LVAD.KH	8.2 kD	46.8 kD	0.002	50.000	LISDTY	0.080	>95%
17	453 to 458	HHLD.LR	50.9 kD	4.2 kD	0.002	53.125	DHVDLS	0.080	>95%
18	53 to 58	QKVD.HF	6.0 kD	49.0 kD	0.001	56.250	EEIDHA	0.065	>95%
19	59 to 64	GFAD.MR	6.7 kD	48.3 kD	0.001	54.054	GWADER	0.053	>95%

>CD68 _MOUSE_

rank	position	site	N fragment	C fragment	frequency score	similarity maxscore	similarity maxsite	average score	specificity
1	18 to 23	TEED.CP	2.3 kD	32.5 kD	0.048	53.333	VEVDAP	2.566	>95%
2	260 to 265	VHLD.LL	27.8 kD	7.0 kD	0.029	53.125	VHRDMA	1.547	>95%
3	208 to 213	VYLD.YM	22.1 kD	12.7 kD	0.012	45.455	DYMDAM	0.554	>95%
4	273 to 278	QLPD.KG	29.2 kD	5.6 kD	0.007	48.485	SQPDTG	0.320	>95%
5	175 to 180	GGCD.GT	18.4 kD	16.4 kD	0.003	51.613	DGADST	0.169	>95%

>COX1
7_MOU
SE

>U119 A_MOUSE_

rank	position	site	N fragment	C fragment	frequency score	similarity maxscore	similarity maxsite	average score	specificity
1	214 to 219	TQSD.SF	24.3 kD	2.7 kD	0.032	67.742	TETDAF	2.148	>95%
2	94 to 99	RDMD.SG	10.2 kD	16.8 kD	0.012	70.000	ESMDSG	0.816	>95%
3	230 to 235	NKAD.YS	26.2 kD	0.8 kD	0.004	51.613	MEVDYS	0.217	>95%
4	220 to 225	YFVD.DR	25.1 kD	1.9 kD	0.005	39.474	YWIDGS	0.187	>95%
5	150 to 155	TVGD.KP	16.5 kD	10.5 kD	0.003	40.741	TIADLA	0.123	>95%
6	61 to 66	GPED.VL	6.2 kD	20.7 kD	0.002	63.333	GVEDTL	0.099	>95%
7	33 to 38	AEAE.SG	3.4 kD	23.5 kD	0.002	58.621	VEVDSG	0.096	>95%
8	71 to 76	ITGD.YL	7.3 kD	19.7 kD	0.002	41.935	DTGDAV	0.091	>95%
9	84 to 89	YKID.FV	8.9 kD	18.1 kD	0.002	43.750	DGVDFV	0.087	>95%
10	221 to 226	FVDD.RL	25.2 kD	1.8 kD	0.001	63.333	FIQDRA	0.075	>95%

>U2AF2_MOUSE_

rank	position	site	N fragment	C fragment	frequency score	similarity maxscore	similarity maxsite	average score	specificity
1	253 to 258	VVPD.SA	29.4 kD	24.1 kD	0.625	75.862	IVPDIA	47.439	>99%
2	125 to 130	MTPD.GL	15.4 kD	38.1 kD	0.169	65.625	TLPDGL	11.099	>99%
3	464 to 469	CDPD.SY	52.4 kD	1.1 kD	0.076	66.667	CDPDAP	5.073	>99%
4	388 to 393	ELLD.DE	43.8 kD	9.7 kD	0.080	62.500	DLLDDG	5.005	>99%
5	290 to 295	LVKD.SA	33.5 kD	20.0 kD	0.068	44.828	IVPDIA	3.033	>95%
6	306 to 311	EYVD.IN	35.2 kD	18.3 kD	0.042	70.000	EEVDLN	2.972	>95%
7	419 to 424	RPVD.GV	47.4 kD	6.1 kD	0.016	64.706	YPVDGL	1.031	>95%
8	212 to 217	MAFD.GI	24.9 kD	28.6 kD	0.014	64.286	VASDGV	0.919	>95%
9	311 to 316	NVTD.QA	35.7 kD	17.8 kD	0.014	59.259	SVTDSV	0.812	>95%
10	438 to 443	SVFD.CQ	49.4 kD	4.1 kD	0.011	55.172	SVIDGQ	0.591	>95%
11	54 to 59	ASRD.RR	7.3 kD	46.2 kD	0.005	50.000	ASTDSK	0.254	>95%
12	401 to 406	DVRD.EC	45.4 kD	8.1 kD	0.005	53.125	DLRDDK	0.246	>95%
13	2 to 7	SDFD.EF	0.6 kD	52.9 kD	0.003	56.250	VDFDDI	0.181	>95%
14	203 to 208	RSVD.ET	23.9 kD	29.6 kD	0.003	57.143	ESVDKS	0.181	>95%
15	16 to 21	QERD.KE	2.4 kD	51.1 kD	0.003	51.613	EERDSG	0.132	>95%
16	398 to 403	IVED.VR	45.0 kD	8.5 kD	0.002	51.724	IVPDIA	0.083	>95%
17	269 to 274	YLND.DQ	31.1 kD	22.4 kD	0.002	44.828	VLKDTQ	0.070	>95%
18	389 to 394	LLDD.EE	43.9 kD	9.6 kD	0.002	38.710	AMEDGE	0.063	>95%
19	164 to 169	AMMD.FF	19.6 kD	33.9 kD	0.001	60.606	SMTDFY	0.053	>95%
20	422 to 427	DGVE.VP	47.7 kD	5.8 kD	0.001	63.636	DDVDIP	0.052	>95%

>CATS_MOUSE_

rank	position	site	N fragment	C fragment	frequency score	similarity maxscore	similarity maxsite	average score	specificity
1	295 to 300	GTLD.GK	33.6 kD	4.9 kD	0.253	68.750	DTVDGK	17.406	>99%
2	207 to 212	IEAD.AS	24.1 kD	14.3 kD	0.196	71.429	IEADSE	14.018	>99%
3	259 to 264	VGID.AS	29.8 kD	8.7 kD	0.082	64.286	SGVDAA	5.255	>99%
4	125 to 130	DTVD.WR	15.4 kD	23.1 kD	0.075	65.625	DTVDGK	4.924	>99%
5	122 to 127	TLPD.TV	15.1 kD	23.4 kD	0.072	65.625	TLPDGL	4.714	>99%
6	242 to 247	GDED.AL	28.1 kD	10.3 kD	0.057	60.000	GVEDTL	3.404	>95%
7	174 to 179	NLVD.CS	20.6 kD	17.9 kD	0.053	51.724	DLVDAE	2.725	>95%
8	200 to 205	YIID.NG	23.5 kD	15.0 kD	0.007	80.000	LVIDNG	0.587	>95%
9	87 to 92	DMGD.MT	11.0 kD	27.5 kD	0.007	80.645	DMGDLV	0.540	>95%
10	274 to 279	GVYD.DP	31.5 kD	7.0 kD	0.008	50.000	DLRDDP	0.376	>95%
11	30 to 35	PTLD.YH	3.8 kD	34.6 kD	0.003	63.889	DELDYH	0.211	>95%
12	216 to 221	KATD.EK	25.1 kD	13.3 kD	0.004	57.143	SSTDAK	0.210	>95%
13	298 to 303	DGKD.YW	33.9 kD	4.6 kD	0.003	50.000	DGVDFV	0.130	>95%
14	275 to 280	VYDD.PS	31.6 kD	6.8 kD	0.002	48.387	VMRDPA	0.103	>95%
15	26 to 31	LQRD.PT	3.4 kD	35.1 kD	0.002	61.290	VMRDPA	0.099	>95%
16	46 to 51	EYKD.KN	6.0 kD	32.4 kD	0.001	46.875	DYKDIA	0.061	>95%
17	84 to 89	GMND.MG	10.7 kD	27.8 kD	0.001	48.571	SYNDFG	0.052	>95%

>KGD4_MOUSE_

rank	position	site	N fragment	C fragment	frequency score	similarity maxscore	similarity maxsite	average score	specificity
1	59 to 64	TSPD.LL	6.4 kD	4.6 kD	0.108	58.065	DSPDSL	6.277	>99%
2	68 to 73	GPPD.TA	7.4 kD	3.7 kD	0.011	51.515	SQPDTG	0.574	>95%

>CHC H7_M OUSE_

rank	position	site	N fragment	C fragment	frequency score	similarity maxscore	similarity maxsite	average score	specificity
1	18 to 23	SESD.AS	2.4 kD	7.7 kD	1.087	88.889	SESDSS	96.617	>99.9%
2	71 to 76	AERD.EI	8.8 kD	1.3 kD	0.009	48.148	SESDAV	0.451	>95%
3	74 to 79	DEIL.GA	9.2 kD	0.9 kD	0.005	51.613	DEVDGV	0.264	>95%
4	9 to 14	RDPD.IN	1.5 kD	8.6 kD	0.001	57.143	NDPDMR	0.070	>95%

>PSB8_
MOUS
E_

rank	position	site	N fragment	C fragment	frequency score	similarity maxscore	similarity maxsite	average score	specificity
1	263 to 268	DVSD.LL	29.1 kD	1.1 kD	0.322	67.857	DVLDVL	21.837	>99%
2	18 to 23	AALD.AG	2.2 kD	28.0 kD	0.230	89.286	AAVDAG	20.530	>99%
3	86 to 91	VAVD.SR	9.4 kD	20.8 kD	0.283	60.714	TAVDAK	17.188	>99%
4	260 to 265	ESSD.VS	28.8 kD	1.4 kD	0.072	82.143	ESQDVS	5.891	>99%
5	209 to 214	GVMD.SG	23.0 kD	7.2 kD	0.080	66.667	EVMDAG	5.366	>99%
6	223 to 228	EAYD.LG	24.6 kD	5.6 kD	0.039	66.667	DAADLG	2.572	>95%
7	184 to 189	YYVD.DN	20.4 kD	9.8 kD	0.008	47.368	YWIDGS	0.366	>95%
8	236 to 241	THRD.NY	26.1 kD	4.1 kD	0.005	63.636	TEVDNY	0.305	>95%
9	185 to 190	YVDD.NG	20.5 kD	9.7 kD	0.004	60.000	LVIDNG	0.242	>95%
10	2 to 7	ALLD.LC	0.5 kD	29.7 kD	0.004	51.515	AVVDGC	0.211	>95%
11	120 to 125	CAAD.CQ	13.0 kD	17.2 kD	0.003	41.026	TCADCG	0.127	>95%
12	26 to 31	GRSD.PG	2.9 kD	27.3 kD	0.001	58.065	GSSDPL	0.077	>95%
13	251 to 256	MKED.GW	27.8 kD	2.4 kD	0.002	44.828	VKVDGS	0.072	>95%

>SUMF
1_MO
USE_

rank	position	site	N fragment	C fragment	frequency score	similarity maxscore	similarity maxsite	average score	specificity
1	190 to 195	EGPD.SS	20.5 kD	20.1 kD	0.514	67.742	EEPDSA	34.818	>99%
2	117 to 122	VTVD.GF	12.3 kD	28.4 kD	0.170	75.000	VSVDAF	12.786	>99%
3	350 to 355	NTPD.SS	38.7 kD	1.9 kD	0.135	75.862	SVPDSS	10.209	>99%
4	277 to 282	APVD.AF	30.3 kD	10.3 kD	0.017	67.857	VSVDAF	1.141	>95%
5	99 to 104	MGTD.DP	10.3 kD	30.4 kD	0.014	58.065	LGTDSD	0.835	>95%
6	268 to 273	TGED.GF	29.4 kD	11.2 kD	0.017	48.485	DGTDGL	0.824	>95%
7	208 to 213	SWND.AV	22.6 kD	18.0 kD	0.009	59.259	SESDAV	0.548	>95%
8	30 to 35	AGSD.EA	3.5 kD	37.1 kD	0.007	50.000	SGVDAA	0.330	>95%
9	363 to 368	CAAD.HL	40.0 kD	0.6 kD	0.005	50.000	CEVDAL	0.241	>95%
10	33 to 38	DEAE.AR	3.8 kD	36.8 kD	0.004	54.545	DEPDSR	0.227	>95%
11	122 to 127	FYMD.AY	12.9 kD	27.7 kD	0.003	66.667	FYSDSF	0.214	>95%
12	130 to 135	SNAD.FE	13.7 kD	26.9 kD	0.004	44.118	DDADYK	0.183	>95%
13	321 to 326	SGKD.RV	35.3 kD	5.3 kD	0.004	46.429	SGVDAA	0.163	>95%
14	106 to 111	IRQD.GE	11.1 kD	29.5 kD	0.003	54.839	AMEDGE	0.138	>95%
15	36 to 41	EARE.GA	4.2 kD	36.4 kD	0.001	58.065	EERDGS	0.058	>95%

>TPP1_MOUSE_

rank	position	site	N fragment	C fragment	frequency score	similarity maxscore	similarity maxsite	average score	specificity
1	326 to 331	DDED.SL	36.3 kD	25.0 kD	1.669	70.000	DEEDIL	116.813	>99.9%
2	401 to 406	EVVD.YI	44.3 kD	17.0 kD	0.101	62.963	ELVDSV	6.364	>99%
3	453 to 458	ALSD.GY	49.9 kD	11.4 kD	0.100	54.545	SETDGY	5.453	>99%
4	81 to 86	TLED.VA	9.4 kD	51.9 kD	0.032	62.963	TIADLA	2.023	>95%
5	447 to 452	AYPD.VA	49.3 kD	12.0 kD	0.030	62.069	IVPDIA	1.851	>95%
6	272 to 277	ASLD.VE	30.2 kD	31.1 kD	0.025	55.556	SSLDAR	1.369	>95%
7	528 to 533	EEVE.GQ	58.1 kD	3.2 kD	0.014	74.194	EEVDGK	1.069	>95%
8	356 to 361	ASGD.TG	39.5 kD	21.8 kD	0.015	53.125	DAGDVG	0.801	>95%
9	397 to 402	LITD.EV	43.9 kD	17.4 kD	0.011	59.259	SVTDSV	0.666	>95%
10	165 to 170	PHVD.FV	18.6 kD	42.7 kD	0.009	53.125	DGVDFV	0.471	>95%
11	67 to 72	AVSD.PS	7.8 kD	53.5 kD	0.009	45.161	VMRDPA	0.400	>95%
12	23 to 28	PEPD.QR	2.7 kD	58.6 kD	0.006	66.667	DEPDSR	0.380	>95%
13	211 to 216	TAKD.VG	23.7 kD	37.6 kD	0.006	53.125	DAGDVG	0.313	>95%
14	115 to 120	TTQD.FL	13.0 kD	48.3 kD	0.007	37.931	LSSDFT	0.281	>95%
15	513 to 518	GLFD.VT	56.4 kD	4.9 kD	0.003	67.742	DLFDLT	0.224	>95%
16	323 to 328	SYGD.DE	35.9 kD	25.4 kD	0.004	42.424	MQGDGE	0.177	>95%
17	39 to 44	GRVD.PE	4.6 kD	56.7 kD	0.003	59.375	GLLDPK	0.160	>95%
18	168 to 173	DFVG.GL	18.9 kD	42.4 kD	0.001	77.419	DFVEGL	0.080	>95%

>ZC3H
3_MO
USE_

rank	position	site	N fragment	C fragment	frequency score	similarity maxscore	similarity maxsite	average score	specificity
1	82 to 87	ESSD.PA	9.7 kD	93.5 kD	0.047	58.065	GSSDPL	2.714	>95%
2	704 to 709	KKTD.GS	77.9 kD	25.3 kD	0.038	66.667	TSTDGS	2.519	>95%
3	183 to 188	YSVD.DP	20.5 kD	82.7 kD	0.041	58.621	VSLDSP	2.395	>95%
4	809 to 814	GPSD.GA	89.5 kD	13.7 kD	0.039	57.143	GDSDGP	2.245	>95%
5	207 to 212	RVSD.SS	23.1 kD	80.1 kD	0.018	55.556	SESDSS	1.018	>95%
6	772 to 777	LCPD.FA	85.4 kD	17.8 kD	0.016	62.069	IVPDIA	1.017	>95%
7	16 to 21	GLID.DY	2.4 kD	100.9 kD	0.013	63.636	GFLDSY	0.808	>95%
8	97 to 102	RSED.SQ	11.3 kD	91.9 kD	0.010	51.724	EQEDSS	0.499	>95%
9	412 to 417	TSGD.RP	45.2 kD	58.0 kD	0.008	48.485	DSTDKP	0.409	>95%
10	630 to 635	GRLD.PA	69.3 kD	33.9 kD	0.006	62.500	GLLDPK	0.352	>95%
11	184 to 189	SVDD.PL	20.6 kD	82.6 kD	0.007	50.000	GVEDTL	0.342	>95%
12	750 to 755	VCSD.FL	83.0 kD	20.3 kD	0.005	51.724	LSSDFT	0.284	>95%
13	117 to 122	LSPD.QN	13.7 kD	89.5 kD	0.006	44.828	LSVDRG	0.268	>95%
14	936 to 941	RTKD.SG	102.0 kD	1.2 kD	0.004	58.824	DTKDGG	0.233	>95%
15	86 to 91	PASD.PA	10.1 kD	93.1 kD	0.003	55.172	PAADAI	0.155	>95%
16	147 to 152	EGCD.DP	16.7 kD	86.5 kD	0.003	54.286	DGLDGP	0.148	>95%
17	17 to 22	LIDD.YK	2.5 kD	100.7 kD	0.002	48.485	DVTDYK	0.120	>95%
18	162 to 167	SEVE.VP	18.4 kD	84.8 kD	0.001	59.375	GQVDVP	0.089	>95%
19	164 to 169	VEVP.GG	18.6 kD	84.6 kD	0.001	62.069	VEVDSG	0.073	>95%
20	350 to 355	PKVD.PE	38.7 kD	64.5 kD	0.002	47.059	NKTDPR	0.072	>95%

>HNRPL_MOUSE_

rank	position	site	N fragment	C fragment	frequency score	similarity maxscore	similarity maxsite	average score	specificity
1	181 to 186	DSDD.SR	19.3 kD	44.6 kD	0.383	68.966	DSSDSE	26.383	>99%
2	233 to 238	VEFD.SV	25.1 kD	38.8 kD	0.348	65.625	VDFDDI	22.868	>99%
3	105 to 110	GLID.GV	11.1 kD	52.9 kD	0.236	58.065	YLLDGI	13.727	>99%
4	463 to 468	GLED.GS	50.4 kD	13.6 kD	0.179	61.290	DLRDGS	10.965	>99%
5	422 to 427	EMAD.GY	45.9 kD	18.1 kD	0.122	63.636	EELDGY	7.773	>99%
6	111 to 116	VEAD.LV	11.7 kD	52.3 kD	0.118	51.852	VEVDAA	6.125	>99%
7	548 to 553	SKSD.AL	60.0 kD	3.9 kD	0.059	74.074	SESDAV	4.372	>99%
8	427 to 432	YAVD.RA	46.4 kD	17.6 kD	0.028	51.724	LSVDRG	1.453	>95%
9	180 to 185	GDSD.DS	19.2 kD	44.8 kD	0.019	62.069	TDSDSS	1.177	>95%
10	374 to 379	PHAD.SP	40.5 kD	23.5 kD	0.016	71.053	PHLDGP	1.154	>95%
11	114 to 119	DLVE.AL	12.0 kD	52.0 kD	0.016	64.286	DAIDAL	1.022	>95%
12	140 to 145	EFED.VL	15.0 kD	49.0 kD	0.012	63.333	DFVDIL	0.784	>95%
13	203 to 208	ITTD.VL	21.7 kD	42.2 kD	0.014	55.172	IETDAM	0.746	>95%
14	470 to 475	SYKD.FS	51.1 kD	12.8 kD	0.009	65.714	SYNDFG	0.620	>95%
15	87 to 92	ENYD.DP	9.1 kD	54.8 kD	0.006	54.839	ERVDSP	0.303	>95%
16	236 to 241	DSVQ.SA	25.4 kD	38.5 kD	0.005	61.538	SSVDSA	0.302	>95%
17	153 to 158	YAAD.NQ	16.2 kD	47.7 kD	0.005	50.000	ESLDNQ	0.248	>95%
18	368 to 373	PPPD.YG	39.9 kD	24.1 kD	0.003	68.571	AEPDYG	0.210	>95%
19	306 to 311	LLGD.HP	33.1 kD	30.8 kD	0.004	43.333	VLGDGV	0.193	>95%
20	20 to 25	QQPD.EQ	3.0 kD	61.0 kD	0.003	53.125	DQPDSA	0.181	>95%

>ODPA
_MOU
SE_

rank	position	site	N fragment	C fragment	frequency score	similarity maxscore	similarity maxsite	average score	specificity
1	345 to 350	EIED.AA	38.5 kD	4.7 kD	0.133	62.069	EQEDAS	8.268	>99%
2	252 to 257	LRVD.GM	27.9 kD	15.3 kD	0.060	62.500	NKVDGM	3.736	>95%
3	193 to 198	LYGD.GA	21.3 kD	21.9 kD	0.060	60.000	VLGDGV	3.625	>95%
4	238 to 243	ASTD.YY	26.2 kD	17.0 kD	0.047	63.636	SMTDFY	3.001	>95%
5	255 to 260	DGMD.IL	28.2 kD	15.0 kD	0.038	64.286	DVLDVL	2.470	>95%
6	57 to 62	TRED.GL	6.6 kD	36.6 kD	0.040	58.621	TVADGL	2.324	>95%
7	293 to 298	SMSD.PG	32.5 kD	10.7 kD	0.009	48.387	VMRDPA	0.439	>95%
8	336 to 341	KEID.VE	37.4 kD	5.8 kD	0.006	62.069	EEIDAQ	0.382	>95%
9	109 to 114	NPTD.HL	12.6 kD	30.7 kD	0.003	56.250	NPQDSV	0.183	>95%
10	92 to 97	HLCD.GQ	10.9 kD	32.3 kD	0.003	55.172	SVIDGQ	0.162	>95%
11	369 to 374	YSSD.PP	41.1 kD	2.1 kD	0.003	48.387	GSSDPL	0.132	>95%
12	353 to 358	ATAD.PE	39.3 kD	3.9 kD	0.002	42.857	IEADSE	0.103	>95%
13	30 to 35	FAND.AT	3.5 kD	39.7 kD	0.002	40.741	MAVDAV	0.097	>95%
14	312 to 317	SKSD.PI	34.7 kD	8.5 kD	0.001	62.963	SESDAV	0.093	>95%
15	319 to 324	LLKD.RM	35.5 kD	7.7 kD	0.001	51.724	TLRDSM	0.071	>95%
16	76 to 81	LKAD.QL	9.0 kD	34.3 kD	0.002	46.429	LDVDSL	0.070	>95%

>BCL7
A_MO
USE_

rank	position	site	N fragment	C fragment	frequency score	similarity maxscore	similarity maxsite	average score	specificity
1	135 to 140	AQAD.GK	15.2 kD	7.6 kD	0.155	63.333	AQMDGA	9.817	>99%
2	98 to 103	SIAD.AS	11.4 kD	11.3 kD	0.136	58.621	SVPDSS	7.995	>99%
3	144 to 149	GAED.AS	16.1 kD	6.7 kD	0.050	55.172	EQEDAS	2.768	>95%
4	88 to 93	DMHD.DN	10.4 kD	12.4 kD	0.019	72.222	DEHDEN	1.352	>95%
5	123 to 128	VPSD.GT	14.0 kD	8.8 kD	0.013	67.857	VASDGV	0.910	>95%
6	37 to 42	TVGD.TS	4.7 kD	18.1 kD	0.016	53.571	TLTDSS	0.834	>95%
7	54 to 59	PKVD.DK	6.7 kD	16.1 kD	0.008	50.000	DEVDNK	0.406	>95%
8	185 to 190	VSQD.LE	20.3 kD	2.4 kD	0.007	50.000	ESQDVS	0.366	>95%
9	130 to 135	AKAD.EA	14.6 kD	8.1 kD	0.005	51.852	AEVDAA	0.282	>95%
10	126 to 131	DGTE.AK	14.3 kD	8.5 kD	0.003	58.621	DSVDAK	0.168	>95%
11	89 to 94	MHDD.NS	10.5 kD	12.2 kD	0.003	54.839	MDVDNS	0.147	>95%

>CLIC1_MOUSE_

rank	position	site	N fragment	C fragment	frequency score	similarity maxscore	similarity maxsite	average score	specificity
1	150 to 155	EEVD.ET	17.0 kD	10.0 kD	0.263	76.667	DEVDES	20.165	>99%
2	44 to 49	TTVD.TK	5.2 kD	21.8 kD	0.101	71.429	TAVDAK	7.243	>99%
3	14 to 19	AGSD.GA	1.9 kD	25.1 kD	0.124	57.143	VASDGV	7.108	>99%
4	156 to 161	SAED.EG	17.6 kD	9.4 kD	0.018	63.333	SATDRG	1.116	>95%
5	73 to 78	VHTD.TN	8.5 kD	18.5 kD	0.013	65.625	LHTDSR	0.883	>95%
6	166 to 171	KFLD.GN	18.8 kD	8.2 kD	0.014	57.143	SLLDSN	0.784	>95%
7	124 to 129	ALND.NL	14.1 kD	12.9 kD	0.014	56.250	DLNDGL	0.766	>95%
8	174 to 179	TLAD.CN	19.6 kD	7.4 kD	0.011	58.974	TCADCG	0.628	>95%
9	106 to 111	SGLD.IF	12.1 kD	14.9 kD	0.007	60.000	SGVDIG	0.429	>95%
10	138 to 143	KVLD.NY	15.6 kD	11.4 kD	0.006	60.000	DVLDNV	0.371	>95%
11	222 to 227	TCPD.DE	25.1 kD	1.9 kD	0.007	51.515	ECVDSE	0.341	>95%
12	153 to 158	DETS.AE	17.3 kD	9.7 kD	0.001	66.667	DETDAS	0.089	>95%
13	227 to 232	EEIE.LA	25.8 kD	1.2 kD	0.001	58.621	DEVDVA	0.085	>95%

>TRXR1_MOUSE_

rank	position	site	N fragment	C fragment	frequency score	similarity maxscore	similarity maxsite	average score	specificity
1	88 to 93	STPD.SR	9.9 kD	57.0 kD	0.218	68.966	SVPDSS	15.026	>99%
2	577 to 582	QQLD.ST	63.7 kD	3.2 kD	0.043	64.286	EELDSA	2.750	>95%
3	153 to 158	LVLD.FV	16.8 kD	50.1 kD	0.038	44.828	IVLDGT	1.693	>95%
4	193 to 198	ALKD.SR	21.0 kD	46.0 kD	0.022	58.621	VLKDTQ	1.298	>95%
5	292 to 297	ISSD.DL	32.4 kD	34.5 kD	0.009	50.000	VASDGV	0.458	>95%
6	479 to 484	CDYD.NV	52.7 kD	14.2 kD	0.007	56.250	VDFDDI	0.419	>95%
7	293 to 298	SSDD.LF	32.6 kD	34.4 kD	0.006	53.571	ALDDLI	0.342	>95%
8	448 to 453	DILE.GK	49.4 kD	17.5 kD	0.005	59.375	DTVDGK	0.307	>95%
9	123 to 128	GSYD.FD	13.8 kD	53.1 kD	0.005	52.941	GSYDSY	0.240	>95%
10	431 to 436	PVTD.EE	47.5 kD	19.5 kD	0.005	50.000	DVTDAQ	0.225	>95%
11	602 to 607	SGGD.IL	66.3 kD	0.7 kD	0.005	43.333	SGVDIG	0.214	>95%
12	528 to 533	PSRD.NN	58.4 kD	8.5 kD	0.004	43.750	DTRDNV	0.188	>95%
13	117 to 122	GSKD.PP	13.2 kD	53.7 kD	0.002	64.516	GSSDPL	0.146	>95%
14	203 to 208	KVED.TV	22.2 kD	44.7 kD	0.002	63.333	GVEDTL	0.142	>95%
15	326 to 331	IGLD.VT	35.9 kD	31.0 kD	0.003	44.828	IVLDGT	0.118	>95%
16	445 to 450	AIGD.IL	49.1 kD	17.9 kD	0.002	53.571	ALDDLI	0.105	>95%
17	405 to 410	VGRD.SC	44.7 kD	22.2 kD	0.002	41.935	LGTDSD	0.105	>95%
18	342 to 347	FDQD.MA	37.7 kD	29.2 kD	0.002	56.667	FIQDRA	0.089	>95%
19	542 to 547	NLKD.DE	60.0 kD	6.9 kD	0.001	62.500	DLRDDK	0.088	>95%
20	125 to 130	YDFD.LI	14.1 kD	52.9 kD	0.001	53.125	VDFDDI	0.077	>95%

>SYCE
2_MO
USE_

rank	position	site	N fragment	C fragment	frequency score	similarity maxscore	similarity maxsite	average score	specificity
1	53 to 58	SSLD.SS	5.9 kD	13.6 kD	1.905	76.923	SSVDSA	146.530	>99.9%
2	86 to 91	NFRD.SL	9.8 kD	9.8 kD	0.020	58.621	TLRDSM	1.167	>95%
3	165 to 170	NYKD.GE	19.3 kD	0.3 kD	0.006	54.545	DYADGA	0.335	>95%
4	34 to 39	EETP.GS	4.1 kD	15.4 kD	0.002	58.065	EETDGI	0.113	>95%
5	12 to 17	ELKD.QE	1.6 kD	17.9 kD	0.002	48.276	DLVDAE	0.111	>95%
6	94 to 99	KVSD.LT	10.7 kD	8.9 kD	0.001	64.286	KESDLS	0.091	>95%

>CENP
F_MO
USE_

rank	position	site	N fragment	C fragment	frequency score	similarity maxscore	similarity maxsite	average score	specificity
1	2713 to 2718	SLVD.SN	311.9 kD	30.6 kD	1.125	89.286	SLLDSN	100.445	>99.9%
2	1129 to 1134	SATD.GL	130.8 kD	211.6 kD	1.312	63.636	DGTDGL	83.479	>99.9%
3	1965 to 1970	EELD.NV	225.6 kD	116.8 kD	0.886	75.000	EELDSA	66.445	>99.9%
4	820 to 825	CSVD.GG	95.4 kD	247.0 kD	0.839	76.667	AAVDGG	64.286	>99.9%
5	676 to 681	SMLD.SK	79.0 kD	263.4 kD	0.396	71.429	SLLDSN	28.264	>99%
6	1805 to 1810	MEAD.SK	207.0 kD	135.5 kD	0.358	75.000	IEADSE	26.841	>99%
7	2250 to 2255	MEED.SK	258.4 kD	84.0 kD	0.234	70.968	DEEDSK	16.607	>99%
8	1862 to 1867	SEED.LG	213.6 kD	128.9 kD	0.241	58.065	DEEDLQ	14.016	>99%
9	1440 to 1445	SYVD.TL	166.2 kD	176.2 kD	0.176	65.385	SSVDSL	11.476	>99%
10	649 to 654	VSPD.SN	75.7 kD	266.7 kD	0.228	48.387	DSPDSV	11.026	>99%
11	2937 to 2942	SSAD.SH	336.4 kD	6.0 kD	0.150	61.538	SSVDSA	9.210	>99%
12	1647 to 1652	QELD.LS	189.3 kD	153.1 kD	0.122	66.667	SELDAS	8.126	>99%
13	2227 to 2232	SELD.ER	255.6 kD	86.8 kD	0.118	66.667	SSLDAR	7.894	>99%
14	1385 to 1390	TPLD.SS	159.8 kD	182.6 kD	0.126	57.143	DALDSS	7.220	>99%
15	39 to 44	FQLD.SL	5.1 kD	337.4 kD	0.094	67.857	SQLDSM	6.404	>99%
16	1206 to 1211	ISLD.SY	139.6 kD	202.8 kD	0.099	64.286	VSVDAF	6.378	>99%
17	223 to 228	LSSD.AL	26.2 kD	316.2 kD	0.102	51.724	IETDAM	5.297	>99%
18	894 to 899	LQED.AA	103.8 kD	238.7 kD	0.080	62.069	EQEDAS	4.941	>99%
19	2173 to 2178	AILD.SE	249.3 kD	93.1 kD	0.074	60.714	SLLDSN	4.505	>99%
20	2147 to 2152	RESD.SL	246.3 kD	96.2 kD	0.062	62.069	EEADSM	3.863	>99%

>LC7L2_MOUSE_

rank	position	site	N fragment	C fragment	frequency score	similarity maxscore	similarity maxsite	average score	specificity
1	17 to 22	TSRD.GD	2.2 kD	44.3 kD	0.111	66.667	TSTDGS	7.400	>99%
2	82 to 87	FELD.AM	9.7 kD	36.9 kD	0.088	65.517	IETDAM	5.740	>99%
3	85 to 90	DAMD.HL	10.0 kD	36.5 kD	0.069	67.857	DAIDAL	4.693	>99%
4	322 to 327	SSRD.RS	38.4 kD	8.2 kD	0.042	50.000	SATDRG	2.111	>95%
5	353 to 358	SSRD.RS	42.3 kD	4.3 kD	0.042	50.000	SATDRG	2.111	>95%
6	9 to 14	AMLD.QL	1.3 kD	45.2 kD	0.006	70.370	SLLDEL	0.442	>95%
7	157 to 162	DEVE.KA	18.1 kD	28.5 kD	0.006	67.742	DEVDKM	0.426	>95%
8	363 to 368	DRKD.KK	43.6 kD	3.0 kD	0.005	58.065	DEEDSK	0.263	>95%
9	346 to 351	ASRD.RD	41.4 kD	5.2 kD	0.005	44.118	GEDDRD	0.237	>95%
10	52 to 57	TRMD.LG	6.2 kD	40.4 kD	0.005	46.875	TELDGG	0.222	>95%
11	198 to 203	GLHD.ND	22.8 kD	23.8 kD	0.005	41.935	DVSDNE	0.203	>95%
12	61 to 66	KVHD.LA	7.2 kD	39.4 kD	0.003	57.576	EVHDGA	0.150	>95%
13	385 to 390	EERE.AG	46.2 kD	0.4 kD	0.002	77.419	EERDSG	0.140	>95%
14	341 to 346	RDQD.LA	40.9 kD	5.7 kD	0.002	53.571	KESDLS	0.112	>95%
15	67 to 72	LRAD.YE	7.8 kD	38.7 kD	0.002	53.571	IEADSE	0.096	>95%
16	358 to 363	SPRD.RD	42.9 kD	3.7 kD	0.002	41.176	GEDDRD	0.068	>95%
17	30 to 35	FSDD.RV	3.7 kD	42.8 kD	0.001	50.000	FIQDRA	0.068	>95%
18	348 to 353	RDRD.RS	41.7 kD	4.9 kD	0.001	48.387	DDRDSA	0.066	>95%
19	76 to 81	KEQD.FF	8.9 kD	37.7 kD	0.001	50.000	KESDLS	0.053	>95%

>SRSF 7_MO USE_

rank	position	site	N fragment	C fragment	frequency score	similarity maxscore	similarity maxsite	average score	specificity
1	88 to 93	DAED.AV	10.3 kD	20.5 kD	0.570	64.286	DIADAV	36.648	>99%
2	85 to 90	DPRD.AE	10.0 kD	20.8 kD	0.062	65.625	DPSDSQ	4.044	>99%
3	94 to 99	RGLD.GK	11.0 kD	19.8 kD	0.021	63.333	QSVDGK	1.336	>95%
4	118 to 123	SRFD.RP	13.6 kD	17.2 kD	0.003	51.613	ERVDSP	0.166	>95%
5	91 to 96	DAVR.GL	10.7 kD	20.1 kD	0.002	63.333	DAVDGV	0.135	>95%
6	82 to 87	EFED.PR	9.7 kD	21.1 kD	0.002	55.882	DFQDGR	0.131	>95%

>HMG B2_MO USE_

rank	position	site	N fragment	C fragment	frequency score	similarity maxscore	similarity maxsite	average score	specificity
1	198 to 203	EEED.DE	23.0 kD	1.1 kD	0.070	75.000	DEEDDS	5.284	>99%
2	205 to 210	EEED.EE	23.9 kD	0.3 kD	0.038	62.500	EEEDGK	2.348	>95%
3	30 to 35	KHPD.SS	3.8 kD	20.3 kD	0.022	64.706	DYPDSS	1.451	>95%
4	199 to 204	EEDD.EE	23.1 kD	1.0 kD	0.018	65.714	DEDDDD	1.162	>95%
5	121 to 126	SIGD.TA	14.2 kD	9.9 kD	0.016	51.852	TIADLA	0.832	>95%
6	155 to 160	YEKD.IA	18.2 kD	5.9 kD	0.008	44.828	DEVDVA	0.345	>95%
7	64 to 69	AKSD.KA	7.7 kD	16.4 kD	0.003	58.621	VETDKA	0.161	>95%
8	137 to 142	SAKD.KQ	16.0 kD	8.1 kD	0.002	43.333	SATDRG	0.096	>95%
9	59 to 64	KFED.LA	7.2 kD	16.9 kD	0.002	46.875	DYKDIA	0.086	>95%
10	186 to 191	EPED.EE	21.5 kD	2.7 kD	0.002	53.125	DPSDSQ	0.081	>95%

>PPID_
MOUS
E_

rank	position	site	N fragment	C fragment	frequency score	similarity maxscore	similarity maxsite	average score	specificity
1	140 to 145	PHLD.GK	15.6 kD	25.1 kD	0.051	78.947	PHLDGP	3.998	>99%
2	251 to 256	RYVD.SS	27.9 kD	12.8 kD	0.060	54.839	SYLDSG	3.272	>95%
3	209 to 214	ADID.LK	22.9 kD	17.8 kD	0.035	54.839	ADIDGQ	1.929	>95%
4	201 to 206	SHPD.FP	22.0 kD	18.7 kD	0.030	61.765	SFPDSP	1.831	>95%
5	212 to 217	DLKD.VD	23.3 kD	17.4 kD	0.032	55.172	DLVDAE	1.786	>95%
6	326 to 331	ALAD.LK	36.1 kD	4.6 kD	0.031	55.556	TIADLA	1.730	>95%
7	300 to 305	LEMD.PS	33.2 kD	7.5 kD	0.021	61.290	VEVDPM	1.270	>95%
8	20 to 25	FDVD.IG	2.5 kD	38.2 kD	0.022	58.621	LEVDAG	1.262	>95%
9	261 to 266	EKAD.RS	28.9 kD	11.8 kD	0.015	65.517	EEADTS	0.989	>95%
10	207 to 212	EDAD.ID	22.7 kD	18.0 kD	0.017	55.172	EEADTS	0.916	>95%
11	197 to 202	GSGD.SH	21.6 kD	19.1 kD	0.016	55.882	GSYDSY	0.905	>95%
12	291 to 296	GAID.SC	32.2 kD	8.5 kD	0.006	66.667	DSVDSC	0.409	>95%
13	222 to 227	ISED.LK	24.4 kD	16.3 kD	0.008	41.935	DEEDLQ	0.350	>95%
14	193 to 198	FPKD.GS	21.3 kD	19.4 kD	0.003	53.125	FPADEA	0.178	>95%
15	321 to 326	KEYD.QA	35.6 kD	5.1 kD	0.003	55.172	KRIDQS	0.175	>95%
16	36 to 41	LFAD.IV	4.2 kD	36.5 kD	0.004	43.333	DFVDIL	0.156	>95%
17	214 to 219	KDVD.KI	23.5 kD	17.2 kD	0.002	57.143	LDVDSL	0.101	>95%
18	186 to 191	EGDD.WG	20.4 kD	20.3 kD	0.002	47.059	DGDDDA	0.093	>95%
19	205 to 210	FPED.AD	22.5 kD	18.2 kD	0.001	46.875	FPADEA	0.056	>95%
20	18 to 23	VFFD.VD	2.3 kD	38.5 kD	0.002	34.375	DLFDIG	0.052	>95%

>SIAE_
MOUS
E_

rank	position	site	N fragment	C fragment	frequency score	similarity maxscore	similarity maxsite	average score	specificity
1	170 to 175	TEVD.LS	18.9 kD	41.9 kD	1.024	71.429	TEVDAA	73.138	>99.9%
2	164 to 169	EELD.DL	18.2 kD	42.5 kD	0.673	68.750	DETDDL	46.298	>99%
3	142 to 147	ELSD.TA	15.7 kD	45.0 kD	0.126	58.621	DLTDAA	7.381	>99%
4	393 to 398	CDRD.SP	44.5 kD	16.2 kD	0.050	71.429	CSTDSP	3.604	>95%
5	30 to 35	SYID.NY	3.5 kD	57.3 kD	0.028	63.636	TEVDNY	1.786	>95%
6	356 to 361	NSSD.YG	40.2 kD	20.5 kD	0.016	60.606	DESDFG	0.954	>95%
7	165 to 170	ELDD.LT	18.3 kD	42.4 kD	0.012	64.286	ALDDLI	0.798	>95%
8	391 to 396	DLCD.RD	44.2 kD	16.5 kD	0.012	59.375	DEVDRD	0.686	>95%
9	490 to 495	LILD.LN	55.4 kD	5.3 kD	0.012	50.000	SLLDSN	0.594	>95%
10	84 to 89	VVLD.PM	9.3 kD	51.4 kD	0.008	67.742	VEVDPM	0.537	>95%
11	336 to 341	GQTD.RF	37.9 kD	22.9 kD	0.006	54.545	GQKDSY	0.318	>95%
12	369 to 374	QTAD.FG	41.8 kD	18.9 kD	0.005	54.545	DLADYG	0.286	>95%
13	306 to 311	SNAD.YN	34.2 kD	26.6 kD	0.005	52.941	DDADYK	0.282	>95%
14	202 to 207	YLYD.TL	22.5 kD	38.2 kD	0.005	51.613	YLLDGI	0.271	>95%
15	396 to 401	DSPF.GS	44.8 kD	15.9 kD	0.002	58.824	DSPDGQ	0.112	>95%
16	458 to 463	QMQD.NK	51.8 kD	9.0 kD	0.002	68.750	DMQDNS	0.106	>95%
17	388 to 393	VAID.LC	43.9 kD	16.9 kD	0.002	44.444	MAVDAV	0.068	>95%

>ARSA_MOUSE_

rank	position	site	N fragment	C fragment	frequency score	similarity maxscore	similarity maxsite	average score	specificity
1	251 to 256	MELD.GA	27.2 kD	26.6 kD	2.199	75.758	MELDGP	166.626	>99.9%
2	353 to 358	DGVD.IS	37.5 kD	16.2 kD	0.463	77.419	DGVDLK	35.876	>99%
3	350 to 355	VTLD.GV	37.3 kD	16.5 kD	0.362	68.966	ATIDGL	24.935	>99%
4	331 to 336	SSLD.LL	35.4 kD	18.3 kD	0.259	65.385	SSVDSL	16.964	>99%
5	463 to 468	AQYD.AA	49.6 kD	4.1 kD	0.068	73.333	TQFDAA	4.989	>99%
6	207 to 212	LMAD.AQ	22.1 kD	31.7 kD	0.022	53.333	LMVDGK	1.196	>95%
7	263 to 268	TVGD.LG	28.2 kD	25.5 kD	0.016	66.667	TIADLA	1.091	>95%
8	426 to 431	LLYD.LS	45.6 kD	8.2 kD	0.021	45.161	DLFDLT	0.927	>95%
9	407 to 412	TTSD.PA	43.5 kD	10.2 kD	0.011	48.387	VMRDPA	0.524	>95%
10	277 to 282	FTAD.NG	29.7 kD	24.0 kD	0.007	56.667	LVIDNG	0.424	>95%
11	245 to 250	PFGD.SL	26.5 kD	27.3 kD	0.007	51.613	DEGDSL	0.363	>95%
12	31 to 36	GYGD.LG	3.4 kD	50.3 kD	0.005	54.286	SYNDFG	0.278	>95%
13	430 to 435	LSQD.PG	46.0 kD	7.7 kD	0.005	55.172	LSVDRG	0.276	>95%
14	380 to 385	DEIH.GV	40.6 kD	13.2 kD	0.003	74.194	DEVDGV	0.193	>95%
15	403 to 408	AHSD.TT	43.1 kD	10.7 kD	0.003	50.000	SHTDQS	0.138	>95%
16	25 to 30	IFAD.DL	2.8 kD	50.9 kD	0.003	42.857	VVADGV	0.129	>95%
17	442 to 447	ESIE.GV	47.3 kD	6.4 kD	0.002	60.000	DAVDGV	0.110	>95%
18	26 to 31	FADD.LG	2.9 kD	50.8 kD	0.002	50.000	DAADLG	0.108	>95%
19	203 to 208	FSRD.LM	21.6 kD	32.1 kD	0.002	35.484	DQRDLT	0.078	>95%
20	57 to 62	RFTD.FY	6.1 kD	47.6 kD	0.001	69.697	SMTDFY	0.076	>95%

>KAT3_MOUSE_

rank	position	site	N fragment	C fragment	frequency score	similarity maxscore	similarity maxsite	average score	specificity
1	438 to 443	STLD.AA	49.5 kD	1.6 kD	0.711	67.857	SSLDGA	48.259	>99%
2	47 to 52	EGLD.SN	5.5 kD	45.6 kD	0.227	60.714	DALDSS	13.764	>99%
3	366 to 371	IVPD.GG	41.3 kD	9.8 kD	0.185	62.500	TVTDGG	11.554	>99%
4	145 to 150	GLVD.PG	16.2 kD	34.9 kD	0.050	68.750	GLLDPK	3.428	>95%
5	384 to 389	DLSD.MN	43.1 kD	8.0 kD	0.051	60.606	DLPDMK	3.106	>95%
6	381 to 386	LGAD.LS	42.8 kD	8.3 kD	0.014	45.161	LGTDSD	0.632	>95%
7	148 to 153	DPGD.EV	16.5 kD	34.6 kD	0.008	64.516	DTGDAV	0.502	>95%
8	72 to 77	GFPD.IS	8.2 kD	42.9 kD	0.009	50.000	DYPDSS	0.463	>95%
9	415 to 420	AFCD.SK	46.8 kD	4.4 kD	0.006	65.714	DFCDSS	0.412	>95%
10	60 to 65	LAAD.PS	7.0 kD	44.1 kD	0.008	44.444	AAVDTS	0.336	>95%
11	182 to 187	KPTD.GM	20.4 kD	30.7 kD	0.006	58.065	ETTDGV	0.329	>95%
12	392 to 397	EPYD.YK	44.0 kD	7.1 kD	0.005	60.000	DEYDYS	0.314	>95%
13	90 to 95	AFID.NM	10.2 kD	40.9 kD	0.005	48.485	DFLDNE	0.258	>95%
14	374 to 379	IIAD.VS	42.1 kD	9.0 kD	0.003	55.556	TIADLA	0.192	>95%
15	190 to 195	TSSD.WT	21.3 kD	29.9 kD	0.003	65.517	LSSDFT	0.169	>95%
16	238 to 243	VKHD.TL	26.8 kD	24.3 kD	0.003	38.710	TSHDAS	0.117	>95%
17	434 to 439	IKKD.ST	49.1 kD	2.0 kD	0.002	60.714	LKTDSS	0.113	>95%
18	244 to 249	CISD.EV	27.4 kD	23.7 kD	0.002	48.148	SVTDSV	0.076	>95%

>GSTO 1_MOUSE_

rank	position	site	N fragment	C fragment	frequency score	similarity maxscore	similarity maxsite	average score	specificity
1	170 to 175	SMVD.YL	19.6 kD	7.9 kD	0.131	57.692	SSVDSL	7.550	>99%
2	91 to 96	EYLD.EA	10.5 kD	17.0 kD	0.075	68.750	EYIDGA	5.142	>99%
3	228 to 233	YLQD.SP	26.5 kD	0.9 kD	0.023	68.571	YVPDSP	1.571	>95%
4	164 to 169	LGGD.SP	18.9 kD	8.5 kD	0.020	54.839	LGTDSD	1.092	>95%
5	102 to 107	LFPD.DP	11.8 kD	15.7 kD	0.012	70.588	SFPDSP	0.860	>95%
6	155 to 160	EGMD.NY	18.0 kD	9.5 kD	0.010	60.000	ESLDNQ	0.582	>95%
7	234 to 239	EACD.YG	27.1 kD	0.3 kD	0.008	60.606	DLADYG	0.459	>95%
8	214 to 219	HKID.AK	24.8 kD	2.7 kD	0.005	62.500	HNLDAS	0.315	>95%
9	135 to 140	RKED.SP	15.6 kD	11.8 kD	0.003	54.839	EEADSP	0.162	>95%
10	205 to 210	MQQD.PV	23.8 kD	3.6 kD	0.001	48.387	VEVDPM	0.065	>95%

>HOT_
MOUS
E_

rank	position	site	N fragment	C fragment	frequency score	similarity maxscore	similarity maxsite	average score	specificity
1	111 to 116	EPTD.GS	12.3 kD	37.6 kD	0.416	63.333	TSTDGS	26.326	>99%
2	450 to 455	SEED.LS	48.6 kD	1.4 kD	0.255	67.857	KESDLS	17.307	>99%
3	400 to 405	VLAD.AL	43.1 kD	6.8 kD	0.147	62.069	VVPDAL	9.146	>99%
4	157 to 162	EFLD.YV	17.1 kD	32.8 kD	0.055	56.667	DFLDAL	3.124	>95%
5	219 to 224	GLVD.PL	23.5 kD	26.4 kD	0.042	68.750	GLLDPK	2.911	>95%
6	413 to 418	NVDD.GL	44.6 kD	5.3 kD	0.032	62.069	TVADGL	2.004	>95%
7	90 to 95	IVMD.SL	10.0 kD	39.9 kD	0.032	55.172	VVPDAL	1.759	>95%
8	412 to 417	LNVD.DG	44.5 kD	5.4 kD	0.022	65.517	LSVDRG	1.473	>95%
9	127 to 132	GAFD.AY	14.1 kD	35.9 kD	0.014	70.588	GSYDSY	0.967	>95%
10	116 to 121	SFMD.AI	12.9 kD	37.0 kD	0.014	66.667	DFLDAL	0.915	>95%
11	237 to 242	SGFD.VL	25.4 kD	24.5 kD	0.010	50.000	SGVDAA	0.477	>95%
12	76 to 81	LMTD.KN	8.4 kD	41.5 kD	0.004	59.375	LQTDGN	0.214	>95%
13	39 to 44	KTAD.YA	4.4 kD	45.5 kD	0.004	48.387	SQRDYA	0.212	>95%
14	62 to 67	VGMD.LQ	6.9 kD	43.0 kD	0.004	48.387	DGVDLK	0.204	>95%
15	300 to 305	DDLE.AR	32.5 kD	17.5 kD	0.003	66.667	DDLDAL	0.178	>95%
16	346 to 351	YNVD.HP	37.3 kD	12.6 kD	0.004	46.667	VEVDAP	0.163	>95%
17	103 to 108	QVYD.DV	11.4 kD	38.5 kD	0.003	46.667	DVLDNV	0.133	>95%
18	393 to 398	RIQD.AG	42.4 kD	7.5 kD	0.002	63.333	EVMDAG	0.131	>95%
19	408 to 413	FLFD.LN	44.1 kD	5.8 kD	0.002	54.839	DLFDLT	0.112	>95%
20	424 to 429	SKDD.IP	45.7 kD	4.2 kD	0.003	43.750	GQVDVP	0.111	>95%

>AMP
L_MO
USE_

rank	position	site	N fragment	C fragment	frequency score	similarity maxscore	similarity maxsite	average score	specificity
1	361 to 366	DNTD.AE	38.9 kD	17.2 kD	0.334	70.000	DETDAS	23.389	>99%
2	370 to 375	ILAD.AL	39.9 kD	16.2 kD	0.047	58.621	VVPDAL	2.748	>95%
3	44 to 49	DKDD.DL	5.1 kD	51.0 kD	0.026	78.788	DEDDDV	2.070	>95%
4	284 to 289	ITFD.SG	31.1 kD	25.0 kD	0.026	58.621	VEVDSG	1.510	>95%
5	106 to 111	AGVD.DQ	11.7 kD	44.4 kD	0.026	51.613	ADIDGQ	1.363	>95%
6	358 to 363	IQVD.NT	38.6 kD	17.5 kD	0.020	58.065	MDVDNS	1.143	>95%
7	514 to 519	FSKD.SS	55.9 kD	0.2 kD	0.021	53.333	DEKDSS	1.140	>95%
8	478 to 483	AHLD.IA	51.8 kD	4.3 kD	0.018	53.125	VHRDMA	0.975	>95%
9	160 to 165	YEYD.DL	17.5 kD	38.6 kD	0.015	56.250	VDFDDI	0.826	>95%
10	140 to 145	VEVD.PC	15.5 kD	40.6 kD	0.009	80.645	VEVDPM	0.749	>95%
11	424 to 429	ETGD.RV	45.5 kD	10.6 kD	0.008	70.968	DTGDAV	0.588	>95%
12	161 to 166	EYDD.LK	17.6 kD	38.5 kD	0.012	46.875	DYKDIA	0.562	>95%
13	394 to 399	GAMD.VA	42.4 kD	13.7 kD	0.009	53.571	DALDAA	0.466	>95%
14	28 to 33	STAD.MT	3.4 kD	52.7 kD	0.006	75.862	STTDLT	0.437	>95%
15	441 to 446	QVID.CQ	47.8 kD	8.4 kD	0.004	55.172	SVIDGQ	0.193	>95%
16	179 to 184	GSGD.LE	19.5 kD	36.6 kD	0.004	41.379	DSSDSE	0.186	>95%
17	446 to 451	QLAD.VN	48.3 kD	7.8 kD	0.003	46.429	SLLDSN	0.137	>95%
18	89 to 94	LHQD.FP	10.1 kD	46.1 kD	0.002	48.276	LSSDFT	0.101	>95%
19	144 to 149	PCGD.AQ	15.9 kD	40.2 kD	0.002	48.276	PAADAI	0.088	>95%
20	45 to 50	KDDD.LP	5.3 kD	50.9 kD	0.001	57.143	KESDLS	0.077	>95%

>TERA_MOUSE_

rank	position	site	N fragment	C fragment	frequency score	similarity maxscore	similarity maxsite	average score	specificity
1	577 to 582	DELD.SI	64.5 kD	24.8 kD	7.179	100.000	DELDSI	717.907	>99.9%
2	304 to 309	DELD.AI	34.1 kD	55.2 kD	4.198	100.000	DELDAI	419.793	>99.9%
3	392 to 397	DDVD.LE	44.1 kD	45.2 kD	0.839	80.645	DDVDSE	67.624	>99.9%
4	428 to 433	DLID.LE	47.9 kD	41.4 kD	0.262	79.310	DLVDAE	20.779	>99%
5	330 to 335	TLMD.GL	37.1 kD	52.2 kD	0.191	77.419	DLMDGL	14.752	>99%
6	606 to 611	TEMD.GM	67.4 kD	21.9 kD	0.199	70.968	TEIDGR	14.151	>99%
7	166 to 171	VETD.PS	19.1 kD	70.2 kD	0.163	68.966	VETDKA	11.239	>99%
8	3 to 8	SGAD.SK	0.5 kD	88.8 kD	0.158	61.290	DGADST	9.701	>99%
9	435 to 440	ETID.AE	48.7 kD	40.6 kD	0.105	68.966	EEIDAQ	7.249	>99%
10	627 to 632	DIID.PA	69.7 kD	19.6 kD	0.109	57.143	DALDAA	6.210	>99%
11	722 to 727	VEED.DP	80.4 kD	8.9 kD	0.061	60.000	VEVDAP	3.663	>95%
12	431 to 436	DLED.ET	48.2 kD	41.1 kD	0.049	57.576	DLNDGT	2.849	>95%
13	176 to 181	VAPD.TV	20.1 kD	69.2 kD	0.037	62.069	VVPDAL	2.315	>95%
14	365 to 370	REVD.IG	41.1 kD	48.2 kD	0.034	62.069	LEVDAG	2.082	>95%
15	746 to 751	SVSD.ND	83.3 kD	6.0 kD	0.029	70.968	DVSDNE	2.039	>95%
16	26 to 31	LIVD.EA	3.1 kD	86.2 kD	0.035	53.333	LVIDNG	1.887	>95%
17	723 to 728	EEDD.PV	80.6 kD	8.7 kD	0.027	66.667	DEDDDV	1.817	>95%
18	370 to 375	GIPD.AT	41.6 kD	47.7 kD	0.025	62.069	SVPDSS	1.544	>95%
19	799 to 804	DNDD.DL	88.9 kD	0.4 kD	0.018	75.758	DEDDDV	1.393	>95%
20	589 to 594	NIGD.GG	65.7 kD	23.6 kD	0.018	71.875	DISDGG	1.318	>95%

>NDKB_MOUSE_

rank	position	site	N fragment	C fragment	frequency score	similarity maxscore	similarity maxsite	average score	specificity
1	138 to 143	ELID.YK	16.0 kD	1.4 kD	0.100	67.742	ESVDYR	6.789	>99%
2	54 to 59	DLKD.RP	6.7 kD	10.7 kD	0.058	67.647	DLRDDP	3.953	>99%
3	121 to 126	DSVE.SA	13.9 kD	3.4 kD	0.049	69.231	SSVDSA	3.399	>95%
4	11 to 16	IKPD.GV	1.6 kD	15.7 kD	0.037	63.636	EEPDGV	2.346	>95%
5	95 to 100	NPAD.SK	11.1 kD	6.2 kD	0.012	68.966	NSVDSK	0.819	>95%
6	118 to 123	HGSD.SV	13.6 kD	3.8 kD	0.008	51.613	DGADST	0.402	>95%
7	51 to 56	HYID.LK	6.3 kD	11.0 kD	0.003	54.839	DYLDLA	0.152	>95%

>MAP1
1_MO
USE_

rank	position	site	N fragment	C fragment	frequency score	similarity maxscore	similarity maxsite	average score	specificity
1	164 to 169	EEID.HA	18.6 kD	24.6 kD	0.343	100.000	EEIDHA	34.286	>99%
2	148 to 153	EVLD.IA	17.0 kD	26.2 kD	0.155	65.517	DVTDIA	10.156	>99%
3	362 to 367	LVTD.TG	40.7 kD	2.5 kD	0.085	63.333	LVIDNG	5.355	>99%
4	347 to 352	VTRD.GK	39.1 kD	4.1 kD	0.097	54.545	VYRDGT	5.297	>99%
5	107 to 112	DYAD.HP	12.4 kD	30.8 kD	0.084	60.606	DYADGA	5.112	>99%
6	9 to 14	CETD.GC	1.3 kD	41.9 kD	0.060	61.290	AETDGQ	3.673	>95%
7	239 to 244	GDVD.EG	27.0 kD	16.2 kD	0.057	55.263	GHVDHG	3.173	>95%
8	258 to 263	QAID.AV	29.2 kD	14.0 kD	0.027	77.778	MAVDAV	2.062	>95%
9	373 to 378	RRLD.SS	42.0 kD	1.2 kD	0.011	65.517	KRIDQS	0.743	>95%
10	132 to 137	SSED.IE	15.1 kD	28.1 kD	0.012	53.571	ESQDVS	0.654	>95%
11	69 to 74	VEGD.VN	8.0 kD	35.2 kD	0.013	48.387	VEVDGN	0.610	>95%
12	339 to 344	TWPD.GW	38.2 kD	5.0 kD	0.006	62.500	TLPDGL	0.347	>95%
13	104 to 109	QRPD.YA	12.1 kD	31.1 kD	0.005	56.757	DQPDYG	0.279	>95%
14	217 to 222	VNVD.IT	24.5 kD	18.7 kD	0.006	48.148	VEVDAA	0.270	>95%
15	212 to 217	QEGD.IV	24.0 kD	19.2 kD	0.005	51.852	SESDAV	0.260	>95%
16	204 to 209	GIPD.RR	23.0 kD	20.2 kD	0.004	48.276	SVPDSS	0.174	>95%
17	240 to 245	DVDE.GA	27.2 kD	16.1 kD	0.003	53.125	DISDGG	0.151	>95%

>TSR2_MOUSE_

rank	position	site	N fragment	C fragment	frequency score	similarity maxscore	similarity maxsite	average score	specificity
1	138 to 143	DDVD.SV	15.3 kD	5.6 kD	10.170	86.207	DEVDSI	876.694	>99.9%
2	80 to 85	VVED.GS	8.9 kD	11.9 kD	0.209	60.714	VVADGV	12.672	>99%
3	167 to 172	SDPD.TQ	18.3 kD	2.5 kD	0.080	60.606	SQPDTG	4.878	>99%
4	150 to 155	ATND.GA	16.6 kD	4.3 kD	0.076	58.621	ATIDGL	4.445	>99%
5	75 to 80	TEFD.TV	8.4 kD	12.5 kD	0.063	66.667	DEYDTV	4.188	>99%
6	131 to 136	KETD.VA	14.6 kD	6.3 kD	0.058	67.857	KESDLS	3.931	>99%
7	155 to 160	ATTD.EV	17.1 kD	3.8 kD	0.020	59.259	SVTDSV	1.191	>95%
8	64 to 69	EIED.FL	7.1 kD	13.8 kD	0.017	57.143	DYEDFM	0.952	>95%
9	101 to 106	QKGD.GA	11.3 kD	9.5 kD	0.011	68.750	DSGDGA	0.772	>95%
10	135 to 140	VAED.DV	15.0 kD	5.9 kD	0.011	60.714	VASDGV	0.642	>95%
11	136 to 141	AEDD.VD	15.1 kD	5.8 kD	0.009	58.824	GEDDRD	0.539	>95%
12	175 to 180	KEED.IV	19.3 kD	1.6 kD	0.005	66.667	DEEDIL	0.316	>95%
13	179 to 184	IVED.GW	19.7 kD	1.1 kD	0.004	53.571	VVADGV	0.191	>95%
14	49 to 54	AVED.YF	5.3 kD	15.5 kD	0.003	52.632	GYEDYY	0.172	>95%
15	4 to 9	AAED.VR	0.6 kD	20.2 kD	0.003	40.741	AAVDTS	0.137	>95%
16	56 to 61	ANAD.LE	6.1 kD	14.7 kD	0.002	42.857	IEADSE	0.101	>95%
17	178 to 183	DIVE.DG	19.6 kD	1.2 kD	0.001	71.875	DLLDDG	0.100	>95%

**>AT2B
1_MO
USE_**

rank	position	site	N fragment	C fragment	frequency score	similarity maxscore	similarity maxsite	average score	specificity
1	136 to 141	EQPD.NG	16.4 kD	16.9 kD	0.191	72.727	SQPDTG	13.911	>99%
2	272 to 277	DERD.KF	31.6 kD	1.7 kD	0.055	63.636	DEQDSY	3.505	>95%
3	221 to 226	GSID.LM	25.7 kD	7.6 kD	0.013	53.571	DSVDLA	0.707	>95%
4	89 to 94	ENLD.VI	10.9 kD	22.4 kD	0.007	58.621	DEIDVV	0.432	>95%
5	268 to 273	IATD.DE	31.1 kD	2.2 kD	0.008	50.000	IEADSE	0.380	>95%
6	65 to 70	TVSD.HT	8.1 kD	25.2 kD	0.006	46.429	TLTDSS	0.256	>95%
7	204 to 209	RDED.AE	23.9 kD	9.4 kD	0.003	51.724	EQEDAS	0.157	>95%
8	116 to 121	PYND.SI	14.0 kD	19.3 kD	0.003	50.000	DYTDAV	0.132	>95%
9	156 to 161	QLGD.CS	18.7 kD	14.7 kD	0.003	48.485	KLTDCV	0.122	>95%
10	96 to 101	NISD.TE	11.6 kD	21.7 kD	0.002	61.290	DVSDNE	0.106	>95%

**>PSA7
_MOU
SE_**

rank	position	site	N fragment	C fragment	frequency score	similarity maxscore	similarity maxsite	average score	specificity
1	181 to 186	IETD.DL	20.5 kD	7.4 kD	0.104	71.875	DETDDL	7.487	>99%
2	10 to 15	FSPD.GH	1.5 kD	26.4 kD	0.038	58.824	DSPDGQ	2.257	>95%
3	175 to 180	NYTD.DA	19.8 kD	8.0 kD	0.028	62.500	DKTDDA	1.743	>95%
4	76 to 81	LTAD.AR	8.5 kD	19.3 kD	0.023	48.148	SSLDAR	1.130	>95%
5	64 to 69	ALDD.NV	7.3 kD	20.5 kD	0.011	71.429	ALDDLI	0.800	>95%
6	136 to 141	FDFD.GT	15.5 kD	12.4 kD	0.012	58.065	MDIDGV	0.694	>95%
7	1 to 6	MSYD.RA	0.5 kD	27.3 kD	0.010	55.172	LSVDRG	0.532	>95%
8	145 to 150	YQTD.PS	16.5 kD	11.4 kD	0.009	53.333	DQTDSS	0.464	>95%
9	182 to 187	ETDD.LT	20.6 kD	7.2 kD	0.007	65.517	STTDLT	0.444	>95%
10	63 to 68	CALD.DN	7.2 kD	20.6 kD	0.008	50.000	SLLDSN	0.424	>95%
11	97 to 102	TVED.PV	11.0 kD	16.8 kD	0.006	48.148	SVTDSV	0.312	>95%
12	134 to 139	VGFD.FD	15.2 kD	12.6 kD	0.002	48.387	LGTDSD	0.099	>95%
13	176 to 181	YTDD.AI	20.0 kD	7.9 kD	0.002	45.161	DTGDAV	0.088	>95%

>SNR40_MOUSE_

rank	position	site	N fragment	C fragment	frequency score	similarity maxscore	similarity maxsite	average score	specificity
1	118 to 123	YNTD.GS	12.9 kD	26.4 kD	0.053	66.667	TSTDGS	3.527	>95%
2	294 to 299	WSPD.GS	32.6 kD	6.7 kD	0.039	56.667	TSTDGS	2.214	>95%
3	234 to 239	GHAD.SV	25.8 kD	13.5 kD	0.039	44.828	EEADSM	1.727	>95%
4	171 to 176	GSDD.GT	18.6 kD	20.7 kD	0.028	48.571	GDSDGP	1.382	>95%
5	204 to 209	DTSD.QI	22.3 kD	16.9 kD	0.013	61.290	DTGDAV	0.781	>95%
6	128 to 133	ASTD.KT	13.9 kD	25.4 kD	0.012	64.286	ASTDSK	0.758	>95%
7	170 to 175	TGSD.DG	18.5 kD	20.8 kD	0.012	50.000	DGDDDA	0.614	>95%
8	212 to 217	GGID.ND	23.1 kD	16.1 kD	0.006	62.500	DGVDNS	0.389	>95%
9	201 to 206	TFND.TS	22.0 kD	17.2 kD	0.005	57.143	TLTDSS	0.294	>95%
10	237 to 242	DSVT.GL	26.1 kD	13.2 kD	0.004	62.500	DNVDGL	0.242	>95%
11	346 to 351	ASSD.KR	38.1 kD	1.1 kD	0.003	60.714	ASTDSK	0.190	>95%
12	85 to 90	AGFD.RL	9.1 kD	30.2 kD	0.003	50.000	SELDRL	0.157	>95%
13	304 to 309	GSAD.RF	33.4 kD	5.8 kD	0.003	48.387	GSSDPL	0.157	>95%
14	254 to 259	NAMD.NT	27.8 kD	11.5 kD	0.002	58.065	RKLDNT	0.141	>95%
15	336 to 341	FHPD.EP	37.1 kD	2.1 kD	0.002	71.053	FDPDDP	0.125	>95%
16	98 to 103	GDCD.NY	10.7 kD	28.6 kD	0.002	54.545	TEVDNY	0.084	>95%
17	214 to 219	IDND.IK	23.3 kD	15.9 kD	0.001	42.424	DDNDSE	0.058	>95%

>PNPH _MOUSE_

rank	position	site	N fragment	C fragment	frequency score	similarity maxscore	similarity maxsite	average score	specificity
1	259 to 264	EVLD.AG	29.4 kD	2.8 kD	0.863	90.000	EVMDAG	77.653	>99.9%
2	164 to 169	DAYD.RD	18.7 kD	13.6 kD	0.031	65.625	DEVDRD	2.017	>95%
3	212 to 217	LGAD.AV	24.2 kD	8.1 kD	0.032	59.259	MAVDAV	1.891	>95%
4	161 to 166	AMSD.AY	18.3 kD	13.9 kD	0.016	56.667	LISDTY	0.883	>95%
5	283 to 288	PLPD.RG	32.0 kD	0.3 kD	0.013	51.613	DIVDRG	0.681	>95%
6	6 to 11	TYED.YE	1.2 kD	31.1 kD	0.005	55.263	GYEDYY	0.268	>95%
7	245 to 250	VVMD.YE	27.7 kD	4.5 kD	0.006	42.424	DVTDYK	0.245	>95%
8	125 to 130	EVGD.IM	14.3 kD	18.0 kD	0.004	58.065	DMGDLV	0.239	>95%
9	152 to 157	DERF.GV	17.4 kD	14.9 kD	0.001	48.387	DEVDGV	0.063	>95%

>HIBC
H_MO
USE_

rank	position	site	N fragment	C fragment	frequency score	similarity maxscore	similarity maxsite	average score	specificity
1	365 to 370	DVTD.ED	41.1 kD	1.9 kD	0.194	75.000	DMTDSD	14.527	>99%
2	136 to 141	ALID.GI	15.7 kD	27.4 kD	0.105	72.414	ATIDGL	7.619	>99%
3	235 to 240	SAED.VA	26.3 kD	16.7 kD	0.036	50.000	ESQDVS	1.820	>95%
4	379 to 384	GSSD.LK	42.6 kD	0.4 kD	0.022	53.571	SSTDAK	1.185	>95%
5	283 to 288	LRQD.GS	31.8 kD	11.2 kD	0.019	58.621	VKVDGS	1.112	>95%
6	214 to 219	HFVD.SE	24.0 kD	19.1 kD	0.020	54.545	DFLDNE	1.073	>95%
7	173 to 178	LFPD.VG	19.5 kD	23.6 kD	0.013	58.621	IVPDIA	0.777	>95%
8	367 to 372	TDED.LN	41.3 kD	1.7 kD	0.012	55.172	TDSDSS	0.664	>95%
9	348 to 353	VLID.KD	39.1 kD	3.9 kD	0.006	53.571	VLVDAG	0.299	>95%
10	76 to 81	QDPD.TF	9.2 kD	33.8 kD	0.003	58.065	EEPDSI	0.202	>95%
11	252 to 257	MDQD.KS	28.2 kD	14.9 kD	0.002	61.290	MDVDNS	0.135	>95%
12	337 to 342	EGHD.FH	37.9 kD	5.1 kD	0.002	50.000	DEHDEH	0.108	>95%
13	95 to 100	AGGD.IK	11.0 kD	32.0 kD	0.002	45.161	DGVDLK	0.075	>95%
14	112 to 117	LTQD.LF	12.9 kD	30.1 kD	0.001	39.286	VSVDAF	0.054	>95%
15	262 to 267	EHMD.KI	29.4 kD	13.6 kD	0.001	50.000	ESVDKS	0.053	>95%
16	250 to 255	SKMD.QD	27.9 kD	15.1 kD	0.001	59.375	DEVDQD	0.050	>95%

>RPP40_MOUSE_

rank	position	site	N fragment	C fragment	frequency score	similarity maxscore	similarity maxsite	average score	specificity
1	149 to 154	DLMD.LS	17.4 kD	24.1 kD	0.312	72.414	DMLDLA	22.569	>99%
2	155 to 160	LNLD.SK	18.1 kD	23.4 kD	0.065	58.621	TNLDSL	3.834	>99%
3	316 to 321	GFAD.SP	36.4 kD	5.1 kD	0.044	64.706	SFPDSP	2.847	>95%
4	99 to 104	TSID.ED	11.8 kD	29.7 kD	0.012	54.839	TELDMD	0.645	>95%
5	117 to 122	LSLD.KD	13.7 kD	27.8 kD	0.009	55.172	LSVDRG	0.507	>95%
6	249 to 254	CSAD.LN	28.9 kD	12.6 kD	0.008	51.429	CFADVG	0.423	>95%
7	59 to 64	LVMD.IG	7.3 kD	34.2 kD	0.007	60.000	LVIDNG	0.417	>95%
8	146 to 151	ISID.LM	17.1 kD	24.5 kD	0.006	53.571	VSVDAF	0.330	>95%
9	101 to 106	IDED.NT	12.1 kD	29.4 kD	0.003	58.065	MDVDNS	0.202	>95%
10	119 to 124	LDKD.TY	14.0 kD	27.5 kD	0.003	63.333	LISDTY	0.194	>95%
11	239 to 244	EFFD.WL	27.9 kD	13.7 kD	0.002	50.000	DFLDAL	0.118	>95%
12	175 to 180	LKFD.FL	20.6 kD	20.9 kD	0.003	44.828	LSSDFT	0.118	>95%
13	302 to 307	DEPK.LA	34.9 kD	6.6 kD	0.001	56.667	DETDLA	0.057	>95%

>AGO2_MOUSE_

rank	position	site	N fragment	C fragment	frequency score	similarity maxscore	similarity maxsite	average score	specificity
1	250 to 255	PLTD.SQ	28.5 kD	68.7 kD	0.121	64.286	TLTDSS	7.756	>99%
2	237 to 242	EVLD.FK	27.0 kD	70.3 kD	0.126	59.375	EEMDFR	7.453	>99%
3	735 to 740	TTVD.TK	83.3 kD	14.0 kD	0.101	71.429	TAVDAK	7.243	>99%
4	821 to 826	KEHD.SA	93.4 kD	3.9 kD	0.065	62.069	QETDSA	4.029	>99%
5	143 to 148	ALHD.AL	16.5 kD	80.8 kD	0.044	50.000	ALDDLI	2.203	>95%
6	478 to 483	ISRD.AG	54.8 kD	42.5 kD	0.040	53.333	IETDSG	2.144	>95%
7	372 to 377	SAPD.RQ	42.6 kD	54.7 kD	0.032	59.375	NSPDAQ	1.886	>95%
8	58 to 63	YELD.IK	6.7 kD	90.6 kD	0.037	50.000	QEIDYK	1.861	>95%
9	405 to 410	EMTD.VT	46.5 kD	50.8 kD	0.025	66.667	EETDLT	1.671	>95%
10	745 to 750	TEFD.FY	84.5 kD	12.8 kD	0.026	59.375	TEVDFN	1.568	>95%
11	497 to 502	QGAD.SV	56.8 kD	40.5 kD	0.019	64.516	DGADST	1.243	>95%
12	667 to 672	FYRD.GV	75.7 kD	21.6 kD	0.017	69.697	VYRDGT	1.186	>95%
13	603 to 608	PAGD.GK	68.2 kD	29.0 kD	0.016	58.824	DAGDGD	0.928	>95%
14	695 to 700	LEKD.YQ	78.9 kD	18.4 kD	0.015	58.065	MEVDYS	0.852	>95%
15	617 to 622	GSMD.AH	69.6 kD	27.7 kD	0.014	59.259	SSLDAR	0.810	>95%
16	93 to 98	PVFD.GR	10.9 kD	86.3 kD	0.013	55.882	DVFDGD	0.738	>95%
17	824 to 829	DSAE.GS	93.7 kD	3.6 kD	0.010	70.968	DEADGS	0.706	>95%
18	356 to 361	KLTD.NQ	40.9 kD	56.4 kD	0.012	54.839	DVVDNQ	0.673	>95%
19	161 to 166	QALD.VV	18.4 kD	78.9 kD	0.009	55.556	MAVDAV	0.490	>95%
20	46 to 51	FEMD.IP	5.2 kD	92.1 kD	0.006	56.667	VEVDAP	0.361	>95%

>BUB3_MOUSE_

rank	position	site	N fragment	C fragment	frequency score	similarity maxscore	similarity maxsite	average score	specificity
1	58 to 63	AVLD.CA	6.8 kD	30.1 kD	0.059	48.148	AEVDAA	2.865	>95%
2	315 to 320	QVTD.AE	36.1 kD	0.8 kD	0.039	70.000	DVTDAQ	2.720	>95%
3	206 to 211	EYLD.PS	23.6 kD	13.3 kD	0.043	59.375	EYIDGA	2.555	>95%
4	93 to 98	GTHD.AP	10.7 kD	26.2 kD	0.016	72.222	ATHDGP	1.187	>95%
5	252 to 257	GGSD.GF	28.8 kD	8.1 kD	0.012	54.545	DGTDGL	0.660	>95%
6	284 to 289	FSND.GT	32.6 kD	4.3 kD	0.009	54.545	DLNDGT	0.517	>95%
7	145 to 150	VSGD.RL	16.5 kD	20.4 kD	0.008	43.333	VLGDGV	0.357	>95%
8	299 to 304	YEMD.DT	34.2 kD	2.7 kD	0.006	58.065	DEMDST	0.322	>95%
9	73 to 78	GGLD.HQ	8.4 kD	28.5 kD	0.006	46.667	ESLDNQ	0.301	>95%
10	300 to 305	EMDD.TE	34.4 kD	2.6 kD	0.004	54.839	DMDDVV	0.221	>95%
11	12 to 17	PPED.GI	1.7 kD	35.2 kD	0.002	51.724	PAADAI	0.117	>95%
12	84 to 89	LNTD.QE	9.7 kD	27.2 kD	0.001	54.839	LGTDSD	0.070	>95%
13	306 to 311	HPED.GI	35.1 kD	1.9 kD	0.001	61.765	YPVDGL	0.070	>95%
14	302 to 307	DDTE.HP	34.6 kD	2.4 kD	0.001	64.706	DETDHS	0.063	>95%

>G6PE_MOUSE_

rank	position	site	N fragment	C fragment	frequency score	similarity maxscore	similarity maxsite	average score	specificity
1	625 to 630	SDPD.SN	71.1 kD	17.8 kD	0.386	65.517	SVPDSS	25.260	>99%
2	486 to 491	PLLD.SL	55.9 kD	33.0 kD	0.172	62.963	SLLDEL	10.818	>99%
3	706 to 711	TGLD.GD	79.9 kD	9.0 kD	0.130	76.667	SALDGD	9.961	>99%
4	240 to 245	ETID.AE	27.6 kD	61.3 kD	0.105	68.966	EEIDAQ	7.249	>99%
5	125 to 130	VQQD.GL	14.2 kD	74.7 kD	0.082	57.143	VASDGV	4.671	>99%
6	220 to 225	KALD.GL	25.1 kD	63.8 kD	0.067	66.667	RAIDAL	4.497	>99%
7	691 to 696	MGTD.GH	78.4 kD	10.5 kD	0.041	58.065	LGTDSD	2.370	>95%
8	682 to 687	SSFD.LV	77.5 kD	11.4 kD	0.043	46.429	DSVDLA	1.981	>95%
9	528 to 533	LIPD.LG	60.4 kD	28.5 kD	0.025	68.966	IVPDIA	1.758	>95%
10	46 to 51	LYLD.EA	5.3 kD	83.6 kD	0.023	48.387	SYLDSG	1.119	>95%
11	662 to 667	AEED.QG	75.4 kD	13.5 kD	0.019	54.286	AEPDYG	1.037	>95%
12	339 to 344	VHID.NL	38.6 kD	50.3 kD	0.018	56.250	DNIDNL	1.028	>95%
13	145 to 150	AYAD.IA	16.5 kD	72.4 kD	0.012	56.250	DYKDIA	0.654	>95%
14	110 to 115	TVED.YQ	12.4 kD	76.5 kD	0.013	48.571	TATDYH	0.626	>95%
15	30 to 35	ATGD.LA	3.4 kD	85.6 kD	0.011	48.276	STTDLT	0.545	>95%
16	451 to 456	REQD.AY	51.7 kD	37.2 kD	0.008	66.667	DEQDSY	0.513	>95%
17	256 to 261	VIRD.TL	29.4 kD	59.5 kD	0.009	58.621	VLKDTQ	0.502	>95%
18	322 to 327	QKPD.GF	36.8 kD	52.1 kD	0.008	63.636	EEPDGV	0.489	>95%
19	507 to 512	QLLD.FE	58.1 kD	30.8 kD	0.008	48.276	DLVDAE	0.394	>95%
20	427 to 432	EVQD.QP	48.9 kD	40.0 kD	0.006	56.667	ELLDSP	0.321	>95%
