# Supplementary material for: Proteome integral solubility alteration high-throughput proteomics assay identifies Collectin-12 as a non-apoptotic microglial caspase-3 substrate
Source: Cell Death Dis. 2023 Mar 11;14(3):192. doi: 10.1038/s41419-023-05714-2 (PMC10008626; doi:10.1038/s41419-023-05714-2)

Full and uncropped western blot for Figure 2C

Lanes 1, 2 are on the figure

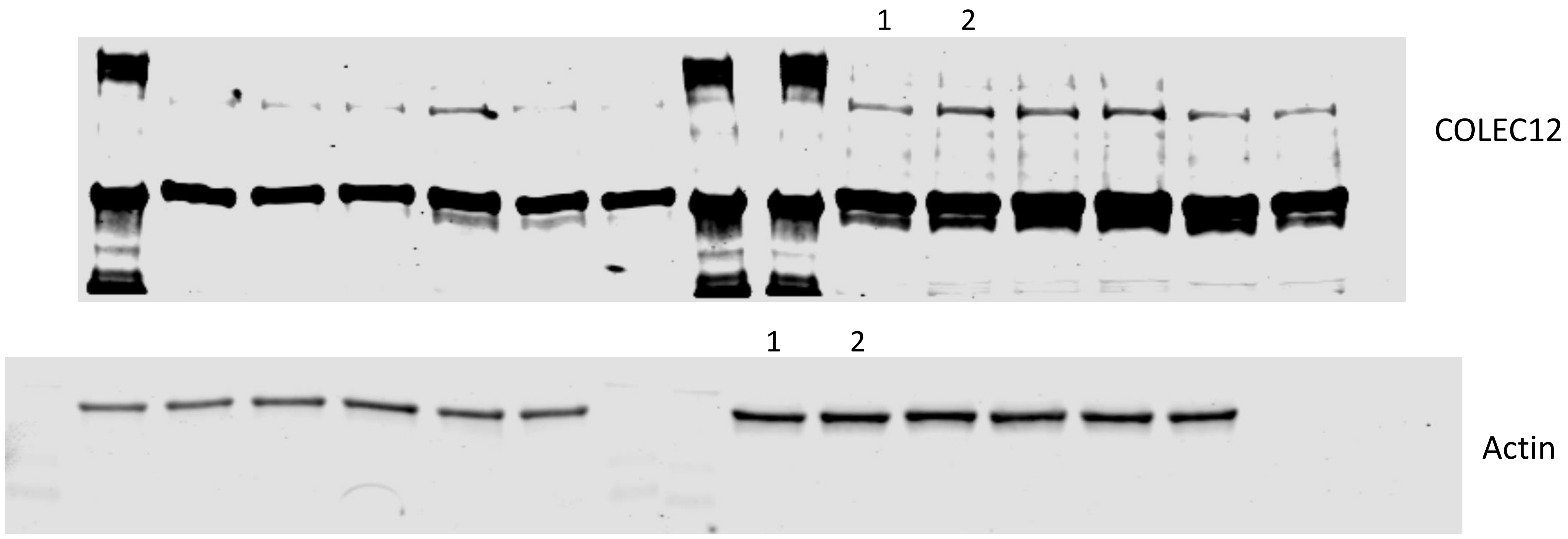

Full and uncropped western blot for Figure 2F

Lanes 3, 4 are on the figure

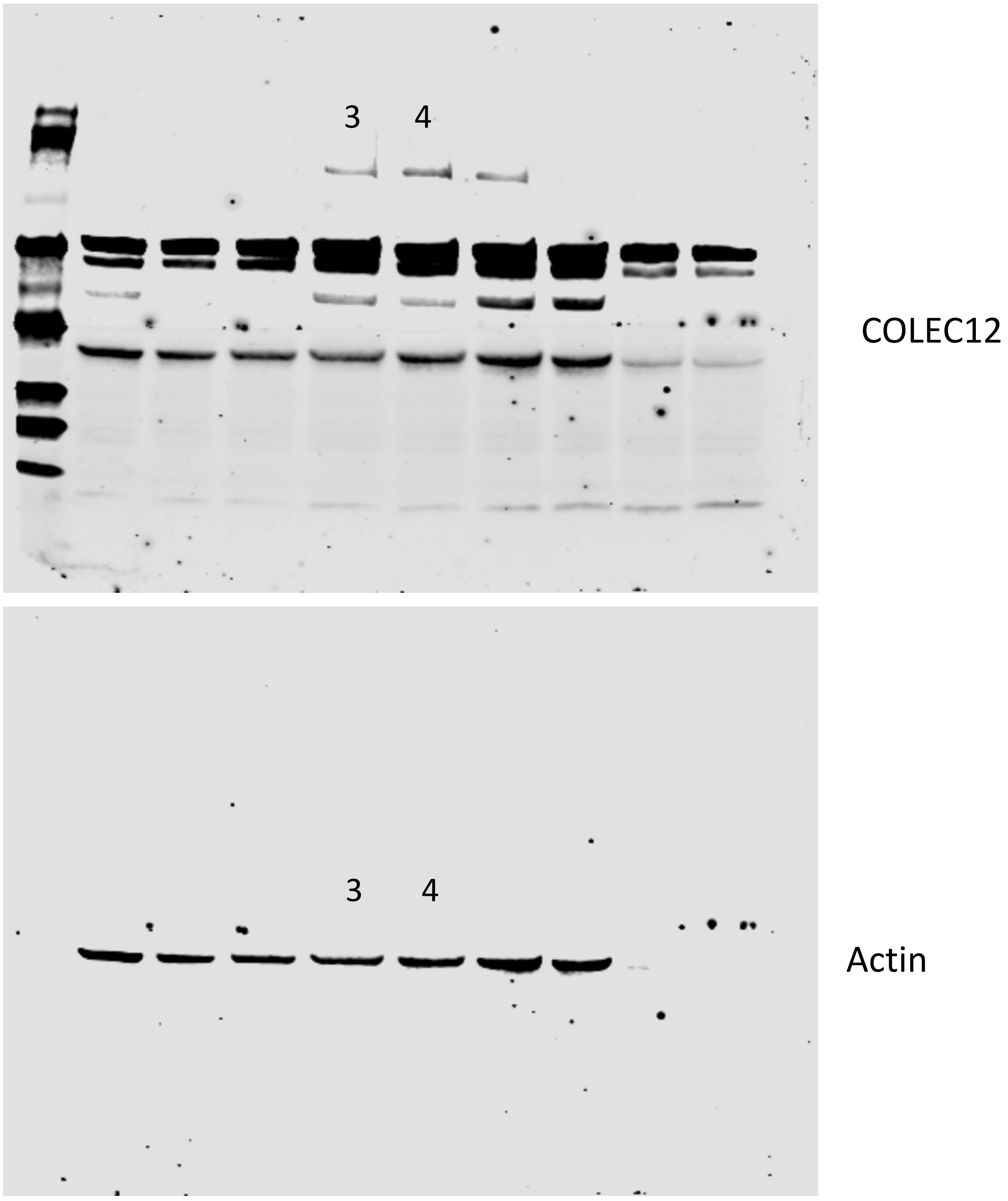

Full and uncropped western blot for Figure 2E
Lanes 5, 6 are on the figure

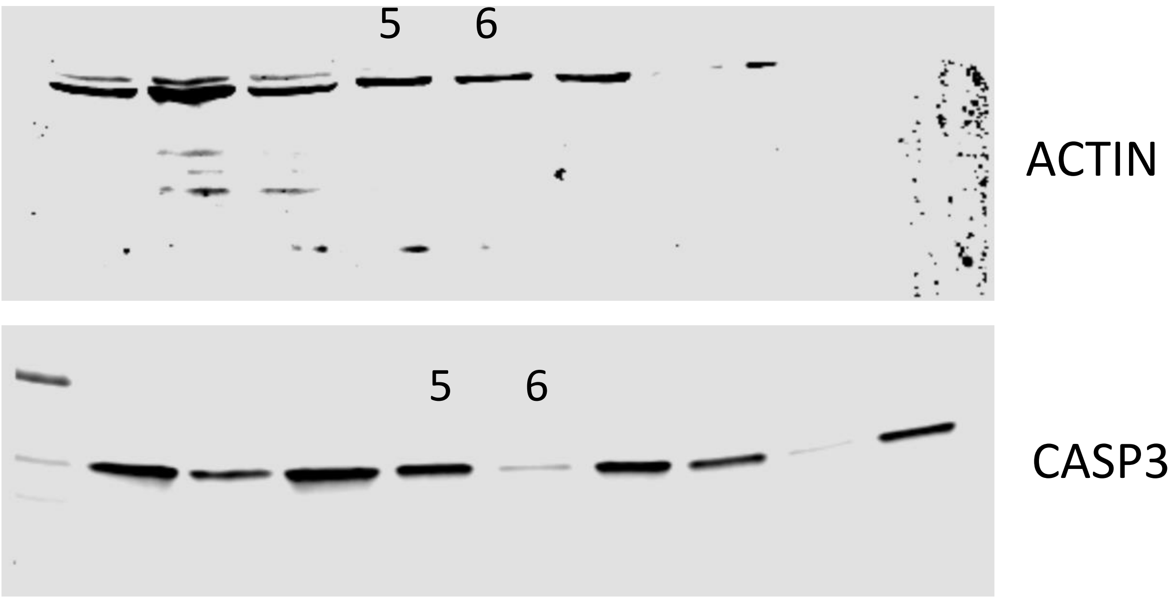

Full and uncropped western blot for Figure 2H
Lanes 7, 8 are on the figure

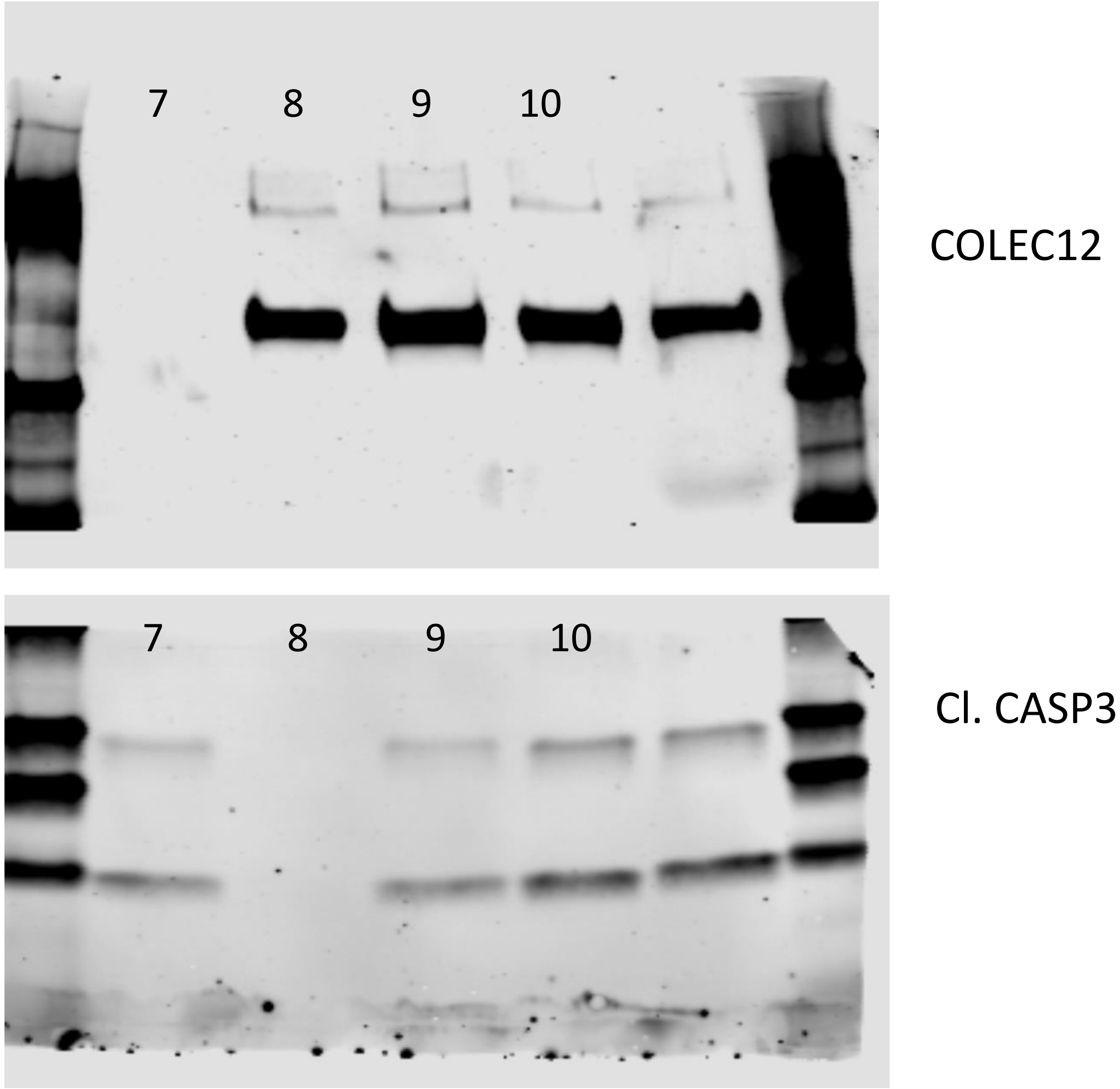

Supplement: Supplementary file 5 — Supplementary data file 1 [file 41419_2023_5714_MOESM5_ESM.pdf]
