# Supplementary material for: Proteome integral solubility alteration high-throughput proteomics assay identifies Collectin-12 as a non-apoptotic microglial caspase-3 substrate
Source: Cell Death Dis. 2023 Mar 11;14(3):192. doi: 10.1038/s41419-023-05714-2 (PMC10008626; doi:10.1038/s41419-023-05714-2)
Supplement: Supplementary file 3 — Supplementary Table 4 [file 41419_2023_5714_MOESM3_ESM.pdf]

Accession	Description	Coverage [%]	# Peptides	# PSMs	# Unique Peptides	# AAs	MW [kDa]	calc. pI	Biological Process	Cellular Component	Molecular Function	Pfam IDs	Entrez Gene ID	Ensembl Gene ID	Gene Symbol	Reactome Pathways	WikiPathways
A0A087WSS2	DNA mismatch repair protein Msh3 OS=Mus musculus OX=10090 GN=Msh3	6	2	2	2	360	41,1	9,42	DNA metabolism;other metabolic processes;stress response		nucleic acid binding activity;other molecular function	Pf01624		ENSMUSG00000014850	Msh3		
A2AF47	Dedicator of cytokinesis protein 11 OS=Mus musculus OX=10090 GN=Dock1	6	11	14	11	2073	237,6	7,78	developmental processes;signal transduction;other biological processes		enzyme regulator activity;other molecular function	Pf00169, Pf06920, Pf11878, Pf14429, Pf2	75974	ENSMUSG00000031093	Dock11	CDC42 GTPase cycle; RAC1 GTPase cycle; Factors involved in m	
P35456	Urokinase plasminogen activator surface receptor OS=Mus musculus OX=10	11	4	4	4	327	35,4	6,83	developmental processes;signal transduction;other biological processes	non-structural extracellular;plasma membrane;other membranes;other cell component	signal transduction activity or receptor binding;other molecular function	Pf00021	18793	ENSMUSG00000046223	Plaur	Neutrophil degranulation; Dissolution of Fibrin Clot; Attachmen	Primary focal segmental glomerulosclerosis (FSGS)
Q9DBN5	Lon protease homolog 2, peroxisomal OS=Mus musculus OX=10090 GN=Lon	3	2	2	2	852	94,5	7,23	cell organization and biogenesis;protein metabolism;other metabolic processes;t	nucleus;other cytoplasmic organelle;other cell component	other molecular function	Pf00004, Pf02190, Pf05362	66887	ENSMUSG00000047866	Lonp2	Peroxisomal protein import	
F6R782	IQ domain-containing protein E (Fragment) OS=Mus musculus OX=10090 GN	3	2	4	2	396	43	4,87				Pf00612		ENSMUSG00000036555	Iqce		
Q9CPX4	Ferritin OS=Mus musculus OX=10090 GN=Ftl1 PE=1 SV=1	64	8	23	8	183	20,8	6	transport;other biological processes		other molecular function	Pf00210	14325	ENSMUSG00000050708; ENSMUSG0000006	Ftl1		Iron homeostasis
O35516	Neurogenic locus notch homolog protein 2 OS=Mus musculus OX=10090 GN=	2	3	4	3	2473	265,5	5,22	cell cycle OR cell proliferation;stress response;developmental processes;signal tr	non-structural extracellular;extracellular matrix;plasma membrane;other membranes;cytosol;nucle	other molecular function	Pf00008, Pf00023, Pf00066, Pf06816, Pf0	18129	ENSMUSG00000027878	Notch2		Neural crest differentiation; Delta-Notch signaling pathv
F8WIP8	Osteopontin OS=Mus musculus OX=10090 GN=Spp1 PE=1 SV=1	45	9	39	9	295	32,6	4,55	cell adhesion;other metabolic processes;other biological processes	non-structural extracellular;ER/Golgi	signal transduction activity or receptor binding	Pf00865	20750	ENSMUSG00000029304	Spp1		TGF-beta signaling pathway; Endochondral ossification;
P06869	Urokinase-type plasminogen activator OS=Mus musculus OX=10090 GN=Pla	14	5	8	5	433	48,2	8,16	protein metabolism;other metabolic processes;stress response;developmental p	non-structural extracellular;other membranes;other cell component	other molecular function	Pf00051, Pf00089	18792	ENSMUSG00000021822	Plau	Neutrophil degranulation; Dissolution of Fibrin Clot	Endochondral ossification; Lung fibrosis; Wnt signaling p
A0A1W2P6U8	RIKEN cDNA 4932415D10 gene OS=Mus musculus OX=10090 GN=4932415D	1	2	4	2	4986	551,5	8		other membranes			102635990	ENSMUSG00000044581	4932415D10Ri		
Q80TA9	Ectopic P granules protein 5 homolog OS=Mus musculus OX=10090 GN=Epg5	1	2	2	2	2572	290,6	6,37	cell organization and biogenesis;other metabolic processes;transport;signal trans	other cytoplasmic organelle;other cell component			100502841	ENSMUSG00000039840	Epg5		
Q61543	Golgi apparatus protein 1 OS=Mus musculus OX=10090 GN=Glg1 PE=1 SV=1	13	13	15	13	1175	133,6	6,84	developmental processes;other biological processes	extracellular matrix;plasma membrane;other membranes;cytoskeleton;ER/Golgi;other cell compor	other molecular function	Pf00839	20340	ENSMUSG00000003316	Glg1	Cell surface interactions at the vascular wall	
Q9D5R3	Centrosomal protein of 83 kDa OS=Mus musculus OX=10090 GN=Cep83 PE=	3	2	2	2	692	81,9	6,61	cell organization and biogenesis;transport;other biological processes	cytoskeleton;ER/Golgi;other cell component	other molecular function		77048	ENSMUSG00000020024	Cep83	Anchoring of the basal body to the plasma membrane	
Q9D1D4	Transmembrane emp24 domain-containing protein 10 OS=Mus musculus OX	14	2	2	2	219	24,9	6,7	cell organization and biogenesis;transport;developmental processes;other biolog	plasma membrane;other membranes;ER/Golgi;other cell component	transporter activity;other molecular function	Pf01105	68581	ENSMUSG00000021248	Tmed10	COPI-mediated anterograde transport; COPI-dependent Golgi-	mRNA processing
B5B2N2	Nuclear factor of activated T-cells c1 isoform IA-IXL OS=Mus musculus OX=1	2	2	2	2	939	101,5	7,05	signal transduction;other biological processes	nucleus;other cell component	nucleic acid binding activity;other molecular function	Pf00554, Pf16179	18018	ENSMUSG00000033016	Nfatc1		Heart development
P52927	High mobility group protein HMGI-C OS=Mus musculus OX=10090 GN=Hmga	56	4	6	4	108	11,8	10,62	cell-cell signaling;cell cycle OR cell proliferation;cell organization and biogenesis;p	nucleus;other cell component	nucleic acid binding activity;other molecular function	Pf02178	15364	ENSMUSG00000056758	Hmga2	Formation of Senescence-Associated Heterochromatin Foci (SA	
Q62351	Transferrin receptor protein 1 OS=Mus musculus OX=10090 GN=Tfrc PE=1 S\	26	19	31	19	763	85,7	6,57	protein metabolism;other metabolic processes;transport;developmental process	non-structural extracellular;plasma membrane;other membranes;mitochondrion;nucleus;other cyt	transporter activity;nucleic acid binding activity;other molecular function	Pf02225, Pf04253, Pf04389	22042	ENSMUSG00000022797	Tfrc	Golgi Associated Vesicle Biogenesis; RHOA GTPase cycle; RHO	
Q5SWZ5	Myosin phosphatase Rho-interacting protein OS=Mus musculus OX=10090 G	1	3	4	2	2269	257,1	5,59				Pf00169	26936	ENSMUSG00000005417	Mprip		
Q9QYE6	Golgin subfamily A member 5 OS=Mus musculus OX=10090 GN=Golga5 PE=	6	4	6	4	729	82,3	6,23	cell organization and biogenesis;transport	other membranes;ER/Golgi	other molecular function	Pf09787	27277	ENSMUSG00000021192	Golga5		
[illegible]	[illegible]	[illegible]	[illegible]	[illegible]	[illegible]	[illegible]	[illegible]	[illegible]	[illegible]	[illegible]	[illegible]	[illegible]	[illegible]	[illegible]	[illegible]	[illegible]	[illegible]

Accession	# Razor Peptides	C<br>Abundances (Grouped) CV [%]: Control	T<br>Abundances (Grouped) CV [%]: Treated	C1<br>Abundance: F1: 130N, Control	C2<br>Abundance: F1: 129C, Control	C3<br>Abundance: F1: 128C, Control	C4<br>Abundance: F1: 127N, Control	C5<br>Abundance: F1: 130C, Control	T1<br>Abundance: F1: 129N, Treated	T2<br>Abundance: F1: 131, Treated	T3<br>Abundance: F1: 127C, Treated	T4<br>Abundance: F1: 126, Treated	T5<br>Abundance: F1: 128N, Treated	P>0.05<br>t.test	increased ΔSm<br>delta Sm	ratio	LOG2(FC)	-Log10(p)	Gene Symbol
A0A087WSS2	0	10,05	192,47	1,14720E+00	1,22967E+00	0,00000E+00	1,43242E+00	1,19071E+00	9,58829E-01	1,12390E+00	1,30107E+00	1,10502E+00	3,58687E+01	0,33898	7,07150	8,07150E+00	3,012836123	0,46982849	Msh3
A2AF47	0	10,66	100,57	8,99024E-01	1,08436E+00	8,70274E-01	1,08987E+00	1,05647E+00	9,01107E-01	1,16302E+00	1,00239E+00	9,36163E-01	5,08892E+00	0,34768	0,81832	1,81832E+00	0,862605563	0,45882364	Dock11
P35456	0	7,17	2,62	1,04889E+00	1,09602E+00	9,19506E-01	9,68803E-01	9,66786E-01	1,60406E+00	1,56891E+00	1,61568E+00	1,67160E+00	1,66635E+00	0,00000	0,62532	1,62532E+00	0,70072287	6,79635957	Plaur
Q9DBN5	0	21,67	13,52	9,54384E-01	8,77681E-01	9,79000E-01	8,18126E-01	1,37081E+00	1,32109E+00	1,77584E+00	1,31334E+00	1,50038E+00	1,35764E+00	0,00836	0,45366	1,45366E+00	0,53969138	2,0778112	Lonp2
F6R782	0	9,6	51	1,11345E+00	8,91443E-01	9,31567E-01	9,78948E-01	1,08459E+00	1,01042E+00	1,08886E+00	9,65158E-01	2,57794E+00	1,11306E+00	0,29143	0,35109	1,35109E+00	0,434120337	0,53546221	Iqce
Q9CPX4	0	6	6,37	1,00189E+00	1,01486E+00	9,87766E-01	9,14416E-01	1,08107E+00	1,31541E+00	1,39687E+00	1,31000E+00	1,40255E+00	1,19784E+00	0,00010	0,32453	1,32453E+00	0,405484993	3,9860012	Ftl1
O35516	0	5,52	5,9	9,62545E-01	1,09495E+00	9,69275E-01	9,76563E-01	9,96668E-01	1,29609E+00	1,20341E+00	1,35220E+00	1,35210E+00	1,41464E+00	0,00007	0,32369	1,32369E+00	0,404564246	4,16812509	Notch2
F8WIP8	0	6,14	3,78	1,05130E+00	1,00097E+00	9,07737E-01	9,81216E-01	1,05878E+00	1,23871E+00	1,23360E+00	1,32416E+00	1,30249E+00	1,34235E+00	0,00004	0,28826	1,28826E+00	0,36542528	4,43107421	Spp1
P06869	0	5,13	5,84	1,01042E+00	1,02363E+00	9,30091E-01	9,72127E-01	1,06374E+00	1,25470E+00	1,26995E+00	1,23545E+00	1,41930E+00	1,25900E+00	0,00010	0,28768	1,28768E+00	0,364774002	3,99913331	Plau
A0A1W2P6U8	0	8,11	17,25	9,95389E-01	1,05514E+00	8,67598E-01	1,00769E+00	1,07419E+00	1,18165E+00	1,10782E+00	1,47370E+00	1,56547E+00	1,08698E+00	0,02753	0,28312	1,28312E+00	0,359658641	1,56014008	4932415D10Rik
Q80TA9	0	36,32	19,1	7,54458E-01	8,19301E-01	7,35572E-01	1,09312E+00	1,59754E+00	1,13362E+00	1,05509E+00	1,50145E+00	1,57392E+00	1,10845E+00	0,19813	0,27451	1,27451E+00	0,349937287	0,70304061	Epg5
Q61543	0	8,96	4,86	9,79462E-01	9,31367E-01	9,50070E-01	9,83081E-01	1,15602E+00	1,24864E+00	1,32633E+00	1,19521E+00	1,24559E+00	1,34451E+00	0,00052	0,27206	1,27206E+00	0,347161494	3,28022811	Glg1
Q9D5R3	0	11,87	17,26	9,43265E-01	9,64481E-01	8,71985E-01	1,03470E+00	1,18557E+00	1,19224E+00	1,20803E+00	1,07053E+00	1,63912E+00	1,20952E+00	0,04455	0,26389	1,26389E+00	0,337869399	1,35110757	Cep83
Q9D1D4	0	23,37	44,29	7,76336E-01	1,06390E+00	1,32354E+00	7,65711E-01	1,07051E+00	1,06922E+00	2,15555E+00	7,70408E-01	9,05028E-01	1,30504E+00	0,39256	0,24105	1,24105E+00	0,31155966	0,40609501	Tmed10
B5B2N2	0	17,89	13,85	1,18371E+00	1,18616E+00	8,50896E-01	8,11836E-01	9,67400E-01	1,05929E+00	1,44609E+00	1,20905E+00	1,38039E+00	1,09716E+00	0,06265	0,23839	1,23839E+00	0,308471119	1,2031024	Nfatc1
P52927	0	11,88	19,38	9,23235E-01	9,75280E-01	9,17113E-01	9,77678E-01	1,20669E+00	1,04028E+00	1,08707E+00	1,08180E+00	1,58867E+00	1,38076E+00	0,08428	0,23571	1,23571E+00	0,305345414	1,07428525	Hmga2
Q62351	0	10,8	7,61	9,47785E-01	1,03281E+00	9,24567E-01	9,19851E-01	1,17499E+00	1,27786E+00	1,35693E+00	1,13294E+00	1,15450E+00	1,20030E+00	0,00771	0,22451	1,22451E+00	0,29219874	2,1131648	Tfrc
Q5SWZ5	0	16,45	15,98	7,47579E-01	1,00644E+00	9,76887E-01	1,19873E+00	1,07036E+00	1,22332E+00	1,02416E+00	1,15866E+00	1,14234E+00	1,54500E+00	0,09269	0,21870	1,21870E+00	0,285337986	1,0329758	Mprip
Q9QYE6	0	7,93	11,79	9,49663E-01	1,08232E+00	8,90721E-01	1,05880E+00	1,01850E+00	1,30914E+00	1,13983E+00	1,36939E+00	1,24801E+00	1,00932E+00	0,01859	0,21514	1,21514E+00	0,281120494	1,73069029	Golga5
[illegible]	[illegible]	[illegible]	[illegible]	[illegible]	[illegible]	[illegible]	[illegible]	[illegible]	[illegible]	[illegible]	[illegible]	[illegible]	[illegible]	[illegible]	[illegible]	[illegible]	[illegible]	[illegible]	[illegible]

P35969	Vascular endothelial growth factor receptor 1 OS=Mus musculus OX=10090	2	2	4	2	1333	149,8	8,31	[illegible]
Q9R1P3	Proteasome subunit beta type-2 OS=Mus musculus OX=10090 GN=Psmb2 P	58	9	102	9	201	22,9	7,02	[illegible]
A0A0R4J0P5	Proline-serine-threonine phosphatase-interacting protein 1 OS=Mus musculu	7	2	3	2	415	47,5	5,53	[illegible]
Q8CCS6	Polyadenylate-binding protein 2 OS=Mus musculus OX=10090 GN=Pabpn1 P	47	8	25	3	302	32,3	5,17	[illegible]
Q3UGR5	Haloacid dehalogenase-like hydrolase domain-containing protein 2 OS=Mus	46	9	21	9	259	28,7	6,05	[illegible]
Q9CPT4	Myeloid-derived growth factor OS=Mus musculus OX=10090 GN=Mydgf PE=	28	5	60	5	166	18	6,79	[illegible]
Q9JMH6	Thioredoxin reductase 1, cytoplasmic OS=Mus musculus OX=10090 GN=Txnr	44	19	97	12	613	67	7,44	[illegible]
Q9D0B6	Protein PBDC1 OS=Mus musculus OX=10090 GN=Pbdc1 PE=1 SV=1	54	10	41	10	198	22,2	4,55	[illegible]
A0A0A0MQ89	Actin-related protein 5 OS=Mus musculus OX=10090 GN=Actr5 PE=1 SV=1	8	4	4	4	608	68,1	5,16	[illegible]
E9PYH4	Synaptonemal complex central element protein 2 OS=Mus musculus OX=10	50	8	13	7	177	20,3	5,31	[illegible]
P56391	Cytochrome c oxidase subunit 6B1 OS=Mus musculus OX=10090 GN=Cox6b1	53	5	17	5	86	10,1	8,72	[illegible]
P30276	G2/mitotic-specific cyclin-B2 OS=Mus musculus OX=10090 GN=Ccnb2 PE=1 S	5	2	2	2	398	45,4	8,92	[illegible]
Q3U9G9	Delta(14)-sterol reductase LBR OS=Mus musculus OX=10090 GN=Lbr PE=1 SV	3	2	3	2	626	71,4	9,36	[illegible]
E9Q3P4	Centromere protein F OS=Mus musculus OX=10090 GN=Cenpf PE=1 SV=1	2	6	7	6	2997	342,3	5,16	[illegible]
Q9DCZ1	GMP reductase 1 OS=Mus musculus OX=10090 GN=Gmpr PE=1 SV=1	37	11	19	9	345	37,5	7,09	[illegible]
Q924Z6	Exportin-6 OS=Mus musculus OX=10090 GN=Xpo6 PE=1 SV=2	4	3	3	3	1125	128,6	6,37	[illegible]
E0CYQ0	Potassium channel tetramerisation domain-containing 17 OS=Mus musculus	15	4	5	3	296	33,3	5,27	[illegible]
Q61686	Chromobox protein homolog 5 OS=Mus musculus OX=10090 GN=Cbx5 PE=1	61	12	40	11	191	22,2	5,86	[illegible]
Q80X71	Transmembrane protein 106B OS=Mus musculus OX=10090 GN=Tmem106b	7	2	3	2	275	31,2	6,68	[illegible]
D3Z723	CAAT box DNA-binding protein subunit B (Fragment) OS=Mus musculus OX=	20	2	5	2	106	11,7	4,58	[illegible]
Q9Z2I0	Mitochondrial proton/calcium exchanger protein OS=Mus musculus OX=100	10	6	8	6	738	82,9	6,52	[illegible]
Q7TNC4	Putative RNA-binding protein Luc7-like 2 OS=Mus musculus OX=10090 GN=L	38	16	38	9	392	46,6	10,1	[illegible]
Q5SUT0	RNA-binding protein EWS OS=Mus musculus OX=10090 GN=Ewsr1 PE=1 SV=	16	8	57	8	618	64,9	9,38	[illegible]
Q9JMG1	Endothelial differentiation-related factor 1 OS=Mus musculus OX=10090 GN=	70	16	66	16	148	16,4	9,99	[illegible]
A0A0G2JF97	Proteasome subunit alpha type-5 (Fragment) OS=Mus musculus OX=10090	58	7	28	5	55	6,1	6,77	[illegible]
P97450	ATP synthase-coupling factor 6, mitochondrial OS=Mus musculus OX=10090	51	7	28	7	108	12,5	9,36	[illegible]
Q8BM72	Heat shock 70 kDa protein 13 OS=Mus musculus OX=10090 GN=Hspa13 PE=	13	6	7	6	471	51,7	5,63	[illegible]
Q9CQA9	Cancer-related nucleoside-triphosphatase homolog OS=Mus musculus OX=1	21	3	3	3	190	20,7	9,72	[illegible]
Q99LN9	Deoxyhypusine hydroxylase OS=Mus musculus OX=10090 GN=Dohh PE=1 SV	33	7	23	7	302	32,9	4,84	[illegible]
E9Q401	Ryanodine receptor 2 OS=Mus musculus OX=10090 GN=Ryr2 PE=1 SV=1	1	4	13	2	4966	564,5	6,09	[illegible]
A0A0A0MQ80	Vesicle-fusing ATPase OS=Mus musculus OX=10090 GN=Spata5 PE=1 SV=1	4	3	30	2	892	97,1	8,24	[illegible]
Q80XN0	D-beta-hydroxybutyrate dehydrogenase, mitochondrial OS=Mus musculus O	7	2	2	2	343	38,3	9,01	[illegible]
Q9CR46	Spindle and kinetochore-associated protein 2 OS=Mus musculus OX=10090	29	4	5	4	120	13,7	5,55	[illegible]
Q9DC33	High mobility group protein 20A OS=Mus musculus OX=10090 GN=Hmg20a	5	2	2	2	346	39,9	6,4	[illegible]
O88188	Lymphocyte antigen 86 OS=Mus musculus OX=10090 GN=Ly86 PE=1 SV=1	17	2	4	2	162	17,8	5,22	[illegible]
P19536	Cytochrome c oxidase subunit 5B, mitochondrial OS=Mus musculus OX=100	31	4	14	4	128	13,8	8,38	[illegible]
Q9CWW6	Peptidyl-prolyl cis-trans isomerase NIMA-interacting 4 OS=Mus musculus OX	45	7	30	7	131	13,8	9,77	[illegible]
O70494	Transcription factor Sp3 OS=Mus musculus OX=10090 GN=Sp3 PE=1 SV=2	7	3	4	3	783	82,3	5,26	[illegible]
Q9JIA7	Sphingosine kinase 2 OS=Mus musculus OX=10090 GN=Sphk2 PE=1 SV=2	5	2	3	2	617	65,6	6,57	[illegible]
Q9D051	Pyruvate dehydrogenase E1 component subunit beta, mitochondrial OS=Mu	53	14	73	14	359	38,9	6,87	[illegible]
P25976	Nucleolar transcription factor 1 OS=Mus musculus OX=10090 GN=Ubtf PE=1	27	20	28	20	765	89,5	5,76	[illegible]
Q8VE10	N-alpha-acetyltransferase 40 OS=Mus musculus OX=10090 GN=Naa40 PE=1	19	4	4	4	237	27,2	7,36	[illegible]
Q60972	Histone-binding protein RBBP4 OS=Mus musculus OX=10090 GN=Rbbp4 PE=	47	16	90	9	425	47,6	4,89	[illegible]
P97449	Aminopeptidase N OS=Mus musculus OX=10090 GN=Anpep PE=1 SV=4	14	12	20	12	966	109,6	5,9	[illegible]
Q8VC31	Coiled-coil domain-containing protein 9 OS=Mus musculus OX=10090 GN=Cc	19	12	25	12	543	61,4	5	[illegible]
D3Z3C1	Fas apoptotic inhibitory molecule 1 OS=Mus musculus OX=10090 GN=Faim P	23	5	9	5	201	22,6	5,02	[illegible]
Q8R5I6	Glutathione S-transferase Mu 4 OS=Mus musculus OX=10090 GN=Gstm4 PE	46	9	26	4	218	25,5	6,93	[illegible]
O54879	High mobility group protein B3 OS=Mus musculus OX=10090 GN=Hmgb3 PE=	38	7	19	7	200	23	8,37	[illegible]
Q9CWU9	Nucleoporin Nup37 OS=Mus musculus OX=10090 GN=Nup37 PE=1 SV=2	28	8	22	8	326	36,7	6,09	[illegible]
Q8CJ96	Ras association domain-containing protein 8 OS=Mus musculus OX=10090 G	7	2	2	2	419	48,1	5,95	[illegible]
P51163	Uroporphyrinogen-III synthase OS=Mus musculus OX=10090 GN=Uros PE=1	29	5	6	5	265	28,5	6,54	[illegible]
P29595	NEDD8 OS=Mus musculus OX=10090 GN=Nedd8 PE=1 SV=2	28	3	45	3	81	9	7,25	[illegible]
P81117	Nucleobindin-2 OS=Mus musculus OX=10090 GN=Nucb2 PE=1 SV=2	55	18	51	18	420	50,3	5,15	[illegible]
Q8BG95	Protein phosphatase 1 regulatory subunit 12B OS=Mus musculus OX=10090	7	6	9	4	976	109	5,74	[illegible]
P43277	Histone H1.3 OS=Mus musculus OX=10090 GN=H1-3 PE=1 SV=2	44	12	139	3	221	22,1	11,03	[illegible]
Q8BGC0	HIV Tat-specific factor 1 homolog OS=Mus musculus OX=10090 GN=Htatsf1	42	29	45	29	757	86,2	4,4	[illegible]
P08226	Apolipoprotein E OS=Mus musculus OX=10090 GN=Apoe PE=1 SV=2	44	13	22	13	311	35,8	5,68	[illegible]
Q8BL97	Serine/arginine-rich splicing factor 7 OS=Mus musculus OX=10090 GN=Srsf7	31	10	53	9	267	30,8	11,9	[illegible]
A0A0N4SUI7	Negative regulator of P-body association OS=Mus musculus OX=10090 GN=	78	4	9	4	68	7	9,42	[illegible]
Q6P7W2	SH3KBP1-binding protein 1 OS=Mus musculus OX=10090 GN=Shkbp1 PE=1 S	13	6	8	4	704	76,1	8,44	[illegible]
P08249	Malate dehydrogenase, mitochondrial OS=Mus musculus OX=10090 GN=M	70	22	612	22	338	35,6	8,68	[illegible]
Q6P5D8	Structural maintenance of chromosomes flexible hinge domain-containing pr	4	7	8	7	2007	225,5	7,24	[illegible]
Q9DB91	Mediator of RNA polymerase II transcription subunit 29 OS=Mus musculus O	8	2	3	2	199	21	6,29	[illegible]
Q9CY97	RNA polymerase II subunit A C-terminal domain phosphatase SSU72 OS=Mu	18	3	6	3	194	22,5	5,21	[illegible]
O35298	Acyloxyacyl hydrolase OS=Mus musculus OX=10090 GN=Aoah PE=1 SV=1	3	2	2	2	574	65,1	6,98	[illegible]
Q8C6E0	Cilia- and flagella-associated protein 36 OS=Mus musculus OX=10090 GN=Cf	23	7	8	7	343	39,6	4,93	[illegible]
Q9CQG3	Zinc finger MYND domain-containing protein 19 OS=Mus musculus OX=100	7	2	2	2	227	26,4	6,86	[illegible]
E9Q3Q6	Activated leukocyte cell adhesion molecule OS=Mus musculus OX=10090 GN	7	4	5	4	570	63,6	7,27	[illegible]
P62309	Small nuclear ribonucleoprotein G OS=Mus musculus OX=10090 GN=Snrpg P	37	5	37	5	76	8,5	8,88	[illegible]
P43276	Histone H1.5 OS=Mus musculus OX=10090 GN=H1-5 PE=1 SV=2	36	8	79	6	223	22,6	10,92	[illegible]
Q921G7	Electron transfer flavoprotein-ubiquinone oxidoreductase, mitochondrial OS=	9	5	5	5	616	68	7,58	[illegible]
Q9CRD2	ER membrane protein complex subunit 2 OS=Mus musculus OX=10090 GN=E	8	2	4	2	297	34,9	6,81	[illegible]
Q9CPY6	Glucose-induced degradation protein 4 homolog OS=Mus musculus OX=100	36	7	9	7	217	24,8	6,52	[illegible]
P62960	Y-box-binding protein 1 OS=Mus musculus OX=10090 GN=Ybx1 PE=1 SV=3	73	18	264	15	322	35,7	9,88	[illegible]
P30681	High mobility group protein B2 OS=Mus musculus OX=10090 GN=Hmgb2 PE=	61	16	114	15	210	24,1	7,31	[illegible]
A2AP74	CD99 antigen-like protein 2 OS=Mus musculus OX=10090 GN=Cd99l2 PE=1 S	15	2	2	2	216	23,2	4,93	[illegible]
A0A0R4J138	Arylsulfatase B OS=Mus musculus OX=10090 GN=Arsb PE=1 SV=1	18	10	19	10	534	59,7	7,14	[illegible]
O88531	Palmitoyl-protein thioesterase 1 OS=Mus musculus OX=10090 GN=Ppt1 PE=	30	6	9	6	306	34,5	8	[illegible]
Q9CYC5	Kinetochore-associated protein DSN1 homolog OS=Mus musculus OX=1009	6	2	2	2	348	39,5	6,21	[illegible]
P63158	High mobility group protein B1 OS=Mus musculus OX=10090 GN=Hmgb1 PE=	39	11	209	6	215	24,9	5,74	[illegible]
O54941	SWI/SNF-related matrix-associated actin-dependent regulator of chromatin s	42	13	26	12	411	46,6	4,88	[illegible]
P49813	Tropomodulin-1 OS=Mus musculus OX=10090 GN=Tmod1 PE=1 SV=2	22	7	11	6	359	40,4	5,1	[illegible]
Q9CQK7	RWD domain-containing protein 1 OS=Mus musculus OX=10090 GN=Rwdd1	17	6	14	6	243	27,8	4,26	[illegible]
D3YVY9	Sister chromatid cohesion protein DCC1 OS=Mus musculus OX=10090 GN=D	5	2	2	2	462	52	4,87	[illegible]
F8VPM7	ELKS/Rab6-interacting/CAST family member 1 OS=Mus musculus OX=1009	26	26	29	26	1120	128,3	5,91	[illegible]
Q99M51	Cytoplasmic protein NCK1 OS=Mus musculus OX=10090 GN=Nck1 PE=1 SV=	32	11	15	10	377	42,9	6,47	[illegible]
P14211	Calreticulin OS=Mus musculus OX=10090 GN=Calr PE=1 SV=1	77	37	526	37	416	48	4,49	[illegible]
Q9CZ13	Cytochrome b-c1 complex subunit 1, mitochondrial OS=Mus musculus OX=1	31	14	23	13	480	52,8	6,21	[illegible]
Q9CQE1	Protein NipSnap homolog 3B OS=Mus musculus OX=10090 GN=Nipsnap3b P	16	4	11	4	247	28,3	9,48	[illegible]
Q9ERF3	WD repeat-containing protein 61 OS=Mus musculus OX=10090 GN=Wdr61 P	47	13	64	13	305	33,8	5,36	[illegible]
A2AUT1	Kinetochore protein SPC25 (Fragment) OS=Mus musculus OX=10090 GN=Sp	20	4	4	4	133	15,7	8,03	[illegible]
Q3UMU9	Hepatoma-derived growth factor-related protein 2 OS=Mus musculus OX=1	31	21	45	20	669	74,2	8,66	[illegible]
O08601	Microsomal triglyceride transfer protein large subunit OS=Mus musculus OX	8	6	8	6	894	99	7,62	[illegible]
A6ZI44	Fructose-bisphosphate aldolase OS=Mus musculus OX=10090 GN=Aldoa PE=	82	34	913	11	418	45,1	7,91	[illegible]
P62878	E3 ubiquitin-protein ligase RBX1 OS=Mus musculus OX=10090 GN=Rbx1 PE=	25	3	17	3	108	12,3	6,96	[illegible]
P45377	Aldose reductase-related protein 2 OS=Mus musculus OX=10090 GN=Akr1b	51	12	39	5	316	36,1	6,37	[illegible]
E9PWW9	Remodeling and spacing factor 1 OS=Mus musculus OX=10090 GN=Rsf1 PE=	7	7	9	7	1441	161,7	5,02	[illegible]
Q9CR16	Peptidyl-prolyl cis-trans isomerase D OS=Mus musculus OX=10090 GN=Ppid	71	23	144	22	370	40,7	7,43	[illegible]
Q8CAK3	Shiftless antiviral inhibitor of ribosomal frameshifting protein homolog OS=M	31	8	12	8	290	33	7,28	[illegible]
P70665	Sialate O-acetylesterase OS=Mus musculus OX=10090 GN=Siae PE=1 SV=3	13	6	10	6	541	60,7	6,79	[illegible]
Q80V86	Integrator complex subunit 8 OS=Mus musculus OX=10090 GN=Ints8 PE=1 S	6	4	4	4	995	113,3	6,96	[illegible]
P17665	Cytochrome c oxidase subunit 7C, mitochondrial OS=Mus musculus OX=100	29	2	3	2	63	7,3	11	[illegible]
Q91YR7	Pre-mRNA-processing factor 6 OS=Mus musculus OX=10090 GN=Prpf6 PE=1	4	4	4	4	941	106,7	8,09	[illegible]
F6S742	RING-type E3 ubiquitin transferase (Fragment) OS=Mus musculus OX=1009	5	2	2	2	344	38	7,8	[illegible]
E9Q309	Centrosome-associated protein 350 OS=Mus musculus OX=10090 GN=Cep3	0	2	2	2	3095	346,2	6,35	[illegible]
P21460	Cystatin-C OS=Mus musculus OX=10090 GN=Cst3 PE=1 SV=2	39	5	22	5	140	15,5	9	[illegible]
P26350	Prothymosin alpha OS=Mus musculus OX=10090 GN=Ptma PE=1 SV=2	39	8	523	4	111	12,2	3,79	[illegible]
Q8K409	DNA polymerase beta OS=Mus musculus OX=10090 GN=Polb PE=1 SV=3	11	2	3	2	335	38,3	8,85	[illegible]
P06909	Complement factor H OS=Mus musculus OX=10090 GN=Cfh PE=1 SV=2	10	7	8	7	1234	139	6,99	[illegible]
Q9D1M0	Protein SEC13 homolog OS=Mus musculus OX=10090 GN=Sec13 PE=1 SV=3	57	13	59	13	322	35,5	5,38	[illegible]
Q99LI9	Polyribonucleotide 5'-hydroxyl-kinase Clp1 OS=Mus musculus OX=10090 GN	7	2	2	2	425	47,7	6,62	[illegible]
A0A2R8VK76	Cleavage and polyadenylation-specificity factor subunit 1 OS=Mus musculus	15	21	38	21	1450	161,7	6,58	[illegible]
Q60676	Serine/threonine-protein phosphatase 5 OS=Mus musculus OX=10090 GN=P	52	24	98	24	499	56,8	6,2	[illegible]
Q8BTW3	Exosome complex component MTR3 OS=Mus musculus OX=10090 GN=Exos	44	12	42	12	273	28,4	6,11	[illegible]
A0A2I3BRL8	Predicted gene 7324 OS=Mus musculus OX=10090 GN=Gm7324 PE=4 SV=1	41	18	32	3	388	42,2	9,99	[illegible]
P56212	cAMP-regulated phosphoprotein 19 OS=Mus musculus OX=10090 GN=Arpp	53	5	39	4	112	12,3	9,09	[illegible]
P10810	Monocyte differentiation antigen CD14 OS=Mus musculus OX=10090 GN=Cd	16	5	9	5	366	39,2	5,21	[illegible]
P50428	Arylsulfatase A OS=Mus musculus OX=10090 GN=Arsa PE=1 SV=2	8	5	7	4	506	53,7	5,87	[illegible]
Q71RI9	Kynurenine--oxoglutarate transaminase 3 OS=Mus musculus OX=10090 GN=	44	20	49	19	455	51,1	8,37	[illegible]
P04117	Fatty acid-binding protein, adipocyte OS=Mus musculus OX=10090 GN=Fab	56	8	66	8	132	14,6	8,4	[illegible]
Q99K95	Replication termination factor 2 OS=Mus musculus OX=10090 GN=Rtf2 PE=1	52	14	34	14	307	33,9	8,81	[illegible]
Q80X68	Citrate synthase OS=Mus musculus OX=10090 GN=Csl PE=1 SV=1	24	9	69	2	466	52,3	8,79	[illegible]
C0HKD8	Microfibrillar-associated protein 1A OS=Mus musculus OX=10090 GN=Mfap	56	19	35	19	439	51,9	4,98	[illegible]
Q64191	N(4)-(beta-N-acetylglucosaminyl)-L-asparaginase OS=Mus musculus OX=10	27	6	8	6	346	37	6,44	[illegible]
Q8C0I1	Alkyldihydroxyacetonephosphate synthase, peroxisomal OS=Mus musculus	27	13	16	13	645	71,6	7,5	[illegible]
D3Z5P5	DNA-binding protein SMUBP-2 OS=Mus musculus OX=10090 GN=Ighmbp2 P	3	3	3	3	885	97,4	8,05	[illegible]
Q62426	Cystatin-B OS=Mus musculus OX=10090 GN=Cstb PE=1 SV=1	80	7	212	7	98	11	7,39	[illegible]
Q8VE95	UPF0598 protein C8orf82 homolog OS=Mus musculus OX=10090 PE=1 SV=1	46	7	11	7	218	24,3	8,7	[illegible]
E9QM38	Solute carrier family 12 member 2 OS=Mus musculus OX=10090 GN=Slc12a	7	5	6	5	1206	130,6	6,52	[illegible]
Q99KR7	Peptidyl-prolyl cis-trans isomerase F, mitochondrial OS=Mus musculus OX=1	58	10	51	8	206	21,7	9,16	[illegible]
Q9CZN7	Serine hydroxymethyltransferase, mitochondrial OS=Mus musculus OX=100	72	32	407	31	504	55,7	8,47	[illegible]
Q60668	Heterogeneous nuclear ribonucleoprotein D0 OS=Mus musculus OX=10090	36	13	74	2	355	38,3	7,81	[illegible]
Q8C1M2	Zinc finger protein 428 OS=Mus musculus OX=10090 GN=Znf428 PE=1 SV=1	34	4	14	4	176	19	4,23	[illegible]
Q9D0A0	DET1 homolog OS=Mus musculus OX=10090 GN=Det1 PE=2 SV=2	4	2	2	2	550	63,8	7,56	[illegible]
Q8JZQ9	Eukaryotic translation initiation factor 3 subunit B OS=Mus musculus OX=10	47	33	112	33	803	91,3	5,02	[illegible]
Q99P91	Transmembrane glycoprotein NMB OS=Mus musculus OX=10090 GN=Gpnm	23	10	51	10	574	63,6	7,61	[illegible]
O70479	BTB/POZ domain-containing adapter for CUL3-mediated RhoA degradation p	19	5	6	3	316	36,1	7,84	[illegible]
Q8C129	Leucyl-cystinyl aminopeptidase OS=Mus musculus OX=10090 GN=Lnpep PE=	7	6	6	6	1025	117,2	5,96	[illegible]
P15105	Glutamine synthetase OS=Mus musculus OX=10090 GN=Glul PE=1 SV=6	40	12	16	12	373	42,1	7,08	[illegible]
Q3UG98	N-acetyltransferase 9 OS=Mus musculus OX=10090 GN=Nat9 PE=1 SV=2	9	2	3	2	241	27,6	5,27	[illegible]
Q63870	Collagen alpha-1(VII) chain OS=Mus musculus OX=10090 GN=Col7a1 PE=1 S	1	2	3	2	2944	295,1	6,34	[illegible]
Q68FG3	Protein SPT2 homolog OS=Mus musculus OX=10090 GN=Spty2d1 PE=2 SV=	4	3	4	3	682	74,8	9,76	[illegible]
Q8VDY9	Caspase activity and apoptosis inhibitor 1 OS=Mus musculus OX=10090 GN=	33	7	9	7	356	37,8	4,72	[illegible]
Q5RKZ7	Molybdenum cofactor biosynthesis protein 1 OS=Mus musculus OX=10090	13	7	10	7	636	69,8	9,14	[illegible]
A2AFQ2	3-hydroxyacyl-CoA dehydrogenase type-2 OS=Mus musculus OX=10090 GN	69	13	91	13	271	28,4	8,76	[illegible]
Q80SY5	Pre-mRNA-splicing factor 38B OS=Mus musculus OX=10090 GN=Prpf38b PE=	14	7	8	7	542	63,7	10,54	[illegible]
Q6IR42	Zinc finger CW-type PWWP domain protein 1 OS=Mus musculus OX=10090	13	6	6	6	630	70,5	5,45	[illegible]
Q3UHX2	28 kDa heat- and acid-stable phosphoprotein OS=Mus musculus OX=10090	57	13	98	13	181	20,6	7,39	[illegible]
P62962	Profilin-1 OS=Mus musculus OX=10090 GN=Pfn1 PE=1 SV=2	96	13	433	7	140	14,9	8,28	[illegible]
Q91YN9	BAG family molecular chaperone regulator 2 OS=Mus musculus OX=10090 G	55	11	25	11	210	23,5	6,42	[illegible]
Q9CQ49	Nuclear cap-binding protein subunit 2 OS=Mus musculus OX=10090 GN=Ncb	46	8	20	8	156	18	7,81	[illegible]
Q9EQ32	Phosphoinositide 3-kinase adapter protein 1 OS=Mus musculus OX=10090 G	6	3	3	3	811	90,9	5,17	[illegible]
Q8BQ46	TAF15 RNA polymerase II, TATA box binding protein (TBP)-associated factor	38	12	60	9	557	58,6	8,4	[illegible]
Q8BFQ4	WD repeat-containing protein 82 OS=Mus musculus OX=10090 GN=Wdr82 P	37	9	15	9	313	35,1	7,69	[illegible]
O08749	Dihydrolipoyl dehydrogenase, mitochondrial OS=Mus musculus OX=10090 G	44	18	167	18	509	54,2	7,9	[illegible]
Q8BG05	Heterogeneous nuclear ribonucleoprotein A3 OS=Mus musculus OX=10090	45	16	105	14	379	39,6	9,01	[illegible]
B1AU75	Nuclear autoantigenic sperm protein OS=Mus musculus OX=10090 GN=Nasp	72	45	297	21	773	84	4,37	[illegible]
Q99JV5	StAR-related lipid transfer protein 4 OS=Mus musculus OX=10090 GN=Stard	19	3	3	3	224	25,6	5,67	[illegible]
O09131	Glutathione S-transferase omega-1 OS=Mus musculus OX=10090 GN=Gsto1	68	20	170	20	240	27,5	7,36	[illegible]
Q8BFQ1	Prosaposin OS=Mus musculus OX=10090 GN=Psap PE=1 SV=1	48	23	103	23	554	61	5,24	[illegible]
Q923G2	DNA-directed RNA polymerases I, II, and III subunit RPABC3 OS=Mus muscu	72	8	26	8	150	17,1	4,68	[illegible]
F8WIT2	Annexin OS=Mus musculus OX=10090 GN=Anxa6 PE=1 SV=1	51	34	85	33	667	75,2	5,55	[illegible]
Q8R0N6	Hydroxyacid-oxoacid transhydrogenase, mitochondrial OS=Mus musculus O	13	6	10	6	465	49,9	7,58	[illegible]
Q9D6H2	Intraflagellar transport protein 25 homolog OS=Mus musculus OX=10090 GN	31	4	7	4	143	16,3	5,16	[illegible]
Q9CPV5	Polyamine-modulated factor 1 OS=Mus musculus OX=10090 GN=Pmf1 PE=1	51	10	18	10	202	23,1	5,26	[illegible]
P11404	Fatty acid-binding protein, heart OS=Mus musculus OX=10090 GN=Fabp3 PE	70	9	31	9	133	14,8	6,57	[illegible]
Q9Z2X2	26S proteasome non-ATPase regulatory subunit 10 OS=Mus musculus OX=1	42	7	9	7	231	25,1	6,06	[illegible]
Q8VEB4	Phospholipase A2 group XV OS=Mus musculus OX=10090 GN=Pla2g15 PE=1	19	8	17	8	412	47,3	6,47	[illegible]
Q6PCM1	Lysine-specific demethylase 3A OS=Mus musculus OX=10090 GN=Kdm3a PE	2	2	2	2	1323	147,8	7,74	[illegible]
Q6R891	Neurabin-2 OS=Mus musculus OX=10090 GN=Ppp1r9b PE=1 SV=1	16	11	17	11	817	89,5	4,92	[illegible]
D3Z512	Deoxyribonuclease II OS=Mus musculus OX=10090 GN=Dnase2a PE=1 SV=1	22	6	13	6	332	36,5	7,77	[illegible]
P83917	Chromobox protein homolog 1 OS=Mus musculus OX=10090 GN=Cbx1 PE=1	66	12	46	11	185	21,4	4,93	[illegible]
Q8K3G5	Inactive serine/threonine-protein kinase VRK3 OS=Mus musculus OX=1009	4	2	2	2	453	50,8	8,57	[illegible]
Q9EST3	Eukaryotic translation initiation factor 4E transporter OS=Mus musculus OX=	4	3	4	3	983	107,9	7,74	[illegible]
P42125	Enoyl-CoA delta isomerase 1, mitochondrial OS=Mus musculus OX=10090 G	33	9	45	9	289	32,2	8,98	[illegible]
P97864	Caspase-7 OS=Mus musculus OX=10090 GN=Casp7 PE=1 SV=2	20	6	7	6	303	34	6,33	[illegible]
Q921W4	Quinone oxidoreductase-like protein 1 OS=Mus musculus OX=10090 GN=Cry	37	11	18	11	348	38,7	5,97	[illegible]
A0A0R4J0U2	Serine/threonine-protein phosphatase 4 regulatory subunit 2 OS=Mus muscu	75	20	64	20	417	46,4	4,56	[illegible]
P23780	Beta-galactosidase OS=Mus musculus OX=10090 GN=Glb1 PE=1 SV=1	24	13	38	12	647	73,1	7,47	[illegible]
Q9DB05	Alpha-soluble NSF attachment protein OS=Mus musculus OX=10090 GN=Na	72	17	40	17	295	33,2	5,45	[illegible]
P24527	Leukotriene A-4 hydrolase OS=Mus musculus OX=10090 GN=Lta4h PE=1 SV	60	31	139	31	611	69	6,42	[illegible]
Q9QZ73	DCN1-like protein 1 OS=Mus musculus OX=10090 GN=Dcun1d1 PE=2 SV=1	25	6	11	4	259	30,1	5,34	[illegible]
A0A0R4J1S4	SLAM family member 5 OS=Mus musculus OX=10090 GN=Cd84 PE=1 SV=1	22	3	5	3	140	16,1	5,54	[illegible]
G5E8R8	UBX domain-containing protein 7 OS=Mus musculus OX=10090 GN=Ubxn7 P	36	15	25	15	489	54,8	5,16	[illegible]
A0A0R4J0P1	Isobutyryl-CoA dehydrogenase, mitochondrial OS=Mus musculus OX=1009	38	14	23	14	413	45,1	8,02	[illegible]
Q9CZ44	NSFL1 cofactor p47 OS=Mus musculus OX=10090 GN=Nsfl1c PE=1 SV=1	59	24	114	24	370	40,7	5,15	[illegible]
G3UZY2	Thioredoxin, mitochondrial (Fragment) OS=Mus musculus OX=10090 GN=Tx	52	5	20	5	130	14,2	5,81	[illegible]
Q99JR1	Sideroflexin-1 OS=Mus musculus OX=10090 GN=Sfxn1 PE=1 SV=3	22	6	11	6	322	35,6	9,23	[illegible]
G3X8X7	Vacuolar protein sorting-associated protein 16 homolog OS=Mus musculus C	5	4	4	4	839	94,8	6,92	[illegible]
J3QN89	Angio-associated migratory protein OS=Mus musculus OX=10090 GN=Aamp	40	13	39	13	436	46,9	4,41	[illegible]
Q9CPY7	Cytosol aminopeptidase OS=Mus musculus OX=10090 GN=Lap3 PE=1 SV=3	66	32	238	32	519	56,1	7,72	[illegible]
Q9D6Y7	Mitochondrial peptide methionine sulfoxide reductase OS=Mus musculus OX	18	4	7	4	233	26	8,41	[illegible]
Q9CQV8	14-3-3 protein beta/alpha OS=Mus musculus OX=10090 GN=Ywhab PE=1 SV	60	12	250	6	246	28,1	4,83	[illegible]
F8WJ43	Merlin (Fragment) OS=Mus musculus OX=10090 GN=Nf2 PE=1 SV=1	7	5	6	5	521	61	6,1	[illegible]
Q3UMG6	Nucleoside diphosphate kinase 7 OS=Mus musculus OX=10090 GN=Nme7 P	14	4	6	4	395	44,4	7,12	[illegible]
Q9JJA4	Ribosome biogenesis protein WDR12 OS=Mus musculus OX=10090 GN=Wdr	49	16	45	16	423	47,3	5,6	[illegible]
Q91V76	Ester hydrolase C11orf54 homolog OS=Mus musculus OX=10090 PE=1 SV=1	46	13	22	13	315	35	6,29	[illegible]
P62806	Histone H4 OS=Mus musculus OX=10090 GN=H4c1 PE=1 SV=2	40	5	61	5	103	11,4	11,36	[illegible]
Q01853	Transitional endoplasmic reticulum ATPase OS=Mus musculus OX=10090 GN	77	58	659	57	806	89,3	5,26	[illegible]
Q9QZU9	Ubiquitin/ISG15-conjugating enzyme E2 L6 OS=Mus musculus OX=10090 GN	16	4	8	4	153	17,8	8,09	[illegible]
Q9D0K2	Succinyl-CoA:3-ketoacid coenzyme A transferase 1, mitochondrial OS=Mus	45	16	98	16	520	56	8,53	[illegible]
Q3UKC1	Tax1-binding protein 1 homolog OS=Mus musculus OX=10090 GN=Tax1bp1	19	15	16	15	814	93,6	5,33	[illegible]
Q9CQ65	S-methyl-5'-thioadenosine phosphorylase OS=Mus musculus OX=10090 GN=	81	18	134	18	283	31	7,14	[illegible]
A0A0R4J098	Zinc finger protein 326 OS=Mus musculus OX=10090 GN=Zfp326 PE=1 SV=1	7	2	2	2	580	65,1	5,21	[illegible]
Q9QZN4	F-box only protein 6 OS=Mus musculus OX=10090 GN=Fbxo6 PE=1 SV=1	22	5	8	5	295	34,5	8,18	[illegible]
Q8BZJ7	DCN1-like protein 2 OS=Mus musculus OX=10090 GN=Dcun1d2 PE=1 SV=3	19	4	7	2	259	30	5,59	[illegible]
P84104	Serine/arginine-rich splicing factor 3 OS=Mus musculus OX=10090 GN=Srsf3	35	7	73	6	164	19,3	11,65	[illegible]
P29758	Ornithine aminotransferase, mitochondrial OS=Mus musculus OX=10090 GN	64	19	152	19	439	48,3	6,62	[illegible]
Q8R242	Di-N-acetylchitobiase OS=Mus musculus OX=10090 GN=Ctbs PE=1 SV=2	30	9	18	9	366	41,3	5,6	[illegible]
Q921M3	Splicing factor 3B subunit 3 OS=Mus musculus OX=10090 GN=Sf3b3 PE=1 SV	43	48	206	48	1217	135,5	5,26	[illegible]
Q9Z2U1	Proteasome subunit alpha type-5 OS=Mus musculus OX=10090 GN=Psma5	65	11	101	8	241	26,4	4,79	[illegible]
P27661	Histone H2AX OS=Mus musculus OX=10090 GN=H2ax PE=1 SV=2	27	5	53	2	143	15,1	10,74	[illegible]
Q8K2H2	Deubiquitinase OTUD6B OS=Mus musculus OX=10090 GN=Otud6b PE=1 SV=	26	6	7	6	294	33,7	5,53	[illegible]
O35969	Guanidinoacetate N-methyltransferase OS=Mus musculus OX=10090 GN=Ga	25	3	4	3	236	26,3	5,71	[illegible]
P10518	Delta-aminolevulinic acid dehydratase OS=Mus musculus OX=10090 GN=Ala	35	10	44	10	330	36	6,79	[illegible]
Q3THJ3	Probable RNA-binding protein EIF1AD OS=Mus musculus OX=10090 GN=Eif1	49	7	25	7	170	19,5	4,87	[illegible]
Q9D3D9	ATP synthase subunit delta, mitochondrial OS=Mus musculus OX=10090 GN	14	2	10	2	168	17,6	5,08	[illegible]
P28667	MARCKS-related protein OS=Mus musculus OX=10090 GN=Marcksl1 PE=1 S	82	9	39	9	200	20,2	4,61	[illegible]
Q9EQK5	Major vault protein OS=Mus musculus OX=10090 GN=Mvp PE=1 SV=4	32	23	51	23	861	95,9	5,59	[illegible]
P11352	Glutathione peroxidase 1 OS=Mus musculus OX=10090 GN=Gpx1 PE=1 SV=2	64	10	20	10	201	22,3	7,21	[illegible]
Q8C845	EF-hand domain-containing protein D2 OS=Mus musculus OX=10090 GN=Ef	70	17	126	17	240	26,8	5,14	[illegible]
E9PZF0	Nucleoside diphosphate kinase OS=Mus musculus OX=10090 GN=Gm20390	83	16	292	7	267	30,2	8,72	[illegible]
D3YWR7	Dihydropteridine reductase OS=Mus musculus OX=10090 GN=Qdpr PE=1 SV	77	12	89	2	189	20	6,92	[illegible]
Q62189	U1 small nuclear ribonucleoprotein A OS=Mus musculus OX=10090 GN=Snrp	42	10	72	8	287	31,8	9,8	[illegible]
O70201	Baculoviral IAP repeat-containing protein 5 OS=Mus musculus OX=10090 GN	17	2	3	2	140	16,3	5,6	[illegible]
Q09200	Beta-1,4 N-acetylgalactosaminyltransferase 1 OS=Mus musculus OX=10090	7	4	4	4	533	59,2	8,59	[illegible]
A0A0R4J092	Beta-mannosidase OS=Mus musculus OX=10090 GN=Manba PE=1 SV=1	31	24	54	24	879	100,8	7,08	[illegible]
A2BGN7	Protein MANBAL OS=Mus musculus OX=10090 GN=Manbal PE=1 SV=1	24	2	2	2	85	9,4	9,38	[illegible]
Q9Z1D1	Eukaryotic translation initiation factor 3 subunit G OS=Mus musculus OX=10	66	19	75	19	320	35,6	5,9	[illegible]
J3QJX3	Protein sel-1 homolog 1 OS=Mus musculus OX=10090 GN=Sel1l PE=1 SV=1	4	2	3	2	740	82,4	6,02	[illegible]
Q99020	Heterogeneous nuclear ribonucleoprotein A/B OS=Mus musculus OX=10090	48	15	186	14	285	30,8	7,91	[illegible]
Q3U0V2	Tumor necrosis factor receptor type 1-associated DEATH domain protein OS=	16	3	3	3	310	34,6	5,2	[illegible]
P61022	Calcineurin B homologous protein 1 OS=Mus musculus OX=10090 GN=Chp1	26	4	4	4	195	22,4	5,1	[illegible]
Q9DB20	ATP synthase subunit O, mitochondrial OS=Mus musculus OX=10090 GN=At	27	4	7	4	213	23,3	9,99	[illegible]
Q00PI9	Heterogeneous nuclear ribonucleoprotein U-like protein 2 OS=Mus musculus	38	24	40	24	745	84,9	4,89	[illegible]
Q3THW5	Histone H2A.V OS=Mus musculus OX=10090 GN=H2az2 PE=1 SV=3	31	4	45	2	128	13,5	10,58	[illegible]
Q8K1C0	Protein angel homolog 2 OS=Mus musculus OX=10090 GN=Angel2 PE=1 SV=	10	5	8	5	544	62,4	8,21	[illegible]
Q99L27	GMP reductase 2 OS=Mus musculus OX=10090 GN=Gmpr2 PE=1 SV=2	51	15	45	13	348	38	7,44	[illegible]
Q571E4	N-acetylgalactosamine-6-sulfatase OS=Mus musculus OX=10090 GN=Galns	21	11	18	10	520	57,6	6,52	[illegible]
Q8BP48	Methionine aminopeptidase 1 OS=Mus musculus OX=10090 GN=Metap1 PE	69	19	69	19	386	43,2	7,17	[illegible]
Q9D6W8	BLOC-1-related complex subunit 6 OS=Mus musculus OX=10090 GN=Borcs6	11	2	3	2	360	38	5,34	[illegible]
Q9DAV6	R86 OS=Mus musculus OX=10090 GN=Serpinb9b PE=1 SV=1	7	3	4	2	377	42,7	6,67	[illegible]
Q8VEJ4	Notchless protein homolog 1 OS=Mus musculus OX=10090 GN=Nle1 PE=1 SV	45	16	58	16	485	53,1	7,17	[illegible]
P53368	Oxidized purine nucleoside triphosphate hydrolase OS=Mus musculus OX=1	26	4	5	4	156	17,9	5,53	[illegible]
O35657	Sialidase-1 OS=Mus musculus OX=10090 GN=Neu1 PE=1 SV=1	32	11	20	11	409	44,6	6,02	[illegible]
Q8K370	Acyl-CoA dehydrogenase family member 10 OS=Mus musculus OX=10090	8	8	8	4	1069	118,9	8,24	[illegible]
O88892	Small EDRK-rich factor 1 OS=Mus musculus OX=10090 GN=Serf1 PE=1 SV=1	42	4	16	3	62	7,3	10,45	[illegible]
P36552	Oxygen-dependent coproporphyrinogen-III oxidase, mitochondrial OS=Mus	42	16	43	16	443	49,7	8,53	[illegible]
P28798	Progranulin OS=Mus musculus OX=10090 GN=Grn PE=1 SV=2	54	20	62	20	589	63,4	6,8	[illegible]
Q5SWN2	Replication protein A subunit OS=Mus musculus OX=10090 GN=Rpa1 PE=1 S	23	13	30	13	644	71,4	8,18	[illegible]
G3X926	Ribosome production factor 2 homolog OS=Mus musculus OX=10090 GN=Rp	30	7	14	7	273	31,5	9,98	[illegible]
A0A3B2WB50	EMILIN-2 OS=Mus musculus OX=10090 GN=Emilin2 PE=1 SV=1	12	9	11	9	993	108,4	5,38	[illegible]
Q9CZ42	ATP-dependent (S)-NAD(P)H-hydrate dehydratase OS=Mus musculus OX=1	44	11	31	11	343	36,7	7,77	[illegible]
O55234	Proteasome subunit beta type-5 OS=Mus musculus OX=10090 GN=Psmb5 P	57	14	108	14	264	28,5	7,02	[illegible]
Q64520	Guanylate kinase OS=Mus musculus OX=10090 GN=Guk1 PE=1 SV=2	50	9	18	9	198	21,9	6,55	[illegible]
P62317	Small nuclear ribonucleoprotein Sm D2 OS=Mus musculus OX=10090 GN=Sn	62	9	123	9	118	13,5	9,91	[illegible]
Q8K2Q9	Shootin-1 OS=Mus musculus OX=10090 GN=Shtn1 PE=1 SV=1	66	39	79	39	631	71,3	5,44	[illegible]
Q61035	Histidine--tRNA ligase, cytoplasmic OS=Mus musculus OX=10090 GN=Hars1	47	29	87	26	509	57,4	6	[illegible]
Q61818	Retinoic acid-induced protein 1 OS=Mus musculus OX=10090 GN=Rai1 PE=1	1	3	3	2	1889	201,4	8,75	[illegible]
Q6ZWY8	Thymosin beta-10 OS=Mus musculus OX=10090 GN=Tmsb10 PE=1 SV=3	66	4	45	2	44	5	5,36	[illegible]
Q3TCN2	Putative phospholipase B-like 2 OS=Mus musculus OX=10090 GN=Plbd2 PE=	24	13	29	13	594	66,2	6,13	[illegible]
Q8CFG0	Extracellular sulfatase Sulf-2 OS=Mus musculus OX=10090 GN=Sulf2 PE=2 S	5	3	3	3	875	100,4	9,07	[illegible]
Q9EQU5	Protein SET OS=Mus musculus OX=10090 GN=Set PE=1 SV=1	51	17	347	2	289	33,4	4,32	[illegible]
Q00547	Hyaluronan mediated motility receptor OS=Mus musculus OX=10090 GN=Hr	35	20	30	20	794	91,7	5,52	[illegible]
Q8BJ48	N-acetylglucosamine-1-phosphodiester alpha-N-acetylglucosaminidase OS=	12	5	6	5	517	56	5,55	[illegible]
Q3UWE6	WD repeat-containing protein 20 OS=Mus musculus OX=10090 GN=Wdr20 P	7	3	3	3	569	62,8	8	[illegible]
A2A7P9	Small vasohibin-binding protein OS=Mus musculus OX=10090 GN=Svbp PE=	29	3	7	3	102	12	9,86	[illegible]
P62983	Ubiquitin-40S ribosomal protein S27a OS=Mus musculus OX=10090 GN=Rps	67	16	484	3	156	17,9	9,64	[illegible]
P12265	Beta-glucuronidase OS=Mus musculus OX=10090 GN=Gusb PE=1 SV=2	33	19	74	19	648	74,1	6,7	[illegible]
O08807	Peroxiredoxin-4 OS=Mus musculus OX=10090 GN=Prdx4 PE=1 SV=1	48	14	117	12	274	31	7,15	[illegible]
Q62241	U1 small nuclear ribonucleoprotein C OS=Mus musculus OX=10090 GN=Snrp	25	3	14	3	159	17,4	9,67	[illegible]
Q8CCI5	RING1 and YY1-binding protein OS=Mus musculus OX=10090 GN=Rybp PE=1	44	7	13	6	228	24,8	9,63	[illegible]
Q8C5N5	Programmed cell death protein 2-like OS=Mus musculus OX=10090 GN=Pdc	8	3	3	3	364	39,9	4,68	[illegible]
Q8C0L6	Peroxisomal N(1)-acetyl-spermine/spermidine oxidase OS=Mus musculus O	34	12	27	12	504	55,4	5,11	[illegible]
H7BWY8	Pre-rRNA-processing protein TSR2 homolog OS=Mus musculus OX=10090 GN	48	6	30	2	195	21,4	4,26	[illegible]
Q811J3	Iron-responsive element-binding protein 2 OS=Mus musculus OX=10090 GN	7	8	11	7	963	104,9	6,93	[illegible]
A0A1W2P7Y9	Neuronal cell adhesion molecule OS=Mus musculus OX=10090 GN=Nrcam P	2	2	3	2	1134	125,4	6,38	[illegible]
P30416	Peptidyl-prolyl cis-trans isomerase FKBP4 OS=Mus musculus OX=10090 GN=	66	31	200	31	458	51,5	5,72	[illegible]
P15532	Nucleoside diphosphate kinase A OS=Mus musculus OX=10090 GN=Nme1 P	84	12	205	3	152	17,2	7,37	[illegible]
G3X961	Kelch domain-containing protein 4 OS=Mus musculus OX=10090 GN=Klhdc4	36	18	60	18	584	64,8	5,66	[illegible]
Q8CFE6	Sodium-coupled neutral amino acid transporter 2 OS=Mus musculus OX=10	9	2	4	2	504	55,5	7,94	[illegible]
P20060	Beta-hexosaminidase subunit beta OS=Mus musculus OX=10090 GN=Hexb P	43	23	64	23	536	61,1	8,12	[illegible]
Q793I8	TRAF-interacting protein with FHA domain-containing protein A OS=Mus mu	30	5	15	5	184	21,5	5	[illegible]
Q8R2S9	Actin-related protein 8 OS=Mus musculus OX=10090 GN=Actr8 PE=2 SV=1	4	2	2	2	624	70,5	7,77	[illegible]
O88851	Putative hydrolase RBBP9 OS=Mus musculus OX=10090 GN=Rbbp9 PE=1 SV	54	7	11	7	186	20,9	5,97	[illegible]
Q8K2I1	Protein farnesyltransferase subunit beta OS=Mus musculus OX=10090 GN=F	17	4	5	4	437	48,8	5,8	[illegible]
P24369	Peptidyl-prolyl cis-trans isomerase B OS=Mus musculus OX=10090 GN=Ppib	57	13	121	12	216	23,7	9,55	[illegible]
Q91WT4	DnaJ homolog subfamily C member 17 OS=Mus musculus OX=10090 GN=Dr	27	7	12	7	303	34,4	8,57	[illegible]
B2C3G8	Signal transducer and activator of transcription OS=Mus musculus OX=1009	4	3	3	3	797	91,6	7,68	[illegible]
A0A0R4J2B6	Retinoblastoma-binding protein 5 OS=Mus musculus OX=10090 GN=Rbbp5 F	43	21	58	21	537	59	5,1	[illegible]

P56960	Exosome component 10 OS=Mus musculus OX=10090 GN=Exosc10 PE=1 SV	7	6	8	6	887	100,9	8,29	cell organization and biogenesis;RNA metabolism OR transcription;other metaboli	cytosol;nucleus;other cell component	nucleic acid binding activity;other molecular function	Pf00570, Pf01612, Pf08066
O70469	Docking protein 2 OS=Mus musculus OX=10090 GN=Dok2 PE=1 SV=1	10	4	5	4	412	45,5	6,16	signal transduction;other biological processes		signal transduction activity or receptor binding	Pf00169, Pf02174
Q99LL5	Periodic tryptophan protein 1 homolog OS=Mus musculus OX=10090 GN=Pw	31	11	16	11	501	55,6	4,82	protein metabolism;RNA metabolism OR transcription;other metabolic processes;	ER/Golgi;nucleus;other cell component	other molecular function	Pf00400
Q6PGH1	Protein BUD31 homolog OS=Mus musculus OX=10090 GN=Bud31 PE=1 SV=2	54	7	30	7	144	17	8,82	RNA metabolism OR transcription;other metabolic processes;other biological proc	nucleus;other cell component	other molecular function	Pf01125
Q8BGV0	Probable asparagine--tRNA ligase, mitochondrial OS=Mus musculus OX=100	16	7	8	7	477	53,9	7,36	protein metabolism;RNA metabolism OR transcription;other metabolic processes	cytosol;mitochondrion;nucleus	nucleic acid binding activity;other molecular function	Pf00152, Pf01336
P67984	60S ribosomal protein L22 OS=Mus musculus OX=10090 GN=Rpl22 PE=1 SV=	35	4	81	4	128	14,8	9,19	protein metabolism;other metabolic processes;developmental processes;other bi	cytosol;translational apparatus;nucleus;other cell component	nucleic acid binding activity;other molecular function	Pf01776
Q3UMT1	Protein phosphatase 1 regulatory subunit 12C OS=Mus musculus OX=10090	19	11	13	11	782	84,6	6	signal transduction;other biological processes	cytoskeleton;other cell component	enzyme regulator activity;other molecular function	Pf12796, Pf15898
Q9R0Q7	Prostaglandin E synthase 3 OS=Mus musculus OX=10090 GN=Ptges3 PE=1 S	51	8	78	8	160	18,7	4,55	cell cycle OR cell proliferation;cell organization and biogenesis;DNA metabolism;o	cytosol;cytoskeleton;nucleus;other cell component	other molecular function	Pf04969
P29452	Caspase-1 OS=Mus musculus OX=10090 GN=Casp1 PE=1 SV=1	13	5	6	5	402	45,6	6,04	protein metabolism;other metabolic processes;stress response;transport;signal tr	non-structural extracellular;plasma membrane;other membranes;cytosol;mitochondrion;nucleus;o	other molecular function	Pf00619, Pf00656
Q9JKX4	Protein AATF OS=Mus musculus OX=10090 GN=Aatf PE=1 SV=1	8	4	6	3	526	59,4	4,93	cell adhesion;stress response;developmental processes;other biological processe	cytosol;ER/Golgi;nucleus;other cell component	cytoskeletal activity;other molecular function	Pf08164, Pf13339
A0A1D5RLS3	Calpain-15 OS=Mus musculus OX=10090 GN=Capn15 PE=1 SV=1	4	3	3	3	1196	128,9	6,92	protein metabolism		other molecular function	Pf00641, Pf00648
Q9JHT5	AMME syndrome candidate gene 1 protein homolog OS=Mus musculus OX=	19	6	12	6	344	36	8,63		mitochondrion;nucleus		Pf01871
Q810D6	Glutamate-rich WD repeat-containing protein 1 OS=Mus musculus OX=1009	42	12	22	12	446	49,2	4,81	cell organization and biogenesis;other metabolic processes	cytosol;nucleus;other cell component	nucleic acid binding activity;other molecular function	Pf00400, Pf12265
Q9CPR1	RWD domain-containing protein 4 OS=Mus musculus OX=10090 GN=Rwdd4	27	5	8	5	188	21,1	5,63				Pf05773
G5E829	Plasma membrane calcium-transporting ATPase 1 OS=Mus musculus OX=10	10	8	10	8	1220	134,7	5,91	stress response;transport;developmental processes;other biological processes	plasma membrane;other membranes;ER/Golgi;nucleus;other cell component	transporter activity;other molecular function	Pf00122, Pf00689, Pf00690, Pf00702, Pf1
P30412	Peptidyl-prolyl cis-trans isomerase C OS=Mus musculus OX=10090 GN=Ppic	27	5	59	4	212	22,8	7,5	protein metabolism;other metabolic processes;other biological processes	other cell component	other molecular function	Pf00160
E9Q7U2	Calcium-binding and coiled-coil domain-containing protein 1 OS=Mus muscul	17	11	13	11	633	70,9	4,86				Pf07888, Pf17751
Q64669	NAD(P)H dehydrogenase [quinone] 1 OS=Mus musculus OX=10090 GN=Nqo	10	3	3	3	274	30,9	8,72	other metabolic processes;stress response;other biological processes	cytosol;other cell component	other molecular function	Pf02525
Q9WVL3	Solute carrier family 12 member 7 OS=Mus musculus OX=10090 GN=Slc12a	2	2	2	2	1083	119,4	6,51	cell-cell signaling;cell organization and biogenesis;transport;other biological proce	plasma membrane;other membranes;other cell component	transporter activity;other molecular function	Pf00324, Pf03522
Q921K9	B-cell CLL/lymphoma 7 protein family member B OS=Mus musculus OX=100	28	6	17	3	202	22,2	4,75	cell-cell signaling;signal transduction;other biological processes			Pf04714
A0A0R4J107	Acyl-peptide hydrolase (Fragment) OS=Mus musculus OX=10090 GN=Apeh	47	25	86	25	716	79,8	5,59	protein metabolism		other molecular function	Pf00326, Pf19283
Q9Z2U0	Proteasome subunit alpha type-7 OS=Mus musculus OX=10090 GN=Psma7	62	15	159	15	248	27,8	8,46	protein metabolism;other metabolic processes	cytosol;nucleus;other cell component	other molecular function	Pf00227, Pf10584
Q9CQE8	RNA transcription, translation and transport factor protein OS=Mus musculus	48	11	34	11	244	28,1	6,89	RNA metabolism OR transcription;other metabolic processes;other biological proc	cytosol;cytoskeleton;nucleus;other cell component	nucleic acid binding activity;other molecular function	Pf10036
Q7TQ95	Endoplasmic reticulum junction formation protein lunapark OS=Mus musculu	16	4	4	4	425	47,5	5,27	cell organization and biogenesis;stress response;developmental processes;other	other membranes;ER/Golgi;nucleus	other molecular function	Pf10058
P57725	SAM domain-containing protein SAMSN-1 OS=Mus musculus OX=10090 GN	35	10	11	10	372	41,6	5,25	other biological processes	cytosol;nucleus;other cell component	other molecular function	Pf07647, Pf07653, Pf12485
P05202	Aspartate aminotransferase, mitochondrial OS=Mus musculus OX=10090 GN	66	27	276	27	430	47,4	9	other metabolic processes;transport;developmental processes;other biological pr	plasma membrane;other membranes;mitochondrion;other cell component	other molecular function	Pf00155
P18181	CD48 antigen OS=Mus musculus OX=10090 GN=Cd48 PE=1 SV=1	19	4	4	4	240	27,4	7,69	signal transduction;other biological processes	plasma membrane;other membranes;other cell component	other molecular function	Pf07686
P59108	Copine-2 OS=Mus musculus OX=10090 GN=Cpne2 PE=1 SV=1	13	5	7	4	548	61	5,96	other biological processes	plasma membrane;other membranes;nucleus;other cell component	other molecular function	Pf00168, Pf07002
Q9D7X3	Dual specificity protein phosphatase 3 OS=Mus musculus OX=10090 GN=Dus	59	6	10	5	185	20,5	6,54	protein metabolism;other metabolic processes;developmental processes;other bi	plasma membrane;cytosol;nucleus	signal transduction activity or receptor binding;cytoskeletal activity;other molec	Pf00782
P00493	Hypoxanthine-guanine phosphoribosyltransferase OS=Mus musculus OX=10	55	10	90	10	218	24,6	6,68	cell cycle OR cell proliferation;cell organization and biogenesis;other metabolic pr	cytosol;other cell component	other molecular function	Pf00156
P09671	Superoxide dismutase [Mn], mitochondrial OS=Mus musculus OX=10090 GN	42	10	89	10	222	24,6	8,62	cell organization and biogenesis;other metabolic processes;stress response;devel	mitochondrion;other cell component	nucleic acid binding activity;other molecular function	Pf00081, Pf02777
Q6PE01	U5 small nuclear ribonucleoprotein 40 kDa protein OS=Mus musculus OX=10	57	14	34	14	358	39,3	8,1	RNA metabolism OR transcription;other metabolic processes	cytosol;nucleus		Pf00400
Q9JKF6	Nectin-1 OS=Mus musculus OX=10090 GN=Nectin1 PE=1 SV=3	11	6	7	6	515	57	6,35	cell adhesion;cell organization and biogenesis;transport;developmental processe	plasma membrane;other membranes;other cell component	other molecular function	Pf07686, Pf08205, Pf13927
Q9D7J9	Enoyl-CoA hydratase domain-containing protein 3, mitochondrial OS=Mus m	33	8	18	8	300	32,4	8,34	other metabolic processes;other biological processes		other molecular function	Pf00378
Q8VDI7	Ubiquitin-associated domain-containing protein 1 OS=Mus musculus OX=100	23	7	10	7	409	45,5	4,97	protein metabolism;other metabolic processes	plasma membrane;other membranes;cytosol;ER/Golgi		Pf00627
P26043	Radixin OS=Mus musculus OX=10090 GN=Rdx PE=1 SV=3	50	38	207	21	583	68,5	6,2	cell organization and biogenesis;other biological processes	plasma membrane;other membranes;cytosol;cytoskeleton;other cell component	cytoskeletal activity;other molecular function	Pf00373, Pf00769, Pf09379, Pf09380, Pf2
O09159	Lysosomal alpha-mannosidase OS=Mus musculus OX=10090 GN=Man2b1 P	23	20	34	20	1013	114,6	8,13	protein metabolism;other metabolic processes;other biological processes	non-structural extracellular;other membranes;nucleus;other cytoplasmic organelle;other cell compo	other molecular function	Pf01074, Pf07748, Pf09261, Pf17677
Q9QXE0	2-hydroxyacyl-CoA lyase 1 OS=Mus musculus OX=10090 GN=Hacl1 PE=1 SV	9	5	5	5	581	63,6	6,27	cell organization and biogenesis;other metabolic processes;transport	nucleus;other cytoplasmic organelle;other cell component	other molecular function	Pf00205, Pf02775, Pf02776
Q9D937	Uncharacterized protein C11orf98 homolog OS=Mus musculus OX=10090 PE	43	6	24	6	123	14,1	11,47				Pf17719
Q8CJ53	Cdc42-interacting protein 4 OS=Mus musculus OX=10090 GN=Trip10 PE=1 S	15	7	8	7	603	68,4	5,9	cell organization and biogenesis;stress response;transport;developmental proces	plasma membrane;cytoskeleton;ER/Golgi;nucleus;other cytoplasmic organelle;other cell compone	other molecular function	Pf00018, Pf00611
B2KGA7	Protein archease OS=Mus musculus OX=10090 GN=Zbtb8os PE=1 SV=1	32	6	16	4	167	19,6	4,56	RNA metabolism OR transcription;other metabolic processes		other molecular function	Pf01951
Q9D2R0	Acetoacetyl-CoA synthetase OS=Mus musculus OX=10090 GN=Aacs PE=1 S	22	15	25	15	672	75,2	6,71	other metabolic processes;stress response;developmental processes;other biolog	cytosol	other molecular function	Pf00501, Pf16177
G3X8U3	Queuosine salvage protein OS=Mus musculus OX=10090 GN=2210016F16R	21	8	11	8	338	38,6	5,64	RNA metabolism OR transcription;other metabolic processes			Pf10343
P26041	Moesin OS=Mus musculus OX=10090 GN=Msn PE=1 SV=3	68	48	369	34	577	67,7	6,6	cell adhesion;cell cycle OR cell proliferation;cell organization and biogenesis;deve	plasma membrane;other membranes;cytosol;cytoskeleton;other cell component	signal transduction activity or receptor binding;cytoskeletal activity;nucleic acid	Pf00373, Pf00769, Pf09379, Pf09380, Pf2
G3UX26	Outer mitochondrial membrane protein porin 2 OS=Mus musculus OX=1009	20	4	4	4	283	30,4	7,58	transport	other membranes;mitochondrion	transporter activity	Pf01459
Q8CE46	Pseudouridylate synthase 7 homolog-like protein OS=Mus musculus OX=100	29	17	29	17	702	79,1	7,81	RNA metabolism OR transcription;other metabolic processes	nucleus	nucleic acid binding activity;other molecular function	Pf01142
P46414	Cyclin-dependent kinase inhibitor 1B OS=Mus musculus OX=10090 GN=Cdkn	18	3	4	3	197	22,2	7,02	cell cycle OR cell proliferation;stress response;transport;developmental processes	cytosol;nucleus;other cytoplasmic organelle;other cell component	enzyme regulator activity;other molecular function	Pf02234
A6X8Z5	Rho GTPase-activating protein 31 OS=Mus musculus OX=10090 GN=Arhgap3	3	3	4	3	1425	155,2	5,77	signal transduction	other cell component	enzyme regulator activity;other molecular function	Pf00620
Q9ERI5	Bifunctional arginine demethylase and lysyl-hydroxylase JMJD6 OS=Mus mu	34	9	24	9	403	46,5	8,9	cell organization and biogenesis;protein metabolism;RNA metabolism OR transcri	plasma membrane;other membranes;cytosol;nucleus;other cell component	nucleic acid binding activity;other molecular function	Pf02373
P62315	Small nuclear ribonucleoprotein Sm D1 OS=Mus musculus OX=10090 GN=Sn	55	5	36	5	119	13,3	11,56	cell organization and biogenesis;RNA metabolism OR transcription;other metaboli	cytosol;nucleus;other cell component	nucleic acid binding activity;other molecular function	Pf01423
G3X8S8	tRNA-splicing endonuclease subunit Sen15 OS=Mus musculus OX=10090 GN	23	2	2	2	168	18,5	4,78	RNA metabolism OR transcription;other metabolic processes		nucleic acid binding activity;other molecular function	Pf09631
P62774	Myotrophin OS=Mus musculus OX=10090 GN=Mtpn PE=1 SV=2	35	4	43	4	118	12,9	5,52	cell organization and biogenesis;other metabolic processes;developmental proce	cytosol;cytoskeleton;nucleus;other cell component	nucleic acid binding activity	Pf12796
P37040	NADPH--cytochrome P450 reductase OS=Mus musculus OX=10090 GN=Por	31	21	32	21	678	77	5,53	protein metabolism;other metabolic processes;other biological processes	other membranes;cytosol;mitochondrion;ER/Golgi;other cell component	other molecular function	Pf00175, Pf00258, Pf00667
O88878	AN1-type zinc finger protein 5 OS=Mus musculus OX=10090 GN=Zfand5 PE=	69	9	29	8	213	23	8,51	developmental processes;signal transduction;other biological processes	other cell component	nucleic acid binding activity;other molecular function	Pf01428, Pf01754
Q9QUM9	Proteasome subunit alpha type-6 OS=Mus musculus OX=10090 GN=Psma6	55	13	137	13	246	27,4	6,76	protein metabolism;other metabolic processes;developmental processes;other bi	cytosol;cytoskeleton;nucleus;other cell component	nucleic acid binding activity;other molecular function	Pf00227, Pf10584
Q9CZ96	Zinc finger CCHC-type and RNA-binding motif-containing protein 1 OS=Mus m	20	4	7	4	217	24,6	8,54	RNA metabolism OR transcription;other metabolic processes	nucleus	nucleic acid binding activity;other molecular function	Pf00076, Pf00098
Q9CX53	Gem-associated protein 6 OS=Mus musculus OX=10090 GN=Gemin6 PE=1 S	27	4	7	4	166	18,7	5,25	cell organization and biogenesis;RNA metabolism OR transcription;other metaboli	cytosol;nucleus;other cell component		Pf06372, Pf20417
P40142	Transketolase OS=Mus musculus OX=10090 GN=Tkt PE=1 SV=1	69	34	755	34	623	67,6	7,5	other metabolic processes;other biological processes	other membranes;ER/Golgi;nucleus;other cytoplasmic organelle;other cell component	other molecular function	Pf00456, Pf02779, Pf02780
[illegible]												

A6P3E4	Stress-activated protein kinase JNK OS=Mus musculus OX=10090 GN=Mapk	20	7	9	3	384	44	7,49	protein metabolism;other metabolic processes;signal transduction;other biologica
Q3UDK1	TRAF-type zinc finger domain-containing protein 1 OS=Mus musculus OX=10	12	5	5	5	580	64,2	5,48	other biological processes
Q8BYB9	Protein O-glucosyltransferase 1 OS=Mus musculus OX=10090 GN=Poglut1 P	28	12	23	12	392	46,4	8,85	protein metabolism;other metabolic processes;developmental processes;other b
A0A3B2WBH9	Tight junction protein ZO-2 OS=Mus musculus OX=10090 GN=Tjp2 PE=1 SV=	15	16	19	16	1165	131	6,79	
Q8BHG2	CXXC motif containing zinc binding protein OS=Mus musculus OX=10090 GN=	60	9	19	9	160	18	5,12	
P20108	Thioredoxin-dependent peroxide reductase, mitochondrial OS=Mus musculu	43	8	74	8	257	28,1	7,58	cell organization and biogenesis;protein metabolism;other metabolic processes;s
Q3TCJ1	BRISC complex subunit Abraxas 2 OS=Mus musculus OX=10090 GN=Abraxas	41	14	40	14	415	46,9	6,18	cell cycle OR cell proliferation;cell organization and biogenesis;protein metabolism
Q8CG79	Apoptosis-stimulating of p53 protein 2 OS=Mus musculus OX=10090 GN=Tp	12	9	9	9	1128	125,2	6,34	cell cycle OR cell proliferation;developmental processes;signal transduction;other
Q99K85	Phosphoserine aminotransferase OS=Mus musculus OX=10090 GN=Psat1 PE	68	25	361	24	370	40,4	8,03	other metabolic processes
O70139	cAMP-dependent protein kinase inhibitor gamma OS=Mus musculus OX=10	92	4	14	4	76	7,9	4,21	signal transduction;other biological processes
O08992	Syntenin-1 OS=Mus musculus OX=10090 GN=Sdcbp PE=1 SV=1	10	2	2	2	299	32,4	7,15	cell-cell signaling;cell organization and biogenesis;developmental processes;signa
Q8CFI2	Ubiquitin-conjugating enzyme E2 R1 OS=Mus musculus OX=10090 GN=Cdc3	14	3	4	3	235	26,6	4,56	cell cycle OR cell proliferation;protein metabolism;other metabolic processes;othe
A0A0R4J101	Anion exchange protein OS=Mus musculus OX=10090 GN=Slc4a2 PE=1 SV=1	3	2	3	2	1237	136,7	6,14	transport;developmental processes;other biological processes
B9EJR8	Dynein axonemal assembly factor 5 OS=Mus musculus OX=10090 GN=Dnaa	5	4	4	4	853	93,8	6,15	cell organization and biogenesis;other biological processes
P18242	Cathepsin D OS=Mus musculus OX=10090 GN=Ctsd PE=1 SV=1	48	14	114	14	410	44,9	7,15	cell organization and biogenesis;protein metabolism;other metabolic processes;o
Q78JW9	Ubiquitin domain-containing protein UBFD1 OS=Mus musculus OX=10090 G	32	10	31	10	368	40,1	8,85	
Q7TMY7	Importin-8 OS=Mus musculus OX=10090 GN=Ipo8 PE=1 SV=3	5	4	4	4	1010	117	5,16	transport
Q9DD24	Transcription elongation factor A protein-like 9 OS=Mus musculus OX=1009	28	4	4	4	104	12,7	4,84	
A0A494B9H1	Nucleotide exchange factor SIL1 OS=Mus musculus OX=10090 GN=Sil1 PE=1	9	3	6	3	417	46,8	6	
A2CG76	Histone-lysine N-methyltransferase EHMT2 OS=Mus musculus OX=10090 G	6	6	8	6	1229	134,6	5,96	protein metabolism;other metabolic processes
A0A1L1SV73	Ubiquitin carboxyl-terminal hydrolase 47 OS=Mus musculus OX=10090 GN=	5	5	5	5	1376	157,1	5,06	protein metabolism;other metabolic processes
P06801	NADP-dependent malic enzyme OS=Mus musculus OX=10090 GN=Me1 PE=	47	20	52	20	572	63,9	7,44	other metabolic processes;other biological processes
Q8CAK1	Putative transferase CAF17 homolog, mitochondrial OS=Mus musculus OX=	25	7	8	7	358	38,4	9,01	cell organization and biogenesis;other metabolic processes
Q9CPV4	Glyoxalase domain-containing protein 4 OS=Mus musculus OX=10090 GN=G	70	21	82	21	298	33,3	5,47	
Q99PM3	Transcription initiation factor IIA subunit 1 OS=Mus musculus OX=10090 GN=	14	5	16	5	378	41,6	4,55	RNA metabolism OR transcription;other metabolic processes
Q91V77	Protein S100 OS=Mus musculus OX=10090 GN=S100a1 PE=1 SV=1	21	2	4	2	94	10,5	4,59	other biological processes
D3Z7B5	Protein CIP2A OS=Mus musculus OX=10090 GN=Cip2a PE=1 SV=1	2	2	2	2	907	102,1	6,39	
Q91W39	Nuclear receptor coactivator 5 OS=Mus musculus OX=10090 GN=Ncoa5 PE=	4	2	2	2	579	65,3	9,82	other biological processes
Q60709	Amyloid-like protein 2 OS=Mus musculus OX=10090 GN=Aplp2 PE=1 SV=1	9	7	8	6	751	85,2	4,73	other biological processes

[illegible]

Q8K2H6	Anaphase-promoting complex subunit 10 OS=Mus musculus OX=10090 GN=	18	3	4	3	185	21,3	9,06	cell cycle OR cell proliferation;protein metabolism;other metabolic processes;othe	nucleus;other cell component
Q8BVG4	Dipeptidyl peptidase 9 OS=Mus musculus OX=10090 GN=Dpp9 PE=1 SV=2	32	21	46	20	862	97,9	6,65	protein metabolism	cytosol;cytoskeleton;other cell component
Q9D115	Zinc finger protein 706 OS=Mus musculus OX=10090 GN=Znf706 PE=1 SV=1	57	5	23	5	76	8,5	10,01	other biological processes	nucleus;other cell component
Q5SF07	Insulin-like growth factor 2 mRNA-binding protein 2 OS=Mus musculus OX=1	6	3	4	3	592	65,5	8,03	transport;developmental processes;other biological processes	cytosol;cytoskeleton;nucleus;other cell component
Q99KR3	Endoribonuclease LACTB2 OS=Mus musculus OX=10090 GN=Lactb2 PE=1 SV	50	13	45	13	288	32,7	6,33	RNA metabolism OR transcription;other metabolic processes	mitochondrion
Q80UY1	Carnosine N-methyltransferase OS=Mus musculus OX=10090 GN=Carnmt1 F	36	14	38	14	400	46,3	6,16	other metabolic processes	cytosol;nucleus
Q9D2E2	Target of EGR1 protein 1 OS=Mus musculus OX=10090 GN=Toe1 PE=1 SV=1	27	13	29	13	511	56,8	6,64	RNA metabolism OR transcription;other metabolic processes	nucleus;other cell component
P35979	60S ribosomal protein L12 OS=Mus musculus OX=10090 GN=Rpl12 PE=1 SV=	68	10	100	10	165	17,8	9,42	protein metabolism;other metabolic processes	cytosol;translational apparatus;nucleus;other cell component
P26443	Glutamate dehydrogenase 1, mitochondrial OS=Mus musculus OX=10090 G	52	28	284	23	558	61,3	8	other metabolic processes;developmental processes;other biological processes	mitochondrion;ER/Golgi;other cell component
Q99PT1	Rho GDP-dissociation inhibitor 1 OS=Mus musculus OX=10090 GN=Arhgdia P	64	12	274	12	204	23,4	5,2	signal transduction;other biological processes	non-structural extracellular;plasma membrane;other membranes;cyto
Q9D273	Corrinoid adenosyltransferase OS=Mus musculus OX=10090 GN=Mmab PE=	30	7	10	7	237	26,3	9,2	other metabolic processes	
O88668	Protein CREG1 OS=Mus musculus OX=10090 GN=Creg1 PE=1 SV=1	35	8	16	8	220	24,4	6,42	other biological processes	non-structural extracellular;other cell component
A0A0R4J023	Methylglutaconyl-CoA hydratase, mitochondrial OS=Mus musculus OX=100	36	11	24	11	314	33,3	9,42		
Q8CC21	Tetratricopeptide repeat protein 19, mitochondrial OS=Mus musculus OX=10	7	2	3	2	365	41,2	6,21	cell organization and biogenesis	other membranes;mitochondrion
Q60778	NF-kappa-B inhibitor beta OS=Mus musculus OX=10090 GN=Nfkbib PE=1 SV	12	4	4	4	359	37,9	4,74	signal transduction;other biological processes	cytosol;nucleus;other cell component
Q9CQI7	U2 small nuclear ribonucleoprotein B'' OS=Mus musculus OX=10090 GN=Snr	35	7	87	5	225	25,3	9,72	RNA metabolism OR transcription;other metabolic processes	nucleus;other cell component
Q99LE1	RILP-like protein 2 OS=Mus musculus OX=10090 GN=Rilpl2 PE=1 SV=1	39	6	7	6	197	22,4	5,12	cell organization and biogenesis;transport;other biological processes	other membranes;cytosol;cytoskeleton;other cell component
Q9JHW2	Omega-amidase NIT2 OS=Mus musculus OX=10090 GN=Nit2 PE=1 SV=1	81	18	74	9	276	30,5	6,9	other metabolic processes	cytosol;cytoskeleton
O35601	FYN-binding protein 1 OS=Mus musculus OX=10090 GN=Fyb1 PE=1 SV=2	33	20	40	20	819	90	6,98	signal transduction;other biological processes	plasma membrane;other membranes;cytosol;cytoskeleton;nucleus;ot
P97429	Annexin A4 OS=Mus musculus OX=10090 GN=Anxa4 PE=1 SV=4	56	19	68	19	319	35,9	5,57	developmental processes;signal transduction;other biological processes	plasma membrane;other membranes;nucleus;other cell component
Q9JI13	Something about silencing protein 10 OS=Mus musculus OX=10090 GN=Utp	11	4	5	4	469	53,4	5,54	cell organization and biogenesis;RNA metabolism OR transcription;other metabol	nucleus;other cell component
A0A140T8Q6	Allergin-1 OS=Mus musculus OX=10090 GN=Milr1 PE=1 SV=1	15	3	3	3	245	27,2	7,25	transport;other biological processes	other membranes
O70172	Phosphatidylinositol 5-phosphate 4-kinase type-2 alpha OS=Mus musculus C	28	10	17	5	405	46,1	6,99	cell organization and biogenesis;other metabolic processes;transport;developme	plasma membrane;other membranes;cytosol;nucleus;other cytoplasm
Q8BP78	Protein FRA10AC1 homolog OS=Mus musculus OX=10090 GN=Fra10ac1 PE=	33	10	13	10	315	37,2	7,28	other metabolic processes	nucleus
Q922I7	Polypyrimidine tract-binding protein 1 OS=Mus musculus OX=10090 GN=Ptb	17	8	22	8	555	59,3	9,16	RNA metabolism OR transcription;other metabolic processes	nucleus
O08759	Ubiquitin-protein ligase E3A OS=Mus musculus OX=10090 GN=Ube3a PE=1 S	34	25	63	25	870	99,8	5,08	protein metabolism;other metabolic processes;stress response;transport;develop	cytosol;nucleus;other cell component
Q8C167	Prolyl endopeptidase-like OS=Mus musculus OX=10090 GN=Prepl PE=1 SV=	15	9	16	9	725	83,1	6,65	protein metabolism;transport;other biological processes	cytosol;cytoskeleton;ER/Golgi;nucleus
P14152	Malate dehydrogenase, cytoplasmic OS=Mus musculus OX=10090 GN=Mdh	44	16	182	16	334	36,5	6,58	other metabolic processes	cytosol;cytoskeleton
A0A0R4J091	DNA polymerase epsilon subunit 3 OS=Mus musculus OX=10090 GN=Pole3 F	59	10	22	10	145	16,7	4,74	protein metabolism;other metabolic processes	nucleus
P61971	Nuclear transport factor 2 OS=Mus musculus OX=10090 GN=Nutf2 PE=1 SV=	51	4	51	4	127	14,5	5,38	transport;other biological processes	other membranes;cytosol;nucleus
A2A7S7	Tyrosine--tRNA ligase OS=Mus musculus OX=10090 GN=Yars PE=1 SV=1	67	38	174	2	564	63	8,44	protein metabolism;RNA metabolism OR transcription;other metabolic processes	cytosol;nucleus
Q9ER00	Syntaxin-12 OS=Mus musculus OX=10090 GN=Stx12 PE=1 SV=1	15	4	6	4	274	31,2	5,44	cell organization and biogenesis;other metabolic processes;transport;other biolog	other membranes;cytosol;ER/Golgi;nucleus;other cytoplasmic organel
Q9QWZ1	Cell cycle checkpoint protein RAD1 OS=Mus musculus OX=10090 GN=Rad1 P	27	6	11	6	280	31,6	4,84	cell cycle OR cell proliferation;DNA metabolism;other metabolic processes;stress	nucleus;other cell component
Q80Y56	Rabenosyn-5 OS=Mus musculus OX=10090 GN=Rbsn PE=1 SV=1	4	3	3	3	783	88,4	5,3	transport;other biological processes	plasma membrane;other membranes;cytosol;other cytoplasmic organ
Q8BTN3	Transport and Golgi organization 2 homolog OS=Mus musculus OX=10090 G	24	6	8	6	276	30,9	5,1		ER/Golgi
Q8BVU5	ADP-ribose pyrophosphatase, mitochondrial OS=Mus musculus OX=10090 G	47	15	35	15	350	38,6	6,76	other metabolic processes	other membranes;nucleus;other cell component
Q3ULW8	Protein mono-ADP-ribosyltransferase PARP3 OS=Mus musculus OX=10090 G	4	2	2	2	533	59,9	7,12	cell organization and biogenesis;protein metabolism;DNA metabolism;other meta	non-structural extracellular;nucleus;other cell component
P50396	Rab GDP dissociation inhibitor alpha OS=Mus musculus OX=10090 GN=Gdi1 F	44	17	99	10	447	50,5	5,08	cell organization and biogenesis;transport;developmental processes;signal transd	ER/Golgi;other cell component
Q8BS90	Protein aurora borealis OS=Mus musculus OX=10090 GN=Bora PE=1 SV=1	7	2	4	2	525	57	5,19	cell cycle OR cell proliferation;other biological processes	cytoskeleton;nucleus;other cell component
Q9QUJ7	Long-chain-fatty-acid--CoA ligase 4 OS=Mus musculus OX=10090 GN=Acsl4	12	8	11	8	711	79	8,28	cell organization and biogenesis;other metabolic processes;transport;developme	plasma membrane;other membranes;mitochondrion;ER/Golgi;other c
P35564	Calnexin OS=Mus musculus OX=10090 GN=Canx PE=1 SV=1	26	17	32	17	591	67,2	4,64	stress response;transport;signal transduction;other biological processes	plasma membrane;other membranes;ER/Golgi;translational apparatus
Q8BSI6	R3H and coiled-coil domain-containing protein 1 OS=Mus musculus OX=100	11	3	4	3	488	54,1	4,94		
Q9WVA4	Transgelin-2 OS=Mus musculus OX=10090 GN=Tagln2 PE=1 SV=4	80	17	179	17	199	22,4	8,24	other biological processes	
Q9CR80	Protein FAM32A OS=Mus musculus OX=10090 GN=Fam32a PE=2 SV=1	29	4	4	4	112	13,2	9,94	cell cycle OR cell proliferation;other biological processes	nucleus
E9QAS5	DNA helicase OS=Mus musculus OX=10090 GN=Chd4 PE=1 SV=1	13	20	34	17	1922	218,5	5,86	cell organization and biogenesis;other biological processes	
Q8BWT1	3-ketoacyl-CoA thiolase, mitochondrial OS=Mus musculus OX=10090 GN=Ac	63	17	64	15	397	41,8	8,09	cell organization and biogenesis;other metabolic processes;stress response;trans	mitochondrion
D3Z4U0	Zinc finger Ran-binding domain-containing protein 2 OS=Mus musculus OX=1	48	19	89	19	320	36,3	9,67		nucleus
D3Z2H9	Tropomyosin 3, related sequence 7 OS=Mus musculus OX=10090 GN=Tpm3	71	26	313	8	248	29	4,81	cell organization and biogenesis;other biological processes	cytoskeleton
Q9JKB3	Y-box-binding protein 3 OS=Mus musculus OX=10090 GN=Ybx3 PE=1 SV=2	67	13	179	10	361	38,8	9,69	stress response;developmental processes;other biological processes	cytosol;nucleus;other cell component
P50580	Proliferation-associated protein 2G4 OS=Mus musculus OX=10090 GN=Pa2g	73	24	306	23	394	43,7	6,86	RNA metabolism OR transcription;other metabolic processes;other biological proc	nucleus;other cell component
Q8BJ90	Zinc finger protein 771 OS=Mus musculus OX=10090 GN=Znf771 PE=2 SV=1	23	5	5	5	317	35,7	8,82	other biological processes	nucleus
O35900	U6 snRNA-associated Sm-like protein LSm2 OS=Mus musculus OX=10090 GN	67	5	29	5	95	10,8	6,52	cell organization and biogenesis;RNA metabolism OR transcription;other metabol	nucleus;other cell component
P61164	Alpha-centractin OS=Mus musculus OX=10090 GN=Actr1a PE=1 SV=1	55	16	62	7	376	42,6	6,64	other biological processes	cytoskeleton;ER/Golgi;other cell component
Q9CQU0	Thioredoxin domain-containing protein 12 OS=Mus musculus OX=10090 GN=	68	9	21	9	170	19	5,26	other biological processes	ER/Golgi
O35639	Annexin A3 OS=Mus musculus OX=10090 GN=Anxa3 PE=1 SV=4	67	24	113	24	323	36,4	5,76	stress response;transport;developmental processes;other biological processes	plasma membrane;other membranes;other cell component
Q9D8X2	Coiled-coil domain-containing protein 124 OS=Mus musculus OX=10090 GN=	44	12	18	12	217	25,3	9,64	cell cycle OR cell proliferation;other biological processes	plasma membrane;other membranes;cytosol;cytoskeleton;other cell c
A2AMW0	F-actin-capping protein subunit beta OS=Mus musculus OX=10090 GN=Capz	72	19	146	3	260	29,3	6,92	cell organization and biogenesis	cytoskeleton
Q9QZ23	NFU1 iron-sulfur cluster scaffold homolog, mitochondrial OS=Mus musculus	36	6	12	6	255	28,5	5,03	cell organization and biogenesis;protein metabolism;other metabolic processes	cytosol;nucleus
P63005	Platelet-activating factor acetylhydrolase IB subunit beta OS=Mus musculus	63	24	106	23	410	46,6	7,37	cell-cell signaling;cell cycle OR cell proliferation;cell organization and biogenesis;o	other membranes;cytosol;cytoskeleton;nucleus;other cell component
Q6P6J0	tRNA-specific adenosine deaminase 2 OS=Mus musculus OX=10090 GN=Ada	41	6	13	6	191	21,3	5,88	RNA metabolism OR transcription;other metabolic processes	
P70445	Eukaryotic translation initiation factor 4E-binding protein 2 OS=Mus musculu	73	5	25	5	120	12,9	6,52	signal transduction;other biological processes	other cell component
Q8VDF2	E3 ubiquitin-protein ligase UHRF1 OS=Mus musculus OX=10090 GN=Uhrf1 P	24	17	23	16	782	88,2	8,31	cell cycle OR cell proliferation;protein metabolism;DNA metabolism;other metab	plasma membrane;other membranes;nucleus;other cell component
Q9JJU8	SH3 domain-binding glutamic acid-rich-like protein OS=Mus musculus OX=1	87	8	61	8	114	12,8	4,92		
Q8BYW9	EGF domain-specific O-linked N-acetylglucosamine transferase OS=Mus mu	22	11	14	11	527	61,4	6,81	protein metabolism;other metabolic processes	ER/Golgi
Q99MN9	Propionyl-CoA carboxylase beta chain, mitochondrial OS=Mus musculus OX=	36	17	32	17	541	58,4	7,66		mitochondrion
B2RXQ2	Ppfia1 protein OS=Mus musculus OX=10090 GN=Ppfia1 PE=1 SV=1	2	2	2	2	1266	142,6	6,25	cell organization and biogenesis;other biological processes	cytosol;other cell component
O08804	NK13 OS=Mus musculus OX=10090 GN=Serpinb6b PE=1 SV=2	48	15	69	15	377	42,5	5,26	developmental processes;other biological processes	non-structural extracellular;nucleus;other cell component
P56382	ATP synthase subunit epsilon, mitochondrial OS=Mus musculus OX=10090 G	42	3	7	3	52	5,8	10,01	other metabolic processes;transport	other membranes;mitochondrion
Q9DCJ5	NADH dehydrogenase [ubiquinone] 1 alpha subcomplex subunit 8 OS=Mus n	26	4	4	4	172	20	8,46	cell organization and biogenesis;other metabolic processes	other membranes;mitochondrion
Q8QZR5	Alanine aminotransferase 1 OS=Mus musculus OX=10090 GN=Gpt PE=1 SV=	50	20	137	19	496	55,1	6,64	other metabolic processes;stress response;other biological processes	non-structural extracellular;other cell component
Q3U1J4	DNA damage-binding protein 1 OS=Mus musculus OX=10090 GN=Ddb1 PE=	58	56	321	56	1140	126,8	5,26	cell-cell signaling;protein metabolism;DNA metabolism;other metabolic processe	nucleus;other cell component
Q3UIX4	Serine and arginine-rich-splicing factor 11 OS=Mus musculus OX=10090 GN=	28	8	13	8	476	53,1	10,58	RNA metabolism OR transcription;other metabolic processes	nucleus
P08905	Lysozyme C-2 OS=Mus musculus OX=10090 GN=Lyz2 PE=1 SV=2	24	4	8	4	148	16,7	8,81	other metabolic processes;stress response;other biological processes	non-structural extracellular;ER/Golgi;other cell component
Q9D6N1	Carbonic anhydrase 13 OS=Mus musculus OX=10090 GN=Ca13 PE=1 SV=1	19	4	4	4	262	29,5	6,65	other metabolic processes	cytosol;other cell component
P17918	Proliferating cell nuclear antigen OS=Mus musculus OX=10090 GN=Pcna PE=	78	19	448	19	261	28,8	4,77	cell cycle OR cell proliferation;cell organization and biogenesis;DNA metabolism;c	cytoskeleton;nucleus;other cell component
Q69ZC8	GPALPP motifs-containing protein 1 OS=Mus musculus OX=10090 GN=Gpalp	44	13	27	13	346	38,9	5,41		
Q8VDP3	[F-actin]-monooxygenase MICAL1 OS=Mus musculus OX=10090 GN=Mical1	8	7	9	7	1048	116,7	6,05	cell organization and biogenesis;other metabolic processes;other biological proce	non-structural extracellular;cytoskeleton;other cell component
[illegible]	[illegible]	[illegible]	[illegible]	[illegible]	[illegible]	[illegible]	[illegible]	[illegible]	[illegible]	[illegible]

Q91YP3	Deoxyribose-phosphate aldolase OS=Mus musculus OX=10090 GN=Dera PE	46	12	28	12	318	35	8,72	other metabolic processes	nucleus;other cell component
Q9CXZ1	NADH dehydrogenase [ubiquinone] iron-sulfur protein 4, mitochondrial OS=M	37	6	14	6	175	19,8	9,99	cell organization and biogenesis;other metabolic processes;stress response;trans	other membranes;mitochondrion
P50247	Adenosylhomocysteinase OS=Mus musculus OX=10090 GN=Ahcy PE=1 SV=	73	30	407	28	432	47,7	6,54	other metabolic processes;stress response;other biological processes	cytosol;nucleus;other cell component
Q9JM93	ADP-ribosylation factor-like protein 6-interacting protein 4 OS=Mus musculus	22	4	8	4	229	25,5	11,19	RNA metabolism OR transcription;other metabolic processes	nucleus
Q14B01	Ring finger protein 113A2 OS=Mus musculus OX=10090 GN=Rnf113a2 PE=1	51	15	48	10	337	38	6,46	protein metabolism;RNA metabolism OR transcription;other metabolic processes;	nucleus
Q05CL8	La-related protein 7 OS=Mus musculus OX=10090 GN=Larp7 PE=1 SV=2	37	20	37	20	570	64,8	9,54	cell cycle OR cell proliferation;RNA metabolism OR transcription;other metabolic p	cytosol;nucleus;other cell component
Q921L5	Conserved oligomeric Golgi complex subunit 2 OS=Mus musculus OX=10090	6	3	4	3	731	82	6,21	cell organization and biogenesis;transport	other membranes;cytosol;ER/Golgi
Q91WK5	Glycine cleavage system H protein, mitochondrial OS=Mus musculus OX=10	18	2	56	2	170	18,6	4,75	protein metabolism;other metabolic processes	other cell component
Q8BUK6	Protein Hook homolog 3 OS=Mus musculus OX=10090 GN=Hook3 PE=1 SV=	8	5	7	5	718	83,2	5,19	cell cycle OR cell proliferation;cell organization and biogenesis;transport;developr	other membranes;cytosol;cytoskeleton;ER
Q4FCQ7	Arginyl-tRNA--protein transferase 1 OS=Mus musculus OX=10090 GN=Ate1	35	14	23	3	509	58,3	8,16	protein metabolism;other metabolic processes	
P05063	Fructose-bisphosphate aldolase C OS=Mus musculus OX=10090 GN=Aldoc P	73	27	365	19	363	39,4	7,12	other metabolic processes;other biological processes	cytosol;other cell component
O70310	Glycylpeptide N-tetradecanoyltransferase 1 OS=Mus musculus OX=10090 G	39	17	56	14	496	56,9	8	protein metabolism;other metabolic processes;developmental processes	plasma membrane;other membranes;cyto
Q8BGD9	Eukaryotic translation initiation factor 4B OS=Mus musculus OX=10090 GN=E	52	34	257	34	611	68,8	5,67	cell organization and biogenesis;protein metabolism;other metabolic processes	cytosol;other cell component
Q9DBL7	Bifunctional coenzyme A synthase OS=Mus musculus OX=10090 GN=Coasy	3	2	2	2	563	62	7,11	other metabolic processes	mitochondrion
Q91VJ1	Interferon-inducible protein AIM2 OS=Mus musculus OX=10090 GN=Aim2 P	17	6	8	6	354	40,1	8,62	cell organization and biogenesis;stress response;signal transduction;other biologi	cytosol;mitochondrion;nucleus;other cell co
Q922Q9	Chitinase domain-containing protein 1 OS=Mus musculus OX=10090 GN=Chi	18	7	12	7	393	44,9	7,97	other metabolic processes;stress response;other biological processes	non-structural extracellular;ER/Golgi;other c
O70274	Protein tyrosine phosphatase type IVA 2 OS=Mus musculus OX=10090 GN=F	53	8	16	5	167	19,1	8,37	protein metabolism;other metabolic processes	plasma membrane;other membranes;nucle
Q9CRC9	Glucosamine-6-phosphate isomerase 2 OS=Mus musculus OX=10090 GN=G	43	9	26	6	276	31,1	6,9	other metabolic processes	other cell component
P01901	H-2 class I histocompatibility antigen, K-B alpha chain OS=Mus musculus OX	22	6	11	4	369	41,3	6,39	stress response;developmental processes;other biological processes	non-structural extracellular;plasma membra
Q99JR6	Nicotinamide/nicotinic acid mononucleotide adenylyltransferase 3 OS=Mus	15	3	4	3	245	27,7	8,56	other metabolic processes;stress response;other biological processes	mitochondrion;nucleus;other cell componer
Q80TZ9	Arginine-glutamic acid dipeptide repeats protein OS=Mus musculus OX=100	4	7	9	6	1558	171,6	7,69	cell cycle OR cell proliferation;cell organization and biogenesis;developmental pro	nucleus
Q9CZ91	Serum response factor-binding protein 1 OS=Mus musculus OX=10090 GN=S	39	14	25	14	441	48,7	9,54	RNA metabolism OR transcription;other metabolic processes	nucleus;other cell component
Q91YP2	Neurolysin, mitochondrial OS=Mus musculus OX=10090 GN=Nln PE=1 SV=1	45	30	114	23	704	80,4	6,44	protein metabolism;other metabolic processes;other biological processes	plasma membrane;other membranes;cyto
E9Q634	Unconventional myosin-Ie OS=Mus musculus OX=10090 GN=Myo1e PE=1 S	28	27	45	24	1107	126,7	9,07	cell organization and biogenesis;other metabolic processes;transport;developme	plasma membrane;other membranes;cyto
Q9CR29	Coiled-coil domain-containing protein 43 OS=Mus musculus OX=10090 GN=C	49	11	27	11	222	25	4,97		cytosol
F7BJB9	MORC family CW-type zinc finger protein 3 OS=Mus musculus OX=10090 GN	11	7	9	7	942	106,5	5,68	protein metabolism;other metabolic processes;developmental processes;other b	nucleus
Q9QY93	dCTP pyrophosphatase 1 OS=Mus musculus OX=10090 GN=Dctpp1 PE=1 SV	55	9	43	9	170	18,8	5,03	DNA metabolism;other metabolic processes;stress response	cytosol
Q00612	Glucose-6-phosphate 1-dehydrogenase X OS=Mus musculus OX=10090 GN=	63	33	261	12	515	59,2	6,49	other metabolic processes;stress response;developmental processes;other biolog	plasma membrane;other membranes;cyto
O35161	Cadherin EGF LAG seven-pass G-type receptor 1 OS=Mus musculus OX=100	3	4	7	4	3034	330,3	5,88	cell adhesion;cell-cell signaling;stress response;developmental processes;signal t	plasma membrane;other membranes;nucle

Q9D104	Signal recognition particle 19 kDa protein OS=Mus musculus OX=10090 GN=	38	5	25	5	144	16,1	9,88
P24668	Cation-dependent mannose-6-phosphate receptor OS=Mus musculus OX=1	31	8	9	8	278	31,2	5,39
D3YYT1	Glyoxylate reductase 1 homolog OS=Mus musculus OX=10090 GN=Glyr1 PE	11	5	7	5	552	60,4	9,13
Q9R0B7	Zinc finger protein 346 OS=Mus musculus OX=10090 GN=Znf346 PE=1 SV=1	31	8	10	8	294	32,7	8,88
Q91ZF0	DnaJ homolog subfamily C member 24 OS=Mus musculus OX=10090 GN=Dr	29	4	6	4	148	16,9	4,92
G5E866	Splicing factor 3B subunit 1 OS=Mus musculus OX=10090 GN=Sf3b1 PE=1 SV	34	38	78	38	1304	145,7	7,09
P58058	NAD kinase OS=Mus musculus OX=10090 GN=Nadk PE=1 SV=2	32	11	40	11	439	48,6	6,52
Q99J77	Sialic acid synthase OS=Mus musculus OX=10090 GN=Nans PE=1 SV=1	52	15	60	15	359	40	7,06
Q9JI95	CPN10-like protein OS=Mus musculus OX=10090 GN=Hspe1-rs1 PE=3 SV=1	68	7	151	7	102	11	8,92
P06800	Receptor-type tyrosine-protein phosphatase C OS=Mus musculus OX=10090	14	14	20	14	1293	144,7	5,94
Q8CAA7	Glucose 1,6-bisphosphate synthase OS=Mus musculus OX=10090 GN=Pgm2	27	15	24	14	621	70,2	6,49
P28076	Proteasome subunit beta type-9 OS=Mus musculus OX=10090 GN=Psmb9 P	14	3	5	3	219	23,4	5,22
F8WJ64	Acyl carrier protein (Fragment) OS=Mus musculus OX=10090 GN=Ndufab1 P	18	2	11	2	128	14,4	4,77
Q62318	Transcription intermediary factor 1-beta OS=Mus musculus OX=10090 GN=T	50	26	69	26	834	88,8	5,77
O08997	Copper transport protein ATOX1 OS=Mus musculus OX=10090 GN=Atox1 PE	88	6	105	6	68	7,3	6,51
Q9D8L5	Coiled-coil domain-containing protein 91 OS=Mus musculus OX=10090 GN=C	17	9	12	9	442	50	5,07
P83741	Serine/threonine-protein kinase WNK1 OS=Mus musculus OX=10090 GN=W	12	25	32	25	2377	250,8	6,43
P42128	Forkhead box protein K1 OS=Mus musculus OX=10090 GN=Foxk1 PE=1 SV=	23	13	17	12	719	74,9	9,17
O08582	GTP-binding protein 1 OS=Mus musculus OX=10090 GN=Gtpbp1 PE=1 SV=2	26	13	18	12	668	72,3	8,29
Q9CQ80	Vacuolar protein-sorting-associated protein 25 OS=Mus musculus OX=10090	20	4	4	4	176	20,7	6,34
Q8BP47	Asparagine--tRNA ligase, cytoplasmic OS=Mus musculus OX=10090 GN=NA	45	24	168	23	559	64,2	5,86
F8WIJ2	DDB1- and CUL4-associated factor 17 OS=Mus musculus OX=10090 GN=Dca	18	3	4	3	156	18	9,39
Q9DCA2	28S ribosomal protein S11, mitochondrial OS=Mus musculus OX=10090 GN=	29	4	4	4	191	20,2	10,77
P62242	40S ribosomal protein S8 OS=Mus musculus OX=10090 GN=Rps8 PE=1 SV=2	51	10	64	10	208	24,2	10,32
Q9D753	Exosome complex component RRP43 OS=Mus musculus OX=10090 GN=Exos	46	10	36	10	276	29,9	5,2
Q9JKB1	Ubiquitin carboxyl-terminal hydrolase isozyme L3 OS=Mus musculus OX=10	57	12	61	12	230	26,1	5,05
Q8BHB4	WD repeat-containing protein 3 OS=Mus musculus OX=10090 GN=Wdr3 PE=	19	13	17	13	942	105,7	6,64
A0A1D5RLR3	RNA-binding protein 34 OS=Mus musculus OX=10090 GN=Rbm34 PE=1 SV=1	17	6	10	6	422	46,7	9,98
Q8VE88	Protein FAM114A2 OS=Mus musculus OX=10090 GN=Fam114a2 PE=1 SV=2	14	5	6	5	497	54	4,93
O55029	Coatomer subunit beta' OS=Mus musculus OX=10090 GN=Copb2 PE=1 SV=2	46	35	95	35	905	102,4	5,3
O70194	Eukaryotic translation initiation factor 3 subunit D OS=Mus musculus OX=10	46	19	94	19	548	63,9	6,05
O35435	Dihydroorotate dehydrogenase (quinone), mitochondrial OS=Mus musculus	10	3	4	3	395	42,7	9,55
Q8VED9	Galectin-related protein OS=Mus musculus OX=10090 GN=Lgalsl PE=1 SV=1	48	7	9	7	172	18,9	5,35
Q61239	Protein farnesyltransferase/geranylgeranyltransferase type-1 subunit alpha	20	8	13	8	377	44	4,93
Q9DBR1	5'-3' exoribonuclease 2 OS=Mus musculus OX=10090 GN=Xrn2 PE=1 SV=1	36	34	146	34	951	108,6	7,59
A0A0G2JGX4	Sodium/potassium-transporting ATPase subunit alpha OS=Mus musculus O	19	15	26	6	1026	112,9	5,41
P70353	Nuclear transcription factor Y subunit gamma OS=Mus musculus OX=10090	8	3	3	3	335	37,2	4,98
Q8VDN2	Sodium/potassium-transporting ATPase subunit alpha-1 OS=Mus musculus	30	26	48	17	1023	112,9	5,45
Q9ERH4	Nucleolar and spindle-associated protein 1 OS=Mus musculus OX=10090 GN	20	8	12	8	427	48,5	9,89
G5E8J3	WD repeat-containing protein 11 OS=Mus musculus OX=10090 GN=Wdr11 P	9	8	11	8	1223	135,9	7,11
Q8CI11	Guanine nucleotide-binding protein-like 3 OS=Mus musculus OX=10090 GN=	28	14	25	13	538	60,7	9,11
Q8R332	Nucleoporin p58/p45 OS=Mus musculus OX=10090 GN=Nup58 PE=1 SV=1	14	8	15	8	587	59,4	9,29
Q91XA2	Golgi membrane protein 1 OS=Mus musculus OX=10090 GN=Golm1 PE=1 SV	14	5	5	5	393	44,3	5,27
O54786	DNA fragmentation factor subunit alpha OS=Mus musculus OX=10090 GN=D	36	9	24	9	331	36,6	4,91
P52480	Pyruvate kinase PKM OS=Mus musculus OX=10090 GN=Pkm PE=1 SV=4	83	42	1256	38	531	57,8	7,47
A0A2I3BPX9	ETS-related transcription factor Elf-1 OS=Mus musculus OX=10090 GN=Elf1	7	2	3	2	575	62,3	5,16
P62627	Dynein light chain roadblock-type 1 OS=Mus musculus OX=10090 GN=Dynlr	70	6	29	6	96	11	7,25
P62259	14-3-3 protein epsilon OS=Mus musculus OX=10090 GN=Ywhae PE=1 SV=1	76	21	692	18	255	29,2	4,74
A6H611	Mitochondrial intermediate peptidase OS=Mus musculus OX=10090 GN=Mi	15	11	12	11	711	80,8	7,15
Q9CQ71	Replication protein A 14 kDa subunit OS=Mus musculus OX=10090 GN=Rpa3	61	5	9	5	121	13,6	4,84
A0A0R4J009	Actin-related protein 6 OS=Mus musculus OX=10090 GN=Actr6 PE=1 SV=1	13	4	7	4	396	45,8	5,03
B7ZC46	Septin OS=Mus musculus OX=10090 GN=Septin8 PE=1 SV=1	47	15	80	10	440	50,9	6,09
Q9D1P0	39S ribosomal protein L13, mitochondrial OS=Mus musculus OX=10090 GN=	58	11	20	11	178	20,7	9,35
Q8R107	PRELI domain-containing protein 1, mitochondrial OS=Mus musculus OX=10	8	2	3	2	217	24,9	9,55
Q9D7W5	Mediator of RNA polymerase II transcription subunit 8 OS=Mus musculus OX	26	6	8	6	268	29,2	6,95
Q8VDJ3	Vigilin OS=Mus musculus OX=10090 GN=Hdlbp PE=1 SV=1	27	30	39	30	1268	141,7	6,87
Q80SY6	Adenosine deaminase-like protein OS=Mus musculus OX=10090 GN=Adal P	21	8	14	8	360	41	6,35
Q9CX66	NOP protein chaperone 1 OS=Mus musculus OX=10090 GN=Nopchap1 PE=1	21	3	3	3	185	20	4,84
Q3UKJ7	WD40 repeat-containing protein SMU1 OS=Mus musculus OX=10090 GN=Sr	33	14	28	14	513	57,5	7,18
Q9ES56	Trafficking protein particle complex subunit 4 OS=Mus musculus OX=10090	39	8	11	8	219	24,4	6,21
E9Q360	Integrator complex subunit 10 OS=Mus musculus OX=10090 GN=Ints10 PE=	5	4	4	4	736	84,8	7,33
C0HKG5	Ribonuclease T2-A OS=Mus musculus OX=10090 GN=Rnaset2a PE=1 SV=1	14	3	4	3	259	29,6	6,37
A0A1Y7VKX7	RNA helicase OS=Mus musculus OX=10090 GN=Ddx24 PE=1 SV=1	10	7	8	7	791	88,6	8,91
Q3UXL4	Centrosomal protein kizuna OS=Mus musculus OX=10090 GN=Kiz PE=1 SV=1	4	2	2	2	695	76,5	5,62
Q8BTU7	RCC1 domain-containing protein 1 OS=Mus musculus OX=10090 GN=Rccd1 P	9	2	2	2	377	40,6	5,26
Q80ZM7	Transcription initiation factor IIA subunit 2 OS=Mus musculus OX=10090 GN=	28	4	13	4	109	12,5	6,62
Q9CZG3	COMM domain-containing protein 8 OS=Mus musculus OX=10090 GN=Comm	51	7	9	7	183	20,8	5,59
Q8BKE6	Cytochrome P450 20A1 OS=Mus musculus OX=10090 GN=Cyp20a1 PE=1 SV	10	3	3	3	462	52,1	6,95
Q8VBT1	Beta-taxilin OS=Mus musculus OX=10090 GN=Txlnb PE=1 SV=2	3	2	2	2	685	77,2	5,01
Q8BGT7	Survival of motor neuron-related-splicing factor 30 OS=Mus musculus OX=1	44	8	36	8	238	26,7	7,24
E9Q405	Unconventional myosin-XVIIIa OS=Mus musculus OX=10090 GN=Myo18a P	7	10	10	10	1700	193,5	5,77
D3Z093	Lipoyltransferase 1, mitochondrial (Fragment) OS=Mus musculus OX=10090	15	4	5	4	230	25,7	9,25
F8WIE5	HECT-type E3 ubiquitin transferase OS=Mus musculus OX=10090 GN=Hectd	9	19	26	19	2610	289	5,36
Q99J09	Methylosome protein 50 OS=Mus musculus OX=10090 GN=Wdr77 PE=1 SV=	47	11	67	11	342	36,9	5,27
Q810V0	U3 small nucleolar ribonucleoprotein protein MPP10 OS=Mus musculus OX=	12	8	8	8	681	78,7	4,87
P58686	Bublin coiled-coil protein OS=Mus musculus OX=10090 GN=Bbln PE=3 SV=1	30	2	6	2	83	9	4,26
A2AI52	Ubiquitin carboxyl-terminal hydrolase 8 OS=Mus musculus OX=10090 GN=U	14	13	14	13	1091	123,8	8,54
Q9D338	39S ribosomal protein L19, mitochondrial OS=Mus musculus OX=10090 GN=	34	8	18	8	292	33,6	9,44
A0A0R4J0J8	Cytospin-A OS=Mus musculus OX=10090 GN=Specc1l PE=1 SV=1	4	5	6	4	1135	126,5	5,94
Q9CPU0	Lactoylglutathione lyase OS=Mus musculus OX=10090 GN=Glo1 PE=1 SV=3	65	10	38	10	184	20,8	5,47
Q78JE5	F-box only protein 22 OS=Mus musculus OX=10090 GN=Fbxo22 PE=1 SV=2	34	11	25	5	402	44,2	7,61
Q8BIP0	Aspartate--tRNA ligase, mitochondrial OS=Mus musculus OX=10090 GN=Da	43	25	55	25	653	74,1	6,98
P23591	GDP-L-fucose synthase OS=Mus musculus OX=10090 GN=Gfus PE=1 SV=3	60	13	45	13	321	35,9	6,74
Q9Z2X1	Heterogeneous nuclear ribonucleoprotein F OS=Mus musculus OX=10090 G	43	14	77	12	415	45,7	5,49
Q9D385	ADP-ribosylation factor-like protein 2-binding protein OS=Mus musculus OX=	20	3	7	3	163	18,7	4,32
A2AN61	CDKN1A-interacting zinc finger protein 1 OS=Mus musculus OX=10090 GN=C	5	4	7	4	821	91	5,53
Q9D287	Pre-mRNA-splicing factor SPF27 OS=Mus musculus OX=10090 GN=Bcas2 PE	49	9	12	9	225	26,1	5,66
Q6ZPU9	KIF-binding protein OS=Mus musculus OX=10090 GN=Kifbp PE=1 SV=2	6	2	2	2	617	71	5,48
Q9EPB5	Serine hydrolase-like protein OS=Mus musculus OX=10090 GN=Serhl PE=1 S	23	6	6	6	311	35,3	8,1
Q9ERL7	Glia maturation factor gamma OS=Mus musculus OX=10090 GN=Gmfg PE=1	36	5	11	4	142	16,7	5,71
Q8VCQ3	Nuclear receptor-binding factor 2 OS=Mus musculus OX=10090 GN=Nrbf2 PE	18	5	9	5	287	32,5	5,76
Q62446	Peptidyl-prolyl cis-trans isomerase FKBP3 OS=Mus musculus OX=10090 GN=	61	13	105	13	224	25,1	9,28
P55264	Adenosine kinase OS=Mus musculus OX=10090 GN=Adk PE=1 SV=2	47	16	92	16	361	40,1	6,21
Q9EQ61	Pescadillo homolog OS=Mus musculus OX=10090 GN=Pes1 PE=1 SV=1	23	12	22	12	584	67,8	6,84
Q8BFV2	PCI domain-containing protein 2 OS=Mus musculus OX=10090 GN=Pcid2 PE=	21	8	11	8	399	46,1	8,53
P40336	Vacuolar protein sorting-associated protein 26A OS=Mus musculus OX=1009	60	15	85	15	327	38,1	6,57
Q9CX00	IST1 homolog OS=Mus musculus OX=10090 GN=Ist1 PE=1 SV=1	12	5	7	5	362	39,4	5,44
O70378	ER membrane protein complex subunit 8 OS=Mus musculus OX=10090 GN=E	30	5	10	5	207	23,3	6,15
A0A0N4SUH4	Pumilio homolog 3 OS=Mus musculus OX=10090 GN=Pum3 PE=1 SV=1	4	3	6	3	648	72,9	9,66
D3Z2D7	Phenazine biosynthesis-like domain-containing protein 2 (Fragment) OS=Mu	13	2	2	2	175	19,9	10,48
Q3ULB0	RNA-binding motif protein 6 OS=Mus musculus OX=10090 GN=Rbm6 PE=1 S	6	7	8	6	986	113,6	6,2
O35658	Complement component 1 Q subcomponent-binding protein, mitochondrial	40	7	115	2	278	31	4,92
Q9D0U6	Repressor of RNA polymerase III transcription MAF1 homolog OS=Mus musc	26	3	5	3	258	28,8	4,64
Q9CR86	Calcium-regulated heat stable protein 1 OS=Mus musculus OX=10090 GN=C	28	4	7	4	148	16,1	8,21
Q80TM9	Nischarin OS=Mus musculus OX=10090 GN=Nisch PE=1 SV=2	17	17	26	17	1593	174,9	5,16
Q8VI86	COMM domain-containing protein 1 OS=Mus musculus OX=10090 GN=Comm	10	3	5	3	243	26,8	6,64
A0A0R4J1J0	Striated muscle-specific serine/threonine-protein kinase OS=Mus musculus	41	5	7	5	113	12,7	7,87
Q80XC2	tRNA (adenine(58)-N(1))-methyltransferase catalytic subunit TRMT61A OS=	42	9	22	9	290	31,6	6,79
O35295	Transcriptional activator protein Pur-beta OS=Mus musculus OX=10090 GN=	40	11	33	9	324	33,9	5,43
P51432	1-phosphatidylinositol 4,5-bisphosphate phosphodiesterase beta-3 OS=Mus	22	24	33	24	1234	139,4	5,94
Q9DBR0	A-kinase anchor protein 8 OS=Mus musculus OX=10090 GN=Akap8 PE=1 SV	24	14	24	14	687	76,2	5,14
B1AZP2	Disks large-associated protein 4 OS=Mus musculus OX=10090 GN=Dlgap4 P	22	16	38	16	992	108	7,08
Q61584	Fragile X mental retardation syndrome-related protein 1 OS=Mus musculus	31	20	27	17	677	76,2	6,98
E9QJT2	H/ACA ribonucleoprotein complex non-core subunit NAF1 OS=Mus musculus	12	7	16	7	597	63,6	5,21
Q8R1K4	5-phosphohydroxy-L-lysine phospho-lyase OS=Mus musculus OX=10090 GN	19	8	13	8	467	51,9	6,46
P49586	Choline-phosphate cytidylyltransferase A OS=Mus musculus OX=10090 GN=	33	11	18	11	367	41,6	7,03
Q99JF8	PC4 and SFRS1-interacting protein OS=Mus musculus OX=10090 GN=Psip1 P	42	21	49	8	528	59,7	9,13
P47968	Ribose-5-phosphate isomerase OS=Mus musculus OX=10090 GN=Rpia PE=1	49	12	30	12	303	32,4	8,02
D3YU88	NKAP domain-containing 1 (Fragment) OS=Mus musculus OX=10090 GN=Nk	43	7	12	7	214	25,2	9,94
F7AI87	26S proteasome non-ATPase regulatory subunit 4 (Fragment) OS=Mus musc	27	6	16	3	194	21,3	4,45
H3BJJ1	Geminin (Fragment) OS=Mus musculus OX=10090 GN=Gmnn PE=1 SV=1	50	7	21	7	167	19,2	5,15
B1AU25	Apoptosis-inducing factor 1, mitochondrial OS=Mus musculus OX=10090 GN	44	19	98	19	608	66,1	9,11
Q9QYH6	Melanoma-associated antigen D1 OS=Mus musculus OX=10090 GN=Maged	7	6	7	6	775	85,6	7,5
P58252	Elongation factor 2 OS=Mus musculus OX=10090 GN=Eef2 PE=1 SV=2	71	56	1131	55	858	95,3	6,83
Q61466	SWI/SNF-related matrix-associated actin-dependent regulator of chromatin	5	2	2	2	515	58,2	9,25
Q9CX97	WD repeat-containing protein 55 OS=Mus musculus OX=10090 GN=Wdr55 P	47	14	31	14	388	42,6	4,89
Q8BJU0	Small glutamine-rich tetratricopeptide repeat-containing protein alpha OS=M	36	10	50	10	315	34,3	5,06
P67871	Casein kinase II subunit beta OS=Mus musculus OX=10090 GN=Csnk2b PE=1	49	8	40	8	215	24,9	5,55
Q64378	Peptidyl-prolyl cis-trans isomerase FKBP5 OS=Mus musculus OX=10090 GN=	19	5	6	5	456	50,9	7,8
Q8R1N0	Zinc finger protein 830 OS=Mus musculus OX=10090 GN=Znf830 PE=1 SV=1	60	18	35	18	363	40,6	5,4
A0A1D5RM50	CKLF-like MARVEL transmembrane domain-containing protein 3 (Fragment)	18	2	3	2	142	15,5	6,01
E9Q8D0	DnaJ homolog subfamily C member 21 OS=Mus musculus OX=10090 GN=Dn	6	2	2	2	531	61,7	5,9
P47199	Quinone oxidoreductase OS=Mus musculus OX=10090 GN=Cryz PE=1 SV=1	53	13	34	13	331	35,2	8,07
Q9CXY6	Interleukin enhancer-binding factor 2 OS=Mus musculus OX=10090 GN=Ilf2	47	14	49	14	390	43	5,26
Q06180	Tyrosine-protein phosphatase non-receptor type 2 OS=Mus musculus OX=1	22	8	12	8	406	47,3	8,37
H7BWY4	Disks large homolog 1 OS=Mus musculus OX=10090 GN=Dlg1 PE=1 SV=1	7	5	5	5	872	96,8	5,9
F8VPN4	4-alpha-glucanotransferase OS=Mus musculus OX=10090 GN=Agl PE=1 SV=	17	22	25	22	1532	174,2	6,74
A0A140LIH0	Mediator complex subunit 25 (Fragment) OS=Mus musculus OX=10090 GN=	5	2	3	2	599	63,3	8,24
Q9WUK2	Eukaryotic translation initiation factor 4H OS=Mus musculus OX=10090 GN=	51	14	36	14	248	27,3	7,23
O54956	DNA polymerase epsilon subunit 2 OS=Mus musculus OX=10090 GN=Pole2	5	3	3	3	527	59,3	6,25
P62827	GTP-binding nuclear protein Ran OS=Mus musculus OX=10090 GN=Ran PE=1	44	10	162	10	216	24,4	7,49
P10639	Thioredoxin OS=Mus musculus OX=10090 GN=Txn PE=1 SV=3	40	5	100	5	105	11,7	4,92
Q9D394	Protein RUFY3 OS=Mus musculus OX=10090 GN=Rufy3 PE=1 SV=1	4	2	2	2	469	53	5,49
Q5SSI6	U3 small nucleolar RNA-associated protein 18 homolog OS=Mus musculus O	31	11	16	11	552	61,2	8,78
P23249	Putative helicase MOV-10 OS=Mus musculus OX=10090 GN=Mov10 PE=1 S	19	16	20	16	1004	113,5	9,01
P52825	Carnitine O-palmitoyltransferase 2, mitochondrial OS=Mus musculus OX=10	5	4	5	4	658	73,9	8,37
Q3UYC0	Protein phosphatase 1H OS=Mus musculus OX=10090 GN=Ppm1h PE=1 SV=	24	10	17	9	513	56,3	6,73
Q3THS6	S-adenosylmethionine synthase isoform type-2 OS=Mus musculus OX=1009	56	19	138	19	395	43,7	6,48
Q6AXB7	Synaptic functional regulator FMR1 OS=Mus musculus OX=10090 GN=Fmr1	21	11	14	9	614	68,8	7,42
A0A1L1SVK0	Platelet-activating factor acetylhydrolase IB subunit alpha2 (Fragment) OS=	21	2	6	2	197	22	6,14
Q91XH5	Sepiapterin reductase OS=Mus musculus OX=10090 GN=Spr PE=1 SV=1	70	14	83	14	262	27,9	6,21
Q9D142	Uridine diphosphate glucose pyrophosphatase NUDT14 OS=Mus musculus O	28	5	10	5	222	24,4	5,14
Q8K1E6	Alpha-ketoglutarate-dependent dioxygenase alkB homolog 3 OS=Mus musc	52	12	26	12	286	33	8,06
Q80Y55	BSD domain-containing protein 1 OS=Mus musculus OX=10090 GN=Bsdc1 P	24	9	11	9	427	46,9	4,4
Q8CI71	Syndetin OS=Mus musculus OX=10090 GN=Vps50 PE=1 SV=2	7	5	5	5	964	111,1	6,07
F8WJL8	UPF0462 protein C4orf33 homolog (Fragment) OS=Mus musculus OX=10090	19	3	3	3	118	13,5	5,24
Q3URQ0	Testis-expressed protein 10 OS=Mus musculus OX=10090 GN=Tex10 PE=1 S	10	7	9	7	928	105,1	9,16
Q8R326	Paraspeckle component 1 OS=Mus musculus OX=10090 GN=Pspc1 PE=1 SV=	26	13	32	12	523	58,7	6,67
Q61166	Microtubule-associated protein RP/EB family member 1 OS=Mus musculus	49	10	57	8	268	30	5,22
Q80X73	Protein pelota homolog OS=Mus musculus OX=10090 GN=Pelo PE=1 SV=3	13	5	5	5	385	43,3	5,99
P80316	T-complex protein 1 subunit epsilon OS=Mus musculus OX=10090 GN=Cct5 P	62	27	220	26	541	59,6	6,02
Q9CXJ1	Probable glutamate--tRNA ligase, mitochondrial OS=Mus musculus OX=1009	5	2	2	2	523	58,3	8,75
A2A8W8	MAP kinase-interacting serine/threonine-protein kinase 1 OS=Mus musculus	9	3	3	3	415	46,5	6,96
Q8BQ30	Phostensin OS=Mus musculus OX=10090 GN=Ppp1r18 PE=1 SV=1	46	24	36	24	594	65,6	5,39
P98078	Disabled homolog 2 OS=Mus musculus OX=10090 GN=Dab2 PE=1 SV=2	44	23	115	23	766	82,3	6,1
Q61598	Rab GDP dissociation inhibitor beta OS=Mus musculus OX=10090 GN=Gdi2 P	87	33	343	26	445	50,5	6,25
Q8VCA8	Secernin-2 OS=Mus musculus OX=10090 GN=Scrn2 PE=1 SV=1	23	7	11	7	425	46,6	5,71
Q9CQR6	Serine/threonine-protein phosphatase 6 catalytic subunit OS=Mus musculus	49	14	46	14	305	35,1	5,69
Q9Z1K6	E3 ubiquitin-protein ligase ARIH2 OS=Mus musculus OX=10090 GN=Arih2 PE	11	4	4	4	492	57,7	5,69
Q09014	Neutrophil cytosol factor 1 OS=Mus musculus OX=10090 GN=Ncf1 PE=1 SV=	38	13	19	13	390	44,6	9,01
Q8BP56	Protein-glucosylgalactosylhydroxylysine glucosidase OS=Mus musculus OX=	14	9	11	9	690	76,4	5,33
Q9CY21	Probable 18S rRNA (guanine-N(7))-methyltransferase OS=Mus musculus OX	33	6	19	6	281	31,6	8,88
Q9CWR1	WD repeat-containing protein 73 OS=Mus musculus OX=10090 GN=Wdr73 P	45	12	22	12	371	40,8	5,54
A0A140LJ70	Protein arginine N-methyltransferase 1 OS=Mus musculus OX=10090 GN=Pr	69	14	102	2	254	28,9	6,18
A2BDX3	Adenylyltransferase and sulfurtransferase MOCS3 OS=Mus musculus OX=1	23	8	11	8	460	49,3	7,49
Q8BGQ7	Alanine--tRNA ligase, cytoplasmic OS=Mus musculus OX=10090 GN=Aars1 P	49	40	213	40	968	106,8	5,67
P62307	Small nuclear ribonucleoprotein F OS=Mus musculus OX=10090 GN=Snrpf P	56	5	47	5	86	9,7	4,67
Q91WM1	Spermatid perinuclear RNA-binding protein OS=Mus musculus OX=10090 GN	14	9	20	5	672	73,7	8,72
E9QNA7	Sorbin and SH3 domain-containing protein 1 OS=Mus musculus OX=10090 G	35	21	30	21	740	82,8	7,85
Q91Z67	SLIT-ROBO Rho GTPase-activating protein 2 OS=Mus musculus OX=10090 G	6	5	5	5	1071	120,7	6,64
F6SIJ4	Transcription factor IIIA OS=Mus musculus OX=10090 GN=Gtf3a PE=1 SV=2	8	3	5	3	342	39,2	9,28
E9Q912	Rap1 GTPase-GDP dissociation stimulator 1 OS=Mus musculus OX=10090 G	38	24	54	24	607	66	5,35
Q8VDK1	Deaminated glutathione amidase OS=Mus musculus OX=10090 GN=Nit1 PE=	44	11	25	11	323	35,7	7,94
Q9JKR6	Hypoxia up-regulated protein 1 OS=Mus musculus OX=10090 GN=Hyou1 PE=	42	34	120	34	999	111,1	5,19
P42567	Epidermal growth factor receptor substrate 15 OS=Mus musculus OX=10090	30	22	39	22	897	98,4	4,6
O08856	RNA polymerase II elongation factor ELL OS=Mus musculus OX=10090 GN=E	14	8	9	8	602	67,1	9,19
Q921X9	Protein disulfide-isomerase A5 OS=Mus musculus OX=10090 GN=Pdia5 PE=1	20	10	17	10	517	59,2	7,5
Q5PR72	Phosphodiesterase OS=Mus musculus OX=10090 GN=Pde2a PE=1 SV=1	4	3	3	3	935	105,2	5,41
P70670	Nascent polypeptide-associated complex subunit alpha, muscle-specific form	5	8	175	8	2187	220,4	9,35
Q91VW3	SH3 domain-binding glutamic acid-rich-like protein 3 OS=Mus musculus OX=	51	6	154	6	93	10,5	5,14
P55302	Alpha-2-macroglobulin receptor-associated protein OS=Mus musculus OX=1	50	19	62	11	360	42,2	7,87
Q9Z315	U4/U6.U5 tri-snRNP-associated protein 1 OS=Mus musculus OX=10090 GN=	34	23	41	23	806	90,8	5,82
Q9D0F9	Phosphoglucomutase-1 OS=Mus musculus OX=10090 GN=Pgm1 PE=1 SV=4	63	29	130	29	562	61,4	6,57
P10649	Glutathione S-transferase Mu 1 OS=Mus musculus OX=10090 GN=Gstm1 PE	80	18	186	14	218	26	7,94
Q5U4D9	THO complex subunit 6 homolog OS=Mus musculus OX=10090 GN=Thoc6 PE	44	9	14	9	341	37,3	7,12
O08797	SPI6 OS=Mus musculus OX=10090 GN=Serpinb9 PE=1 SV=1	30	10	25	8	374	42,2	5,4
E9QMA2	Cotranscriptional regulator FAM172A OS=Mus musculus OX=10090 GN=Fam	25	7	12	7	371	42,9	6,43
Q3TJD7	PDZ and LIM domain protein 7 OS=Mus musculus OX=10090 GN=Pdlim7 PE=	7	3	4	3	457	50,1	8,47
Q6NZN0	RNA-binding protein 26 OS=Mus musculus OX=10090 GN=Rbm26 PE=1 SV=2	19	16	29	15	1012	114,1	9,16
Q8VBT0	Thioredoxin-related transmembrane protein 1 OS=Mus musculus OX=10090	16	3	4	3	278	31,4	5,29
A2A517	Cytohesin-1 (Fragment) OS=Mus musculus OX=10090 GN=Cyth1 PE=1 SV=1	9	4	4	3	460	52,5	5,2
P01899	H-2 class I histocompatibility antigen, D-B alpha chain OS=Mus musculus OX	39	12	27	9	362	40,8	6,73
Q9Z1X9	Cell division control protein 45 homolog OS=Mus musculus OX=10090 GN=Cd	3	2	2	2	566	65,3	5,43
Q9DCJ1	Target of rapamycin complex subunit LST8 OS=Mus musculus OX=10090 GN	32	7	9	7	326	35,8	5,86
Q9JL62	Glycolipid transfer protein OS=Mus musculus OX=10090 GN=Gltp PE=1 SV=3	39	8	12	8	209	23,7	7,39
Q61191	Host cell factor 1 OS=Mus musculus OX=10090 GN=Hcfc1 PE=1 SV=2	28	43	105	43	2045	210,3	7,18
Q80VJ2	Steroid receptor RNA activator 1 OS=Mus musculus OX=10090 GN=Sra1 PE=	46	9	23	2	232	25,5	6,43
Q6P3A8	2-oxoisovalerate dehydrogenase subunit beta, mitochondrial OS=Mus musc	16	5	6	5	390	42,9	6,7
Q9ERG2	Striatin-3 OS=Mus musculus OX=10090 GN=Strn3 PE=1 SV=1	34	21	44	2	796	87,1	5,29
E9PY43	Glutamate-rich 1 OS=Mus musculus OX=10090 GN=Erich1 PE=1 SV=1	20	6	9	6	332	37,3	5,45
Q9DA08	SAGA-associated factor 29 OS=Mus musculus OX=10090 GN=Sgf29 PE=2 SV	15	4	5	4	293	33,3	8,1
Q8BHL8	Proteasome inhibitor PI31 subunit OS=Mus musculus OX=10090 GN=Psmf1	8	3	3	3	271	29,6	5,25
Q8CFE2	Histone PARylation factor 1 OS=Mus musculus OX=10090 GN=Hpf1 PE=1 SV	34	10	17	10	346	39,3	7,69
A0A0N4SV40	Plasminogen activator inhibitor 1 RNA-binding protein (Fragment) OS=Mus m	48	14	77	2	186	20,6	10,73
O35309	N-myc-interactor OS=Mus musculus OX=10090 GN=Nmi PE=1 SV=1	38	12	26	12	314	35,2	5,05
Q8C2Q7	Heterogeneous nuclear ribonucleoprotein H OS=Mus musculus OX=10090 G	41	14	65	7	472	51,2	6,8
Q9R0Q6	Actin-related protein 2/3 complex subunit 1A OS=Mus musculus OX=10090	31	10	14	9	370	41,6	8,18
Q8C3F2	Constitutive coactivator of PPAR-gamma-like protein 2 OS=Mus musculus O	7	6	9	4	1091	119,6	8,46
Q9CQ13	Coordinator of PRMT5 and differentiation stimulator OS=Mus musculus OX=	42	4	7	4	173	18,7	4,08
Q64311	Protein N-terminal asparagine amidohydrolase OS=Mus musculus OX=1009	28	8	12	8	310	34,6	6,19
Q9JIK9	28S ribosomal protein S34, mitochondrial OS=Mus musculus OX=10090 GN=	37	7	14	7	218	25,8	10,43
A0A1Y7VNF4	ERO1-like protein beta OS=Mus musculus OX=10090 GN=Ero1b PE=1 SV=1	20	9	14	8	493	55,9	7,8
O55135	Eukaryotic translation initiation factor 6 OS=Mus musculus OX=10090 GN=Ei	60	9	100	9	245	26,5	4,74
Q8CE96	tRNA (adenine(58)-N(1))-methyltransferase non-catalytic subunit TRM6 OS=	45	20	79	20	497	55,5	6,95
Q922H1	Protein arginine N-methyltransferase 3 OS=Mus musculus OX=10090 GN=Pr	33	14	34	14	532	59,9	5,3
Q61599	Rho GDP-dissociation inhibitor 2 OS=Mus musculus OX=10090 GN=Arhgdib P	79	14	134	9	200	22,8	5,11

Q9D4L6	E3 ubiquitin-protein ligase Rnf220 OS=Mus musculus OX=10090 GN=Rnf220	24	6	9	6	353	39,4	5,66	protein metabolism;other metabolic processes		other molecular function	Pf13923, Pf15926	66743
Q9DCI9	39S ribosomal protein L32, mitochondrial OS=Mus musculus OX=10090 GN=	19	3	7	3	187	21,7	9,7	protein metabolism;other metabolic processes	mitochondrion;translational apparatus	other molecular function	Pf01783	75398
H3BKI6	ATPase family, AAA domain-containing 3A OS=Mus musculus OX=10090 GN	8	2	3	2	211	23,8	8,07	cell organization and biogenesis	mitochondrion	other molecular function	Pf12037	
Q7TPE5	Probable RNA polymerase II nuclear localization protein SLC7A6OS OS=Mus	57	11	33	11	306	35	4,58	transport;developmental processes;other biological processes	nucleus;other cell component		Pf08574	66432
Q9D8S4	Oligoribonuclease, mitochondrial OS=Mus musculus OX=10090 GN=Rexo2 P	34	7	48	7	237	26,7	7,15	RNA metabolism OR transcription;other metabolic processes	mitochondrion;nucleus;other cell component	nucleic acid binding activity;other molecular function	Pf00929	104444
Q6PAC3	DDB1- and CUL4-associated factor 13 OS=Mus musculus OX=10090 GN=Dcaf	37	15	20	15	445	51,4	9,32	protein metabolism;RNA metabolism OR transcription;other metabolic processes	cytosol;cytoskeleton;nucleus;other cell component	other molecular function	Pf00400, Pf04158	223499
P52479	Ubiquitin carboxyl-terminal hydrolase 10 OS=Mus musculus OX=10090 GN=U	21	9	12	9	792	87	5,17	protein metabolism;DNA metabolism;other metabolic processes;stress response;	cytosol;cytoskeleton;nucleus;other cytoplasmic organelle;other cell component	other molecular function	Pf00443, Pf07145	22224
P15626	Glutathione S-transferase Mu 2 OS=Mus musculus OX=10090 GN=Gstm2 PE	54	12	40	7	218	25,7	7,39	other metabolic processes;stress response	non-structural extracellular;plasma membrane;other membranes;cytosol;other cell component	other molecular function	Pf00043, Pf02798	14863
O35864	COP9 signalosome complex subunit 5 OS=Mus musculus OX=10090 GN=Cop	55	15	57	15	334	37,5	6,54	protein metabolism;other metabolic processes;transport;other biological processe	cytosol;ER/Golgi;nucleus;other cell component	other molecular function	Pf01398, Pf18323	26754
Q99LJ0	CTTNBP2 N-terminal-like protein OS=Mus musculus OX=10090 GN=Cttnbp2n	37	20	25	19	638	69,8	7,71	protein metabolism;other metabolic processes;other biological processes	cytoskeleton;other cell component	other molecular function	Pf09727	80281
O88967	ATP-dependent zinc metalloprotease YME1L1 OS=Mus musculus OX=10090	14	9	16	9	715	80	8,97	cell cycle OR cell proliferation;cell organization and biogenesis;protein metabolism	other membranes;mitochondrion;nucleus	other molecular function	Pf00004, Pf01434, Pf17862	27377
Q3UY34	Protein CUSTOS OS=Mus musculus OX=10090 GN=Custos PE=2 SV=1	50	10	27	10	256	27,6	8,7	cell-cell signaling;signal transduction;other biological processes	nucleus			72357
Q8K212	Phosphofurin acidic cluster sorting protein 1 OS=Mus musculus OX=10090 GN	13	8	10	8	961	104,8	7,74	other biological processes	ER/Golgi;other cell component	other molecular function	Pf10254	107975
Q8C7E9	Cleavage stimulation factor subunit 2 tau variant OS=Mus musculus OX=100	26	12	41	6	632	65,8	7,25	RNA metabolism OR transcription;other metabolic processes	nucleus;other cell component	nucleic acid binding activity	Pf00076, Pf14304, Pf14327	83410
Q9JK81	MYG1 exonuclease OS=Mus musculus OX=10090 GN=Myg1 PE=1 SV=1	55	16	92	16	380	42,7	7,02	other metabolic processes;other biological processes	mitochondrion;nucleus;other cell component	other molecular function	Pf03690	60315
Q8BYL4	Tyrosine--tRNA ligase, mitochondrial OS=Mus musculus OX=10090 GN=Yars	18	8	8	8	472	52,6	9,16	protein metabolism;RNA metabolism OR transcription;other metabolic processes	cytosol;mitochondrion;nucleus	nucleic acid binding activity;other molecular function	Pf00579	70120
Q9CR39	WD repeat domain phosphoinositide-interacting protein 3 OS=Mus musculus	20	6	11	5	344	38	7,56	cell organization and biogenesis;protein metabolism;other metabolic processes;s	other membranes;cytosol;other cytoplasmic organelle;other cell component	other molecular function		66840
Q8CI04	Conserved oligomeric Golgi complex subunit 3 OS=Mus musculus OX=10090	4	3	3	3	820	93,2	5,78	cell organization and biogenesis;protein metabolism;other metabolic processes;tr	plasma membrane;other membranes;cytosol;ER/Golgi		Pf04136	
Q6GT24	Peroxiredoxin-6 OS=Mus musculus OX=10090 GN=Prdx6 PE=1 SV=1	83	16	209	12	224	24,8	6,37	other biological processes	other cell component	other molecular function	Pf00578, Pf10417	11758
O35218	Cleavage and polyadenylation specificity factor subunit 2 OS=Mus musculus	15	8	12	8	782	88,3	5,11	RNA metabolism OR transcription;other metabolic processes	nucleus	nucleic acid binding activity	Pf07521, Pf10996, Pf13299, Pf16661	51786
P59999	Actin-related protein 2/3 complex subunit 4 OS=Mus musculus OX=10090 GN	74	12	68	12	168	19,7	8,43	cell organization and biogenesis	cytoskeleton;nucleus;other cell component	cytoskeletal activity;other molecular function	Pf05856	68089
Q6P1B9	Bin1 protein OS=Mus musculus OX=10090 GN=Bin1 PE=1 SV=1	37	15	31	14	477	52,7	5,24	other biological processes	other cell component		Pf03114, Pf14604	30948
Q9CQQ4	Gem-associated protein 2 OS=Mus musculus OX=10090 GN=Gemin2 PE=2 S	14	3	3	3	269	30,4	5,53	cell organization and biogenesis;RNA metabolism OR transcription;other metaboli	cytosol;nucleus;other cell component		Pf04938	66603
P62880	Guanine nucleotide-binding protein G(I)/G(S)/G(T) subunit beta-2 OS=Mus m	26	8	12	5	340	37,3	6	signal transduction	plasma membrane;other membranes;other cell component	other molecular function	Pf00400	14693
O88879	Apoptotic protease-activating factor 1 OS=Mus musculus OX=10090 GN=Apa	7	9	10	8	1249	140,9	6,43	stress response;developmental processes;signal transduction;other biological pro	cytosol;nucleus;other cell component	enzyme regulator activity;other molecular function	Pf00400, Pf00619, Pf00931, Pf17908	11783
Q8BW96	Calcium/calmodulin-dependent protein kinase type 1D OS=Mus musculus O	23	7	18	4	385	42,9	7,17	protein metabolism;other metabolic processes;stress response;developmental pr	nucleus;other cell component	kinase activity;other molecular function	Pf00069	227541
P56477	Interferon regulatory factor 5 OS=Mus musculus OX=10090 GN=Irf5 PE=1 SV	18	6	11	6	497	56	5,31	stress response;signal transduction;other biological processes	nucleus;other cell component	nucleic acid binding activity;other molecular function	Pf00605, Pf10401	27056
Q8K339	DNA/RNA-binding protein KIN17 OS=Mus musculus OX=10090 GN=Kin PE=1	27	11	26	11	391	44,7	9,04	DNA metabolism;RNA metabolism OR transcription;other metabolic processes;str	nucleus;other cell component	nucleic acid binding activity;other molecular function	Pf10357, Pf18131	16588
O70145	Neutrophil cytosol factor 2 OS=Mus musculus OX=10090 GN=Ncf2 PE=1 SV=	25	12	17	12	525	59,4	6,61	other metabolic processes;transport;other biological processes	plasma membrane;other membranes;cytosol;other cell component	enzyme regulator activity;other molecular function	Pf00018, Pf00515, Pf00564, Pf13181	17970
Q9DAT5	Mitochondrial tRNA-specific 2-thiouridylase 1 OS=Mus musculus OX=10090	36	13	21	13	417	47,2	8,4	RNA metabolism OR transcription;other metabolic processes	mitochondrion	nucleic acid binding activity;other molecular function	Pf03054, Pf20258, Pf20259	72026
Q91VT4	3-oxoacyl-[acyl-carrier-protein] reductase OS=Mus musculus OX=10090 GN=	35	7	12	7	236	25,4	9,69	cell organization and biogenesis;other metabolic processes	mitochondrion;other cell component	other molecular function	Pf13561	234309
Q8BZG5	Methyltransferase-like protein 25B OS=Mus musculus OX=10090 GN=Mettl	5	2	6	2	475	53,2	8,12		other membranes		Pf13679	229503
Q80Y14	Glutaredoxin-related protein 5, mitochondrial OS=Mus musculus OX=10090	30	4	41	3	152	16,3	6,55	cell organization and biogenesis;protein metabolism;other metabolic processes;d	mitochondrion;nucleus;other cell component	other molecular function	Pf00462	73046
Q8BUH1	Thioredoxin-like protein 4B OS=Mus musculus OX=10090 GN=Txnl4b PE=2 S	9	2	2	2	149	17	6,33	cell cycle OR cell proliferation;RNA metabolism OR transcription;other metabolic p	cytosol;nucleus		Pf02966	234723
P59644	Phosphatidylinositol 4,5-bisphosphate 5-phosphatase A OS=Mus musculus	2	2	2	2	1003	107,5	9,36	other metabolic processes;other biological processes	plasma membrane;other membranes;other cell component	other molecular function	Pf03372, Pf17751	170835
Q9WUM4	Coronin-1C OS=Mus musculus OX=10090 GN=Coro1c PE=1 SV=2	41	21	97	20	474	53,1	7,08	cell organization and biogenesis;transport;developmental processes;other biolog	plasma membrane;other membranes;cytoskeleton;other cytoplasmic organelle;other cell compon	cytoskeletal activity;other molecular function	Pf00400, Pf08953, Pf16300	23790
Q8JZK9	Hydroxymethylglutaryl-CoA synthase, cytoplasmic OS=Mus musculus OX=1	37	15	74	15	520	57,5	5,99	other metabolic processes;developmental processes;other biological processes	cytosol	other molecular function	Pf01154, Pf08540	208715
Q9QZE7	Translin-associated protein X OS=Mus musculus OX=10090 GN=Tsnax PE=1 S	58	14	57	14	290	32,9	6,55	RNA metabolism OR transcription;other metabolic processes;other biological proc	ER/Golgi;nucleus;other cell component	signal transduction activity or receptor binding;nucleic acid binding activity;othe	Pf01997	53424
P52760	2-iminobutanoate/2-iminopropanoate deaminase OS=Mus musculus OX=10	70	8	19	8	135	14,2	8,68	cell cycle OR cell proliferation;RNA metabolism OR transcription;other metabolic p	cytosol;mitochondrion;nucleus;other cytoplasmic organelle;other cell component	nucleic acid binding activity;other molecular function	Pf01042	15473
P38647	Stress-70 protein, mitochondrial OS=Mus musculus OX=10090 GN=Hspa9 PE	64	43	515	42	679	73,4	6,07	cell organization and biogenesis;protein metabolism;other metabolic processes;s	mitochondrion;nucleus;other cell component	other molecular function	Pf00012	15526
P47809	Dual specificity mitogen-activated protein kinase kinase 4 OS=Mus musculus	13	4	4	4	397	44,1	8,07	protein metabolism;other metabolic processes;stress response;developmental pr	cytosol;nucleus;other cell component	kinase activity;other molecular function	Pf00069	26398
Q8BG30	Negative elongation factor A OS=Mus musculus OX=10090 GN=Nelfa PE=1 S	8	4	4	3	530	57,5	9,11	other biological processes	cytosol;nucleus	other molecular function		24116
Q61142	Spindlin-1 OS=Mus musculus OX=10090 GN=Spin1 PE=1 SV=2	21	5	6	5	262	29,6	6,96	cell-cell signaling;cell cycle OR cell proliferation;cell organization and biogenesis;R	other membranes;cytosol;cytoskeleton;nucleus	nucleic acid binding activity;other molecular function	Pf02513	20729
Q99KP3	Lambda-crystallin homolog OS=Mus musculus OX=10090 GN=Cryl1 PE=1 SV	40	9	22	9	319	35,2	5,86	other metabolic processes	cytosol	other molecular function	Pf00725, Pf02737	68631
Q9DCD5	Tight junction-associated protein 1 OS=Mus musculus OX=10090 GN=Tjap1 F	23	9	13	9	549	60,5	6,14	cell organization and biogenesis	plasma membrane;other membranes;ER/Golgi;other cytoplasmic organelle;other cell component		Pf15453	74094
Q76LS9	Ubiquitin carboxyl-terminal hydrolase MINDY-1 OS=Mus musculus OX=1009	11	3	4	3	468	51,2	4,73	protein metabolism;other metabolic processes	cytosol;nucleus;other cell component	other molecular function	Pf04424	75007
Q8R105	Vacuolar protein sorting-associated protein 37C OS=Mus musculus OX=1009	17	4	7	4	352	38,4	5,31	protein metabolism;other metabolic processes;transport	other membranes;other cytoplasmic organelle;other cell component	other molecular function	Pf07200	107305
Q9CR00	26S proteasome non-ATPase regulatory subunit 9 OS=Mus musculus OX=10	50	10	40	10	222	24,7	6,43	cell organization and biogenesis;other biological processes	nucleus;other cell component	other molecular function	Pf02163, Pf17820, Pf18265	67151
G5E895	Aldo-keto reductase family 1, member B10 (aldose reductase) OS=Mus mus	59	16	47	9	316	35,8	7,28	other metabolic processes	cytosol	other molecular function	Pf00248	67861
Q8K2Y7	39S ribosomal protein L47, mitochondrial OS=Mus musculus OX=10090 GN=	19	5	15	5	252	29,7	10,21	protein metabolism;other metabolic processes	mitochondrion;translational apparatus	other molecular function	Pf06984	74600
Q8CFI7	DNA-directed RNA polymerase II subunit RPB2 OS=Mus musculus OX=10090	47	49	104	49	1174	133,8	6,87	RNA metabolism OR transcription;other metabolic processes	nucleus;other cell component	nucleic acid binding activity;other molecular function	Pf00562, Pf04560, Pf04561, Pf04563, Pf0	231329
Q58A65	C-Jun-amino-terminal kinase-interacting protein 4 OS=Mus musculus OX=10	15	17	21	4	1321	146,1	5,15	transport;other biological processes	other membranes;cytosol;cytoskeleton;other cytoplasmic organelle;other cell component	cytoskeletal activity;other molecular function	Pf09744, Pf16471, Pf19056	70834
Q8BU27	Protein phosphatase 1M OS=Mus musculus OX=10090 GN=Ppm1m PE=2 SV	7	3	3	2	462	51,2	6,38	protein metabolism;other metabolic processes	nucleus	other molecular function	Pf00481	67905
Q6PGG2	GEM-interacting protein OS=Mus musculus OX=10090 GN=Gmip PE=1 SV=1	12	8	9	8	971	107,5	5,77	signal transduction;other biological processes	plasma membrane;other membranes;cytosol;nucleus	enzyme regulator activity;other molecular function	Pf00620	78816
Q3U1C4	SECIS-binding protein 2 OS=Mus musculus OX=10090 GN=Secisbp2 PE=1 SV	4	2	2	2	858	94,8	8,6	protein metabolism;other metabolic processes;developmental processes;other b	nucleus;other cell component	nucleic acid binding activity;other molecular function	Pf01248	75420
Q62422	Osteoclast-stimulating factor 1 OS=Mus musculus OX=10090 GN=Ostf1 PE=	59	10	29	10	215	23,8	5,68	signal transduction	other cell component	other molecular function	Pf00018, Pf00023	20409
Q791N7	DNA-directed RNA polymerase I subunit RPA12 OS=Mus musculus OX=1009	37	4	17	4	123	13,6	5,27	RNA metabolism OR transcription;other metabolic processes	nucleus	nucleic acid binding activity;other molecular function	Pf01096	66136
Q9D1J3	SAP domain-containing ribonucleoprotein OS=Mus musculus OX=10090 GN=	56	9	59	3	210	23,5	6,65	other metabolic processes;transport;other biological processes	nucleus;other cell component	nucleic acid binding activity;other molecular function	Pf02037	66118
Q9D0R8	Protein LSM12 homolog OS=Mus musculus OX=10090 GN=Lsm12 PE=1 SV=	31	5	18	5	195	21,7	7,74				Pf09793	268490
A0A087WPF5	AN1-type zinc finger protein 2B (Fragment) OS=Mus musculus OX=10090 GN	28	5	8	5	255	27,3	7,11			other molecular function	Pf01428	
Q9JHE7	Protein TSSC4 OS=Mus musculus OX=10090 GN=Tssc4 PE=1 SV=1	39	9	16	9	317	33,5	5,14				Pf15264	56844
Q99LC5	Electron transfer flavoprotein subunit alpha, mitochondrial OS=Mus musculu	56	15	67	15	333	35	8,38	other metabolic processes	mitochondrion	other molecular function	Pf00766, Pf01012	110842
Q5NCB2	Predicted gene 12185 OS=Mus musculus OX=10090 GN=Gm12185 PE=1 SV	3	2	2	2	835	94,4	6,29	stress response;other biological processes	other membranes;ER/Golgi	other molecular function	Pf05049	620913
D3YUP8	Inhibitor of growth protein OS=Mus musculus OX=10090 GN=Ing3 PE=1 SV=	9	2	2	2	410	45,5	5,63	cell organization and biogenesis;other biological processes	nucleus	other molecular function	Pf12998	71777
A2ADE0	Pleckstrin homology domain-containing family M member 2 OS=Mus muscu	6	5	8	5	1013	112,1	4,97				Pf00169, Pf02759	69582
Q9Z1B5	Mitotic spindle assembly checkpoint protein MAD2A OS=Mus musculus OX=	32	7	19	7	205	23,6	5,2	cell cycle OR cell proliferation;cell organization and biogenesis;signal transduction	cytosol;cytoskeleton;nucleus;other cell component	other molecular function	Pf02301	56150
Q99L13	3-hydroxyisobutyrate dehydrogenase, mitochondrial OS=Mus musculus OX=	33	8	36	8	335	35,4	8,13	other metabolic processes		other molecular function	Pf03446, Pf14833	58875
Q8R180	ERO1-like protein alpha OS=Mus musculus OX=10090 GN=Ero1a PE=1 SV=2	44	18	43	17	464	54,1	6,54	cell organization and biogenesis;protein metabolism;other metabolic processes;s	other membranes;ER/Golgi;other cell component	other molecular function	Pf04137	50527
Q9JJT9	Phosphorylated adapter RNA export protein OS=Mus musculus OX=10090 GN	57	19	54	19	385	43,2	5,34	transport;other biological processes	cytoskeleton;nucleus;other cell component	nucleic acid binding activity;other molecular function	Pf10258	56698
A2AC16	L-xylulose reductase OS=Mus musculus OX=10090 GN=Dcxr PE=1 SV=1	35	7	12	7	236	25	6,55			other molecular function	Pf00106	67880
Q99LX5	Multiple myeloma tumor-associated protein 2 homolog OS=Mus musculus C	25	6	7	6	260	29,3	9,86				Pf10159	67862
E9QMK9	D-glutamate cyclase, mitochondrial OS=Mus musculus OX=10090 GN=Dgluc	40	20	41	20	647	69,9	7,34			other molecular function	Pf07286, Pf14336	
Q80YD6	Secretion-regulating guanine nucleotide exchange factor OS=Mus musculus	30	9	12	9	464	49,2	6,19	other biological processes	cytosol;nucleus;other cell component	enzyme regulator activity	Pf00415, Pf13540	27414
P54822	Adenylosuccinate lyase OS=Mus musculus OX=10090 GN=Adsl PE=1 SV=2	55	22	129	22	484	54,8	7,27	other metabolic processes;stress response;other biological processes	cytosol;other cell component	other molecular function	Pf00206, Pf10397	11564
Q91XU3	Phosphatidylinositol 5-phosphate 4-kinase type-2 gamma OS=Mus musculu	18	6	8	6	421	47,3	6,89	other metabolic processes;other biological processes	plasma membrane;other membranes;cytosol;ER/Golgi;nucleus;other cytoplasmic organelle;other	kinase activity;other molecular function	Pf01504	117150
P70388	DNA repair protein RAD50 OS=Mus musculus OX=10090 GN=Rad50 PE=1 SV	24	31	40	31	1312	153,4	6,95	cell cycle OR cell proliferation;cell organization and biogenesis;DNA metabolism;o	nucleus;other cell component	nucleic acid binding activity;other molecular function	Pf04423, Pf13476, Pf13558	
P54071	Isocitrate dehydrogenase [NADP], mitochondrial OS=Mus musculus OX=100	51	24	167	22	452	50,9	8,69	other metabolic processes;developmental processes;other biological processes	cytosol;other cytoplasmic organelle	other molecular function	Pf00180	269951
Q80WR5	UPF0688 protein C1orf174 homolog OS=Mus musculus OX=10090 PE=1 SV=	59	9	15	9	230	24,8	6,04		nucleus		Pf15772	
A0A1L1SSC0	Leucine-rich repeat flightless-interacting protein 2 OS=Mus musculus OX=10	27	14	52	13	670	75,8	6,38	other biological processes			Pf09738	
D3Z0V7	TSC22 domain family protein 1 OS=Mus musculus OX=10090 GN=Tsc22d1 P	9	8	19	5	995	102	5,77	other biological processes			Pf01166	21807
Q9D032	Single-stranded DNA-binding protein 3 OS=Mus musculus OX=10090 GN=Ssb	12	4	5	3	388	40,4	6,9	cell organization and biogenesis;developmental processes;other biological proces	nucleus;other cell component	nucleic acid binding activity;other molecular function	Pf04503	72475
Q9Z1M8	Protein Red OS=Mus musculus OX=10090 GN=Ik PE=1 SV=2	17	8	8	8	557	65,6	6,64	cell cycle OR cell proliferation;RNA metabolism OR transcription;other metabolic p	cytoskeleton;nucleus;other cell component	other molecular function	Pf07807, Pf07808	24010
Q792F9	Alpha-4 integrin OS=Mus musculus OX=10090 GN=Itga4 PE=1 SV=1	11	8	13	8	1032	115	6,81	cell adhesion;cell organization and biogenesis;transport;developmental processe	non-structural extracellular;plasma membrane;other cell component	other molecular function	Pf01839, Pf08441	16401
Q64737	Trifunctional purine biosynthetic protein adenosine-3 OS=Mus musculus OX	53	42	208	42	1010	107,4	6,68	other metabolic processes;developmental processes;other biological processes	cytosol	other molecular function	Pf00551, Pf00586, Pf01071, Pf02769, Pf0	14450
Q9DBX2	Phosducin-like protein OS=Mus musculus OX=10090 GN=Pdcl PE=1 SV=1	16	3	4	3	301	34,4	4,87	cell organization and biogenesis;other biological processes	other cell component	other molecular function	Pf02114	67466
P60840	Alpha-endosulfine OS=Mus musculus OX=10090 GN=Ensa PE=1 SV=1	55	7	28	6	121	13,3	7,24	cell cycle OR cell proliferation;other biological processes	other cell component	signal transduction activity or receptor binding;enzyme regulator activity;other	Pf04667	56205
Q3U276	Succinate dehydrogenase assembly factor 1, mitochondrial OS=Mus musculu	27	3	3	3	118	13,1	11,24	cell organization and biogenesis	mitochondrion	other molecular function	Pf13232	68332
Q8C0E2	Vacuolar protein sorting-associated protein 26B OS=Mus musculus OX=1009	45	15	41	15	336	39,1	7,37	stress response;transport	other membranes;cytosol;other cytoplasmic organelle;other cell component		Pf03643	69091
Q2NL51	Glycogen synthase kinase-3 alpha OS=Mus musculus OX=10090 GN=Gsk3a	31	9	13	6	490	51,6	8,81	cell-cell signaling;cell organization and biogenesis;protein metabolism;other meta	cytosol;cytoskeleton;mitochondrion;nucleus;other cell component	signal transduction activity or receptor binding;kinase activity;other molecular f	Pf00069	606496
Q9ESY9	Gamma-interferon-inducible lysosomal thiol reductase OS=Mus musculus O	42	6	25	6	248	27,8	5,01	other biological processes	non-structural extracellular;cytosol;other cytoplasmic organelle;other cell component	other molecular function	Pf03227	65972
D3YU12	NmrA-like family domain-containing protein 1 OS=Mus musculus OX=10090	38	11	20	11	299	33,1	6,8				Pf05368	67824
Q9CY58	Plasminogen activator inhibitor 1 RNA-binding protein OS=Mus musculus OX	63	26	227	3	407	44,7	8,54	cell organization and biogenesis;other biological processes	plasma membrane;other membranes;cytosol;nucleus;other cell component	translation activity;nucleic acid binding activity;other molecular function	Pf04774, Pf16174	66870
O88811	Signal transducing adapter molecule 2 OS=Mus musculus OX=10090 GN=Sta	38	19	30	17	523	57,4	5,07	transport;signal transduction	other membranes;cytosol;nucleus;other cytoplasmic organelle;other cell component	other molecular function	Pf00018, Pf00790, Pf02809	56324
A0A0R4IZY9	tRNA-dihydrouridine(47) synthase [NAD(P)(+)] OS=Mus musculus OX=10090	43	21	44	21	637	71,1	7,9	RNA metabolism OR transcription;other metabolic processes		other molecular function	Pf01207, Pf18044	224907
Q7TNG5	Echinoderm microtubule-associated protein-like 2 OS=Mus musculus OX=10	26	15	22	15	649	70,7	6,28	cell organization and biogenesis;other biological processes	cytoskeleton;other cell component	signal transduction activity or receptor binding;cytoskeletal activity;other molec	Pf00400, Pf03451	72205
Q91YS8	Calcium/calmodulin-dependent protein kinase type 1 OS=Mus musculus OX	40	10	32	7	374	41,6	5,35	cell cycle OR cell proliferation;protein metabolism;other metabolic processes;tran	nucleus;other cell component	kinase activity;other molecular function	Pf00069	52163
Q9D554	Splicing factor 3A subunit 3 OS=Mus musculus OX=10090 GN=Sf3a3 PE=1 S	33	15	37	15	501	58,8	5,34	cell organization and biogenesis;RNA metabolism OR transcription;other metaboli	nucleus;other cell component	nucleic acid binding activity;other molecular function	Pf11931, Pf12108, Pf13297, Pf16837	75062
B1ASZ3	Glycerol kinase OS=Mus musculus OX=10090 GN=Gk PE=1 SV=1	38	19	37	18	552	59,8	5,64	other metabolic processes	other membranes;mitochondrion	kinase activity;other molecular function	Pf00370, Pf02782	14933
Q8BGC4	Prostaglandin reductase-3 OS=Mus musculus OX=10090 GN=Zadh2 PE=1 SV	24	7	16	7	377	40,5	7,42	other metabolic processes;other biological processes	other cytoplasmic organelle	other molecular function	Pf00107, Pf08240	225791
A0A0R4J1G4	RAD51-associated protein 1 OS=Mus musculus OX=10090 GN=Rad51ap1 PE	36	10	22	10	337	36,2	9,32	DNA metabolism;other metabolic processes;stress response;other biological proc	nucleus;other cell component	nucleic acid binding activity	Pf15696	19362
Q14CH7	Alanine--tRNA ligase, mitochondrial OS=Mus musculus OX=10090 GN=Aars2	9	7	11	7	980	106,7	6,18	protein metabolism;RNA metabolism OR transcription;other metabolic processes		nucleic acid binding activity;other molecular function	Pf01411, Pf07973	224805
Q3UL36	Arginine and glutamate-rich protein 1 OS=Mus musculus OX=10090 GN=Arg	22	9	22	9	271	32,9	10,36		cytosol;mitochondrion;nucleus		Pf15346	234023
Q9D0L8	mRNA cap guanine-N7 methyltransferase OS=Mus musculus OX=10090 GN=	56	21	78	21	465	53,3	6,48	RNA metabolism OR transcription;other metabolic processes;other biological proc	nucleus;other cell component	nucleic acid binding activity;other molecular function	Pf03291	67897
Q8JZN5	Complex I assembly factor ACAD9, mitochondrial OS=Mus musculus OX=100	39	21	47	21	625	68,7	7,46	cell organization and biogenesis;other metabolic processes	other membranes;mitochondrion;nucleus;other cell component	other molecular function	Pf00441, Pf02770, Pf02771	229211
Q8C5P5	5'-nucleotidase domain-containing protein 1 OS=Mus musculus OX=10090 G	23	10	16	10	467	53,1	6,33	other metabolic processes		other molecular function	Pf05761	319638
Q8CFQ3	RNA helicase aquarius OS=Mus musculus OX=10090 GN=Aqr PE=1 SV=2	14	19	25	19	1481	170,2	6,27	RNA metabolism OR transcription;other metabolic processes	nucleus	nucleic acid binding activity;other molecular function	Pf13086, Pf13087, Pf16399	11834
Q9JIB4	General transcription factor IIH subunit 2 OS=Mus musculus OX=10090 GN=	15	6	8	6	396	44,7	6,83	DNA metabolism;RNA metabolism OR transcription;other metabolic processes;str	nucleus	other molecular function	Pf04056, Pf07975	23894
E9PU87	Non-specific serine/threonine protein kinase OS=Mus musculus OX=10090 G	8	9	11	9	1369	150,6	7,02	protein metabolism;other metabolic processes;other biological processes		kinase activity;other molecular function	Pf00069	70661
Q91X20	Set1/Ash2 histone methyltransferase complex subunit ASH2 OS=Mus muscu	34	16	22	16	623	68,2	5,69	cell organization and biogenesis;protein metabolism;other metabolic processes;s	nucleus;other cell component	nucleic acid binding activity;other molecular function	Pf00622	23808
Q8VC57	BTB/POZ domain-containing protein KCTD5 OS=Mus musculus OX=10090 GN	39	7	12	6	234	26,1	5,95	cell organization and biogenesis;protein metabolism;other metabolic processes	cytosol;nucleus;other cell component	other molecular function	Pf02214	69259
A2AQN4	Propionate--CoA ligase OS=Mus musculus OX=10090 GN=Acss2 PE=1 SV=1	15	9	14	9	714	80,3	6,95	other metabolic processes	other membranes;cytosol;nucleus	other molecular function	Pf00501, Pf13193, Pf16177	
A0A6I8MX03	UBC core domain-containing protein OS=Mus musculus OX=10090 PE=3 SV=	42	5	7	5	223	24,2	7,91			other molecular function	Pf00179	
Q80YD1	ATP-dependent RNA helicase SUPV3L1, mitochondrial OS=Mus musculus OX	9	6	8	6	779	87	7,84	cell organization and biogenesis;DNA metabolism;RNA metabolism OR transcripti	mitochondrion;nucleus;other cytoplasmic organelle	nucleic acid binding activity;other molecular function	Pf00271, Pf12513, Pf18114, Pf18147	338359
Q9D8P4	39S ribosomal protein L17, mitochondrial OS=Mus musculus OX=10090 GN=	21	4	7	4	176	20,2	9,82	cell organization and biogenesis;protein metabolism;other metabolic processes	mitochondrion;translational apparatus	other molecular function	Pf01196	27397
Q9D1P4	Cysteine and histidine-rich domain-containing protein 1 OS=Mus musculus O	51	16	47	16	331	37,3	7,9	cell cycle OR cell proliferation;cell organization and biogenesis;other biological pro		other molecular function	Pf04968, Pf04969	66917
Q91VL9	Zinc finger and BTB domain-containing protein 1 OS=Mus musculus OX=100	6	3	4	3	713	81,9	6,38	cell organization and biogenesis;DNA metabolism;RNA metabolism OR transcripti	other membranes;cytoskeleton;nucleus	nucleic acid binding activity;other molecular function	Pf00651	268564
Q9EPQ7	StAR-related lipid transfer protein 5 OS=Mus musculus OX=10090 GN=Stard	31	4	13	4	213	23,9	6,39	cell organization and biogenesis;transport		transporter activity;other molecular function	Pf01852	170460
O35609	Secretory carrier-associated membrane protein 3 OS=Mus musculus OX=100	7	2	3	2	349	38,4	7,64	transport;other biological processes	other membranes;ER/Golgi;other cytoplasmic organelle;other cell component	other molecular function	Pf04144	24045
Q9DBL1	Short/branched chain specific acyl-CoA dehydrogenase, mitochondrial OS=N	36	13	30	13	432	47,8	7,87	other metabolic processes	mitochondrion	other molecular function	Pf00441, Pf02770, Pf02771	66885
Q9JHF5	V-type proton ATPase subunit a OS=Mus musculus OX=10090 GN=Tcirg1 PE	9	5	6	5	834	93,4	6,38	cell cycle OR cell proliferation;cell organization and biogenesis;protein metabolism	plasma membrane;other membranes;nucleus;other cytoplasmic organelle;other cell component	transporter activity;other molecular function	Pf01496	27060
Q9CQE5	Regulator of G-protein signaling 10 OS=Mus musculus OX=10090 GN=Rgs10	38	6	18	6	181	21,1	6,81	cell-cell signaling;signal transduction;other biological processes	cytosol;nucleus;other cell component	enzyme regulator activity;other molecular function	Pf00615	67865
Q9ER39	Torsin-1A OS=Mus musculus OX=10090 GN=Tor1a PE=1 SV=1	10	3	4	3	333	37,8	7,17	cell adhesion;cell organization and biogenesis;protein metabolism;other metaboli	other membranes;cytoskeleton;ER/Golgi;nucleus;other cell component	cytoskeletal activity;other molecular function	Pf06309	30931
Q9DCN2	NADH-cytochrome b5 reductase 3 OS=Mus musculus OX=10090 GN=Cyb5r3	26	6	7	6	301	34,1	8,38	other metabolic processes	other membranes;mitochondrion;ER/Golgi	other molecular function	Pf00175, Pf00970	109754
Q91ZA3	Propionyl-CoA carboxylase alpha chain, mitochondrial OS=Mus musculus OX	36	22	49	22	724	79,9	7,25	other metabolic processes	mitochondrion	other molecular function	Pf00289, Pf00364, Pf02785, Pf02786, Pf1	110821
P28741	Kinesin-like protein KIF3A OS=Mus musculus OX=10090 GN=Kif3a PE=1 SV=	10	6	6	6	701	80,1	6,54	cell cycle OR cell proliferation;cell organization and biogenesis;transport;developr	cytosol;cytoskeleton;other cell component	cytoskeletal activity;other molecular function	Pf00225	16568
Q8C181	Muscleblind-like protein 2 OS=Mus musculus OX=10090 GN=Mbnl2 PE=2 SV	23	8	13	2	373	40,1	9	RNA metabolism OR transcription;other metabolic processes;other biological proc	nucleus;other cell component	nucleic acid binding activity;other molecular function	Pf00642, Pf14608	105559
Q99LZ3	DNA replication complex GINS protein SLD5 OS=Mus musculus OX=10090 GN	33	6	13	6	223	25,9	5,24	cell cycle OR cell proliferation;DNA metabolism;other metabolic processes;stress	nucleus;other cell component		Pf05916, Pf16922	109145
Q4G5Y1	Kelch domain-containing protein 2 OS=Mus musculus OX=10090 GN=Klhdc2	15	5	7	5	406	45,9	6,61	protein metabolism;other metabolic processes	other membranes;nucleus;other cell component	other molecular function	Pf13418	69554
Q8BVL3	Sorting nexin-17 OS=Mus musculus OX=10090 GN=Snx17 PE=1 SV=2	15	4	4	4	470	52,8	7,49	transport;developmental processes;signal transduction	other membranes;cytosol;ER/Golgi;other cytoplasmic organelle;other cell component	signal transduction activity or receptor binding;other molecular function	Pf00787, Pf18116	266781
P58929	Glucocorticoid modulatory element-binding protein 2 OS=Mus musculus OX=	13	5	6	5	530	56,6	5,31	other biological processes	cytosol;nucleus	nucleic acid binding activity;other molecular function	Pf01342	229004
Q6P7W5	tRNA-splicing endonuclease subunit Sen2 OS=Mus musculus OX=10090 GN=	15	6	10	6	460	52,2	5,9	RNA metabolism OR transcription;other metabolic processes	cytosol;cytoskeleton;nucleus	nucleic acid binding activity;other molecular function	Pf01974, Pf02778	381802
Q99JR8	SWI/SNF-related matrix-associated actin-dependent regulator of chromatin s	5	2	2	2	531	59	9,64	cell organization and biogenesis;other biological processes	nucleus		Pf02201	83796
E9PUK6	Serine/arginine repetitive matrix protein 1 OS=Mus musculus OX=10090 GN=	23	21	47	21	923	104,2	11,85	RNA metabolism OR transcription;other metabolic processes	nucleus		Pf01480	51796
Q62415	Apoptosis-stimulating of p53 protein 1 OS=Mus musculus OX=10090 GN=Ppp	5	5	11	4	1087	119,1	6,68	signal transduction;other biological processes	plasma membrane;other membranes;cytosol;nucleus;other cell component	other molecular function	Pf00018, Pf12796	21981
Q8K2W3	Thioredoxin domain-containing protein 11 OS=Mus musculus OX=10090 GN=	6	5	5	5	948	105,9	7,88		other membranes;ER/Golgi		Pf00085	106200
Q9D1K2	V-type proton ATPase subunit F OS=Mus musculus OX=10090 GN=Atp6v1f F	41	5	21	5	119	13,4	5,82	transport	other membranes;other cytoplasmic organelle	transporter activity	Pf01990	66144
Q9EST4	Proteasome assembly chaperone 2 OS=Mus musculus OX=10090 GN=Psmg	33	9	31	9	264	29,5	6,05	cell cycle OR cell proliferation;cell organization and biogenesis;signal transduction	cytosol;nucleus;other cell component	other molecular function	Pf09754	107047
Q9D7E4	UPF0449 protein C19orf25 homolog OS=Mus musculus OX=10090 PE=1 SV=	80	6	16	6	109	12,1	5,25				Pf15136	
P61087	Ubiquitin-conjugating enzyme E2 K OS=Mus musculus OX=10090 GN=Ube2k	58	12	34	12	200	22,4	5,44	cell organization and biogenesis;protein metabolism;other metabolic processes;s	nucleus;other cell component	other molecular function	Pf00179, Pf00627	53323
P28656	Nucleosome assembly protein 1-like 1 OS=Mus musculus OX=10090 GN=Na	49	15	91	14	391	45,3	4,46	cell organization and biogenesis;developmental processes;other biological proces	nucleus;other cell component	other molecular function	Pf00956	53605
O08760	N-glycosylase/DNA lyase OS=Mus musculus OX=10090 GN=Ogg1 PE=1 SV=	8	3	3	3	345	38,9	8,85	DNA metabolism;other metabolic processes;stress response;other biological proc	mitochondrion;nucleus;other cell component	cytoskeletal activity;nucleic acid binding activity;other molecular function	Pf00730, Pf07934	18294
Q9Z1P6	NADH dehydrogenase [ubiquinone] 1 alpha subcomplex subunit 7 OS=Mus m	53	6	9	6	113	12,6	10,17	protein metabolism;other metabolic processes	other membranes;mitochondrion;translational apparatus	other molecular function	Pf07347	66416
Q8K1T1	Leucine-rich repeat-containing protein 25 OS=Mus musculus OX=10090 GN=	23	4	4	4	297	32,7	8,24		other membranes;other cell component			211228
Q6WKZ8	E3 ubiquitin-protein ligase UBR2 OS=Mus musculus OX=10090 GN=Ubr2 PE=	11	17	22	17	1755	199	6,33	cell cycle OR cell proliferation;cell organization and biogenesis;protein metabolism	nucleus;other cell component	other molecular function	Pf02207, Pf02617, Pf18995	224826
Q8R179	Kelch repeat and BTB domain-containing protein 4 OS=Mus musculus OX=10	17	8	14	8	534	59,8	5,74				Pf00651, Pf07707, Pf13964	67136
A2AR02	Peptidyl-prolyl cis-trans isomerase G OS=Mus musculus OX=10090 GN=Ppig	8	7	13	6	752	88,3	10,27	protein metabolism;other metabolic processes;other biological processes	cytosol;nucleus;other cell component	other molecular function	Pf00160	228005
A0A3B2WBC6	DNA-directed RNA polymerase II subunit GRINL1A OS=Mus musculus OX=10	65	3	16	3	84	8,6	10,48					
Q3UCV8	Ubiquitin thioesterase otulin OS=Mus musculus OX=10090 GN=Otulin PE=1 S	34	11	15	11	352	40,3	5,68	cell-cell signaling;protein metabolism;other metabolic processes;stress response;	other cell component	other molecular function	Pf16218	432940
P49025	Citron Rho-interacting kinase OS=Mus musculus OX=10090 GN=Cit PE=1 SV=	4	6	7	6	2055	235,2	6,54	cell cycle OR cell proliferation;cell organization and biogenesis;protein metabolism	plasma membrane;other membranes;cytosol;cytoskeleton;ER/Golgi;nucleus;other cytoplasmic or	enzyme regulator activity;kinase activity;other molecular function	Pf00069, Pf00169, Pf00780	12704
Q9ES97	Reticulon-3 OS=Mus musculus OX=10090 GN=Rtn3 PE=1 SV=2	6	3	7	3	964	103,8	4,92	cell organization and biogenesis;transport;other biological processes	other membranes;ER/Golgi;other cell component		Pf02453	20168
F6UK66	Coiled-coil domain-containing protein 50 (Fragment) OS=Mus musculus OX=	42	12	26	12	256	29,8	6,67				Pf15295	
Q9D0G0	28S ribosomal protein S30, mitochondrial OS=Mus musculus OX=10090 GN=	33	12	21	12	442	49,9	9,38	protein metabolism;other metabolic processes	mitochondrion;translational apparatus	other molecular function	Pf07147	59054
Q8BRM2	RAB6-interacting golgin OS=Mus musculus OX=10090 GN=Gorab PE=1 SV=1	23	7	9	7	368	41,5	7,36	cell organization and biogenesis;developmental processes;other biological proces	cytosol;ER/Golgi;nucleus;other cell component		Pf04949	98376
Q91WQ5	TAF5-like RNA polymerase II p300/CBP-associated factor-associated factor 6	6	2	2	2	589	65,9	5,92	protein metabolism;other metabolic processes;other biological processes	nucleus;other cell component	other molecular function	Pf00400, Pf04494	102162
P34022	Ran-specific GTPase-activating protein OS=Mus musculus OX=10090 GN=Ra	62	9	78	8	203	23,6	5,22	cell cycle OR cell proliferation;cell organization and biogenesis;transport;other bio	cytosol;cytoskeleton;nucleus;other cell component	enzyme regulator activity	Pf00638	19385
Q91VC3	Eukaryotic initiation factor 4A-III OS=Mus musculus OX=10090 GN=Eif4a3 PE	37	15	39	2	411	46,8	6,73	RNA metabolism OR transcription;other metabolic processes;transport;developm	cytosol;nucleus;other cell component	nucleic acid binding activity;other molecular function	Pf00270, Pf00271	192170
Q8C1A3	Methionine synthase reductase OS=Mus musculus OX=10090 GN=Mtrr PE=	13	7	9	7	696	77,5	6,38	DNA metabolism;other metabolic processes;other biological processes	cytosol;cytoskeleton;nucleus	other molecular function	Pf00175, Pf00258, Pf00667	210009
Q9D8B3	Charged multivesicular body protein 4b OS=Mus musculus OX=10090 GN=Ch	53	16	81	16	224	24,9	4,82	cell cycle OR cell proliferation;cell organization and biogenesis;protein metabolism	plasma membrane;other membranes;cytosol;nucleus;other cytoplasmic organelle;other cell comp	other molecular function	Pf03357	75608
Q99JT1	Glutamyl-tRNA(Gln) amidotransferase subunit B, mitochondrial OS=Mus mus	12	5	6	5	557	62,1	8,54	protein metabolism;RNA metabolism OR transcription;other metabolic processes	mitochondrion;other cell component	other molecular function	Pf02637, Pf02934	229487
Q8VD62	UPF0696 protein C11orf68 homolog OS=Mus musculus OX=10090 GN=Bles0	27	7	11	7	298	31,8	6,1				Pf08939	107242
Q8BTR5	Dual specificity phosphatase 28 OS=Mus musculus OX=10090 GN=Dusp28 P	12	2	2	2	163	17,5	7,75	protein metabolism;other metabolic processes		other molecular function	Pf00782	67446
Q9JKY0	CCR4-NOT transcription complex subunit 9 OS=Mus musculus OX=10090 GN=	22	5	10	5	299	33,6	8,03	RNA metabolism OR transcription;other metabolic processes;signal transduction;	nucleus;other cell component	signal transduction activity or receptor binding;other molecular function	Pf04078	58184
Q9Z0G0	PDZ domain-containing protein GIPC1 OS=Mus musculus OX=10090 GN=Gip	29	8	14	8	333	36,1	5,91	cell-cell signaling;transport;signal transduction;other biological processes	other membranes;cytosol;ER/Golgi;other cell component	signal transduction activity or receptor binding;enzyme regulator activity;cytosl	Pf00595	67903
Q8BG94	COMM domain-containing protein 7 OS=Mus musculus OX=10090 GN=Com	33	6	13	6	200	22,6	5,94	signal transduction;other biological processes	other cell component	other molecular function	Pf07258	99311
Q8K296	Myotubularin-related protein 3 OS=Mus musculus OX=10090 GN=Mtmr3 PE	17	13	17	12	1196	133,8	5,83	protein metabolism;other metabolic processes;stress response;other biological pr	other membranes;other cell component	other molecular function	Pf01363, Pf06602	74302
P35385	Heat shock protein beta-7 OS=Mus musculus OX=10090 GN=Hspb7 PE=1 SV	29	4	6	4	169	18,6	6,42	stress response;developmental processes	cytoskeleton;nucleus;other cell component	cytoskeletal activity	Pf00011	29818
Q00941	Granulocyte-macrophage colony-stimulating factor receptor subunit alpha O	5	2	2	2	388	41,7	7,78	signal transduction	plasma membrane;other membranes;other cell component	other molecular function	Pf09240, Pf18611	12982
Q5UE59	Kinesin light chain OS=Mus musculus OX=10090 GN=Klc1 PE=1 SV=1	54	27	61	20	542	61,6	5,76		cytoskeleton;other cell component		Pf13374, Pf13424	16593
P49615	Cyclin-dependent-like kinase 5 OS=Mus musculus OX=10090 GN=Cdk5 PE=1	22	6	10	5	292	33,3	7,66	cell adhesion;cell-cell signaling;cell cycle OR cell proliferation;cell organization anc	plasma membrane;other membranes;cytosol;cytoskeleton;nucleus;other cell component	signal transduction activity or receptor binding;transporter activity;cytoskeletal	Pf00069	12568
P56390	Cyclin-dependent kinases regulatory subunit 2 OS=Mus musculus OX=10090	33	3	5	2	79	9,9	8,46	cell cycle OR cell proliferation;cell organization and biogenesis;other biological pro	other cell component	enzyme regulator activity;other molecular function	Pf01111	66197
A0A0G2JGD2	Protein S100-A4 (Fragment) OS=Mus musculus OX=10090 GN=S100a4 PE=1	71	7	101	7	78	9,1	5,21		nucleus;other cell component	other molecular function	Pf01023	
Q9DCB8	Iron-sulfur cluster assembly 2 homolog, mitochondrial OS=Mus musculus OX	77	9	24	9	154	16,7	5,6	cell organization and biogenesis;protein metabolism;other metabolic processes		other molecular function	Pf01521	74316
A0A0A6YXW4	39S ribosomal protein L30, mitochondrial (Fragment) OS=Mus musculus OX=	23	3	4	3	128	14,8	10,65	protein metabolism;other metabolic processes	translational apparatus	other molecular function	Pf00327	
A0A087WQK5	alpha-1,2-Mannosidase OS=Mus musculus OX=10090 GN=Edem3 PE=1 SV=	2	2	3	2	917	102,5	5,01	protein metabolism;other metabolic processes;stress response	other membranes;ER/Golgi	other molecular function	Pf01532, Pf02225	66967
G8JL51	DNA-directed RNA polymerase I subunit RPA49 (Fragment) OS=Mus musculu	5	2	2	2	407	45,9	9,16	RNA metabolism OR transcription;other metabolic processes	nucleus	nucleic acid binding activity;other molecular function	Pf06870	
Q8R5C5	Beta-centractin OS=Mus musculus OX=10090 GN=Actr1b PE=1 SV=1	49	13	52	5	376	42,3	6,4		cytoskeleton;other cell component	other molecular function	Pf00022	226977
P10852	4F2 cell-surface antigen heavy chain OS=Mus musculus OX=10090 GN=Slc3a	23	12	19	12	526	58,3	5,91	other metabolic processes;transport;other biological processes	plasma membrane;other membranes;nucleus;other cytoplasmic organelle;other cell component	transporter activity;nucleic acid binding activity;other molecular function	Pf00128, Pf16028	17254
P22437	Prostaglandin G/H synthase 1 OS=Mus musculus OX=10090 GN=Ptgs1 PE=1	6	2	2	2	602	69	6,83	other metabolic processes;stress response;developmental processes;other biolog	other membranes;ER/Golgi;nucleus;other cell component	other molecular function	Pf03098	19224
Q8BP40	Lysophosphatidic acid phosphatase type 6 OS=Mus musculus OX=10090 GN	27	9	22	9	418	47,6	7,72	other metabolic processes;developmental processes	other cell component	other molecular function	Pf00328	66659
Q8VC85	U6 snRNA-associated Sm-like protein LSm1 OS=Mus musculus OX=10090 GN	55	6	15	6	133	15,2	5,38	RNA metabolism OR transcription;other metabolic processes;other biological proc	nucleus;other cell component	nucleic acid binding activity	Pf01423	67207
Q922M3	BTB/POZ domain-containing adapter for CUL3-mediated RhoA degradation p	35	10	16	8	315	35,7	6,34	cell organization and biogenesis;protein metabolism;other metabolic processes;d	cytosol;nucleus;other cell component	signal transduction activity or receptor binding;other molecular function	Pf02214	330171
Q6NVE8	WD repeat-containing protein 44 OS=Mus musculus OX=10090 GN=Wdr44 P	28	19	35	19	915	101,5	5,24	other biological processes	other membranes;cytosol;cytoskeleton;ER/Golgi;other cytoplasmic organelle;other cell componen	other molecular function	Pf00400	72404
P08752	Guanine nucleotide-binding protein G(i) subunit alpha-2 OS=Mus musculus O	28	8	16	5	355	40,5	5,45	cell-cell signaling;cell cycle OR cell proliferation;signal transduction;other biologica	plasma membrane;other membranes;cytosol;cytoskeleton;nucleus;other cell component	signal transduction activity or receptor binding;other molecular function	Pf00503	14678
Q9CZB3	THUMP domain-containing protein 2 OS=Mus musculus OX=10090 GN=Thur	15	7	10	7	528	57,6	7,06	RNA metabolism OR transcription;other metabolic processes		nucleic acid binding activity;other molecular function	Pf01170, Pf02926	72167
O70591	Prefoldin subunit 2 OS=Mus musculus OX=10090 GN=Pfdn2 PE=1 SV=2	58	8	38	8	154	16,5	6,58	other biological processes	cytosol;mitochondrion;nucleus;other cell component	other molecular function	Pf01920	18637
P63038	60 kDa heat shock protein, mitochondrial OS=Mus musculus OX=10090 GN=	74	34	790	34	573	60,9	6,18	cell cycle OR cell proliferation;cell organization and biogenesis;DNA metabolism;c	non-structural extracellular;plasma membrane;other membranes;cytosol;cytoskeleton;mitochondr	other molecular function	Pf00118	15510
P47915	60S ribosomal protein L29 OS=Mus musculus OX=10090 GN=Rpl29 PE=1 SV=	26	5	43	5	160	17,6	11,84	cell adhesion;cell cycle OR cell proliferation;protein metabolism;other metabolic p	other membranes;cytosol;translational apparatus;other cell component	other molecular function	Pf01779	19944
A0A0R4J0G0	Phosphoenolpyruvate carboxykinase (GTP) OS=Mus musculus OX=10090 GN	46	24	77	24	667	73,4	7,74	other metabolic processes;other biological processes	mitochondrion	other molecular function	Pf00821, Pf17297	74551
O35286	Pre-mRNA-splicing factor ATP-dependent RNA helicase DHX15 OS=Mus mus	45	34	177	34	795	90,9	7,46	RNA metabolism OR transcription;other metabolic processes;other biological proc	nucleus;other cell component	nucleic acid binding activity;other molecular function	Pf00270, Pf00271, Pf04408, Pf07717	13204
Q8QZT1	Acetyl-CoA acetyltransferase, mitochondrial OS=Mus musculus OX=10090 G	51	17	85	17	424	44,8	8,51	other metabolic processes;stress response;developmental processes;other biolog	mitochondrion	other molecular function	Pf00108, Pf02803	110446
Q91YL2	E3 ubiquitin-protein ligase RNF126 OS=Mus musculus OX=10090 GN=Rnf126	32	5	7	5	313	34,1	5,17	protein metabolism;other metabolic processes;stress response;transport;other bi	cytosol;nucleus;other cell component	signal transduction activity or receptor binding;other molecular function	Pf13639, Pf14369	70294
Q924T2	28S ribosomal protein S2, mitochondrial OS=Mus musculus OX=10090 GN=M	37	8	15	8	291	32,3	9,14	cell organization and biogenesis;protein metabolism;other metabolic processes	mitochondrion;translational apparatus	other molecular function	Pf00318	118451
Q8VDW0	ATP-dependent RNA helicase DDX39A OS=Mus musculus OX=10090 GN=Dd	60	21	80	11	427	49	5,68	RNA metabolism OR transcription;other metabolic processes;transport	nucleus;other cell component	nucleic acid binding activity;other molecular function	Pf00270, Pf00271	68278
E9Q446	E3 ubiquitin-protein ligase RNF25 OS=Mus musculus OX=10090 GN=Rnf25 P	16	7	8	7	455	51,1	6,19	protein metabolism;other metabolic processes		other molecular function	Pf05773	57751
Q8BJF9	Charged multivesicular body protein 2b OS=Mus musculus OX=10090 GN=Ch	29	7	15	7	213	23,9	8,78	cell cycle OR cell proliferation;cell organization and biogenesis;transport;other bio	plasma membrane;other membranes;cytosol;other cytoplasmic organelle;other cell component	other molecular function	Pf03357	68942
P68510	14-3-3 protein eta OS=Mus musculus OX=10090 GN=Ywhah PE=1 SV=2	71	18	275	12	246	28,2	4,89	cell organization and biogenesis;other metabolic processes;transport;developme	plasma membrane;other membranes;cytosol;other cell component	cytoskeletal activity;other molecular function	Pf00244	22629
P70362	Ubiquitin recognition factor in ER-associated degradation protein 1 OS=Mus r	43	12	24	11	307	34,5	6,7	protein metabolism;other metabolic processes;stress response;transport;other bi	other membranes;cytosol;ER/Golgi;nucleus;other cell component	signal transduction activity or receptor binding;other molecular function	Pf03152	22230
Q8CBY8	Dynactin subunit 4 OS=Mus musculus OX=10090 GN=Dctn4 PE=1 SV=1	44	15	31	15	467	53	7,72		cytoskeleton;other cell component	other molecular function	Pf05502	67665
Q8VBT6	Apolipoprotein B receptor OS=Mus musculus OX=10090 GN=Apobr PE=1 SV=	49	33	56	33	942	102,6	4,36	other metabolic processes;transport	non-structural extracellular;plasma membrane;other membranes	other molecular function		171504
A6PWX8	Ubiquinol-cytochrome-c reductase complex assembly factor 1 (Fragment) OS	17	3	4	3	262	30,3	8,22				Pf03981	
Q8R1R3	StAR-related lipid transfer protein 7, mitochondrial OS=Mus musculus OX=10	10	4	6	4	373	43,1	8,95	stress response;transport;developmental processes;other biological processes	non-structural extracellular;mitochondrion;other cell component	other molecular function	Pf01852	99138
Q99JF5	Diphosphomevalonate decarboxylase OS=Mus musculus OX=10090 GN=Mv	53	17	49	17	401	44	6,3	other metabolic processes;other biological processes	cytosol;other cytoplasmic organelle	other molecular function	Pf00288, Pf18376	192156
Q9D7H3	RNA 3'-terminal phosphate cyclase OS=Mus musculus OX=10090 GN=RtcA P	41	14	27	14	366	39,2	7,9	RNA metabolism OR transcription;other metabolic processes	nucleus	other molecular function	Pf01137, Pf05189	66368
O35685	Nuclear migration protein nudC OS=Mus musculus OX=10090 GN=Nudc PE=1	67	25	316	25	332	38,3	5,26	cell cycle OR cell proliferation;cell organization and biogenesis;transport;other bio	cytosol;cytoskeleton;ER/Golgi;nucleus;other cell component	other molecular function	Pf04969, Pf14050, Pf16273	18221
Q3UYU2	tRNA (uracil-5-)-methyltransferase homolog A OS=Mus musculus OX=10090	43	18	29	18	613	67,4	7,18	RNA metabolism OR transcription;other metabolic processes		nucleic acid binding activity;other molecular function	Pf05958	15547
Q3UM45	Protein phosphatase 1 regulatory subunit 7 OS=Mus musculus OX=10090 GN	61	22	97	22	361	41,3	4,92	other biological processes	nucleus;other cell component		Pf12799, Pf14580	66385
Q9CRB2	H/ACA ribonucleoprotein complex subunit 2 OS=Mus musculus OX=10090 GN	36	4	12	4	153	17,2	8,41	cell organization and biogenesis;DNA metabolism;RNA metabolism OR transcripti	nucleus;other cell component	nucleic acid binding activity	Pf01248	52530
Q80UW8	DNA-directed RNA polymerases I, II, and III subunit RPABC1 OS=Mus muscu	40	8	15	8	210	24,6	5,95	RNA metabolism OR transcription;other metabolic processes	nucleus	nucleic acid binding activity;other molecular function	Pf01191, Pf03871	66420
Q9WVE8	Protein kinase C and casein kinase substrate in neurons protein 2 OS=Mus m	47	20	47	20	486	55,8	5,2	cell organization and biogenesis;transport;signal transduction;other biological pro	plasma membrane;other membranes;cytosol;cytoskeleton;nucleus;other cytoplasmic organelle;o	cytoskeletal activity;other molecular function	Pf00018, Pf00611	23970
Q9CPR5	39S ribosomal protein L15, mitochondrial OS=Mus musculus OX=10090 GN=	35	9	12	9	295	33,5	10,07	cell organization and biogenesis;protein metabolism;other metabolic processes;o	mitochondrion;translational apparatus	other molecular function	Pf00828	27395
Q923E4	NAD-dependent protein deacetylase sirtuin-1 OS=Mus musculus OX=10090	19	9	13	9	737	80,3	4,73	cell organization and biogenesis;protein metabolism;DNA metabolism;other meta	other membranes;cytosol;mitochondrion;nucleus;other cell component	nucleic acid binding activity;other molecular function	Pf02146	93759
Q8C1B7	Septin-11 OS=Mus musculus OX=10090 GN=Septin11 PE=1 SV=4	48	18	127	9	431	49,7	6,68	cell cycle OR cell proliferation;other biological processes	cytoskeleton;other cell component	other molecular function	Pf00735	52398
P09242	Alkaline phosphatase, tissue-nonspecific isozyme OS=Mus musculus OX=10	9	5	6	5	524	57,5	7,01	other metabolic processes;developmental processes;other biological processes	non-structural extracellular;extracellular matrix;plasma membrane;other membranes	other molecular function	Pf00245	11647
Q9CQL5	39S ribosomal protein L18, mitochondrial OS=Mus musculus OX=10090 GN=	17	3	8	3	180	20,7	9,28	protein metabolism;other metabolic processes;transport	mitochondrion;translational apparatus	nucleic acid binding activity;other molecular function	Pf00861	67681
P60122	RuvB-like 1 OS=Mus musculus OX=10090 GN=Ruvbl1 PE=1 SV=1	56	20	121	20	456	50,2	6,42	cell cycle OR cell proliferation;cell organization and biogenesis;protein metabolism	cytosol;nucleus;other cell component	other molecular function	Pf06068, Pf17856	56505
Q61249	Immunoglobulin-binding protein 1 OS=Mus musculus OX=10090 GN=Igbp1 F	37	11	27	11	340	38,9	6,18	other biological processes	cytosol;cytoskeleton;other cell component	enzyme regulator activity;other molecular function	Pf04177	18518
E0CYH7	Filamin A-interacting protein 1-like OS=Mus musculus OX=10090 GN=Filip1l	12	12	13	12	1131	129,8	6,33				Pf09727	78749
Q6ZPR1	Cilia- and flagella-associated protein 97 OS=Mus musculus OX=10090 GN=Cf	13	6	7	6	541	60,8	9,51				Pf13879	66756
Q9CPP6	NADH dehydrogenase [ubiquinone] 1 alpha subcomplex subunit 5 OS=Mus n	33	4	9	4	116	13,4	8,1	cell organization and biogenesis;other metabolic processes;transport	other membranes;mitochondrion;other cell component	transporter activity	Pf04716	68202
G3UVU6	Mediator of RNA polymerase II transcription subunit 20 OS=Mus musculus O	33	5	5	5	152	16,5	8	other biological processes	nucleus	other molecular function	Pf08612	
E9Q3L2	Phosphatidylinositol 4-kinase alpha OS=Mus musculus OX=10090 GN=Pi4ka	3	5	6	5	2105	236,9	7,06	cell organization and biogenesis;other metabolic processes;signal transduction;ot	plasma membrane;other membranes;ER/Golgi;other cell component	kinase activity;other molecular function	Pf00454, Pf00613, Pf19274	224020
Q3US17	Zinc finger protein 48 OS=Mus musculus OX=10090 GN=Znf48 PE=1 SV=2	5	3	4	3	591	64,6	9,41	other biological processes	nucleus	nucleic acid binding activity;other molecular function	Pf00096	233887
Q9JJ94	Sjoegren syndrome nuclear autoantigen 1 homolog OS=Mus musculus OX=1	71	10	22	10	119	13,5	5,68	cell organization and biogenesis;transport;signal transduction	cytoskeleton;nucleus;other cell component	other molecular function		68475
O35887	Calumenin OS=Mus musculus OX=10090 GN=Calu PE=1 SV=1	47	13	83	3	315	37	4,67	other biological processes	non-structural extracellular;other membranes;cytosol;ER/Golgi;other cell component	enzyme regulator activity;other molecular function	Pf13202, Pf13833	12321
G3X8R1	KICSTOR complex protein kaptin OS=Mus musculus OX=10090 GN=Kptn PE=	14	5	5	5	430	47,5	5	cell organization and biogenesis;stress response;other biological processes	cytoskeleton;other cell component	cytoskeletal activity		70394
Q80VP0	Tectonin beta-propeller repeat-containing protein 1 OS=Mus musculus OX=1	7	7	7	7	1166	130,2	6,48	cell organization and biogenesis;other metabolic processes	other membranes;nucleus;other cytoplasmic organelle;other cell component	other molecular function	Pf06398, Pf19193	70381
E9Q7M2	TSC22 domain family, member 2 OS=Mus musculus OX=10090 GN=Tsc22d2	10	8	18	5	769	78,1	4,89	stress response;other biological processes			Pf01166	72033
Q9D3W4	GPN-loop GTPase 3 OS=Mus musculus OX=10090 GN=Gpn3 PE=2 SV=1	19	3	4	3	284	32,8	4,53		other cell component	other molecular function	Pf03029	68080
Q8BIJ6	Isoleucine--tRNA ligase, mitochondrial OS=Mus musculus OX=10090 GN=Iar	38	33	119	33	1012	112,7	6,81	protein metabolism;RNA metabolism OR transcription;other metabolic processes	mitochondrion	nucleic acid binding activity;other molecular function	Pf00133, Pf08264	381314
Q68FH4	N-acetylgalactosamine kinase OS=Mus musculus OX=10090 GN=Galk2 PE=1	38	13	52	13	458	50,5	6,9	other metabolic processes	cytosol	kinase activity;other molecular function	Pf00288, Pf08544, Pf10509	69976
Q8BLH7	HIRA-interacting protein 3 OS=Mus musculus OX=10090 GN=Hirip3 PE=1 SV=	57	29	67	29	601	65,2	7,84		nucleus		Pf09649	233876
Q497H0	AN1-type zinc finger protein 3 OS=Mus musculus OX=10090 GN=Zfand3 PE=	35	7	10	7	227	25,1	7,15			nucleic acid binding activity;other molecular function	Pf01428, Pf01754	21769
Q9CQ37	Ubiquitin-conjugating enzyme E2 T OS=Mus musculus OX=10090 GN=Ube2t	36	6	9	5	204	23	7,97	protein metabolism;DNA metabolism;other metabolic processes;stress response	nucleus	other molecular function	Pf00179	67196
A0A087WQI6	Glutathione S-transferase (Fragment) OS=Mus musculus OX=10090 GN=Gst	15	2	2	2	133	15,4	5,55	other metabolic processes		other molecular function	Pf00043, Pf02798	
Q91ZW3	SWI/SNF-related matrix-associated actin-dependent regulator of chromatin s	21	23	33	23	1051	121,6	8,15	cell organization and biogenesis;RNA metabolism OR transcription;other metaboli	cytosol;nucleus;other cell component	nucleic acid binding activity;other molecular function	Pf00176, Pf00271, Pf09110, Pf09111	93762
Q8BFY6	Peflin OS=Mus musculus OX=10090 GN=Pef1 PE=1 SV=1	16	4	10	4	275	29,2	6,3	cell organization and biogenesis;protein metabolism;other metabolic processes;tı	other membranes;ER/Golgi;other cell component	other molecular function	Pf13405, Pf13499	67898
Q71FD5	E3 ubiquitin-protein ligase ZNRF2 OS=Mus musculus OX=10090 GN=Znrf2 PE	45	6	6	6	238	23,7	6,76	protein metabolism;other metabolic processes	plasma membrane;other membranes;nucleus;other cytoplasmic organelle;other cell component	other molecular function	Pf13639	387524
A0A0R4J2B0	CUGBP Elav-like family member 2 OS=Mus musculus OX=10090 GN=Celf2 PE	22	9	15	7	440	46,6	8,98		other cell component	nucleic acid binding activity	Pf00076	14007
Q9D975	Sulfiredoxin-1 OS=Mus musculus OX=10090 GN=Srxn1 PE=2 SV=1	26	3	4	3	136	14,1	8,02	stress response;other biological processes	cytosol;other cell component	other molecular function	Pf02195	76650
Q8CG72	ADP-ribose glycohydrolase ARH3 OS=Mus musculus OX=10090 GN=Adprs P	39	12	58	12	370	39,4	4,96	protein metabolism;DNA metabolism;other metabolic processes;stress response	mitochondrion;nucleus;other cell component	other molecular function	Pf03747	100206
Q8K2M0	39S ribosomal protein L38, mitochondrial OS=Mus musculus OX=10090 GN=	38	11	17	11	380	45	8,1		mitochondrion;translational apparatus		Pf01161	60441
Q60932	Voltage-dependent anion-selective channel protein 1 OS=Mus musculus OX=	10	3	4	3	296	32,3	8,43	cell-cell signaling;stress response;transport;other biological processes	plasma membrane;other membranes;mitochondrion;ER/Golgi;other cell component	transporter activity;other molecular function	Pf01459	22333
Q5SUR0	Phosphoribosylformylglycinamidine synthase OS=Mus musculus OX=10090	48	44	205	44	1337	144,5	5,67	other metabolic processes;other biological processes	other cell component	other molecular function	Pf02769, Pf13507, Pf18072, Pf18076	237823
Q3TYD4	Arylsulfatase G OS=Mus musculus OX=10090 GN=Arsg PE=1 SV=1	8	3	7	3	525	57,4	6,39	other metabolic processes	non-structural extracellular;ER/Golgi;other cytoplasmic organelle	other molecular function	Pf00884, Pf14707	74008
Q8BJ05	Zinc finger CCCH domain-containing protein 14 OS=Mus musculus OX=10090	17	10	13	10	735	82,4	7,37	other biological processes	nucleus;other cell component	nucleic acid binding activity;other molecular function	Pf14608	75553
Q9ERS2	NADH dehydrogenase [ubiquinone] 1 alpha subcomplex subunit 13 OS=Mus	24	3	3	3	144	16,8	9,48	cell organization and biogenesis;other metabolic processes;transport;signal trans	other membranes;mitochondrion;nucleus;other cell component	transporter activity;other molecular function	Pf06212	67184
P97770	THUMP domain-containing protein 3 OS=Mus musculus OX=10090 GN=Thur	25	14	29	14	505	56,4	7,43	RNA metabolism OR transcription;other metabolic processes	cytosol;nucleus	nucleic acid binding activity;other molecular function	Pf01170, Pf02926	14911
Q9CYI4	Putative RNA-binding protein Luc7-like 1 OS=Mus musculus OX=10090 GN=L	43	16	36	9	371	43,9	9,88	cell organization and biogenesis;RNA metabolism OR transcription;other metaboli	nucleus	nucleic acid binding activity;other molecular function	Pf03194	66978
Q8BYW1	Rho GTPase-activating protein 25 OS=Mus musculus OX=10090 GN=Arhgap	14	10	13	8	648	73,3	6,38	cell organization and biogenesis;transport;signal transduction;other biological pro	plasma membrane	enzyme regulator activity	Pf00169, Pf00620	232201
A6PWS5	Macrophage-capping protein (Fragment) OS=Mus musculus OX=10090 GN=	21	6	35	2	251	28	7,02	cell organization and biogenesis;other biological processes	nucleus;other cell component	cytoskeletal activity	Pf00626	
Q5NCF2	Trafficking protein particle complex subunit 1 OS=Mus musculus OX=10090	32	4	5	4	145	16,9	9,16	transport	ER/Golgi;other cell component		Pf04099	245828
A0A1B0GSU0	Aldehyde dehydrogenase family 16 member A1 OS=Mus musculus OX=100	30	18	56	18	802	84,7	6,32			other molecular function	Pf00171	69748
Q3TLS3	GDP-D-glucose phosphorylase 1 OS=Mus musculus OX=10090 GN=Gdpgp1 F	19	6	9	6	386	42,5	6,19	other metabolic processes	other cell component	enzyme regulator activity;other molecular function		269952
Q07813	Apoptosis regulator BAX OS=Mus musculus OX=10090 GN=Bax PE=1 SV=1	48	6	27	6	192	21,4	4,98	cell cycle OR cell proliferation;cell organization and biogenesis;other metabolic pro	other membranes;cytosol;mitochondrion;ER/Golgi;nucleus;other cell component	transporter activity;other molecular function	Pf00452	12028
Q3TGS9	ADP-ribosylation factor GTPase-activating protein 1 OS=Mus musculus OX=1	14	4	6	4	394	43,2	5,54			enzyme regulator activity	Pf01412	228998
Q9Z0N1	Eukaryotic translation initiation factor 2 subunit 3, X-linked OS=Mus musculu	60	22	110	22	472	51	8,4	cell organization and biogenesis;protein metabolism;other metabolic processes;o	cytosol;other cell component	nucleic acid binding activity;other molecular function	Pf00009, Pf03144, Pf09173	26905
Q8R3C0	Mini-chromosome maintenance complex-binding protein OS=Mus musculus	30	16	26	16	642	72,8	5,66	cell cycle OR cell proliferation;cell organization and biogenesis;other metabolic pro	cytosol;nucleus;other cell component	nucleic acid binding activity;other molecular function	Pf09739	210711
Q9Z2M7	Phosphomannomutase 2 OS=Mus musculus OX=10090 GN=Pmm2 PE=1 SV=	60	16	81	13	242	27,6	6,42	protein metabolism;other metabolic processes	cytosol;nucleus;other cell component	other molecular function	Pf03332	54128
Q5SSZ5	Tensin-3 OS=Mus musculus OX=10090 GN=Tns3 PE=1 SV=1	7	7	7	6	1440	155,5	6,65	developmental processes;other biological processes	cytosol;other cell component		Pf00017, Pf08416, Pf10409	319939
Q9QZB7	Actin-related protein 10 OS=Mus musculus OX=10090 GN=Actr10 PE=1 SV=2	38	12	27	12	417	46,2	7,61	transport;other biological processes	cytoskeleton;other cell component		Pf00022	56444
Q3TNA1	Xylulose kinase OS=Mus musculus OX=10090 GN=Xylb PE=1 SV=1	26	11	13	11	551	59,5	6,9	other metabolic processes	cytosol	kinase activity;other molecular function	Pf00370, Pf02782	102448
Q9JHI7	Exosome complex component RRP45 OS=Mus musculus OX=10090 GN=Exos	35	12	44	12	438	48,9	5,11	RNA metabolism OR transcription;other metabolic processes;other biological proc	nucleus;other cytoplasmic organelle;other cell component	nucleic acid binding activity;other molecular function	Pf01138, Pf03725	50911
Q9ESX5	H/ACA ribonucleoprotein complex subunit DKC1 OS=Mus musculus OX=1009	38	20	42	20	509	57,4	9,28	cell organization and biogenesis;DNA metabolism;RNA metabolism OR transcripti	nucleus	nucleic acid binding activity;other molecular function	Pf01472, Pf01509, Pf08068, Pf16198	245474
F8WI22	Bcl-2-associated transcription factor 1 OS=Mus musculus OX=10090 GN=Bcla	34	27	63	27	870	100,2	9,96	other biological processes	nucleus		Pf15440	72567
A2AUE1	DnaJ homolog subfamily C member 5 (Fragment) OS=Mus musculus OX=100	17	2	3	2	141	15,8	7,85				Pf00226	
Q99J36	THUMP domain-containing protein 1 OS=Mus musculus OX=10090 GN=Thur	53	18	54	18	350	38,9	6,07	RNA metabolism OR transcription;other metabolic processes		nucleic acid binding activity	Pf02926	233802
Q9D8F1	Alpha-ketoglutarate-dependent dioxygenase alkB homolog 4 OS=Mus musc	14	3	4	3	300	33,4	7,01	cell cycle OR cell proliferation;cell organization and biogenesis;protein metabolism	nucleus;other cell component	cytoskeletal activity;other molecular function		72041
P97823	Acyl-protein thioesterase 1 OS=Mus musculus OX=10090 GN=Lypla1 PE=1 S	25	5	15	5	230	24,7	6,62	protein metabolism;other metabolic processes;other biological processes	plasma membrane;other membranes;cytosol;ER/Golgi;nucleus;other cell component	other molecular function	Pf02230	18777
P0C7N9	Proteasome assembly chaperone 4 OS=Mus musculus OX=10090 GN=Psmg	59	7	21	7	123	14	6,15	cell organization and biogenesis	other cell component	other molecular function	Pf16093	69666
Q9Z1S0	Mitotic checkpoint serine/threonine-protein kinase BUB1 beta OS=Mus musc	9	7	7	7	1052	118,3	5,43	cell cycle OR cell proliferation;cell organization and biogenesis;protein metabolism	cytosol;cytoskeleton;nucleus;other cell component	kinase activity;other molecular function	Pf08311	12236
Q80XI4	Phosphatidylinositol 5-phosphate 4-kinase type-2 beta OS=Mus musculus O	29	11	22	6	416	47,3	7,33	cell organization and biogenesis;other metabolic processes;transport;other biolog	plasma membrane;other membranes;ER/Golgi;nucleus;other cytoplasmic organelle	kinase activity;other molecular function	Pf01504	108083
Q6NZB0	DnaJ homolog subfamily C member 8 OS=Mus musculus OX=10090 GN=Dna	53	16	62	16	253	29,8	9,06		non-structural extracellular;cytosol;nucleus	other molecular function	Pf00226	68598
P21550	Beta-enolase OS=Mus musculus OX=10090 GN=Eno3 PE=1 SV=3	57	22	510	13	434	47	7,18	other metabolic processes;other biological processes	plasma membrane;other membranes;cytosol	other molecular function	Pf00113, Pf03952	13808
Q8K157	Galactose mutarotase OS=Mus musculus OX=10090 GN=Galm PE=1 SV=1	25	7	20	7	342	37,8	6,74	other metabolic processes	other cell component	other molecular function	Pf01263	319625
P62869	Elongin-B OS=Mus musculus OX=10090 GN=Elob PE=1 SV=1	95	10	47	10	118	13,2	5,01	protein metabolism;RNA metabolism OR transcription;other metabolic processes;	cytosol;nucleus;other cell component	other molecular function	Pf00240	67673
Q9CQ79	Thioredoxin domain-containing protein 9 OS=Mus musculus OX=10090 GN=T	31	8	15	8	226	26,2	5,95		cytosol;cytoskeleton;nucleus;other cell component		Pf00085	98258
E9Q624	Zinc finger protein 644 OS=Mus musculus OX=10090 GN=Zfp644 PE=1 SV=1	2	2	3	2	1292	144,9	8,27	other biological processes	nucleus	nucleic acid binding activity;other molecular function		52397
Q8BTM8	Filamin-A OS=Mus musculus OX=10090 GN=Flna PE=1 SV=5	57	116	385	107	2647	281	6,04	cell cycle OR cell proliferation;cell organization and biogenesis;protein metabolism	plasma membrane;other membranes;cytosol;cytoskeleton;ER/Golgi;nucleus;other cell componen	signal transduction activity or receptor binding;cytoskeletal activity;other molec	Pf00307, Pf00630	192176
P16332	Methylmalonyl-CoA mutase, mitochondrial OS=Mus musculus OX=10090 GN	34	20	45	20	748	82,8	6,89	other metabolic processes;developmental processes;other biological processes	mitochondrion;other cell component	other molecular function	Pf01642, Pf02310	17850
Q62383	Transcription elongation factor SPT6 OS=Mus musculus OX=10090 GN=Supt	27	37	56	37	1726	199	4,93	cell organization and biogenesis;RNA metabolism OR transcription;other metaboli	nucleus;other cell component	nucleic acid binding activity;other molecular function	Pf00575, Pf14632, Pf14633, Pf14635, Pf1	20926
Q99JY9	Actin-related protein 3 OS=Mus musculus OX=10090 GN=Actr3 PE=1 SV=3	56	20	231	13	418	47,3	5,88	cell cycle OR cell proliferation;cell organization and biogenesis;stress response;de	other membranes;cytoskeleton;ER/Golgi;nucleus;other cell component	cytoskeletal activity;other molecular function	Pf00022	74117
A0A0R4J0V5	DNA-directed RNA polymerase subunit OS=Mus musculus OX=10090 GN=Po	36	60	149	60	1932	213,3	7,43	RNA metabolism OR transcription;other metabolic processes	nucleus	nucleic acid binding activity;other molecular function	Pf00623, Pf04983, Pf04990, Pf04992, Pf0	
O08583	THO complex subunit 4 OS=Mus musculus OX=10090 GN=Alyref PE=1 SV=3	47	9	42	7	255	26,9	11,15	RNA metabolism OR transcription;other metabolic processes;transport	nucleus;other cell component	nucleic acid binding activity	Pf00076, Pf13865	21681
Q9D2Z4	Sentrin-specific protease 8 OS=Mus musculus OX=10090 GN=Senp8 PE=1 SV	15	3	3	3	221	25	5,95	protein metabolism;other metabolic processes	nucleus	other molecular function	Pf02902	71599
Q99NB8	Ubiquilin-4 OS=Mus musculus OX=10090 GN=Ubqln4 PE=1 SV=1	34	14	90	12	596	63,5	5,03	protein metabolism;DNA metabolism;other metabolic processes;stress response;	other membranes;cytosol;ER/Golgi;nucleus;other cytoplasmic organelle;other cell component	other molecular function	Pf00240, Pf00627	94232
O08970	Tuftelin OS=Mus musculus OX=10090 GN=Tuft1 PE=1 SV=2	8	3	3	3	390	44,5	6,14	developmental processes;signal transduction	non-structural extracellular;other cell component			22156
Q62193	Replication protein A 32 kDa subunit OS=Mus musculus OX=10090 GN=Rpa2	33	6	12	6	270	29,7	6,15	cell cycle OR cell proliferation;cell organization and biogenesis;DNA metabolism;o	nucleus;other cell component	nucleic acid binding activity;other molecular function	Pf08784	
Q9D0I6	WD repeat, SAM and U-box domain-containing protein 1 OS=Mus musculus	8	4	4	4	474	51,7	6,34	protein metabolism;other metabolic processes		other molecular function	Pf00400, Pf04564, Pf07647	72137
Q9CV28	Ubiquitin carboxyl-terminal hydrolase MINDY-3 OS=Mus musculus OX=1009	8	4	6	4	444	49,6	4,78	protein metabolism;other metabolic processes;other biological processes	other membranes;nucleus	other molecular function	Pf13898	66960
Q9CYG7	Mitochondrial import receptor subunit TOM34 OS=Mus musculus OX=10090	48	15	49	15	309	34,3	9,14	cell organization and biogenesis;transport	other membranes;cytosol;mitochondrion;other cell component	other molecular function	Pf00515, Pf13181	67145

Q61069	Upstream stimulatory factor 1 OS=Mus musculus OX=10090 GN=Usf1 PE=1 S	9	3	4	2	310	33,6	5,54	other metabolic processes;stress response;other biological processes	nucleus;other cell component	nucleic acid binding activity;other molecular function	Pf00010	22278	ENSMUSG00000026641
Q9CQQ8	U6 snRNA-associated Sm-like protein LSm7 OS=Mus musculus OX=10090 GN	48	4	13	4	103	11,6	5,27	RNA metabolism OR transcription;other metabolic processes	nucleus;other cell component	nucleic acid binding activity;other molecular function	Pf01423	66094	ENSMUSG00000035215
Q8BPB0	MOB kinase activator 1B OS=Mus musculus OX=10090 GN=Mob1b PE=1 SV	15	3	4	3	216	25,1	6,73	signal transduction;other biological processes	cytosol;nucleus;other cell component	enzyme regulator activity;other molecular function	Pf03637	68473	ENSMUSG00000006262
G3X8R8	Myocardin-related transcription factor B OS=Mus musculus OX=10090 GN=N	5	4	5	4	1091	118,5	6,09	other biological processes		other molecular function	Pf02037, Pf02755	239719	ENSMUSG00000009569
Q9D6R2	Isocitrate dehydrogenase [NAD] subunit alpha, mitochondrial OS=Mus musc	52	18	59	18	366	39,6	6,73	other metabolic processes		other molecular function	Pf00180	67834	ENSMUSG00000032279
Q9CR51	V-type proton ATPase subunit G 1 OS=Mus musculus OX=10090 GN=Atp6v1	47	4	42	4	118	13,7	7,97	transport;other biological processes	plasma membrane;other membranes;cytosol;other cytoplasmic organelle	transporter activity;other molecular function	Pf03179	66290	ENSMUSG00000039105
G3UXL2	Ribose-phosphate diphosphokinase OS=Mus musculus OX=10090 GN=Prps1	58	19	143	9	318	34,8	6,98	other metabolic processes	other cell component	kinase activity;other molecular function	Pf13793, Pf14572	328099	ENSMUSG00000079104
E9PZ24	Mirror-image polydactyly gene 1 protein homolog OS=Mus musculus OX=10	6	2	2	2	419	47,8	5,68						ENSMUSG00000047022
Q6P069	Sorcin OS=Mus musculus OX=10090 GN=Sri PE=1 SV=1	61	10	31	10	198	21,6	5,59	transport;developmental processes;other biological processes	non-structural extracellular;plasma membrane;other membranes;cytosol;cytoskeleton;mitochondr	other molecular function	Pf13405, Pf13833	109552	ENSMUSG00000003161
P63242	Eukaryotic translation initiation factor 5A-1 OS=Mus musculus OX=10090 GN	85	12	180	7	154	16,8	5,24	protein metabolism;other metabolic processes;transport;signal transduction;othe	other membranes;ER/Golgi;nucleus;other cell component	translation activity;nucleic acid binding activity;other molecular function	Pf01287	276770	ENSMUSG00000078812
Q3TKT4	Transcription activator BRG1 OS=Mus musculus OX=10090 GN=Smarca4 PE=	6	10	11	10	1613	181,3	8	cell organization and biogenesis;protein metabolism;DNA metabolism;RNA meta	nucleus;other cell component	nucleic acid binding activity;other molecular function	Pf00176, Pf00271, Pf00439, Pf07529, Pf0	20586	ENSMUSG00000032187
Q91YN5	UDP-N-acetylhexosamine pyrophosphorylase OS=Mus musculus OX=10090	40	17	31	17	522	58,6	6,49	other metabolic processes	plasma membrane;other membranes;cytosol;nucleus	other molecular function	Pf01704	107652	
Q8BMJ2	Leucine--tRNA ligase, cytoplasmic OS=Mus musculus OX=10090 GN=Lars1 P	35	36	81	36	1178	134,1	7,05	cell organization and biogenesis;protein metabolism;RNA metabolism OR transcri	cytosol;ER/Golgi;nucleus;other cytoplasmic organelle;other cell component	enzyme regulator activity;other molecular function	Pf00133, Pf08264	107045	ENSMUSG00000024493
Q3ZB59	GRB2-associated-binding protein 2 OS=Mus musculus OX=10090 GN=Gab2 P	8	4	4	4	665	73,2	8,31				Pf00169	14389	ENSMUSG00000004508
Q80ZX0	Sec24-related gene family, member B (S. cerevisiae) OS=Mus musculus OX=	14	15	18	15	1251	135,5	6,96	cell organization and biogenesis;transport;developmental processes;other biolog	other membranes;cytosol;ER/Golgi;other cell component	other molecular function	Pf00626, Pf04810, Pf04811, Pf04815, Pf0	99683	ENSMUSG00000001052
O88952	Protein lin-7 homolog C OS=Mus musculus OX=10090 GN=Lin7c PE=1 SV=2	62	10	13	10	197	21,8	8,43	cell-cell signaling;transport;other biological processes	plasma membrane;other membranes;ER/Golgi;other cell component	cytoskeletal activity;other molecular function	Pf00595, Pf02828	22343	ENSMUSG00000027162
Q9D1I5	Methylmalonyl-CoA epimerase, mitochondrial OS=Mus musculus OX=10090	18	3	5	3	178	19	9,09	other metabolic processes		other molecular function	Pf13669	73724	ENSMUSG00000033429
Q9CZT6	Protein CMSS1 OS=Mus musculus OX=10090 GN=Cmss1 PE=2 SV=1	14	4	9	4	276	31,6	8,46				Pf14617	66497	ENSMUSG00000022748
Q91YI0	Argininosuccinate lyase OS=Mus musculus OX=10090 GN=Asl PE=1 SV=1	48	22	63	21	464	51,7	6,96	other metabolic processes;developmental processes;other biological processes	other membranes;cytosol;mitochondrion;ER/Golgi;nucleus;other cell component	other molecular function	Pf00206, Pf14698	109900	ENSMUSG00000025533
Q5SSW2	Proteasome activator complex subunit 4 OS=Mus musculus OX=10090 GN=P	15	24	43	24	1843	211,1	7,01	cell organization and biogenesis;protein metabolism;DNA metabolism;other met	cytosol;nucleus;other cell component	enzyme regulator activity;other molecular function	Pf11919, Pf16507	103554	ENSMUSG00000040850
D6RGR4	WD repeat domain-containing protein 83 (Fragment) OS=Mus musculus OX=	21	4	4	4	262	29	7,06				Pf00400		ENSMUSG00000005150
Q8K194	U4/U6.U5 small nuclear ribonucleoprotein 27 kDa protein OS=Mus musculus	12	2	5	2	155	18,9	11,62	RNA metabolism OR transcription;other metabolic processes	nucleus		Pf08648	66618	ENSMUSG00000001158
P09055	Integrin beta-1 OS=Mus musculus OX=10090 GN=Itgb1 PE=1 SV=1	14	9	12	9	798	88,2	5,94	cell adhesion;cell cycle OR cell proliferation;cell organization and biogenesis;other	extracellular matrix;plasma membrane;other membranes;other cytoplasmic organelle;other cell co	signal transduction activity or receptor binding;cytoskeletal activity;other molec	Pf00362, Pf07965, Pf07974, Pf08725, Pf1	16412	ENSMUSG00000025809
A2CG63	AT-rich interactive domain-containing protein 4B OS=Mus musculus OX=100	1	2	2	2	1314	147,6	5,06	cell organization and biogenesis;protein metabolism;other metabolic processes;d	cytosol;mitochondrion;nucleus	nucleic acid binding activity	Pf01388, Pf08169, Pf11717	94246	ENSMUSG00000039219
J3QN19	DNA primase OS=Mus musculus OX=10090 GN=Prim1 PE=1 SV=1	23	11	14	11	418	49,4	8,6	RNA metabolism OR transcription;other metabolic processes		other molecular function	Pf01896		ENSMUSG00000025395
Q9JJA2	Conserved oligomeric Golgi complex subunit 8 OS=Mus musculus OX=10090	3	2	2	2	640	71,6	5,19	transport	other membranes;ER/Golgi		Pf04124	97484	ENSMUSG00000031916
Q9CZN8	Glutamyl-tRNA(Gln) amidotransferase subunit A, mitochondrial OS=Mus mu	19	9	11	9	525	56,7	5,95	protein metabolism;RNA metabolism OR transcription;other metabolic processes;	mitochondrion;other cell component	other molecular function	Pf01425	76563	ENSMUSG00000019863
P63101	14-3-3 protein zeta/delta OS=Mus musculus OX=10090 GN=Ywhaz PE=1 SV=	66	16	440	12	245	27,8	4,79	cell-cell signaling;cell organization and biogenesis;protein metabolism;other meta	cytosol;nucleus;other cell component	other molecular function	Pf00244	22631	ENSMUSG00000022285
P47811	Mitogen-activated protein kinase 14 OS=Mus musculus OX=10090 GN=Map	39	12	21	10	360	41,3	5,88	protein metabolism;other metabolic processes;stress response;developmental pr	cytosol;cytoskeleton;mitochondrion;nucleus;other cell component	kinase activity;other molecular function	Pf00069	26416	ENSMUSG00000053436
Q8BX10	Serine/threonine-protein phosphatase PGAM5, mitochondrial OS=Mus musc	34	9	16	9	288	32	9,04	protein metabolism;other metabolic processes;other biological processes	other membranes;mitochondrion	enzyme regulator activity;other molecular function	Pf00300	72542	ENSMUSG00000029500
Q3U5F4	YrdC domain-containing protein, mitochondrial OS=Mus musculus OX=10090	13	3	3	3	280	29,4	6,07	other biological processes	other membranes;mitochondrion;other cell component	nucleic acid binding activity;other molecular function	Pf01300	230734	ENSMUSG00000028889
Q99LU0	Charged multivesicular body protein 1b-1 OS=Mus musculus OX=10090 GN=	23	6	8	2	199	22,1	8,1	cell cycle OR cell proliferation;cell organization and biogenesis;transport;other bio	other membranes;cytosol;nucleus;other cytoplasmic organelle;other cell component	other molecular function	Pf03357	67064	ENSMUSG00000109901
Q99N84	28S ribosomal protein S18b, mitochondrial OS=Mus musculus OX=10090 GN	41	6	11	6	254	28,7	8,35	protein metabolism;other metabolic processes	mitochondrion;translational apparatus;nucleus;other cell component	other molecular function	Pf01084	66973	ENSMUSG00000024436
Q9D2C6	DNA-directed RNA polymerase III subunit RPC8 OS=Mus musculus OX=1009	27	3	7	3	204	22,9	4,58	RNA metabolism OR transcription;other metabolic processes;stress response	cytoskeleton;nucleus;other cell component	nucleic acid binding activity;other molecular function	Pf03876, Pf08292	78929	ENSMUSG00000022476
P63260	Actin, cytoplasmic 2 OS=Mus musculus OX=10090 GN=Actg1 PE=1 SV=1	79	29	1481	7	375	41,8	5,48	cell organization and biogenesis;stress response;transport;developmental proces	non-structural extracellular;plasma membrane;other membranes;cytosol;cytoskeleton;other cell c	cytoskeletal activity;other molecular function	Pf00022	11465	ENSMUSG00000062825
B2RQC6	CAD protein OS=Mus musculus OX=10090 GN=Cad PE=1 SV=1	44	71	160	71	2225	243,1	6,43	protein metabolism;other metabolic processes;stress response;transport;develop	cytosol;nucleus;other cell component	kinase activity;other molecular function	Pf00117, Pf00185, Pf00988, Pf01979, Pf0	69719	ENSMUSG00000013629
Q8R3P8	Ring finger protein 113A1 OS=Mus musculus OX=10090 GN=Rnf113a1 PE=1	16	7	22	2	341	38,4	7,06	RNA metabolism OR transcription;other metabolic processes	nucleus	other molecular function	Pf00642, Pf13920	69942	ENSMUSG00000036537
Q60848	Lymphocyte-specific helicase OS=Mus musculus OX=10090 GN=Hells PE=1 S	12	7	8	7	821	95,1	8	cell cycle OR cell proliferation;cell organization and biogenesis;DNA metabolism;c	nucleus;other cell component	other molecular function	Pf00176, Pf00271	15201	ENSMUSG00000025001
P43346	Deoxycytidine kinase OS=Mus musculus OX=10090 GN=Dck PE=1 SV=1	33	8	19	8	260	30,3	5,43	other metabolic processes	cytosol;mitochondrion;nucleus;other cell component	kinase activity;other molecular function	Pf01712	13178	ENSMUSG00000029366
Q8R3F5	Malonyl-CoA-acyl carrier protein transacylase, mitochondrial OS=Mus muscu	25	6	14	6	381	41,9	8,1	other metabolic processes		other molecular function	Pf00698	223722	ENSMUSG00000048755
A2ABY3	CTP:phosphoethanolamine cytidylyltransferase OS=Mus musculus OX=1009	46	17	37	17	386	43,4	6,73	other metabolic processes		other molecular function	Pf01467	68671	ENSMUSG00000025137
Q9CPR7	Suppressor of IKBKE 1 OS=Mus musculus OX=10090 GN=Sike1 PE=1 SV=1	24	4	6	4	207	23,5	5,38		other cell component	other molecular function	Pf05769	66641	ENSMUSG00000027854

[illegible]

Q8K114	Integrator complex subunit 9 OS=Mus musculus OX=10090 GN=Ints9 PE=1 S	13	7	10	7	658	74	6,4	RNA metabolism OR transcription;other metabolic processes	cytosol;nucleus		Pf16661	210925	ENSMUSG00000021975	Ints9	RNA polymerase II transcribes snRNA genes		0
A0A3Q4EI12	Ras-related protein Rab-18 OS=Mus musculus OX=10090 GN=Rab18 PE=1 SV	24	4	6	4	212	23,8	5,25	cell organization and biogenesis	plasma membrane;other membranes;ER/Golgi;other cell component	other molecular function	Pf00071	19330	ENSMUSG00000073639	Rab18			0
Q91YD9	Neural Wiskott-Aldrich syndrome protein OS=Mus musculus OX=10090 GN=	19	10	17	10	501	54,2	7,93	cell cycle OR cell proliferation;cell organization and biogenesis;transport;developr	other membranes;cytosol;cytoskeleton;ER/Golgi;nucleus;other cell component	cytoskeletal activity;other molecular function	Pf00568, Pf00786, Pf02205	73178	ENSMUSG00000029684	Wasl	Clathrin-mediated endocytosis; EPHB-mediated forward signali	Chemokine signaling pathway; G13 signaling pathway;	0
Q99PP9	Tripartite motif-containing protein 16 OS=Mus musculus OX=10090 GN=Trim	5	2	2	2	556	62,9	5,86	protein metabolism;other metabolic processes;other biological processes	plasma membrane;other membranes;cytosol;nucleus;other cell component	nucleic acid binding activity;other molecular function	Pf00622, Pf00643, Pf13765	94092	ENSMUSG00000047821	Trim16			0
Q8BUM3	Tyrosine-protein phosphatase non-receptor type 7 OS=Mus musculus OX=10	7	2	3	2	359	40,3	6,7	protein metabolism;other metabolic processes	plasma membrane;other membranes;cytosol;cytoskeleton;other cell component	other molecular function	Pf00102	320139	ENSMUSG00000031506	Ptpn7	Negative regulation of MAPK pathway	Mapk signaling pathway	0
Q9WTP6	Adenylate kinase 2, mitochondrial OS=Mus musculus OX=10090 GN=Ak2 PE	62	15	198	11	239	26,5	7,39	other metabolic processes	cytosol;cytoskeleton;mitochondrion;nucleus;other cell component	kinase activity;other molecular function	Pf00406, Pf05191	11637	ENSMUSG00000028792	Ak2	Interconversion of nucleotide di- and triphosphates	Purine metabolism	8
Q9ER81	Torsin-1A-interacting protein 2, isoform IFRG15 OS=Mus musculus OX=1009	47	4	7	4	131	15,3	7,31	cell organization and biogenesis;other biological processes	other membranes;ER/Golgi	other molecular function		240832	ENSMUSG00000050565	Tor1aip2			0
Q8BGK6	Y+L amino acid transporter 2 OS=Mus musculus OX=10090 GN=Slc7a6 PE=1	8	3	3	3	515	56,7	7,3	transport	plasma membrane;other membranes;other cell component	transporter activity	Pf13520	330836	ENSMUSG00000031904	Slc7a6	Basigin interactions; Amino acid transport across the plasma m		0
Q3U0M1	Trafficking protein particle complex subunit 9 OS=Mus musculus OX=10090 (	3	3	3	3	1148	128,2	6,47	developmental processes;other biological processes	ER/Golgi;other cell component		Pf08626	76510	ENSMUSG00000047921	Trappc9	RAB GEFs exchange GTP for GDP on RABs; COPII-mediated ves		0
A2AIV8	Caspase recruitment domain-containing protein 9 OS=Mus musculus OX=10(	12	5	9	5	536	62,4	5,73	cell organization and biogenesis;stress response;signal transduction;other biologi	cytosol;other cell component	other molecular function	Pf00619	332579	ENSMUSG00000026928	Card9	CLEC7A (Dectin-1) signaling		0
Q8C0M9	Isoaspartyl peptidase/L-asparaginase OS=Mus musculus OX=10090 GN=Asr	57	15	57	15	326	33,9	7,65	protein metabolism;other metabolic processes	other cell component	other molecular function	Pf01112	66514	ENSMUSG00000024654	Asrgl1	Phenylalanine metabolism		0
E9PV41	Protein diaphanous homolog 1 OS=Mus musculus OX=10090 GN=Diaph1 PE=	11	12	13	12	1264	140,3	5,58	cell organization and biogenesis;other biological processes	cytoskeleton	cytoskeletal activity;other molecular function	Pf02181, Pf06346, Pf06367, Pf06371	13367	ENSMUSG00000024456	Diaph1		G13 signaling pathway; Regulation of actin cytoskeleton	0
P53994	Ras-related protein Rab-2A OS=Mus musculus OX=10090 GN=Rab2a PE=1 S	53	9	19	9	212	23,5	6,54	cell organization and biogenesis;transport	other membranes;ER/Golgi;other cell component	other molecular function	Pf00071	59021	ENSMUSG00000047187	Rab2a	RAB geranylgeranylation; Golgi Cisternae Pericentriolar Stack R	Focal adhesion: PI3K-Akt-mTOR signaling pathway	0
Q3UQN2	F-BAR domain only protein 2 OS=Mus musculus OX=10090 GN=Fcho2 PE=1 S	9	7	10	7	809	88,7	6,89	cell organization and biogenesis;transport;other biological processes	plasma membrane;other membranes;other cell component	other molecular function	Pf00611, Pf10291	218503	ENSMUSG00000041685	Fcho2	Cargo recognition for clathrin-mediated endocytosis		0
P58801	Receptor-interacting serine/threonine-protein kinase 2 OS=Mus musculus O)	4	2	2	2	539	60,4	7,2	cell cycle OR cell proliferation;protein metabolism;other metabolic processes;stre	cytosol;cytoskeleton;other cell component	signal transduction activity or receptor binding;kinase activity;other molecular f	Pf00069, Pf07714	192656	ENSMUSG00000041135	Ripk2	Downstream TCR signaling; Ovarian tumor domain proteases;	FAS pathway and stress induction of HSP regulation	0
Q08943	FACT complex subunit SSRP1 OS=Mus musculus OX=10090 GN=Ssrp1 PE=1 S	34	20	39	20	708	80,8	6,76	DNA metabolism;other metabolic processes;stress response;other biological proc	nucleus	nucleic acid binding activity;other molecular function	Pf00505, Pf03531, Pf08512, Pf17292	20833	ENSMUSG00000027067	Ssrp1	Formation of RNA Pol II elongation complex ; RNA Polymerase	Mechanisms associated with pluripotency	0
E9PUK3	Formin-binding protein 1-like OS=Mus musculus OX=10090 GN=Fnbp1l PE=1	15	8	10	8	551	63,9	6,27	other metabolic processes;transport;signal transduction	plasma membrane;other membranes;cytoskeleton;other cell component		Pf00018, Pf00611	214459	ENSMUSG00000039735	Fnbp1l			0
P07356	Annexin A2 OS=Mus musculus OX=10090 GN=Anxa2 PE=1 SV=2	53	20	99	20	339	38,7	7,69	cell organization and biogenesis;transport;developmental processes;other biolog	non-structural extracellular;extracellular matrix;plasma membrane;other membranes;cytosol;nucle	transporter activity;enzyme regulator activity;cytoskeletal activity;other molec	Pf00191	12306	ENSMUSG00000032231	Anxa2	Neutrophil degranulation; Dissolution of Fibrin Clot	Prostaglandin synthesis and regulation	0
B2RXT3	Oxoglutarate dehydrogenase (succinyl-transferring) OS=Mus musculus OX=	29	25	65	17	1010	114,5	6,84	other metabolic processes	mitochondrion;other cell component	other molecular function	Pf00676, Pf02779, Pf16078, Pf16870	239017	ENSMUSG00000020913	Ogdhl			0
Q3TQC7	Ectonucleoside triphosphate diphosphohydrolase 5 OS=Mus musculus OX=1(	4	2	2	2	452	49,8	5,47		non-structural extracellular;other membranes	other molecular function	Pf01150	12499	ENSMUSG00000021236	Entpd5		Purine metabolism	0
Q70FJ1	A-kinase anchor protein 9 OS=Mus musculus OX=10090 GN=Akap9 PE=1 SV	1	3	3	2	3797	435,9	5,03	cell organization and biogenesis;developmental processes;signal transduction;oth	plasma membrane;other membranes;cytoskeleton;ER/Golgi;other cell component	other molecular function	Pf10495	100986		Akap9	Anchoring of the basal body to the plasma membrane; Regulat	G protein signaling pathways	0
Q8CHE4	PH domain leucine-rich repeat-containing protein phosphatase 1 OS=Mus mu	7	8	9	8	1687	182,3	6,19	protein metabolism;other metabolic processes;signal transduction;other biologica	plasma membrane;other membranes;nucleus;other cell component	other molecular function	Pf00169, Pf00481, Pf13516, Pf13855	98432	ENSMUSG00000044340	Phlpp1	Negative regulation of the PI3K/AKT network	Focal adhesion: PI3K-Akt-mTOR signaling pathway	0
Q8CEE6	PAS domain-containing serine/threonine-protein kinase OS=Mus musculus (	4	4	5	4	1383	151,2	5,07	protein metabolism;other metabolic processes;signal transduction;other biologica	cytosol;nucleus;other cell component	kinase activity;other molecular function	Pf00069, Pf13426	269224	ENSMUSG00000026274	Pask			0
Q99N92	39S ribosomal protein L27, mitochondrial OS=Mus musculus OX=10090 GN=	24	3	5	3	148	15,9	10,17	protein metabolism;other metabolic processes	mitochondrion;translational apparatus	other molecular function	Pf01016	94064	ENSMUSG00000024414	Mrpl27	Mitochondrial translation elongation; Mitochondrial translation		0
Q9JMK2	Casein kinase I isoform epsilon OS=Mus musculus OX=10090 GN=Csnk1e PE	8	3	4	2	416	47,3	9,66	cell-cell signaling;protein metabolism;other metabolic processes;transport;signal	cytosol;nucleus;other cell component	kinase activity;other molecular function	Pf00069	27373	ENSMUSG00000022433	Csnk1e	Major pathway of rRNA processing in the nucleolus and cytosol	Wnt signaling pathway; Wnt signaling pathway; Wnt si	1
Q8C7U7	Polypeptide N-acetylgalactosaminyltransferase 6 OS=Mus musculus OX=10(	4	2	3	2	622	71,5	8,53	protein metabolism;other metabolic processes	other membranes;ER/Golgi;other cell component	other molecular function	Pf00535, Pf00652	207839	ENSMUSG00000037280	Galnt6	O-linked glycosylation of mucins		0
Q80ZS3	28S ribosomal protein S26, mitochondrial OS=Mus musculus OX=10090 GN=	28	6	14	6	200	23,4	9,96		mitochondrion;translational apparatus;nucleus		Pf14943	99045	ENSMUSG00000037740	Mrps26	Mitochondrial translation elongation; Mitochondrial translation		0
Q6NXI6	Regulation of nuclear pre-mRNA domain-containing protein 2 OS=Mus muscu	2	2	2	2	1469	156,5	7,59	RNA metabolism OR transcription;other metabolic processes	nucleus	other molecular function	Pf04818	75137	ENSMUSG00000028106	Rprd2	RNA polymerase II transcribes snRNA genes		0
A0A0R4J024	DNA repair protein XRCC4 OS=Mus musculus OX=10090 GN=Xrcc4 PE=1 SV=	39	13	18	13	326	37,1	5,25	DNA metabolism;other metabolic processes;stress response;other biological proc	cytosol;nucleus;other cell component	nucleic acid binding activity;other molecular function	Pf06632	108138	ENSMUSG00000021615	Xrcc4		Non-homologous end joining	0
O70318	Band 4.1-like protein 2 OS=Mus musculus OX=10090 GN=Epb41l2 PE=1 SV=	40	33	61	32	988	109,9	5,43	cell cycle OR cell proliferation;cell organization and biogenesis;other biological pro	plasma membrane;other membranes;cytoskeleton;nucleus;other cell component	cytoskeletal activity;other molecular function	Pf00373, Pf04382, Pf05902, Pf08736, Pf(	13822	ENSMUSG00000019978	Epb41l2	Neurexins and neuroligins	Splicing factor NOVA regulated synaptic proteins	1
A0A0N4SW28	Guanine nucleotide-binding protein subunit gamma OS=Mus musculus OX=1	30	2	3	2	83	9,2	9,25	developmental processes;signal transduction;other biological processes	plasma membrane;other membranes;cytoskeleton	other molecular function	Pf00631		ENSMUSG00000036402	Gng12		Myometrial relaxation and contraction pathways; Calciu	0
P28659	CUGBP Elav-like family member 1 OS=Mus musculus OX=10090 GN=Celf1 PE	14	8	14	8	486	52,1	8,46	cell organization and biogenesis;RNA metabolism OR transcription;other metaboli	nucleus;other cell component	nucleic acid binding activity;other molecular function	Pf00076	13046	ENSMUSG00000005506	Celf1		mRNA processing; Adipogenesis genes	0
Q6PDJ6	F-box only protein 42 OS=Mus musculus OX=10090 GN=Fbxo42 PE=1 SV=1	8	5	6	5	717	77,7	7,58				Pf12937, Pf13415, Pf13854	213499	ENSMUSG00000028920	Fbxo42			0
Q62348	Translin OS=Mus musculus OX=10090 GN=Tsn PE=1 SV=1	50	13	57	13	228	26,2	6,44	RNA metabolism OR transcription;other metabolic processes	ER/Golgi;nucleus;other cell component	nucleic acid binding activity;other molecular function	Pf01997	22099	ENSMUSG00000026374	Tsn	Small interfering RNA (siRNA) biogenesis	mRNA processing	0
Q8BFX3	BTB/POZ domain-containing protein KCTD3 OS=Mus musculus OX=10090 GN	8	6	7	4	815	88,8	7,15	cell organization and biogenesis	plasma membrane;other membranes		Pf02214	226823	ENSMUSG00000026608	Kctd3	CDC42 GTPase cycle		2
Q9D4J7	PHD finger protein 6 OS=Mus musculus OX=10090 GN=Phf6 PE=1 SV=1	18	7	13	7	364	41,1	8,73	developmental processes;other biological processes	nucleus;other cell component	cytoskeletal activity;nucleic acid binding activity;other molecular function	Pf13771	70998	ENSMUSG00000025626	Phf6			0
G3X912	DNA-dependent metalloprotease SPRTN OS=Mus musculus OX=10090 GN=S	8	3	4	3	497	55,3	8,9	protein metabolism;DNA metabolism;other metabolic processes;stress response;	nucleus;other cell component	other molecular function	Pf10263	244666	ENSMUSG00000031986	Sprtn	Translesion Synthesis by POLH		0
Q6NZD2	Sorting nexin-1 OS=Mus musculus OX=10090 GN=Snx1 PE=1 SV=1	46	19	42	17	521	58,8	5,26	cell organization and biogenesis;protein metabolism;other metabolic processes;transport;other biol	other membranes;cytosol;ER/Golgi;other cytoplasmic organelle;other cell component	signal transduction activity or receptor binding;other molecular function	Pf00787, Pf03700, Pf09325	56440	ENSMUSG00000032382	Snx1			0
Q8R5A0	N-lysine methyltransferase SMYD2 OS=Mus musculus OX=10090 GN=Smyd	12	5	7	5	433	49,5	6,95	protein metabolism;other metabolic processes;developmental processes;other b	cytosol;nucleus;other cell component	other molecular function	Pf00856, Pf01753	226830	ENSMUSG00000026603	Smyd2	Regulation of TP53 Activity through Methylation; PKMTs meth		0
Q9Z1J3	Cysteine desulfurase, mitochondrial OS=Mus musculus OX=10090 GN=Nfs1	32	13	22	13	459	50,5	8,16	cell organization and biogenesis;protein metabolism;other metabolic processes	cytosol;mitochondrion;nucleus	other molecular function	Pf00266	18041	ENSMUSG00000027618	Nfs1	Molybdenum cofactor biosynthesis; Mitochondrial iron-sulfur c		0
A0A0R4J0U8	TRMT1-like protein OS=Mus musculus OX=10090 GN=Trmt1l PE=1 SV=1	34	22	30	21	728	80,9	7,61	RNA metabolism OR transcription;other metabolic processes;other biological proc		nucleic acid binding activity;other molecular function	Pf02005	98685	ENSMUSG00000053286	Trmt1l		mRNA processing	1
Q91YW3	DnaJ homolog subfamily C member 3 OS=Mus musculus OX=10090 GN=Dna	30	15	28	15	504	57,4	5,85	protein metabolism;other metabolic processes;stress response;signal transductio	cytosol;ER/Golgi;other cell component	enzyme regulator activity;other molecular function	Pf00226, Pf00515, Pf13181, Pf13432	100037258	ENSMUSG00000022136	Dnajc3	Neutrophil degranulation; Regulation of Insulin-like Growth Fac		0
A0A0A6YW28	Ubiquitin carboxyl-terminal hydrolase OS=Mus musculus OX=10090 GN=Usp	10	7	9	5	915	103,7	5,48	protein metabolism;other metabolic processes	nucleus;other cell component	other molecular function	Pf00443, Pf06337, Pf14836	22258	ENSMUSG00000032612	Usp4			0
E0CYA9	DNA polymerase epsilon subunit 4 OS=Mus musculus OX=10090 GN=Pole4 F	40	4	10	4	119	12,4	5,25		nucleus;other cell component	other molecular function	Pf00808		ENSMUSG00000030042	Pole4			0
Q9CRA5	Golgi phosphoprotein 3 OS=Mus musculus OX=10090 GN=Golph3 PE=1 SV=1	27	8	13	8	298	33,7	6,44	cell adhesion;cell organization and biogenesis;protein metabolism;other metaboli	plasma membrane;other membranes;cytosol;mitochondrion;ER/Golgi;other cytoplasmic organelle	other molecular function	Pf05719	66629	ENSMUSG00000022200	Golph3			0
D3Z380	Thiopurine S-methyltransferase OS=Mus musculus OX=10090 GN=Tpmt PE=	18	3	3	2	205	23,4	6,95	other metabolic processes	other cell component	other molecular function	Pf05724		ENSMUSG00000021376	Tpmt		Methylation; Metapathway biotransformation	0
Q8VE38	Oxidoreductase NAD-binding domain-containing protein 1 OS=Mus musculus	21	6	12	5	311	34,7	8,13			other molecular function	Pf00175	218885	ENSMUSG00000021906	Oxnad1			1
Q9WU80	RanBP-type and C3HC4-type zinc finger-containing protein 1 OS=Mus muscul	19	9	10	9	508	57,5	6,19	protein metabolism;other metabolic processes;signal transduction;other biologica	other cell component	other molecular function	Pf13445	24105	ENSMUSG00000027466	Rbck1	Antigen processing: Ubiquitination & Proteasome degradation;		0
Q60823	RAC-beta serine/threonine-protein kinase OS=Mus musculus OX=10090 GN=	16	7	11	5	481	55,7	6,37	cell organization and biogenesis;protein metabolism;other metabolic processes;s	plasma membrane;other membranes;cytosol;mitochondrion;ER/Golgi;nucleus;other cytoplasmic o	kinase activity;other molecular function	Pf00069, Pf00169, Pf00433	11652	ENSMUSG00000004056	Akt2	RAB GEFs exchange GTP for GDP on RABs; Deactivation of the I	MicroRNAs in cardiomyocyte hypertrophy; Chemokine s	0
Q9WTK2	Chromodomain Y-like protein OS=Mus musculus OX=10090 GN=Cdyl PE=1 S	7	4	4	4	593	65,2	8,6	cell organization and biogenesis;protein metabolism;other metabolic processes;s	nucleus;other cell component	other molecular function	Pf00378, Pf00385	12593	ENSMUSG00000059288	Cdyl			0
Q91WN1	DnaJ homolog subfamily C member 9 OS=Mus musculus OX=10090 GN=Dna	42	12	24	12	259	30	5,94	other biological processes	non-structural extracellular;plasma membrane;other membranes;cytosol;nucleus;other cell compo	other molecular function	Pf00226	108671	ENSMUSG00000021811	Dnajc9			0
P14901	Heme oxygenase 1 OS=Mus musculus OX=10090 GN=Hmox1 PE=1 SV=1	34	7	21	7	289	32,9	6,54	other metabolic processes;stress response;developmental processes;signal trans	plasma membrane;other membranes;cytosol;ER/Golgi;nucleus;other cell component	other molecular function	Pf01126	15368	ENSMUSG00000005413	Hmox1	Iron uptake and transport; Cytoprotection by HMOX1; Heme d	Transcriptional activation by Nfe2l2 in response to phyto	0
A0A3B2W8S3	Kelch domain-containing protein 3 OS=Mus musculus OX=10090 GN=Klhdc3	19	5	10	5	373	42,1	8,05				Pf01344, Pf07646, Pf13418, Pf13964		ENSMUSG00000063576	Klhdc3			0
Q8K4F5	Protein ABHD11 OS=Mus musculus OX=10090 GN=Abhd11 PE=1 SV=1	22	7	11	7	307	33,5	9,61			other molecular function	Pf12697	68758	ENSMUSG00000040532	Abhd11			0
Q921W0	Charged multivesicular body protein 1a OS=Mus musculus OX=10090 GN=Ch	37	10	26	10	196	21,6	8,06	cell cycle OR cell proliferation;cell organization and biogenesis;transport;other bio	other membranes;cytoskeleton;nucleus;other cytoplasmic organelle;other cell component	other molecular function	Pf03357	234852	ENSMUSG00000000743	Chmp1a			0
O88271	Craniofacial development protein 1 OS=Mus musculus OX=10090 GN=Cfdp1	55	17	83	17	295	32,9	4,86	cell adhesion;other biological processes	extracellular matrix;other cell component		Pf07572	23837	ENSMUSG00000031954	Cfdp1			0
Q5SFM8	RNA-binding protein 27 OS=Mus musculus OX=10090 GN=Rbm27 PE=1 SV=3	11	11	18	10	1060	118,5	9,19	RNA metabolism OR transcription;other metabolic processes	nucleus;other cell component	nucleic acid binding activity;other molecular function	Pf00076, Pf00642, Pf01480	225432	ENSMUSG00000024491	Rbm27			0
Q91VA6	Polymerase delta-interacting protein 2 OS=Mus musculus OX=10090 GN=Pol	38	12	32	12	368	41,8	8,63	cell cycle OR cell proliferation;cell organization and biogenesis;DNA metabolism;o	other membranes;cytosol;cytoskeleton;mitochondrion;nucleus;other cell component	other molecular function	Pf04379, Pf08755	67811	ENSMUSG00000001100	Poldip2			0
Q9DAP7	Histone chaperone ASF1B OS=Mus musculus OX=10090 GN=Asf1b PE=1 SV	18	3	4	3	202	22,5	4,44	cell organization and biogenesis;developmental processes	nucleus;other cell component	other molecular function	Pf04729	66929	ENSMUSG00000005470	Asf1b			0
Q8BHT6	Beta-1,3-glucosyltransferase OS=Mus musculus OX=10090 GN=B3glct PE=1	15	5	5	5	489	55,3	6,92	protein metabolism;other metabolic processes	other membranes;ER/Golgi	other molecular function	Pf02434	381694	ENSMUSG00000051950	B3glct	O-glycosylation of TSR domain-containing proteins		0
Q6NXY9	DNA-directed RNA polymerase III subunit RPC7 OS=Mus musculus OX=1009	13	3	4	3	223	25,9	4,59	cell cycle OR cell proliferation;RNA metabolism OR transcription;other metabolic p	cytosol;nucleus	other molecular function	Pf11705	67486	ENSMUSG00000035834	Polr3g	RNA Polymerase III Transcription Initiation From Type 1 Promo	Purine metabolism	0
P97376	Protein FRG1 OS=Mus musculus OX=10090 GN=Frg1 PE=1 SV=2	34	8	13	8	258	29,1	8,92	RNA metabolism OR transcription;other metabolic processes;developmental proc	cytoskeleton;nucleus	cytoskeletal activity;nucleic acid binding activity	Pf06229	14300	ENSMUSG00000031590	Frg1			0
A2A841	Band 4.1 OS=Mus musculus OX=10090 GN=Epb41 PE=1 SV=1	41	27	45	19	869	97,2	5,49	cell cycle OR cell proliferation;cell organization and biogenesis;other biological pr	non-structural extracellular;plasma membrane;other membranes;cytosol;cytoskeleton;nucleus;oth	cytoskeletal activity;other molecular function	Pf00373, Pf04382, Pf05902, Pf08736, Pf(		ENSMUSG00000028906	Epb41			7
Q6P3D0	U8 snoRNA-decapping enzyme OS=Mus musculus OX=10090 GN=Nudt16 PE	21	4	4	3	195	21,8	7,12	cell organization and biogenesis;protein metabolism;RNA metabolism OR transcri	nucleus;other cell component	other molecular function	Pf00293	75686	ENSMUSG00000032565	Nudt16	Phosphate bond hydrolysis by NUDT proteins	Purine metabolism	0
Q9D0B0	Serine/arginine-rich splicing factor 9 OS=Mus musculus OX=10090 GN=Srsf9	28	7	11	6	222	25,6	8,65	RNA metabolism OR transcription;other metabolic processes	nucleus	nucleic acid binding activity;other molecular function	Pf00076	108014	ENSMUSG00000029538	Srsf9	mRNA Splicing - Major Pathway; Transport of Mature mRNA de	mRNA processing	0
Q8R5M8	Cell adhesion molecule 1 OS=Mus musculus OX=10090 GN=Cadm1 PE=1 SV	15	4	7	4	456	49,8	5,03	cell adhesion;cell-cell signaling;cell organization and biogenesis;developmental p	plasma membrane;other membranes;other cell component	signal transduction activity or receptor binding;other molecular function	Pf07686, Pf08205, Pf13927	54725	ENSMUSG00000032076	Cadm1	Nectin/Necl trans heterodimerization	Splicing factor NOVA regulated synaptic proteins	0
E9PXC2	Upstream stimulatory factor 2 OS=Mus musculus OX=10090 GN=Usf2 PE=1 S	23	5	9	5	215	24	5,86			other molecular function	Pf00010		ENSMUSG00000058239	Usf2			1
Q99LF7	DDB1 and CUL4-associated factor 4 OS=Mus musculus OX=10090 GN=Dcaf4	6	3	3	3	519	58,1	8,37		other cell component			73828	ENSMUSG00000021222	Dcaf4			0
Q9JJG9	Nitric oxide-associated protein 1 OS=Mus musculus OX=10090 GN=Noa1 PE=	6	4	5	4	693	77,3	8,12	protein metabolism;other metabolic processes;other biological processes	other membranes;mitochondrion		Pf01926	56412	ENSMUSG00000036285	Noa1			0
P19783	Cytochrome c oxidase subunit 4 isoform 1, mitochondrial OS=Mus musculus (	36	6	7	6	169	19,5	9,23	other metabolic processes;other biological processes	other membranes;cytosol;mitochondrion;nucleus	other molecular function	Pf02936	12857	ENSMUSG00000031818	Cox4i1	Cytoprotection by HMOX1; TP53 Regulates Metabolic Genes; F	Electron transport chain	0
Q9D880	Mitochondrial import inner membrane translocase subunit TIM50 OS=Mus n	23	6	10	6	353	39,8	8,13	cell organization and biogenesis;protein metabolism;other metabolic processes;t	other membranes;mitochondrion;nucleus	signal transduction activity or receptor binding;other molecular function	Pf03031	66525	ENSMUSG00000003438	Timm50			0
O35134	DNA-directed RNA polymerase I subunit RPA1 OS=Mus musculus OX=10090	21	29	40	28	1717	194	6,93	RNA metabolism OR transcription;other metabolic processes	nucleus;other cell component	nucleic acid binding activity;other molecular function	Pf00623, Pf04983, Pf04997, Pf04998, Pf(	20019	ENSMUSG00000049553	Polr1a	RNA Polymerase I Transcription Initiation; RNA Polymerase I P	Purine metabolism; Eukaryotic transcription initiation	0
Q9WTK7	Serine/threonine-protein kinase STK11 OS=Mus musculus OX=10090 GN=St	20	7	9	7	436	49,2	6,84	cell-cell signaling;cell cycle OR cell proliferation;cell organization and biogenesis;p	other membranes;cytosol;mitochondrion;nucleus;other cell component	enzyme regulator activity;kinase activity;other molecular function	Pf00069	20869	ENSMUSG00000003068	Stk11	Regulation of TP53 Activity through Phosphorylation; Energy d	Focal adhesion: PI3K-Akt-mTOR signaling pathway	0

[illegible]

A0A0G2JDJ5	Farnesyl pyrophosphate synthase (Fragment) OS=Mus musculus OX=10090	64	9	68	2	121	13,9	5,95	other metabolic processes			[illegible]
A0A2I3BR81	5'-nucleotidase domain-containing 2 OS=Mus musculus OX=10090 GN=Nt5d	20	10	15	10	553	63,8	7,59	other metabolic processes		other molecular functi	[illegible]
Q9D2V5	Protein AAR2 homolog OS=Mus musculus OX=10090 GN=Aar2 PE=1 SV=3	21	6	6	6	384	43,4	5,48	cell organization and biogenesis;RNA metabolism OR transcription;other metabol	nucleus		[illegible]
Q61334	B-cell receptor-associated protein 29 OS=Mus musculus OX=10090 GN=Bcap	17	5	7	5	240	27,9	9,72	transport;other biological processes	other membranes;ER/Golgi		[illegible]
Q6P9Q6	FK506-binding protein 15 OS=Mus musculus OX=10090 GN=Fkbp15 PE=1 SV	14	14	19	14	1216	132,9	5,07	protein metabolism;other metabolic processes;transport	other membranes;cytoskeleton;other cytoplasmic organelle;other cell component	cytoskeletal activity	[illegible]
Q8R395	COMM domain-containing protein 5 OS=Mus musculus OX=10090 GN=Com	12	3	4	3	224	24,5	8,06		cytosol;nucleus		[illegible]
Q8CG48	Structural maintenance of chromosomes protein 2 OS=Mus musculus OX=10	23	26	40	26	1191	134,2	8,41	cell cycle OR cell proliferation;cell organization and biogenesis;DNA metabolism;s	nucleus;other cell component	nucleic acid binding ac	[illegible]
Q9D6S7	Ribosome-recycling factor, mitochondrial OS=Mus musculus OX=10090 GN=	30	6	20	6	262	29	9,85	cell organization and biogenesis;protein metabolism;other metabolic processes		translation activity	[illegible]
Q9DCH4	Eukaryotic translation initiation factor 3 subunit F OS=Mus musculus OX=100	30	9	19	9	361	38	5,58	cell organization and biogenesis;protein metabolism;other metabolic processes;o	translational apparatus;other cell component	nucleic acid binding ac	[illegible]
Q99LI7	Cleavage stimulation factor subunit 3 OS=Mus musculus OX=10090 GN=Cstf	20	13	21	13	717	82,8	8,12	RNA metabolism OR transcription;other metabolic processes	nucleus	nucleic acid binding ac	[illegible]
Q9CQV1	Mitochondrial import inner membrane translocase subunit TIM16 OS=Mus n	31	4	4	4	125	13,8	9,64	cell organization and biogenesis;DNA metabolism;other metabolic processes;trar	other membranes;mitochondrion;other cell component		[illegible]
Q8BYZ1	ABI gene family member 3 OS=Mus musculus OX=10090 GN=Abi3 PE=1 SV=	10	3	3	3	367	39,1	5,54	stress response;other biological processes	other cell component	cytoskeletal activity;c	[illegible]
E9PW20	Chromatin target of PRMT1 protein OS=Mus musculus OX=10090 GN=Chtop	43	8	24	8	203	22,1	11,97			nucleic acid binding ac	[illegible]
Q61048	WW domain-binding protein 4 OS=Mus musculus OX=10090 GN=Wbp4 PE=	39	12	24	12	376	42,1	7,36	RNA metabolism OR transcription;other metabolic processes	nucleus	nucleic acid binding ac	[illegible]
P70700	DNA-directed RNA polymerase I subunit RPA2 OS=Mus musculus OX=10090	18	20	35	20	1135	128,1	7,58	cell organization and biogenesis;RNA metabolism OR transcription;other metabol	cytosol;nucleus;other cell component	nucleic acid binding ac	[illegible]
Q9DAR7	m7GpppX diphosphatase OS=Mus musculus OX=10090 GN=Dcps PE=1 SV=1	61	17	67	17	338	39	6,48	RNA metabolism OR transcription;other metabolic processes;other biological proc	cytosol;mitochondrion;nucleus;other cell component	nucleic acid binding ac	[illegible]
Q8CFE8	F-box and WD-40 domain protein 17 OS=Mus musculus OX=10090 GN=Fbxw	5	2	2	2	466	52,2	7,66				[illegible]
Q8BHJ9	Pre-mRNA-splicing factor SLU7 OS=Mus musculus OX=10090 GN=Slu7 PE=1	8	3	4	3	585	68	7,08	cell organization and biogenesis;RNA metabolism OR transcription;other metabol	cytosol;nucleus;other cell component	nucleic acid binding ac	[illegible]
O35047	Homologous-pairing protein 2 homolog OS=Mus musculus OX=10090 GN=Ps	18	4	8	4	217	24,7	7,4	cell cycle OR cell proliferation;cell organization and biogenesis;DNA metabolism;c	nucleus	nucleic acid binding ac	[illegible]
A0A087WNZ7	E3 ubiquitin-protein ligase TRIP12 OS=Mus musculus OX=10090 GN=Trip12 F	2	4	5	3	1992	220,5	8,44	protein metabolism;DNA metabolism;other metabolic processes;stress response	nucleus	other molecular functi	[illegible]
E9Q1Y3	Apolipoprotein B-100 (Fragment) OS=Mus musculus OX=10090 GN=Apob PE	1	3	3	3	4456	503,6	6,83	transport		transporter activity	[illegible]
A2ADA5	tRNA pseudouridine synthase-like 1 OS=Mus musculus OX=10090 GN=Pusl1	11	3	3	3	291	32	10,04	RNA metabolism OR transcription;other metabolic processes	mitochondrion;other cell component	nucleic acid binding ac	[illegible]
Q9WUB4	Dynactin subunit 6 OS=Mus musculus OX=10090 GN=Dctn6 PE=1 SV=1	35	6	14	6	190	20,7	6,67	cell cycle OR cell proliferation;cell organization and biogenesis;other metabolic pr	cytoskeleton;mitochondrion;other cell component	other molecular functi	[illegible]
Q9QXK7	Cleavage and polyadenylation specificity factor subunit 3 OS=Mus musculus	16	8	15	8	684	77,5	5,6	RNA metabolism OR transcription;other metabolic processes	nucleus	nucleic acid binding ac	[illegible]
Q8BG17	Nucleolar protein 12 OS=Mus musculus OX=10090 GN=Nol12 PE=1 SV=1	25	5	8	5	217	25,3	10,13		nucleus	nucleic acid binding ac	[illegible]
Q4JK59	Methylcytosine dioxygenase TET2 OS=Mus musculus OX=10090 GN=Tet2 P	3	5	5	5	1912	212	7,84	cell cycle OR cell proliferation;protein metabolism;DNA metabolism;other metabo	nucleus	nucleic acid binding ac	[illegible]
F8VQ54	Endoribonuclease Dicer OS=Mus musculus OX=10090 GN=Dicer1 PE=1 SV=1	1	2	2	2	1906	215,6	5,8	cell organization and biogenesis;RNA metabolism OR transcription;other metabol	non-structural extracellular;ER/Golgi;other cell component	nucleic acid binding ac	[illegible]
Q9D6K7	Tetratricopeptide repeat protein 33 OS=Mus musculus OX=10090 GN=Ttc33	28	6	16	6	262	29,4	5,35				[illegible]
Q3UMW7	MAP kinase-activated protein kinase 3 OS=Mus musculus OX=10090 GN=M	30	10	14	6	384	43,3	8,09	protein metabolism;other metabolic processes;transport;signal transduction;othe	nucleus;other cell component	kinase activity;other r	[illegible]
Q9CY11	rRNA-processing protein UTP23 homolog OS=Mus musculus OX=10090 GN=	10	3	3	3	249	28,4	10,32	RNA metabolism OR transcription;other metabolic processes	nucleus;other cell component	nucleic acid binding ac	[illegible]

[illegible]

P62141	Serine/threonine-protein phosphatase PP1-beta catalytic subunit OS=Mus m	63	17	103	6	327	37,2	6,19	cell cycle OR cell proliferation;protein metabolism;other metabolic processes;othe	plasma membrane;other membranes;cytosol;nucleus;other cell component
A2AGT5	Cytoskeleton-associated protein 5 OS=Mus musculus OX=10090 GN=Ckap5	3	7	8	7	2032	225,5	7,96	cell-cell signaling;cell cycle OR cell proliferation;cell organization and biogenesis;p	plasma membrane;other membranes;cytoskeleton;nucleus;other cell component
F7CC56	RB1-inducible coiled-coil protein 1 (Fragment) OS=Mus musculus OX=10090	1	2	2	2	1468	168,9	5,52	cell organization and biogenesis;other metabolic processes	
Q4VBE8	WD repeat-containing protein 18 OS=Mus musculus OX=10090 GN=Wdr18 P	15	6	7	6	431	47,2	6,89	RNA metabolism OR transcription;other metabolic processes;other biological proc	nucleus;other cell component
A0A338P6K3	Zinc finger protein 148 OS=Mus musculus OX=10090 GN=Zfp148 PE=1 SV=1	7	3	4	3	752	83,7	6,13		
E0CXE7	CST complex subunit CTC1 OS=Mus musculus OX=10090 GN=Ctc1 PE=1 SV=	6	5	5	5	965	106,4	8,28		nucleus;other cell component
Q91WF7	Polyphosphoinositide phosphatase OS=Mus musculus OX=10090 GN=Fig4 P	3	3	3	3	907	103,4	6,98	cell organization and biogenesis;other metabolic processes;developmental proce	other membranes;ER/Golgi;other cytoplasmic organelle;other cell component
Q8BK72	28S ribosomal protein S27, mitochondrial OS=Mus musculus OX=10090 GN=	36	13	21	13	415	47,7	5,5	cell cycle OR cell proliferation;other biological processes	mitochondrion;translational apparatus;nucleus;other cell component
Q7TMY8	E3 ubiquitin-protein ligase HUWE1 OS=Mus musculus OX=10090 GN=Huwe1	18	69	100	44	4377	482,3	5,22	cell organization and biogenesis;protein metabolism;DNA metabolism;other meta	other membranes;cytosol;ER/Golgi;nucleus;other cell component
P32883	GTPase KRas OS=Mus musculus OX=10090 GN=Kras PE=1 SV=1	16	3	9	2	189	21,6	6,77	cell-cell signaling;cell organization and biogenesis;stress response;developmenta	plasma membrane;other membranes;mitochondrion;other cell component
Q06185	ATP synthase subunit e, mitochondrial OS=Mus musculus OX=10090 GN=Atp	42	4	12	3	71	8,2	9,35	other metabolic processes;transport	other membranes;mitochondrion
B2RQ57	Tyrosine-protein kinase OS=Mus musculus OX=10090 GN=Abl2 PE=1 SV=1	3	2	3	2	1078	117,7	7,66	protein metabolism;other metabolic processes	
E9QKG3	tRNA (guanine(10)-N2)-methyltransferase homolog OS=Mus musculus OX=1	19	9	11	9	460	52,9	8,46	other metabolic processes	
Q91YK2	Ribosomal RNA processing protein 1 homolog B OS=Mus musculus OX=1009	22	12	14	12	724	80,5	9,72	RNA metabolism OR transcription;other metabolic processes;other biological proc	nucleus;other cell component
Q71FD7	Filamin-binding LIM protein 1 OS=Mus musculus OX=10090 GN=Fblim1 PE=	56	15	43	14	375	41	6,51	cell adhesion;other biological processes	cytoskeleton;nucleus;other cell component
Q7TSI3	Serine/threonine-protein phosphatase 6 regulatory subunit 1 OS=Mus muscu	13	9	10	8	856	94,5	4,64	other biological processes	cytosol;nucleus
O35343	Importin subunit alpha-3 OS=Mus musculus OX=10090 GN=Kpna4 PE=1 SV=	38	13	36	6	521	57,9	4,94	stress response;transport	other membranes;nucleus;other cell component
A0JNY3	Molybdopterin molybdenumtransferase OS=Mus musculus OX=10090 GN=	41	27	69	27	772	83,6	5,76	cell organization and biogenesis;protein metabolism;other metabolic processes;o	plasma membrane;other membranes;other cell component
Q7TMG8	Glioblastoma amplified sequence OS=Mus musculus OX=10090 GN=Nipsnap	11	4	5	4	281	32,9	9,33	cell organization and biogenesis;other metabolic processes	other membranes;mitochondrion
E9QND8	Atlastin-2 OS=Mus musculus OX=10090 GN=Atl2 PE=1 SV=1	5	2	2	2	412	46,8	5,95		other membranes
Q8VEG6	CCR4-NOT transcription complex subunit 6-like OS=Mus musculus OX=10090	6	2	3	2	555	63	6,57	RNA metabolism OR transcription;other metabolic processes;other biological proc	cytosol;nucleus;other cell component
Q8BGB5	LIM domain-containing protein 2 OS=Mus musculus OX=10090 GN=Limd2 PE	59	8	38	7	128	14,2	9,04		cytosol;nucleus
G3X9K3	Brefeldin A-inhibited guanine nucleotide-exchange protein 1 OS=Mus muscul	12	21	28	15	1846	208,4	5,86	cell organization and biogenesis;transport;developmental processes;other biolog	other membranes;cytosol;ER/Golgi;nucleus;other cell component
Q8R1J3	Zinc finger CCHC domain-containing protein 9 OS=Mus musculus OX=10090	34	8	11	8	271	30,5	9,25	other biological processes	nucleus
Q8CCN5	BCAS3 microtubule associated cell migration factor OS=Mus musculus OX=1	9	6	6	6	928	101	6,7	cell organization and biogenesis;stress response;developmental processes;other	cytoskeleton;nucleus;other cell component
P70444	BH3-interacting domain death agonist OS=Mus musculus OX=10090 GN=Bid	49	7	24	7	195	21,9	4,81	cell organization and biogenesis;other metabolic processes;stress response;trans	other membranes;cytosol;mitochondrion
Q921C3	Bromodomain and WD repeat-containing protein 1 OS=Mus musculus OX=1	2	4	4	4	2304	259,1	8,4	cell organization and biogenesis;other biological processes	cytosol;nucleus
A0A0R4J0R1	Vesicle-associated membrane protein 8 OS=Mus musculus OX=10090 GN=V	50	5	6	5	101	11,4	8,19	cell organization and biogenesis;other metabolic processes;transport;other biolog	plasma membrane;other membranes;cytosol;other cytoplasmic organelle;other cell component
B1AWT7	Ubiquitin-conjugating enzyme E2 J1 (Fragment) OS=Mus musculus OX=1009	15	2	3	2	157	16,8	5,97		

[illegible]

Q3UD01	Ataxin-7-like protein 3B OS=Mus musculus OX=10090 GN=Atxn7l3b PE=1 SV	44	3	10	3	97	10,7	4,32	other biological processes	other cell component
Q3U2A8	Valine--tRNA ligase, mitochondrial OS=Mus musculus OX=10090 GN=Vars2	20	17	27	17	1060	118,4	7,14	protein metabolism;RNA metabolism OR transcription;other metabolic processes	cytosol
Q6P9P6	Kinesin-like protein KIF11 OS=Mus musculus OX=10090 GN=Kif11 PE=1 SV=	9	9	9	9	1052	118	6	cell cycle OR cell proliferation;cell organization and biogenesis;other biological pro	cytosol;cytoskeleton;nucleus;other cell com
P48377	MHC class II regulatory factor RFX1 OS=Mus musculus OX=10090 GN=Rfx1 P	2	2	2	2	963	103,6	6,34	other biological processes	nucleus;other cell component
P36916	Guanine nucleotide-binding protein-like 1 OS=Mus musculus OX=10090 GN=	16	9	13	9	607	68,7	5,68	stress response	
P12787	Cytochrome c oxidase subunit 5A, mitochondrial OS=Mus musculus OX=100	21	3	10	3	146	16,1	6,54	other metabolic processes;transport	other membranes;mitochondrion
P63094	Guanine nucleotide-binding protein G(s) subunit alpha isoforms short OS=Mu	34	12	21	11	394	45,6	5,96	cell adhesion;cell organization and biogenesis;DNA metabolism;other metabolic p	plasma membrane;other membranes;cytos
Q8BP71	RNA binding protein fox-1 homolog 2 OS=Mus musculus OX=10090 GN=Rbfo	18	6	6	6	449	47,3	6,55	cell organization and biogenesis;RNA metabolism OR transcription;other metaboli	cytosol;nucleus;other cell component
Q61656	Probable ATP-dependent RNA helicase DDX5 OS=Mus musculus OX=10090	41	25	53	18	614	69,2	8,92	RNA metabolism OR transcription;other metabolic processes;transport;developm	nucleus;other cell component
Q3TRM8	Hexokinase-3 OS=Mus musculus OX=10090 GN=Hk3 PE=1 SV=2	22	18	32	17	922	100	5,94	other metabolic processes;other biological processes	cytosol;other cell component
Q9R1A8	E3 ubiquitin-protein ligase COP1 OS=Mus musculus OX=10090 GN=Cop1 PE=	3	2	2	2	733	80,4	6,92	protein metabolism;other metabolic processes;other biological processes	other membranes;ER/Golgi;nucleus;other c
P48774	Glutathione S-transferase Mu 5 OS=Mus musculus OX=10090 GN=Gstm5 PE	43	10	23	9	224	26,6	7,21	other metabolic processes;stress response;other biological processes	non-structural extracellular;cytosol;cytoske
Q9EQI8	39S ribosomal protein L46, mitochondrial OS=Mus musculus OX=10090 GN=	22	5	11	5	283	32,1	7,4		mitochondrion;translational apparatus;nucl
Q8BVA5	Lipid droplet-associated hydrolase OS=Mus musculus OX=10090 GN=Ldah P	14	4	6	4	326	37,3	8,28	other biological processes	ER/Golgi;other cell component
Q3TFK5	G patch domain-containing protein 4 OS=Mus musculus OX=10090 GN=Gpat	27	11	15	11	415	46,5	9,39	developmental processes	
Q3TXS7	26S proteasome non-ATPase regulatory subunit 1 OS=Mus musculus OX=10	41	34	107	34	953	105,7	5,39	protein metabolism;other metabolic processes;other biological processes	nucleus;other cell component
Q63829	COMM domain-containing protein 3 OS=Mus musculus OX=10090 GN=Com	33	6	8	6	195	22	5,59	transport	nucleus;other cell component
Q64674	Spermidine synthase OS=Mus musculus OX=10090 GN=Srm PE=1 SV=1	79	17	172	17	302	34	5,5	other metabolic processes;other biological processes	
Q9WVH4	Forkhead box protein O3 OS=Mus musculus OX=10090 GN=Foxo3 PE=1 SV=	6	3	3	3	672	71	5,12	RNA metabolism OR transcription;other metabolic processes;stress response;dev	other membranes;cytosol;mitochondrion;n
Q3URQ7	Methenyltetrahydrofolate synthase domain-containing protein OS=Mus mu	21	9	13	3	372	40,8	9,69		other cell component
P53986	Monocarboxylate transporter 1 OS=Mus musculus OX=10090 GN=Slc16a1 P	11	4	8	4	493	53,2	7,47	cell cycle OR cell proliferation;cell organization and biogenesis;other metabolic pro	plasma membrane;other membranes;cytos
Q3V1T4	Prolyl 3-hydroxylase 1 OS=Mus musculus OX=10090 GN=P3h1 PE=1 SV=2	9	6	9	6	739	83,6	5,14	cell organization and biogenesis;protein metabolism;other metabolic processes;d	non-structural extracellular;extracellular ma
Q9JK23	Proteasome assembly chaperone 1 OS=Mus musculus OX=10090 GN=Psmg	40	12	39	12	289	33,1	6,37	cell cycle OR cell proliferation;cell organization and biogenesis;developmental pro	cytosol;ER/Golgi;nucleus;other cell compon
A0A6I8MWX8	DnaJ homolog subfamily C member 7 OS=Mus musculus OX=10090 GN=Dna	39	21	45	21	579	65,4	8,15	other biological processes	cytosol;nucleus
Q64727	Vinculin OS=Mus musculus OX=10090 GN=Vcl PE=1 SV=4	62	64	146	64	1066	116,6	6	cell adhesion;cell organization and biogenesis;developmental processes;other bio	plasma membrane;other membranes;cytos
E9PX53	Serine/threonine-protein phosphatase 4 regulatory subunit 1 OS=Mus muscu	14	10	11	10	934	104,4	4,86	signal transduction	other cell component
Q9DB34	Charged multivesicular body protein 2a OS=Mus musculus OX=10090 GN=Ch	33	9	26	9	222	25,1	5,97	cell cycle OR cell proliferation;cell organization and biogenesis;transport;other bio	other membranes;cytosol;nucleus;other cy
G5E8F4	Fucose-1-phosphate guanylyltransferase OS=Mus musculus OX=10090 GN=	3	2	2	2	590	65,3	6,48		other cell component
Q9CQK1	Zinc finger HIT domain-containing protein 3 OS=Mus musculus OX=10090 GN	52	7	11	7	151	17,1	6,02	cell organization and biogenesis;RNA metabolism OR transcription;other metaboli	nucleus;other cell component

[illegible]

B1AU76	Nuclear autoantigenic sperm protein OS=Mus musculus OX=10090 GN=Nasp	78	26	242	2	448	48,7	4,45		nucleus
Q8R1Q8	Cytoplasmic dynein 1 light intermediate chain 1 OS=Mus musculus OX=1009	43	19	35	19	523	56,6	6,42	cell cycle OR cell proliferation;cell organization and biogenesis;other biological pro	cytoskeleton;other cell component
Q05D44	Eukaryotic translation initiation factor 5B OS=Mus musculus OX=10090 GN=E	31	30	51	30	1216	137,5	5,59	protein metabolism;other metabolic processes;other biological processes	other cell component
Q8C0C7	Phenylalanine--tRNA ligase alpha subunit OS=Mus musculus OX=10090 GN=	43	20	38	20	508	57,6	8,28	cell organization and biogenesis;protein metabolism;RNA metabolism OR transcri	cytosol;other cell component
A0A0R4J140	Clustered mitochondria protein homolog OS=Mus musculus OX=10090 GN=C	53	58	170	58	1353	151,7	6,02	cell organization and biogenesis	other cell component
Q9CQU5	ZW10 interactor OS=Mus musculus OX=10090 GN=Zwint PE=1 SV=1	7	2	3	2	252	28,7	8,43	cell cycle OR cell proliferation;cell organization and biogenesis;signal transduction	cytosol;nucleus;other cell component
Q80UN9	tRNA dimethylallyltransferase OS=Mus musculus OX=10090 GN=Trit1 PE=2 S	10	4	4	3	467	52,4	8,21	RNA metabolism OR transcription;other metabolic processes	nucleus
Q8K078	Solute carrier organic anion transporter family member 4A1 OS=Mus muscul	4	2	3	2	723	77,6	7,85	other metabolic processes;transport	plasma membrane;other membranes
Q9CW03	Structural maintenance of chromosomes protein 3 OS=Mus musculus OX=10	33	40	67	40	1217	141,5	7,18	cell cycle OR cell proliferation;cell organization and biogenesis;DNA metabolism;o	extracellular matrix;cytoskeleton;nucleus;other cell component
Q91VU6	DDB1- and CUL4-associated factor 11 OS=Mus musculus OX=10090 GN=Dca	20	8	13	8	549	62	6,44	protein metabolism;other metabolic processes	nucleus;other cell component
Q3U3M5	Cyclin-K OS=Mus musculus OX=10090 GN=Ccnk PE=1 SV=1	13	6	13	6	582	64,3	8,41	protein metabolism;other metabolic processes;stress response;other biological pr	nucleus;other cell component
Q5KU39	Vacuolar protein sorting-associated protein 41 homolog OS=Mus musculus C	7	5	6	5	853	98,5	5,81	cell organization and biogenesis;other metabolic processes;stress response;trans	other membranes;cytosol;cytoskeleton;ER/Golgi;other cytoplasmi
P30999	Catenin delta-1 OS=Mus musculus OX=10090 GN=Ctnnd1 PE=1 SV=2	3	3	4	3	938	104,9	6,87	cell adhesion;cell-cell signaling;cell organization and biogenesis;developmental p	plasma membrane;other membranes;cytosol;nucleus;other cell co
Q8JZM7	Parafibromin OS=Mus musculus OX=10090 GN=Cdc73 PE=1 SV=1	36	18	23	18	531	60,5	9,61	cell-cell signaling;cell cycle OR cell proliferation;cell organization and biogenesis;p	cytosol;nucleus
Q6NZB1	Protein arginine N-methyltransferase 6 OS=Mus musculus OX=10090 GN=Pr	24	6	8	6	378	41,8	5,34	protein metabolism;DNA metabolism;other metabolic processes;stress response;	nucleus
P40124	Adenylyl cyclase-associated protein 1 OS=Mus musculus OX=10090 GN=Cap	61	22	115	22	474	51,5	7,52	cell organization and biogenesis;transport;signal transduction;other biological pro	non-structural extracellular;plasma membrane;other membranes;
Q3UKU1	RNA polymerase II elongation factor ELL2 OS=Mus musculus OX=10090 GN=	3	2	2	2	639	72,1	8,81	RNA metabolism OR transcription;other metabolic processes;other biological proc	nucleus
Q9Z1F9	SUMO-activating enzyme subunit 2 OS=Mus musculus OX=10090 GN=Uba2	50	26	77	26	638	70,5	5,24	protein metabolism;other metabolic processes;other biological processes	nucleus;other cell component
G3X8Y3	N-alpha-acetyltransferase 15, NatA auxiliary subunit OS=Mus musculus OX=	45	43	147	38	865	101	7,52	protein metabolism;other metabolic processes;other biological processes	cytosol;nucleus;other cell component
P49138	MAP kinase-activated protein kinase 2 OS=Mus musculus OX=10090 GN=M	39	16	31	12	386	44	8,57	protein metabolism;other metabolic processes;stress response;transport;develop	cytoskeleton;nucleus;other cell component
Q9D2N9	Vacuolar protein sorting-associated protein 33A OS=Mus musculus OX=1009	20	11	11	10	598	67,5	7,08	cell organization and biogenesis;other metabolic processes;transport;developme	other membranes;other cytoplasmic organelle;other cell compone
Q9CQT1	Methylthioribose-1-phosphate isomerase OS=Mus musculus OX=10090 GN=	62	17	58	17	369	39,4	5,91	other metabolic processes	cytosol;nucleus
Q6PDI5	Proteasome adapter and scaffold protein ECM29 OS=Mus musculus OX=100	10	17	24	17	1840	203,6	7,06	cell organization and biogenesis;protein metabolism;other metabolic processes;s	cytoskeleton;ER/Golgi;nucleus;other cytoplasmic organelle;other c
Q5SW15	U6 small nuclear RNA (adenine-(43)-N(6))-methyltransferase OS=Mus muscu	26	14	28	14	593	66,8	7,69	RNA metabolism OR transcription;other metabolic processes;other biological proc	nucleus;other cell component
P57759	Endoplasmic reticulum resident protein 29 OS=Mus musculus OX=10090 GN=	58	13	175	13	262	28,8	6,15	transport;other biological processes	ER/Golgi;other cell component
P61759	Prefoldin subunit 3 OS=Mus musculus OX=10090 GN=Vbp1 PE=1 SV=2	64	14	64	13	196	22,4	6,28	cell organization and biogenesis;other biological processes	cytosol;nucleus;other cell component
O88895	Histone deacetylase 3 OS=Mus musculus OX=10090 GN=Hdac3 PE=1 SV=1	13	5	6	4	424	48,3	5,26	cell cycle OR cell proliferation;cell organization and biogenesis;protein metabolism	plasma membrane;other membranes;cytosol;cytoskeleton;ER/Go
Q8C754	Vacuolar protein sorting-associated protein 52 homolog OS=Mus musculus C	6	4	6	4	723	82	5,9	transport;developmental processes;other biological processes	other membranes;cytosol;ER/Golgi;other cytoplasmic organelle;ot
Q8VI75	Importin-4 OS=Mus musculus OX=10090 GN=Ipo4 PE=1 SV=1	20	20	31	20	1082	119,2	5,03	cell organization and biogenesis;transport;other biological processes	nucleus;other cell component
Q80Y44	Probable ATP-dependent RNA helicase DDX10 OS=Mus musculus OX=10090	5	2	2	2	875	100,7	7,75	RNA metabolism OR transcription;other metabolic processes;other biological proc	nucleus
P70290	55 kDa erythrocyte membrane protein OS=Mus musculus OX=10090 GN=M	12	5	7	5	466	52,2	7,2	other biological processes	plasma membrane;other membranes;cytoskeleton;other cell comp
Q9Z1K5	E3 ubiquitin-protein ligase ARIH1 OS=Mus musculus OX=10090 GN=Arih1 PE	31	13	22	13	555	64	5,08	protein metabolism;other metabolic processes;other biological processes	nucleus;other cell component
Q8CI94	Glycogen phosphorylase, brain form OS=Mus musculus OX=10090 GN=Pygb	43	31	99	31	843	96,7	6,73	other metabolic processes	other cell component
Q6A028	Switch-associated protein 70 OS=Mus musculus OX=10090 GN=Swap70 PE=	40	24	63	24	585	69	6,05	cell organization and biogenesis;DNA metabolism;other metabolic processes;dev	plasma membrane;other membranes;cytoskeleton;nucleus;other
Q91WM2	Haloacid dehalogenase-like hydrolase domain-containing 5 OS=Mus muscul	11	4	6	4	419	46,3	7,88	other metabolic processes	
Q8QZV7	Integrator complex subunit 13 OS=Mus musculus OX=10090 GN=Ints13 PE=	6	5	6	3	732	82,7	6,92	cell cycle OR cell proliferation;cell organization and biogenesis;other biological pro	nucleus;other cell component
E0CX53	TBC1 domain family member 4 OS=Mus musculus OX=10090 GN=Tbc1d4 PE	5	4	4	4	1306	147,2	7,39	transport;other biological processes	cytosol;other cell component
Q9CQ21	Malignant T-cell-amplified sequence 2 OS=Mus musculus OX=10090 GN=Mc	51	7	19	4	181	20,4	9,11	cell organization and biogenesis;protein metabolism;other metabolic processes	other cell component
Q8BHK9	DNA excision repair protein ERCC-6-like OS=Mus musculus OX=10090 GN=Er	4	4	5	4	1240	138,8	5,21	cell cycle OR cell proliferation;cell organization and biogenesis;other biological pro	other cell component
A0A0R4J049	Protein arginine N-methyltransferase 5 OS=Mus musculus OX=10090 GN=Pr	49	27	92	27	637	72,7	6,34	cell organization and biogenesis;protein metabolism;RNA metabolism OR transcri	cytosol;ER/Golgi;nucleus;other cell component
Q9D7A8	Armadillo repeat-containing protein 1 OS=Mus musculus OX=10090 GN=Arm	25	6	10	6	282	31,2	5,57	cell organization and biogenesis	other membranes;cytosol;mitochondrion
Q8CI43	Myosin light chain 6B OS=Mus musculus OX=10090 GN=Myl6b PE=1 SV=1	23	4	13	3	207	22,7	5,53	developmental processes;other biological processes	cytoskeleton
Q9EPL8	Importin-7 OS=Mus musculus OX=10090 GN=Ipo7 PE=1 SV=2	20	17	47	17	1038	119,4	4,82	stress response;transport	cytosol;nucleus
Q8R1S0	Ubiquinone biosynthesis monooxygenase COQ6, mitochondrial OS=Mus mu	16	7	8	7	476	51,4	7,17	other metabolic processes	other membranes;mitochondrion;ER/Golgi;other cell component
Q9D0J4	ADP-ribosylation factor-like protein 2 OS=Mus musculus OX=10090 GN=Arl2	29	4	8	4	184	20,9	5,96	cell cycle OR cell proliferation;cell organization and biogenesis;transport;other bio	plasma membrane;other membranes;cytosol;cytoskeleton;mitoch
Q8C0Q2	Zinc fingers and homeoboxes protein 3 OS=Mus musculus OX=10090 GN=Zh	9	5	5	5	951	104,3	6,57	other biological processes	nucleus;other cell component
D3Z5B1	Zinc finger, BED type-containing 5 (Fragment) OS=Mus musculus OX=10090	26	3	12	3	183	19,2	8,28		
Q7TQK5	Coiled-coil domain-containing protein 93 OS=Mus musculus OX=10090 GN=C	22	14	21	14	629	72,6	8,29	transport	other cytoplasmic organelle;other cell component
B9EJ54	Nucleoporin 205 OS=Mus musculus OX=10090 GN=Nup205 PE=1 SV=1	21	37	57	37	2008	227,3	6,34	cell organization and biogenesis;transport	other membranes;nucleus
Q8K3A9	7SK snRNA methylphosphate capping enzyme OS=Mus musculus OX=10090	21	10	14	10	666	72	9,25	RNA metabolism OR transcription;other metabolic processes;other biological proc	nucleus;other cell component
Q3TXV4	Rab22B OS=Mus musculus OX=10090 GN=Rab31 PE=1 SV=1	21	3	4	2	195	21,4	7,4	cell organization and biogenesis;other metabolic processes;transport;other biolog	other membranes;ER/Golgi;other cytoplasmic organelle;other cell
B1ARD6	Schlafen family member 9 OS=Mus musculus OX=10090 GN=Slfn9 PE=2 SV=	21	17	21	16	910	103,9	7,3	RNA metabolism OR transcription;other metabolic processes;stress response	nucleus;other cell component
E9QPI5	Sister chromatid cohesion protein PDS5 homolog A OS=Mus musculus OX=1	18	19	26	17	1332	150,1	7,85	cell cycle OR cell proliferation;cell organization and biogenesis;other biological pro	plasma membrane;other membranes;nucleus;other cell componen
S4R248	Rho GTPase-activating protein 12 OS=Mus musculus OX=10090 GN=Arhgap	8	4	4	4	791	90	8,41	signal transduction	
Q60876	Eukaryotic translation initiation factor 4E-binding protein 1 OS=Mus muscul	75	5	44	5	117	12,3	5,15	cell cycle OR cell proliferation;protein metabolism;other metabolic processes;stre	cytosol;nucleus;other cell component
P35991	Tyrosine-protein kinase BTK OS=Mus musculus OX=10090 GN=Btk PE=1 SV=	21	13	16	13	659	76,4	7,9	cell-cell signaling;protein metabolism;other metabolic processes;stress response;	plasma membrane;other membranes;cytosol;nucleus;other cell co
Q5SQY2	Biorientation of chromosomes in cell division protein 1 OS=Mus musculus OX	51	7	10	6	173	18,4	5,9	cell cycle OR cell proliferation;cell organization and biogenesis;other biological pro	cytoskeleton;other cell component
Q80ZQ9	Protein Abitram OS=Mus musculus OX=10090 GN=Abitram PE=1 SV=2	52	7	9	7	193	21,6	7,25	cell organization and biogenesis;developmental processes;other biological proces	nucleus;other cell component
A0A0R4J1R7	4a-hydroxytetrahydrobiopterin dehydratase OS=Mus musculus OX=10090 G	82	9	18	9	103	11,7	6,79	other metabolic processes	
P61924	Coatomer subunit zeta-1 OS=Mus musculus OX=10090 GN=Copz1 PE=1 SV=	23	3	10	3	177	20,2	4,81	transport	other membranes;ER/Golgi
Q9CQZ1	Heat shock factor-binding protein 1 OS=Mus musculus OX=10090 GN=Hsbp1	49	3	12	3	76	8,6	4,31	stress response;developmental processes;other biological processes	cytosol;cytoskeleton;nucleus
Q9QY76	Vesicle-associated membrane protein-associated protein B OS=Mus muscul	30	7	16	6	243	26,9	7,78	cell organization and biogenesis;stress response;transport;signal transduction;oth	plasma membrane;other membranes;ER/Golgi;other cell compone
Q8JZM0	Dimethyladenosine transferase 1, mitochondrial OS=Mus musculus OX=100	5	2	2	2	345	38,9	9,47	RNA metabolism OR transcription;other metabolic processes	mitochondrion
Q80TL0	Protein phosphatase 1E OS=Mus musculus OX=10090 GN=Ppm1e PE=1 SV=	10	5	8	5	749	83,4	4,97	protein metabolism;other metabolic processes;other biological processes	mitochondrion;nucleus;other cell component
Q8VE42	Ankyrin repeat domain-containing protein 49 OS=Mus musculus OX=10090	32	6	11	6	238	27,1	5,16	other biological processes	nucleus
Q9D8U8	Sorting nexin-5 OS=Mus musculus OX=10090 GN=Snx5 PE=1 SV=1	53	21	55	19	404	46,8	6,62	protein metabolism;other metabolic processes;transport;other biological processe	plasma membrane;other membranes;cytosol;other cytoplasmic o
P62334	26S proteasome regulatory subunit 10B OS=Mus musculus OX=10090 GN=P	66	20	63	20	389	44,1	7,49	protein metabolism;other metabolic processes;stress response;other biological pr	cytosol;nucleus;other cell component
Q8R5A6	TBC1 domain family member 22A OS=Mus musculus OX=10090 GN=Tbc1d2	7	3	3	3	516	59,3	6,46	transport;other biological processes	
P63085	Mitogen-activated protein kinase 1 OS=Mus musculus OX=10090 GN=Mapk	42	13	52	10	358	41,2	6,98	cell cycle OR cell proliferation;protein metabolism;RNA metabolism OR transcripti	plasma membrane;other membranes;cytosol;cytoskeleton;mitoch
Q922B1	ADP-ribose glycohydrolase MACROD1 OS=Mus musculus OX=10090 GN=Ma	13	4	5	4	323	35,3	8,85	protein metabolism;other metabolic processes;stress response	nucleus
P43247	DNA mismatch repair protein Msh2 OS=Mus musculus OX=10090 GN=Msh2	26	22	40	22	935	104,1	5,96	cell cycle OR cell proliferation;cell organization and biogenesis;DNA metabolism;o	nucleus;other cell component
Q8R1G6	PDZ and LIM domain protein 2 OS=Mus musculus OX=10090 GN=Pdlim2 PE=	26	8	9	8	349	37,7	8,7	cell organization and biogenesis;developmental processes;other biological proces	cytoskeleton;other cell component
Q9Z1G3	V-type proton ATPase subunit C 1 OS=Mus musculus OX=10090 GN=Atp6v1	54	21	76	21	382	43,9	7,46	transport	plasma membrane;other membranes;other cytoplasmic organelle;
E9Q0Y4	Signal-induced proliferation-associated protein 1 OS=Mus musculus OX=100	20	16	20	16	1038	112,2	6,44	stress response;other biological processes	ER/Golgi;other cell component
Q8BH43	Wiskott-Aldrich syndrome protein family member 2 OS=Mus musculus OX=1	19	10	27	10	497	54	5,53	cell organization and biogenesis;transport;developmental processes;signal transd	plasma membrane;other membranes;cytoskeleton;other cytoplas
Q8CJ40	Rootletin OS=Mus musculus OX=10090 GN=Crocc PE=1 SV=2	2	4	4	3	2009	226,8	5,55	cell cycle OR cell proliferation;cell organization and biogenesis;other biological pro	plasma membrane;other membranes;cytosol;cytoskeleton;other
A0A2I3BPG9	Ribosomal protein L36A, pseudogene 1 OS=Mus musculus OX=10090 GN=Rp	25	4	31	4	106	12,4	10,51	protein metabolism;other metabolic processes	cytosol;translational apparatus
Q5EBG8	Uncharacterized protein C1orf50 homolog OS=Mus musculus OX=10090 GN=	31	5	8	5	199	21,8	5,53		
P17427	AP-2 complex subunit alpha-2 OS=Mus musculus OX=10090 GN=Ap2a2 PE=	24	21	36	13	938	104	6,93	transport;other biological processes	plasma membrane;other membranes;ER/Golgi;other cell compone
Q8R480	Nuclear pore complex protein Nup85 OS=Mus musculus OX=10090 GN=Nup	27	15	31	15	656	74,7	5,57	cell organization and biogenesis;other metabolic processes;transport;developme	plasma membrane;other membranes;cytosol;cytoskeleton;nucleu
Q8CFI5	Probable proline--tRNA ligase, mitochondrial OS=Mus musculus OX=10090 G	18	6	10	6	475	53,5	7,84	protein metabolism;RNA metabolism OR transcription;other metabolic processes	mitochondrion
Q8R3N6	THO complex subunit 1 OS=Mus musculus OX=10090 GN=Thoc1 PE=1 SV=1	11	6	8	6	657	75,4	4,97	DNA metabolism;RNA metabolism OR transcription;other metabolic processes;tra	nucleus;other cell component
Q8BZM1	Glomulin OS=Mus musculus OX=10090 GN=Glmn PE=1 SV=1	11	5	7	5	596	67,7	5,06	cell organization and biogenesis;developmental processes;signal transduction;oth	nucleus;other cell component
Q9D1H8	39S ribosomal protein L53, mitochondrial OS=Mus musculus OX=10090 GN=	58	6	15	6	118	12,7	9,52		mitochondrion;translational apparatus
Q4VA98	Zfp219 protein OS=Mus musculus OX=10090 GN=Zfp219 PE=1 SV=1	6	3	3	3	681	73,2	9,33		
Q920Q8	Influenza virus NS1A-binding protein homolog OS=Mus musculus OX=10090	27	15	23	15	642	71,5	5,47	RNA metabolism OR transcription;other metabolic processes;other biological proc	cytosol;cytoskeleton;nucleus
Q9ERU9	E3 SUMO-protein ligase RanBP2 OS=Mus musculus OX=10090 GN=Ranbp2 F	9	23	33	22	3053	340,9	6,18	protein metabolism;other metabolic processes;transport;other biological processe	other membranes;mitochondrion;nucleus;other cell component
Q99MR6	Serrate RNA effector molecule homolog OS=Mus musculus OX=10090 GN=S	26	24	46	24	875	100,4	5,97	RNA metabolism OR transcription;other metabolic processes;developmental proc	nucleus;other cell component
F6ZBR8	Protein Wiz OS=Mus musculus OX=10090 GN=Wiz PE=1 SV=3	9	7	10	7	1059	112,6	9,45		
Q9CQ28	Diphthine--ammonia ligase OS=Mus musculus OX=10090 GN=Dph6 PE=1 SV	12	3	3	3	267	29,9	5,16	protein metabolism;other metabolic processes	nucleus
Q8R2R3	Alpha- and gamma-adaptin-binding protein p34 OS=Mus musculus OX=1009	34	7	7	7	316	34,5	4,64	transport	cytosol;nucleus;other cell component
Q9CY94	DNA replication complex GINS protein PSF3 OS=Mus musculus OX=10090 GN	58	9	11	9	216	24,6	5,64	cell cycle OR cell proliferation;DNA metabolism;other metabolic processes	nucleus
Q9D3U0	tRNA pseudouridine synthase Pus10 OS=Mus musculus OX=10090 GN=Pus1	25	11	17	11	527	59,7	6,49	RNA metabolism OR transcription;other metabolic processes	mitochondrion;nucleus;other cell component
Q9JLV5	Cullin-3 OS=Mus musculus OX=10090 GN=Cul3 PE=1 SV=1	35	26	50	25	768	88,9	8,46	cell-cell signaling;cell cycle OR cell proliferation;cell organization and biogenesis;p	plasma membrane;other membranes;cytoskeleton;ER/Golgi;nucle
Q6A068	Cell division cycle 5-like protein OS=Mus musculus OX=10090 GN=Cdc5l PE=	21	11	18	11	802	92,1	8,02	cell cycle OR cell proliferation;DNA metabolism;RNA metabolism OR transcription	nucleus;other cell component
Q61216	Double-strand break repair protein MRE11 OS=Mus musculus OX=10090 GN	27	16	24	16	706	80,2	6,01	cell cycle OR cell proliferation;cell organization and biogenesis;DNA metabolism;c	nucleus;other cell component
Q99LS3	Phosphoserine phosphatase OS=Mus musculus OX=10090 GN=Psph PE=1 SV	45	10	34	10	225	25,1	6,14	other metabolic processes;developmental processes;other biological processes	cytosol;other cell component
Q8BM39	Pre-mRNA-splicing factor 18 OS=Mus musculus OX=10090 GN=Prpf18 PE=1	8	3	4	3	342	39,9	8,15	cell organization and biogenesis;RNA metabolism OR transcription;other metaboli	other membranes;nucleus
Q99KY4	Cyclin-G-associated kinase OS=Mus musculus OX=10090 GN=Gak PE=1 SV=	6	6	7	6	1305	143,6	5,73	cell cycle OR cell proliferation;cell organization and biogenesis;protein metabolism	cytosol;ER/Golgi;other cell component
Q9CQ40	39S ribosomal protein L49, mitochondrial OS=Mus musculus OX=10090 GN=	56	7	17	5	166	19,1	9,5	protein metabolism;other metabolic processes	mitochondrion;translational apparatus
Q9D600	DNA replication complex GINS protein PSF2 OS=Mus musculus OX=10090 GN	36	6	9	6	185	21,2	5,29	DNA metabolism;other metabolic processes;stress response	nucleus
Q91VI7	Ribonuclease inhibitor OS=Mus musculus OX=10090 GN=Rnh1 PE=1 SV=1	61	20	85	20	456	49,8	4,78	other biological processes	non-structural extracellular;cytosol;nucleus
Q8BMA6	Signal recognition particle subunit SRP68 OS=Mus musculus OX=10090 GN=S	19	12	18	12	625	70,5	8,57	transport;other biological processes	cytosol;translational apparatus;nucleus;other cell component
E9QAT4	Protein transport protein Sec16A OS=Mus musculus OX=10090 GN=Sec16a F	15	23	35	23	2357	254	5,81	cell organization and biogenesis;other metabolic processes;stress response;trans	other membranes;cytosol;ER/Golgi;other cell component
Q9D1J1	Adaptin ear-binding coat-associated protein 2 OS=Mus musculus OX=10090	61	12	30	12	266	28,6	7,94	transport	plasma membrane;other membranes
Q9QXN3	Activating signal cointegrator 1 OS=Mus musculus OX=10090 GN=Trip4 PE=1	6	2	2	2	581	66,2	7,55	transport;signal transduction;other biological processes	cytosol;cytoskeleton;nucleus;other cell component
E9QAF9	Protein TANC1 OS=Mus musculus OX=10090 GN=Tanc1 PE=1 SV=1	1	2	3	2	1849	199,9	8,32		
Q99KP6	Pre-mRNA-processing factor 19 OS=Mus musculus OX=10090 GN=Prpf19 PE	48	15	60	15	504	55,2	6,61	cell organization and biogenesis;protein metabolism;DNA metabolism;RNA meta	cytoskeleton;nucleus;other cell component
Q3UVG3	Protein FAM91A1 OS=Mus musculus OX=10090 GN=Fam91a1 PE=1 SV=1	5	4	4	4	837	93,4	6,35	cell organization and biogenesis;transport	ER/Golgi;other cell component
E9QMH1	DNA dC->dU-editing enzyme APOBEC-3 OS=Mus musculus OX=10090 GN=A	10	3	3	3	429	51	8,97		
P14685	26S proteasome non-ATPase regulatory subunit 3 OS=Mus musculus OX=10	55	29	100	29	530	60,7	8,44	protein metabolism;other metabolic processes;other biological processes	other cell component
A2AJI1	MAP7 domain-containing protein 1 OS=Mus musculus OX=10090 GN=Map7	23	14	26	14	774	85,7	9,95	cell organization and biogenesis	cytoskeleton
P70662	LIM domain-binding protein 1 OS=Mus musculus OX=10090 GN=Ldb1 PE=1 S	15	5	12	5	411	46,5	6,96	cell-cell signaling;cell organization and biogenesis;protein metabolism;RNA metal	nucleus;other cell component
P97310	DNA replication licensing factor MCM2 OS=Mus musculus OX=10090 GN=M	57	41	119	41	904	102	5,72	cell cycle OR cell proliferation;cell organization and biogenesis;DNA metabolism;c	nucleus;other cell component
Q8CGK3	Lon protease homolog, mitochondrial OS=Mus musculus OX=10090 GN=Lon	32	26	71	26	949	105,8	6,57	cell organization and biogenesis;protein metabolism;other metabolic processes;s	cytosol;mitochondrion;nucleus
Q91ZR1	Ras-related protein Rab-4B OS=Mus musculus OX=10090 GN=Rab4b PE=1 S	19	4	11	3	213	23,6	6,06	transport;signal transduction;other biological processes	plasma membrane;other membranes;ER/Golgi;other cytoplasmic
B7ZMQ0	Mitochondrial mRNA pseudouridine synthase Rpusd3 OS=Mus musculus OX	10	2	2	2	279	30	8,84	RNA metabolism OR transcription;other metabolic processes	

Q6DFW0	Guanine nucleotide exchange C9orf72 homolog OS=Mus musculus OX=1009	4	2	2	2	481	54,2	6,09	cell organization and biogenesis;other metabolic processes;transport;developme	non-structural extracellular;other membranes;cytosol;nucleus;other cytoplasmic organelle;other ce	enzyme regulator activity;other molecular function	Pf15019
P28867	Protein kinase C delta type OS=Mus musculus OX=10090 GN=Prkcd PE=1 SV	25	17	21	16	674	77,5	7,39	cell cycle OR cell proliferation;cell organization and biogenesis;protein metabolism	plasma membrane;other membranes;cytosol;mitochondrion;ER/Golgi;nucleus;other cytoplasmic o	enzyme regulator activity;kinase activity;other molecular function	Pf00069, Pf00130,
F6UY95	Mastermind-like protein 1 (Fragment) OS=Mus musculus OX=10090 GN=Ma	6	3	5	3	585	61,8	6,14				
Q9WVS8	Mitogen-activated protein kinase 7 OS=Mus musculus OX=10090 GN=Mapk	4	2	2	2	806	87,7	5,76	cell cycle OR cell proliferation;protein metabolism;other metabolic processes;stre	cytosol;nucleus;other cell component	kinase activity;other molecular function	Pf00069
Q9CRC6	BLOC-1-related complex subunit 7 OS=Mus musculus OX=10090 GN=Borcs7	42	3	5	3	105	11,5	6,79		other membranes;cytosol;other cytoplasmic organelle		Pf16088
P51807	Dynein light chain Tctex-type 1 OS=Mus musculus OX=10090 GN=Dynlt1 PE=	64	4	14	4	113	12,5	5,08	cell cycle OR cell proliferation;cell organization and biogenesis;developmental pro	cytoskeleton;ER/Golgi;other cell component	other molecular function	Pf03645
P12382	ATP-dependent 6-phosphofructokinase, liver type OS=Mus musculus OX=10	33	26	55	20	780	85,3	7,17	other metabolic processes;other biological processes	other membranes;cytosol	kinase activity;other molecular function	Pf00365
Q6NVE9	Protein phosphatase PTC7 homolog OS=Mus musculus OX=10090 GN=Pptc7	12	3	3	3	310	33	5,27	protein metabolism;other metabolic processes;other biological processes	mitochondrion	other molecular function	Pf07228
Q80X50	Ubiquitin-associated protein 2-like OS=Mus musculus OX=10090 GN=Ubap2l	34	27	132	6	1107	116,7	7,11	cell organization and biogenesis;other biological processes	nucleus;other cell component		Pf12478
P62077	Mitochondrial import inner membrane translocase subunit Tim8 B OS=Mus m	60	5	20	5	83	9,3	5,12	transport	other membranes;mitochondrion	other molecular function	Pf02953
Q6PEE2	CBP80/20-dependent translation initiation factor OS=Mus musculus OX=100	11	6	7	6	600	67,8	6,65	RNA metabolism OR transcription;other metabolic processes;other biological proc	cytosol;other cell component	nucleic acid binding activity	Pf02854
Q80WQ2	Protein VAC14 homolog OS=Mus musculus OX=10090 GN=Vac14 PE=1 SV=1	42	26	47	26	782	88	6,13	other metabolic processes;stress response;other biological processes	other membranes;cytosol;ER/Golgi;other cytoplasmic organelle;other cell component	enzyme regulator activity;other molecular function	Pf11916, Pf12755
P59048	p53 and DNA damage-regulated protein 1 OS=Mus musculus OX=10090 GN=	27	4	6	4	133	15,4	8,1	other biological processes	other cell component	other molecular function	Pf01920
P60330	Separin OS=Mus musculus OX=10090 GN=Espl1 PE=1 SV=1	4	6	7	6	2118	232,9	6,9	cell cycle OR cell proliferation;cell organization and biogenesis;protein metabolism	cytoskeleton;nucleus;other cell component	other molecular function	Pf03568
Q9ERR7	Selenoprotein F OS=Mus musculus OX=10090 GN=Selenof PE=1 SV=3	30	4	5	4	162	17,8	5,35	cell organization and biogenesis;other biological processes	ER/Golgi	other molecular function	Pf08806
Q8BZA9	Fructose-2,6-bisphosphatase TIGAR OS=Mus musculus OX=10090 GN=Tigar	26	4	8	4	269	29,2	8,18	other metabolic processes;stress response;developmental processes;other biolog	other membranes;cytosol;mitochondrion;nucleus;other cell component	other molecular function	Pf00300
A0A0H2UH19	Histone-lysine N-methyltransferase EHMT1 OS=Mus musculus OX=10090 G	10	11	13	11	1289	141,2	5,8	protein metabolism;other metabolic processes	nucleus	other molecular function	Pf00856, Pf05033,
Q3UDT3	Phosphatidylinositol-4,5-bisphosphate 3-kinase OS=Mus musculus OX=1009	7	8	8	8	1044	119,8	7,34	other metabolic processes;signal transduction		kinase activity;other molecular function	Pf00454, Pf00613,
Q921K2	Poly [ADP-ribose] polymerase OS=Mus musculus OX=10090 GN=Parp1 PE=1	29	29	59	29	1014	112,7	8,95	cell organization and biogenesis;protein metabolism;DNA metabolism;other meta	mitochondrion;nucleus;other cell component	nucleic acid binding activity;other molecular function	Pf00533, Pf00644,
A2AMY5	Ubiquitin-associated protein 2 OS=Mus musculus OX=10090 GN=Ubap2 PE=	27	21	75	21	1131	117,8	7,72				Pf12478
Q3TYA6	M-phase phosphoprotein 8 OS=Mus musculus OX=10090 GN=Mphosph8 PE	5	3	4	3	858	97,4	5,85	cell organization and biogenesis;other biological processes	plasma membrane;other membranes;cytosol;nucleus;other cell component	other molecular function	Pf00385, Pf12796,
P68181	cAMP-dependent protein kinase catalytic subunit beta OS=Mus musculus O	20	9	20	4	351	40,7	8,56	protein metabolism;other metabolic processes;developmental processes;signal t	plasma membrane;other membranes;cytosol;cytoskeleton;nucleus;other cell component	kinase activity;other molecular function	Pf00069
O54988	STE20-like serine/threonine-protein kinase OS=Mus musculus OX=10090 GN	47	46	99	45	1233	141,4	5,14	cell organization and biogenesis;protein metabolism;other metabolic processes;o	other cell component	kinase activity;other molecular function	Pf00069, Pf12474
Q8C147	Dedicator of cytokinesis protein 8 OS=Mus musculus OX=10090 GN=Dock8 P	2	4	4	4	2100	238,8	6,96	cell cycle OR cell proliferation;signal transduction;other biological processes	plasma membrane;other membranes;other cell component	enzyme regulator activity	Pf06920, Pf11878,
Q9ES52	Phosphatidylinositol 3,4,5-trisphosphate 5-phosphatase 1 OS=Mus musculu	27	29	42	27	1191	133,5	7,8	other metabolic processes;developmental processes;signal transduction;other bio	plasma membrane;other membranes;cytosol;cytoskeleton	other molecular function	Pf00017, Pf03372
F8WHU5	Sister chromatid cohesion protein PDS5 homolog B OS=Mus musculus OX=10	7	9	12	6	1449	164,7	8,54	cell cycle OR cell proliferation;cell organization and biogenesis;other biological pro	nucleus;other cell component	nucleic acid binding activity	Pf20168
Q923Z3	Protein MTO1 homolog, mitochondrial OS=Mus musculus OX=10090 GN=M	9	4	5	4	669	74,3	8,92	RNA metabolism OR transcription;other metabolic processes	mitochondrion	other molecular function	Pf01134, Pf13932
P45481	Histone lysine acetyltransferase CREBBP OS=Mus musculus OX=10090 GN=C	10	19	28	11	2441	265,3	8,54	cell cycle OR cell proliferation;protein metabolism;other metabolic processes;othe	nucleus;other cell component	signal transduction activity or receptor binding;nucleic acid binding activity;othe	Pf00439, Pf00569,
F7AEH4	40S ribosomal protein S12 OS=Mus musculus OX=10090 GN=Rps12 PE=1 SV	69	9	130	9	137	15,2	8,35	protein metabolism;other metabolic processes	translational apparatus	other molecular function	Pf01248

[illegible]

Q6P9R2	Serine/threonine-protein kinase OSR1 OS=Mus musculus OX=10090 GN=Ox	9	4	4	4	527	58,2	6,43	protein metabolism;other metabolic processes;stress response;signal transductio	cytosol
Q9WU62	Inner centromere protein OS=Mus musculus OX=10090 GN=Incenp PE=1 SV=	5	3	3	2	880	101,1	9,55	cell cycle OR cell proliferation;cell organization and biogenesis;protein metabolism	cytosol;cytoskeleton;nucleus;other cell com
P84078	ADP-ribosylation factor 1 OS=Mus musculus OX=10090 GN=Arf1 PE=1 SV=2	64	10	64	4	181	20,7	6,8	cell cycle OR cell proliferation;cell organization and biogenesis;transport;other bio	plasma membrane;other membranes;cytos
A2AIV2	Protein virilizer homolog OS=Mus musculus OX=10090 GN=Virma PE=1 SV=1	1	3	3	3	1811	201,3	4,96	RNA metabolism OR transcription;other metabolic processes;other biological proc	cytosol;nucleus;other cell component
O55229	Choline/ethanolamine kinase OS=Mus musculus OX=10090 GN=Chkb PE=1 S	25	8	13	8	394	45,1	5,41	other metabolic processes;developmental processes	
Q8C3Y4	Kinetochore-associated protein 1 OS=Mus musculus OX=10090 GN=Kntc1 PE	5	10	12	10	2207	250,2	6	cell cycle OR cell proliferation;signal transduction;other biological processes	plasma membrane;other membranes;cytos
Q8R0G9	Nuclear pore complex protein Nup133 OS=Mus musculus OX=10090 GN=Nup	21	21	31	21	1155	128,5	5,2	cell organization and biogenesis;other metabolic processes;transport;developme	other membranes;nucleus;other cell compo
O55091	Protein IMPACT OS=Mus musculus OX=10090 GN=Impact PE=1 SV=2	13	2	2	2	318	36,3	5,05	cell organization and biogenesis;stress response;developmental processes;other	cytosol;other cell component
Q8C605	ATP-dependent 6-phosphofructokinase OS=Mus musculus OX=10090 GN=Pf	41	30	90	26	784	85,5	6,89	other metabolic processes	other cell component
Q3UE37	Ubiquitin-conjugating enzyme E2 Z OS=Mus musculus OX=10090 GN=Ube2z	15	6	6	6	356	38,3	5,62	protein metabolism;other metabolic processes;other biological processes	cytosol;nucleus
Q9CPT3	N-acylneuraminate-9-phosphatase OS=Mus musculus OX=10090 GN=Nanp	21	5	7	5	248	27,8	6,07	other metabolic processes	
P61290	Proteasome activator complex subunit 3 OS=Mus musculus OX=10090 GN=P	43	11	48	11	254	29,5	5,95	cell cycle OR cell proliferation;other biological processes	cytosol;nucleus;other cell component
Q9WTX2	Interferon-inducible double-stranded RNA-dependent protein kinase activato	6	2	2	2	313	34,3	8,43	protein metabolism;RNA metabolism OR transcription;other metabolic processes;	cytosol;nucleus;other cell component
O35841	Apoptosis inhibitor 5 OS=Mus musculus OX=10090 GN=Api5 PE=1 SV=2	35	17	41	17	504	56,8	5,92	other biological processes	nucleus;other cell component
Q91W86	Vacuolar protein sorting-associated protein 11 homolog OS=Mus musculus C	13	10	10	10	941	107,7	7,01	cell organization and biogenesis;protein metabolism;other metabolic processes;tr	other membranes;cytoskeleton;other cytop
Q505F5	Leucine-rich repeat-containing protein 47 OS=Mus musculus OX=10090 GN=	54	25	55	25	581	63,6	8,1		
Q03958	Prefoldin subunit 6 OS=Mus musculus OX=10090 GN=Pfdn6 PE=1 SV=1	61	11	39	11	127	14,4	8,88	cell organization and biogenesis;other biological processes	nucleus;other cell component
Q5HZI1	Microtubule-associated tumor suppressor 1 homolog OS=Mus musculus OX=	2	2	2	2	1210	134,3	6,86	cell cycle OR cell proliferation;other biological processes	plasma membrane;other membranes;cytos
P62245	40S ribosomal protein S15a OS=Mus musculus OX=10090 GN=Rps15a PE=1	69	9	43	9	130	14,8	10,13	protein metabolism;other metabolic processes;other biological processes	cytosol;translational apparatus;other cell co
Q6PD19	Armadillo-like helical domain-containing protein 3 OS=Mus musculus OX=10	8	5	6	5	689	78,6	6,6	other biological processes	other membranes;cytosol;ER/Golgi
P13439	Uridine 5'-monophosphate synthase OS=Mus musculus OX=10090 GN=Ump	58	24	56	24	481	52,3	6,61	other metabolic processes;transport;developmental processes;other biological pr	nucleus;other cell component
Q9JKC8	AP-3 complex subunit mu-1 OS=Mus musculus OX=10090 GN=Ap3m1 PE=1	19	5	5	5	418	46,9	6,93	transport	other membranes;ER/Golgi;other cell comp
Q99KH8	Serine/threonine-protein kinase 24 OS=Mus musculus OX=10090 GN=Stk24	22	8	15	8	431	47,9	5,43	protein metabolism;other metabolic processes;stress response;signal transductio	other membranes;cytosol;ER/Golgi;nucleus
Q9CPR4	60S ribosomal protein L17 OS=Mus musculus OX=10090 GN=Rpl17 PE=1 SV=	54	9	65	9	184	21,4	10,18	protein metabolism;other metabolic processes;other biological processes	cytosol;cytoskeleton;translational apparatu
A0A0R4J0W6	Leucine-rich repeat-containing protein 40 OS=Mus musculus OX=10090 GN=	10	5	5	5	602	68	6,84		
Q8VID5	ATP-dependent DNA helicase Q5 OS=Mus musculus OX=10090 GN=Recql5 P	1	2	2	2	982	108,2	8,68	cell cycle OR cell proliferation;cell organization and biogenesis;DNA metabolism;c	cytosol;nucleus;other cell component
Q7M6Y3	Phosphatidylinositol-binding clathrin assembly protein OS=Mus musculus OX	12	7	11	7	660	71,5	7,91	cell organization and biogenesis;other metabolic processes;transport;developme	plasma membrane;other membranes;ER/G
Q91YI4	Beta-arrestin-2 OS=Mus musculus OX=10090 GN=Arrb2 PE=1 SV=1	54	18	34	17	410	46,3	7,66	protein metabolism;RNA metabolism OR transcription;other metabolic processes;	plasma membrane;other membranes;cytos
A0A3Q4EII3	COMM domain-containing protein 10 OS=Mus musculus OX=10090 GN=Con	39	5	11	5	150	16,9	5,33		
P99024	Tubulin beta-5 chain OS=Mus musculus OX=10090 GN=Tubb5 PE=1 SV=1	69	22	329	3	444	49,6	4,89	cell cycle OR cell proliferation;cell organization and biogenesis;other biological pro	other membranes;cytosol;cytoskeleton;nuc
Q9D0D4	Probable dimethyladenosine transferase OS=Mus musculus OX=10090 GN=	33	9	11	9	313	35,3	9,99	RNA metabolism OR transcription;other metabolic processes;other biological proc	cytosol;mitochondrion;nucleus
Q91V89	Serine/threonine-protein phosphatase 2A 56 kDa regulatory subunit OS=Mu	22	12	23	10	594	69	7,96	protein metabolism;other metabolic processes;signal transduction;other biologica	cytosol;nucleus;other cell component
P97784	Cryptochrome-1 OS=Mus musculus OX=10090 GN=Cry1 PE=1 SV=1	3	2	2	2	606	68	7,97	protein metabolism;other metabolic processes;stress response;other biological pr	cytosol;mitochondrion;nucleus;other cell co
A0A0G2JFR7	Ribosome production factor 1 OS=Mus musculus OX=10090 GN=Rpf1 PE=1 S	17	2	2	2	212	23,8	9,54	RNA metabolism OR transcription;other metabolic processes	nucleus
Q8R2M2	Deoxynucleotidyltransferase terminal-interacting protein 2 OS=Mus musculu	28	17	24	17	758	84,2	6,44		nucleus;other cell component
Q9D8M4	60S ribosomal protein L7-like 1 OS=Mus musculus OX=10090 GN=Rpl7l1 PE=	10	2	2	2	246	28,5	10,64	RNA metabolism OR transcription;other metabolic processes;developmental proc	cytosol;translational apparatus;nucleus
Q9CQF7	Prefoldin 1 OS=Mus musculus OX=10090 GN=Pfdn1 PE=1 SV=1	43	7	29	3	122	14,2	6,81	other biological processes	other cell component
D3YXV8	Leukocyte receptor cluster member 8 homolog (Fragment) OS=Mus musculu	12	2	2	2	131	14,5	4,78		
Q8BWY3	Eukaryotic peptide chain release factor subunit 1 OS=Mus musculus OX=100	43	16	43	16	437	49	5,71	cell organization and biogenesis;protein metabolism;RNA metabolism OR transcri	cytosol;other cell component
Q3UFM6	Splicing factor ESS-2 homolog OS=Mus musculus OX=10090 GN=Ess2 PE=1 S	18	7	11	7	480	52,6	6,7		nucleus
F6U6X3	Dual-specificity kinase OS=Mus musculus OX=10090 GN=Dyrk1a PE=1 SV=1	12	6	7	6	725	81,5	8,79	protein metabolism;other metabolic processes	
Q9CWQ8	Cytosolic arginine sensor for mTORC1 subunit 1 OS=Mus musculus OX=1009	5	2	2	2	331	36,6	4,98	other biological processes	cytosol;other cell component
Q922Q2	Serine/threonine-protein kinase RIO1 OS=Mus musculus OX=10090 GN=Riol	14	8	11	8	567	64,9	6,57	protein metabolism;RNA metabolism OR transcription;other metabolic processes;	cytosol;other cell component
Q5U5M8	Biogenesis of lysosome-related organelles complex 1 subunit 3 OS=Mus mus	23	4	7	4	195	20,4	4,92	cell organization and biogenesis;stress response;transport;developmental proces	cytosol;ER/Golgi;other cell component

E9Q557	Desmoplakin OS=Mus musculus OX=10090 GN=Dsp PE=1 SV=1	3	8	9	8	2883	332,7	6,8	cell adhesion;cell organization and biogenesis;protein metabolism;other metaboli	plasma membrane;other membranes;cytoskeleton;other cell component	other molecular function	Pf00681, Pf17902, Pf18373	109620	ENSMUSG00000054889	Dsp	Neutrophil degranulation; Formation of the cornified envelope;	Lung fibrosis; Alpha 6 beta 4 integrin signaling pathway	0	29,57	14,22	9,67285E-01	6,65721E-01	1,15379E+00	1,41627E+00	7,96932E-01	8,23637E-01	7,85024E-01	7,57493E-01	6,37498E-01	9,50099E-01	#########	-0,20925	7,90750E-01	-0,33870585	0,74899025	Dsp
Q8BP67	60S ribosomal protein L24 OS=Mus musculus OX=10090 GN=Rpl24 PE=1 SV=	45	9	44	9	157	17,8	11,25	cell cycle OR cell proliferation;cell organization and biogenesis;protein metabolism	cytosol;ER/Golgi;translational apparatus;other cell component	nucleic acid binding activity;other molecular function	Pf01246	68193	ENSMUSG00000098274	Rpl24	Major pathway of rRNA processing in the nucleolus and cytoso	Cytoplasmic ribosomal proteins	0	31,59	18,17	8,39720E-01	1,02579E+00	1,53889E+00	7,73871E-01	8,21729E-01	8,71498E-01	9,47261E-01	6,54611E-01	6,19034E-01	8,42417E-01	#########	-0,21304	7,86964E-01	-0,34562992	0,68419748	Rpl24
Q02257	Junction plakoglobin OS=Mus musculus OX=10090 GN=Jup PE=1 SV=3	6	3	3	3	745	81,7	6,14	cell adhesion;cell organization and biogenesis;developmental processes;other bio	plasma membrane;other membranes;cytosol;cytoskeleton;nucleus;other cell component	other molecular function	Pf00514	16480	ENSMUSG00000001552	Jup	Neutrophil degranulation; RHOA GTPase cycle; RHOB GTPase c	Wnt signaling pathway	0	19,12	9,17	1,04327E+00	7,26220E-01	1,00083E+00	1,26273E+00	9,66942E-01	8,45376E-01	7,31308E-01	7,69249E-01	7,07647E-01	8,76616E-01	#########	-0,21396	7,86039E-01	-0,34732684	1,31989581	Jup
P19182	Interferon-related developmental regulator 1 OS=Mus musculus OX=10090	20	8	12	8	449	49,9	7,18	cell organization and biogenesis;developmental processes;other biological proces	nucleus;other cell component	other molecular function	Pf04836, Pf05004	15982	ENSMUSG00000001627	Ifrd1		Hypertrophy model	0	16,22	16,84	8,40975E-01	1,00012E+00	1,25647E+00	1,02120E+00	8,81235E-01	8,65791E-01	9,56279E-01	7,67658E-01	6,27064E-01	6,95954E-01	#########	-0,21745	7,82549E-01	-0,35374626	1,31534825	Ifrd1
Q80V76	Pygopus 2 OS=Mus musculus OX=10090 GN=Pygo2 PE=1 SV=1	23	6	9	6	405	41	7,28	cell-cell signaling;cell organization and biogenesis;developmental processes;signa	nucleus	enzyme regulator activity;other molecular function	Pf00628	68911	ENSMUSG00000047824	Pygo2	Formation of the beta-catenin:TCF transactivating complex		0	38,52	17,57	7,79926E-01	6,93501E-01	6,86224E-01	1,44084E+00	1,39951E+00	6,84524E-01	7,30757E-01	6,91981E-01	7,74473E-01	1,01783E+00	#########	-0,22009	7,79913E-01	-0,35861416	0,57875685	Pygo2
T1ECW4	RNA-binding protein with multiple-splicing OS=Mus musculus OX=10090 GN=	13	2	2	2	191	20,7	7,78		other membranes	nucleic acid binding activity	Pf00076	19663	ENSMUSG00000031586	Rbpms		mRNA processing; Exercise-induced circadian regulation	0	7,15	8,4	1,11676E+00	1,01060E+00	9,34261E-01	9,80489E-01	9,57899E-01	7,99343E-01	8,43043E-01	7,25054E-01	6,96365E-01	8,33403E-01	#########	-0,22056	7,79442E-01	-0,35948732	3,03063656	**Rbpms**
P14131	40S ribosomal protein S16 OS=Mus musculus OX=10090 GN=Rps16 PE=1 SV	70	13	47	13	146	16,4	10,21	protein metabolism;RNA metabolism OR transcription;other metabolic processes;	cytosol;translational apparatus	nucleic acid binding activity;other molecular function	Pf00380	20055	ENSMUSG00000037563	Rps16	Major pathway of rRNA processing in the nucleolus and cytoso	Cytoplasmic ribosomal proteins	0	25,61	21,12	9,26312E-01	9,88448E-01	1,41422E+00	7,10326E-01	9,60692E-01	8,80398E-01	9,53933E-01	6,57641E-01	5,58697E-01	8,39779E-01	#########	-0,22191	7,78090E-01	-0,36199176	0,84884617	Rps16
Q80YS6	Actin filament-associated protein 1 OS=Mus musculus OX=10090 GN=Afap1	2	2	2	2	731	80,6	8,68	other biological processes	cytosol;cytoskeleton;other cell component	cytoskeletal activity;other molecular function	Pf00169	70292	ENSMUSG00000029094	Afap1			0	19,31	19,02	8,88890E-01	1,10637E+00	1,26992E+00	9,60802E-01	7,74021E-01	9,85236E-01	7,34124E-01	7,53162E-01	5,78670E-01	8,23433E-01	#########	-0,22507	7,74925E-01	-0,36787113	1,14157327	Afap1
Q6KCD5	Nipped-B-like protein OS=Mus musculus OX=10090 GN=Nipbl PE=1 SV=1	4	9	11	9	2798	315,3	7,91	cell cycle OR cell proliferation;cell organization and biogenesis;DNA metabolism;s	cytosol;nucleus;other cell component	other molecular function	Pf12765, Pf12830	71175	ENSMUSG00000022141	Nipbl	Cohesin Loading onto Chromatin		0	2,99	8,27	9,63466E-01	1,03842E+00	1,01289E+00	1,00760E+00	9,77632E-01	8,26659E-01	7,63807E-01	8,23303E-01	6,68562E-01	7,83750E-01	#########	-0,22678	7,73216E-01	-0,37105644	4,01806364	**Nipbl**
Q9EPQ8	Transcription factor 20 OS=Mus musculus OX=10090 GN=Tcf20 PE=1 SV=3	2	3	4	2	1987	215,6	8,98	other biological processes	nucleus	nucleic acid binding activity;other molecular function	Pf13771	21411	ENSMUSG00000041852	Tcf20		Matrix metalloproteinases	0	6,75	16,38	9,74770E-01	1,04858E+00	9,04802E-01	1,07814E+00	9,93711E-01	7,87667E-01	6,74221E-01	8,37121E-01	6,22099E-01	9,40024E-01	#########	-0,22777	7,72226E-01	-0,37290452	2,11597956	**Tcf20**
Q80ZU5	Coiled-coil domain-containing protein 181 OS=Mus musculus OX=10090 GN=	3	2	4	2	509	59,2	5,33		cytoskeleton;other cell component	cytoskeletal activity		74895	ENSMUSG00000026578	Ccdc181			0	23,78	24,01	1,14525E+00	6,77402E-01	1,27406E+00	1,05600E+00	8,47287E-01	7,12366E-01	5,39656E-01	8,67361E-01	7,03744E-01	1,03047E+00	#########	-0,22928	7,70719E-01	-0,37572394	0,89275252	Ccdc181
Q9JJY4	Probable ATP-dependent RNA helicase DDX20 OS=Mus musculus OX=10090	3	2	2	2	825	91,7	6,74	cell organization and biogenesis;RNA metabolism OR transcription;other metaboli	cytosol;nucleus;other cell component	nucleic acid binding activity;other molecular function	Pf00270, Pf00271	53975	ENSMUSG00000027905	Ddx20	snRNP Assembly	mRNA processing	0	15,83	12,58	7,99652E-01	1,00455E+00	1,23039E+00	1,04071E+00	9,24693E-01	7,36278E-01	8,47981E-01	8,64108E-01	6,29998E-01	7,33837E-01	#########	-0,23756	7,62441E-01	-0,39130328	1,67967316	Ddx20
Q9CYZ7	Transcription factor OS=Mus musculus OX=10090 GN=Tfdp1 PE=1 SV=1	18	2	2	2	290	32,5	4,96	other biological processes	nucleus;other cell component	nucleic acid binding activity;other molecular function	Pf02319, Pf08781	21781	ENSMUSG00000038482	Tfdp1		G1 to S cell cycle control	0	32,14	19,4	8,57846E-01	5,59977E-01	1,25661E+00	1,36505E+00	9,60522E-01	9,89127E-01	7,46922E-01	7,82399E-01	6,78348E-01	5,96671E-01	#########	-0,24131	7,58693E-01	-0,39841116	0,7802624	Tfdp1
P62270	40S ribosomal protein S18 OS=Mus musculus OX=10090 GN=Rps18 PE=1 SV	53	10	51	10	152	17,7	10,99	protein metabolism;other metabolic processes	cytosol;translational apparatus;nucleus;other cell component	nucleic acid binding activity;other molecular function	Pf00416	20084	ENSMUSG00000008668	Rps18	L13a-mediated translational silencing of Ceruloplasmin express	Cytoplasmic ribosomal proteins	0	13,29	27,12	1,03486E+00	9,68780E-01	1,17972E+00	8,10012E-01	1,00663E+00	8,25492E-01	1,00139E+00	5,98094E-01	4,98268E-01	8,68505E-01	#########	-0,24165	7,58350E-01	-0,39906345	1,23426512	Rps18
O08789	Max-binding protein MNT OS=Mus musculus OX=10090 GN=Mnt PE=2 SV=2	7	3	4	3	591	63,2	9,13	other biological processes	nucleus	nucleic acid binding activity;other molecular function	Pf00010	17428	ENSMUSG00000000282	Mnt			0	57,56	27,13	6,66044E-01	6,08271E-01	1,03908E+00	1,98591E+00	7,00690E-01	6,36856E-01	6,48622E-01	6,61243E-01	7,21724E-01	1,12309E+00	#########	-0,24169	7,58306E-01	-0,39914737	0,39466152	Mnt
A2AVJ7	Ribosome-binding protein 1 OS=Mus musculus OX=10090 GN=Rrbp1 PE=1 S	68	93	321	93	1464	158,3	9,19	transport	other membranes;ER/Golgi		Pf05104	81910	ENSMUSG00000027422	Rrbp1			0	23,45	9,79	1,26764E+00	8,24405E-01	7,32422E-01	9,64407E-01	1,21112E+00	7,04147E-01	8,33500E-01	7,34334E-01	6,37909E-01	7,56411E-01	#########	-0,26674	7,33260E-01	-0,44760285	1,38698918	Rrbp1
A0A087WPW5	Kinectin OS=Mus musculus OX=10090 GN=Ktn1 PE=1 SV=1	5	6	13	5	1252	143,7	6,13	transport;other biological processes	other membranes;ER/Golgi	cytoskeletal activity	Pf05104	16709	ENSMUSG00000021843	Ktn1			0	70,31	53,56	3,91105E-01	1,53703E+00	1,94200E+00	7,26523E-01	4,03339E-01	1,34061E+00	3,71070E-01	6,62460E-01	4,38481E-01	7,81580E-01	#########	-0,28116	7,18841E-01	-0,47625542	0,34152276	Ktn1
A0A2I3BPN6	TATA-box-binding protein-associated factor 4 OS=Mus musculus OX=10090	6	4	6	4	1030	104,1	9,85	RNA metabolism OR transcription;other metabolic processes;developmental proc	cytosol;nucleus;other cell component	signal transduction activity or receptor binding;nucleic acid binding activity;othe	Pf05236, Pf07531		ENSMUSG00000039117	Taf4			0	4,52	16,86	1,05535E+00	1,03511E+00	9,94618E-01	9,54611E-01	9,60312E-01	8,59982E-01	6,59589E-01	6,88992E-01	5,57077E-01	8,08977E-01	#########	-0,28508	7,14923E-01	-0,48413989	2,94711702	**Taf4**
G3X925	Pyruvate kinase OS=Mus musculus OX=10090 GN=Pklr PE=1 SV=1	4	4	57	2	574	62,2	7,09	other metabolic processes;stress response;other biological processes	cytosol	kinase activity;other molecular function	Pf00224, Pf02887	18770	ENSMUSG00000041237	Pklr		Glycolysis and gluconeogenesis; Purine metabolism	0	25,54	7,1	7,68160E-01	1,18268E+00	6,77809E-01	1,16511E+00	1,20625E+00	6,57035E-01	6,94455E-01	7,89025E-01	6,80074E-01	7,15907E-01	#########	-0,29270	7,07299E-01	-0,49960721	1,44257703	Pklr
S4R1W8	Glyceraldehyde-3-phosphate dehydrogenase (Fragment) OS=Mus musculus	78	4	29	2	55	6,2	5,91			other molecular function	Pf00044		ENSMUSG00000057666	Gapdh			0	27	11,03	7,87841E-01	1,11872E+00	6,40275E-01	1,24467E+00	1,20850E+00	6,18508E-01	6,16274E-01	6,22700E-01	7,57707E-01	5,73251E-01	#########	-0,36231	6,37688E-01	-0,64907706	1,70358222	**Gapdh**
G5E8V9	Arfaptin-1 OS=Mus musculus OX=10090 GN=Arfip1 PE=1 SV=1	34	11	31	11	373	41,5	6,76	transport;other biological processes	other membranes;cytosol;ER/Golgi	other molecular function	Pf06456	99889	ENSMUSG00000074513	Arfip1			0	48,99	10,49	5,59055E-01	1,40128E+00	5,45493E-01	8,76146E-01	1,61803E+00	5,85278E-01	5,72874E-01	6,26228E-01	5,50947E-01	4,68092E-01	#########	-0,43932	5,60684E-01	-0,83474098	1,08853606	Arfip1
